# Detection of subclonal L1 transductions in colorectal cancer by long-distance inverse-PCR and Nanopore sequencing

**DOI:** 10.1038/s41598-017-15076-3

**Published:** 2017-11-06

**Authors:** Barun Pradhan, Tatiana Cajuso, Riku Katainen, Päivi Sulo, Tomas Tanskanen, Outi Kilpivaara, Esa Pitkänen, Lauri A. Aaltonen, Liisa Kauppi, Kimmo Palin

**Affiliations:** 1 Genome-Scale Biology Research Program, Research Programs Unit, University of Helsinki, Helsinki, 00014 Finland; 2 Department of Biochemistry and Developmental Biology, Medicum, University of Helsinki, Helsinki, 00014 Finland; 3 Department of Medical and Clinical Genetics, Medicum, University of Helsinki, Helsinki, 00014 Finland; 4 Department of Biosciences and Nutrition, Karolinska Institutet, Stockholm, SE-171 77 Sweden

## Abstract

Long interspersed nuclear elements-1 (L1s) are a large family of retrotransposons. Retrotransposons are repetitive sequences that are capable of autonomous mobility via a copy-and-paste mechanism. In most copy events, only the L1 sequence is inserted, however, they can also mobilize the flanking non-repetitive region by a process known as 3′ transduction. L1 insertions can contribute to genome plasticity and cause potentially tumorigenic genomic instability. However, detecting the activity of a particular source L1 and identifying new insertions stemming from it is a challenging task with current methodological approaches. We developed a long-distance inverse PCR (LDI-PCR) based approach to monitor the mobility of active L1 elements based on their 3′ transduction activity. LDI-PCR requires no prior knowledge of the insertion target region. By applying LDI-PCR in conjunction with Nanopore sequencing (Oxford Nanopore Technologies) on one L1 reported to be particularly active in human cancer genomes, we detected 14 out of 15 3′ transductions previously identified by whole genome sequencing in two different colorectal tumour samples. In addition we discovered 25 novel highly subclonal insertions. Furthermore, the long sequencing reads produced by LDI-PCR/Nanopore sequencing enabled the identification of both the 5′ and 3′ junctions and revealed detailed insertion sequence information.

## Introduction

Long interspersed nuclear elements (LINE)-1, also known as L1 elements, are active mobile repeat elements in the human genome. Germline L1 polymorphic insertions are suggestive of L1’s contribution to genomic diversity^1^. Nevertheless, somatic L1 insertions can drive tumorigenesis and high L1 expression is emerging as a common trait of several cancers^2^. For an L1 to be potentially active, it needs to be a full-length element with a 5′ promoter and two intact open reading frames, ORF1 and ORF2, terminating with a 3′ polyadenylation signal.

Out of more than 500,000 copies of L1 sequences in the human reference genome only 90 to 100 are potentially active^3,4^. Upon activation, the L1 is transcribed into an RNA intermediate, which is then translated into two proteins, ORF1p and ORF2p. The L1 mRNA, ORF1p and ORF2p form a ribonucleoprotein complex that nicks the DNA at the target location via the endonuclease function of ORF2p. The polyadenylated 3′ end of the L1 mRNA anneals to the T-rich region of the target site, which then acts as a primer for reverse transcription^5^. Thus, this process, referred to as target-primed reverse transcription (TPRT), integrates L1 sequence into the target region with a signature polyA sequence marking initiation of reverse transcription. TPRT sometimes is accompanied by a process called twin-priming that leads to inversion of the L1 inserts. This results when one overhang produced on the target region by ORF2p-mediated staggered double-strand cleavage serves as a T-rich or polyT primer for TPRT, and the other anneals to the internal L1 mRNA sequence serving as an internal primer^6^.

**Figure 1:**
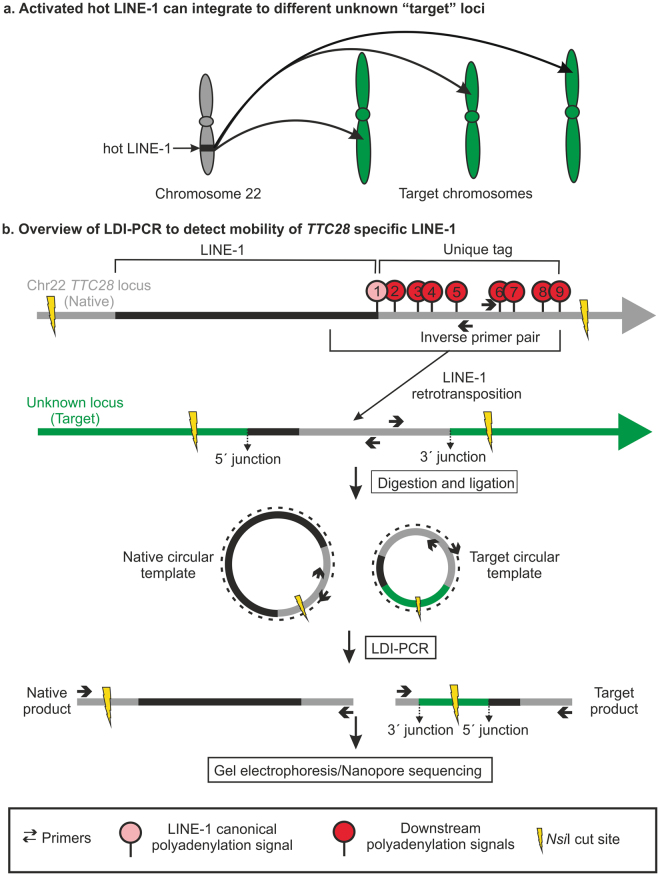
LDI-PCR based method to detect the activity of a hot L1. (**a**) Schematic showing mobility of hot L1 from the *TTC28* locus upon activation. (**b**) LDI-PCR to detect 3′ transduction arising from the L1 at *TTC28*: Schematic representation of a hypothetical *TTC28* specific L1 retrotransposition including transduction of 3′ flanking region or the “unique tag” (=region between the canonical polyadenylation signal and an alternative polyadenylation signal downstream), into an unknown target locus. *Nsi*I produces restriction fragments of two different sizes that are self-ligated to form a circular template. Upon LDI-PCR, an inverse primer pair directed at the unique tag produces a native product and an insertion-specific target product. In addition to *Nsi*I, two further restriction enzymes (*Pst*I and *Sac*I) and primer pairs (not depicted here) were used; see Materials and Methods for details.

Not all potentially active L1 elements contribute equally to retrotransposition events however, and only few “hot” L1 elements are responsible for most retrotranspositions^3^. These hot L1 elements are of considerable interest as they show frequent somatic insertions in various types of cancers (Fig. 1a)^7–11^. Particularly, an L1 located in the first intron of *TTC28* (chromosomal position 22q12.1) (L1Base ID: 135^12^; dbRIP ID: 2000144^13^), also shown to be an active element in an *in vitro* retrotransposition assay^3^, is highly active in colorectal cancer^9,11^. It was possible to identify this particular L1 (hereafter referred to as *TTC28* L1) as the source of the insertions by using short paired-end read sequencing based on the mobilization of its non-repetitive (unique) 3′ flanking sequence, via a mechanism known as 3′ transduction^14^. 3′ transduction occurs when the canonical 3′ polyadenylation signal of the source L1 is weak, causing the transcription machinery to skip it and to continue transcribing the non-repetitive region downstream in the 3′ flanking region until it reaches a stronger polyadenylation/termination signal. Consequently, some of this unique sequence is included in the RNA intermediate and subsequently incorporated into the new chromosomal location, thereby serving as a unique sequence tag that reveals the L1’s origin.

Although whole genome sequencing (WGS) can identify 3′ transductions, detecting these L1-mediated insertions is still a great challenge, principally due to the repetitive nature of L1 sequences and our limited capacity to sequence long fragments of DNA. Several next-generation sequencing strategies targeting young L1s or 3′ transduced regions have been developed (ATLAS^15^, L1-seq.^16^, RC-seq.^17^, transduction-specific ATLAS^18^, TIP-seq.^19^) and used to identify somatic L1 insertions, but they are quite extensive if the aim is to simply assess the activity of a few L1 loci. Furthermore, all current methods targeting L1 insertions are limited in their capacity to simultaneously resolve full insertion sequence. Thus we have developed a direct molecular approach to detect 3′ transductions from specific L1s and hence monitor their activity; this method requires no prior knowledge of the insertion target regions. We apply long-distance inverse (LDI)-PCR^20^ to a particular source L1 (*TTC28* specific L1 in this study) by targeting inverse primers to its frequently transduced 3′ flanking sequence (here referred to as the “unique tag”) (Fig. 1b). Note, however, that L1 insertions exhibiting 3′ transduction represent a quarter of the total L1 insertions emanating from any particular source^11^.

We utilized previously published WGS data^21^ to select two colorectal tumour samples with high number of *TTC28* L1 3′ transductions (hereafter referred simply as insertions) for our proof-of-concept analysis and comparison. By selective amplification of the transduced region using LDI-PCR (Fig. 1b) followed by Nanopore sequencing, we were able to detect 14 out of 15 previously detected insertions, and additionally identified several highly subclonal insertions not detected by WGS. Long reads produced by Nanopore sequencing allowed detailed sequence analysis of the LDI-PCR products, including full inserted sequence and identification of hallmarks of retrotransposition, such as target-site duplications and deletions, polyA sequence and genomic aberrations such as inversions and deletions^22^.

## Results

### Detection of somatically acquired *TTC28* insertions using LDI-PCR/Nanopore sequencing

**Figure 2:**
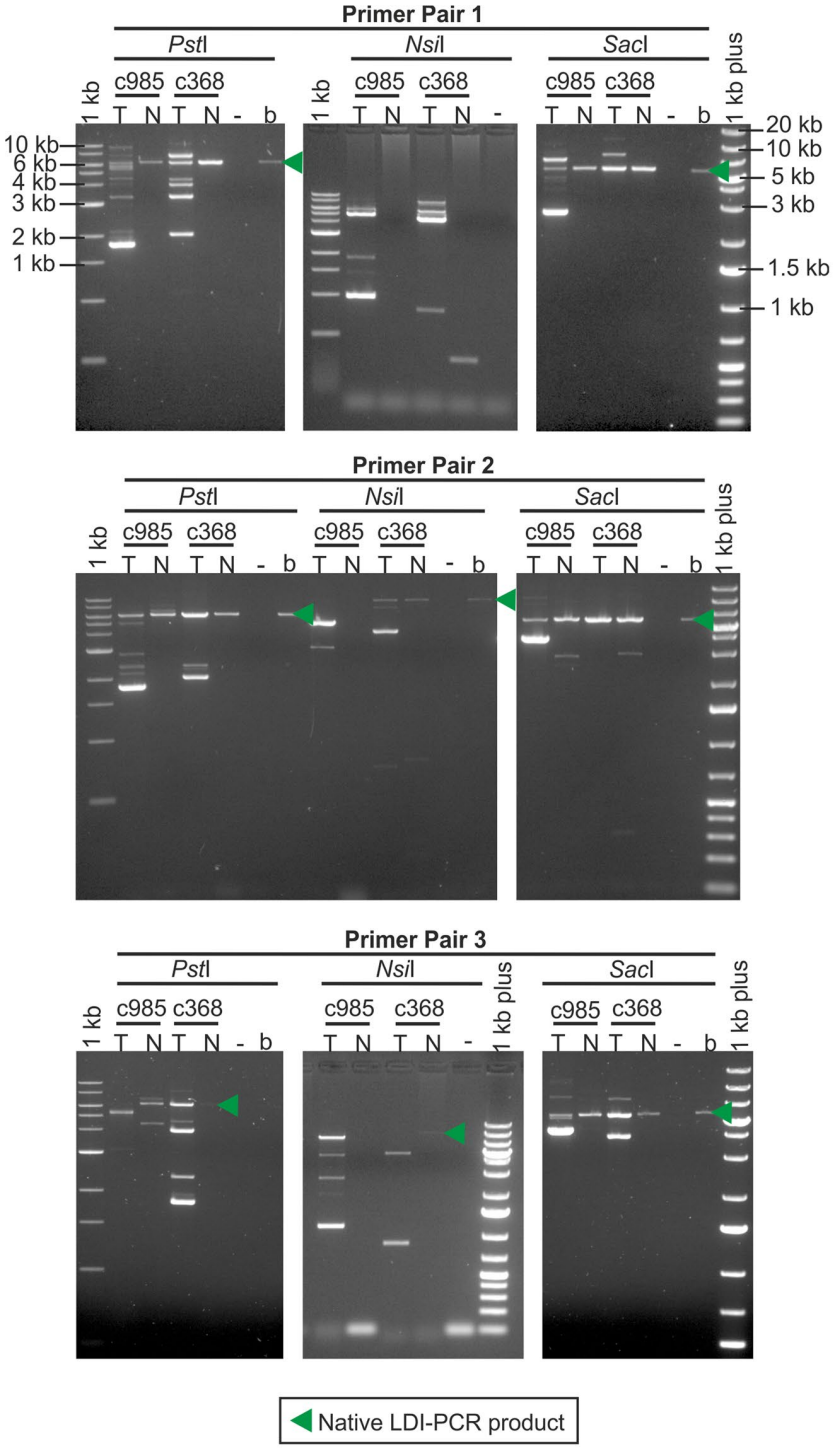
Agarose gel image of the LDI-PCR products. LDI-PCR using three different inverse primer pairs and restriction enzymes on two colorectal tumours (c985T and c368T) and their matching normal (c985N and c368N) DNA samples. The “native” PCR product of expected size and several additional tumour-specific products representing different putative 3′ transduction targets were detected. (Sizes corresponding to native LDI-PCR products: *Pst*I~6.3 kb, *Nsi*I~10.2 kb, *Sac*I~5.6 kb.) Digested/self-ligated blood genomic DNA sample of an unrelated individual was run in the lane labelled “b” and PCR without any template in “−”.

We selected the L1 located in the first intron of *TTC28* for LDI-PCR analysis, as it had been previously reported to be highly active in colorectal cancer^9^. We performed LDI-PCR (Fig. 1b) on DNA obtained from two tumour samples, selected from a previously reported WGS data set^21^ and on DNA from the corresponding normal samples, using three restriction enzymes (*Pst*I, *Nsi*I (Fig. 1b) and *Sac*I) and three different primer pairs (Supplementary Table S1). The LDI-PCR product corresponding to the source *TTC28* L1 (the “native PCR product”) was observed in almost all the samples digested with *Pst*I and *Sac*I (Fig. 2) but only sporadically observed in *Nsi*I digested samples (e.g. as seen in LDI-PCR using primer pair 2, Fig. 2). In addition to the native PCR product, tumour samples exhibited additional PCR products which indicated mobilization of the *TTC28*-specific unique tag via L1 3′ transduction to different target locations. In order to identify the target location of each insertion, we sequenced the LDI-PCR products from the tumour samples using a single-molecule sequencing technique, Nanopore sequencing.

**Figure 3:**
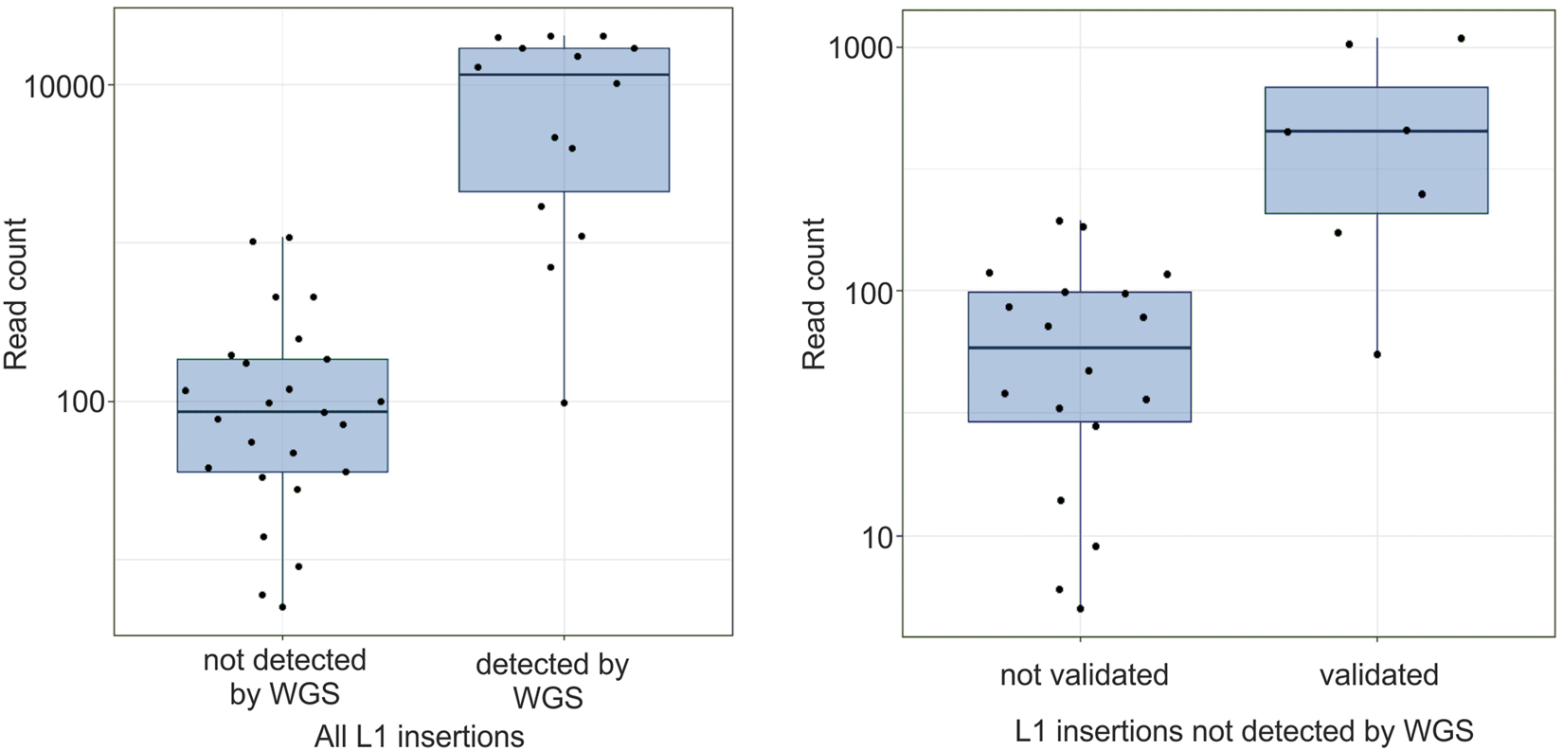
Read counts for insertions called by LDI-PCR.py. On the left, boxplots of read counts for insertions that were either detected (n = 14) or undetected (n = 25) by WGS. On the right, boxplots of read counts for insertions undetected by WGS that were either validated (n = 7) or unvalidated (n = 18). For better visualization, data are presented on a base-10 log scale.

Nanopore sequencing generated 644,669 reads and no bias in read frequency towards particular read lengths was apparent (Supplementary Fig. S1). We developed and applied the LDI-PCR software (LDI-PCR.py) to identify *TTC28* specific 3′ transductions in both tumour samples (c985T and c368T). After filtering the LDI-PCR.py calls, we were able to identify 14 out of 15 previously detected insertions. Additionally, we detected 25 novel insertions not identified by WGS, despite visual inspection of the paired-end read data (Supplementary Fig. S2) (Table 1). Notably, these 25 novel candidate insertions were supported by fewer reads than the 14 WGS-detected insertions (p = 2.43 × 10^−6^ by Wilcoxon rank-sum test) (Table 1 and Fig. 3). The median number of supporting reads was 98 for novel candidate insertions and 11,428 for WGS-detected insertions, suggesting that the novel insertions are subclonal events and therefore difficult to detect by 40x WGS.

**Table 1:** Insertions detected by LDI-PCR/Nanopore sequencing. Note that one insertion was predicted by WGS but not by LDI-PCR/Nanopore and not included in the table. TSM = target-site modification; TP = twin-priming; del. = deletion; dup. = duplication; Read count = Number of LDI-PCR.py reads supporting each insertion, if one insertion was detected by more than one enzyme or primer pair, only the reaction with higher number of reads was included; *includes internal duplication; **some sequence was missing.

Sample	Target cordinates		L1 insertion targets	TSM	Size of TSM	*TTC28* L1 mediated 3′ transduction		Strand	TP	Insertion (bp)	Read count	Validated	WGS detected	WGS read count
	5′ junction	3′junction				start	end							
c985T	1:195769724	1:195769709	intergenic	dup.	16	29065826	29066121	+	Present	295	3984	yes	yes	15
	4:93280482	4:93280454	*GRID2*	dup.	28	29065650	29065893	+	Absent	243	4658	no	yes	20
	4:155900401	4:155900387	intergenic	dup.	14	29064889	29065912	+	Present	1015	1705	no	yes	9
	4:183051382	4:183051401	*AC*1*0814*2.*1*	del.	18	29065306	29065448	−	Absent	142	10103	no	yes	8
	7:146783241	7:146783223	*CNTNAP*2	dup.	18	29065369	29065912	−	Present	536	19889	no	yes	29
	7:152661949	7:152661940	intergenic	dup.	9	29065283	29065887	−	Present	455	20107	no	yes	19
	12:33708291	12:33708277	intergenic	dup.	14	29065722	29066121	−	Present	377	16909	no	yes	20
	2:78612537	2:78612530	intergenic	dup.	8	29065453	29066118	−	Present	665	5	no	no	0
	3:99147126	3:99147111	intergenic	dup.	16	29065138	29066126	−	Present	984	47	no	no	0
	4:90987152	4:90987149	intergenic	dup.	4	29065287	29066121	+	Absent	834	99	no	no	0
	6:74978206	6:74978187	*RP11–554D15*.*1*	dup.	20	29065521	29066118	+	Absent	597	9	no	no	0
	8:111856478	8:111856457	intergenic	dup.	22	29064270	29066083	−	Present	857	6	no	no	0
	14:99070525	14:99070523	intergenic	dup.	3	29065558	29065912	−	Absent	354	55	yes	no	1
	16:26220799	16:26220798	intergenic	dup.	2	29065976	29066118	+	Absent	142	182	no	no	0
	16:5902138	16:5902158	*RP11–420N*3.*2*	del.	19	29065747	29065912	−	Absent	165	72	no	no	1
c368T	1:115147190	1:115147187	*DENND2C*	dup.	4	29065647	29065893	−	Absent	246	12753	no	yes	18
	2:182004540	2:182004515	*AC*1*04820*.*2*	dup.	26	29065285	29065886	+	Present	783*	20217	no	yes	12
	2:229159082	2:229159075	intergenic	dup.	8	29065578	29065908	−	Absent	330	704	no	yes	16
	6:70787202	6:70787188	*COL19A1*	dup.	15	29065808	29066032**	NA	Present	>223	17007	no	yes	21
	6:133527459	6:133527443	intergenic	dup.	17	29065683	29065887	−	Absent	204	98	no	yes	9
	8:88681299	8:88681304	*AF121898*.*3*	del.	4	29065471	29065887	+	Absent	416	1095	no	yes	6
	12:128116403	12:128116405	*RP11–526P6*.*1*	del.	1	29065730	29066121	−	Absent	391	15099	no	yes	10
	2:50947578	2:50947612	*NRXN1*	del.	33	29065849	29066032**	NA	NA	>183	98	no	no	0
	2:129889238	2:129889240	intergenic	del.	1	29065945	29066121	−	Absent	176	193	no	no	0
	4:44621421	4:44621515	intergenic	del.	93	29065305	29065912	−	Absent	607	33	no	no	0
	5:8665955	5:8665942	intergenic	dup.	14	29065849	29066091	+	Absent	242	451	yes	no	0
	5:83347372	5:83347360	*EDIL3*	dup.	13	29065762	29066121	+	Present	340	78	no	no	0
	5:119565858	5:119565843	intergenic	dup.	16	29065419	29065899	+	Present	455	14	no	no	0
	6:112763097	6:112763084	intergenic	dup.	14	29065288	29066118	+	Present	671	38	no	no	0
	7:152870668	7:152870685	intergenic	del.	16	29065491	29065912	−	Present	306	457	yes	no	0
	8:114925191	8:114925178	intergenic	dup.	14	29064763	29065887	−	Absent	1124	1020	yes	no	0
	8:107979180	8:107979169	intergenic	dup.	12	29064270	29066121	−	Present	1055	28	no	no	0
	10:101386670	10:101386662	intergenic	dup.	9	29065621	29065782**	NA	NA	>161	36	no	no	0
	10:107557372	10:107557435**	intergenic	NA	NA	29065912	29066032**	NA	NA	>120	86	no	no	1
	12:33100097	12:33100084	intergenic	dup.	14	29065445	29066118	−	Present	670	248	yes	no	0
	14:79638932	14:79638931	*NRXN3*	dup.	2	29065974	29066121	−	Absent	147	172	yes	no	1
	18:1233989	18:1233975	intergenic	dup.	15	29065387	29066121	−	Present	735	117	no	no	0
	X:108351909	X:108351907	intergenic	dup.	3	29065437	29065893	−	Absent	456	119	no	no	0
	Y:15633117	Y:15633103	intergenic	dup.	15	29065877	29066121	−	Absent	244	1084	yes	no	1

In order to validate the novel candidate insertions, we first performed conventional PCR and Nanopore sequencing and were able to validate two insertions (Y:15633117 and 14:79638932). Subsequently we performed allele-specific PCR and Sanger sequencing on another ten insertions, of which we successfully sequenced the insertion-to-target junctions for six of them (Table 1, Supplementary Fig. S2). Again, non-validated insertions were supported by fewer reads as compared to validated insertions (p = 7.53 × 10^–4^ by Wilcoxon rank-sum test) (Fig. 3). Furthermore, the insertion located at Y:15633117 was confirmed with both methods.

### Analysis of consensus sequence generated by LDI-PCR/Nanopore sequencing to elucidate insertion characteristics

**Figure 4:**
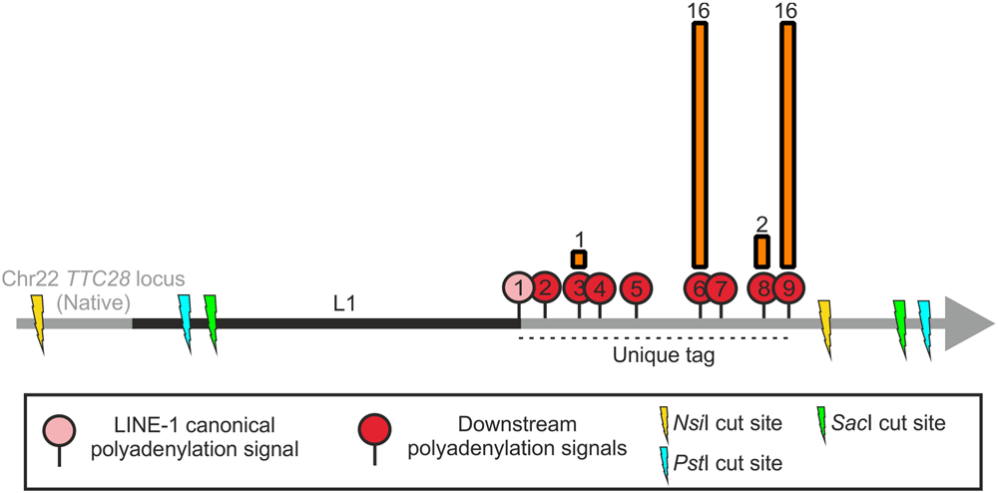
Polyadenylation signal of choice for *TTC28* L1 mediated 3′ transduction. 8 polyadenylation signals following the L1 canonical polyadenylation signal (no. 2–9) were present in the unique tag assessed for *TTC28* L1 3′ transduction. Most of the 3′ transductions identified in this study terminated utilizing the 6th and 9th polyadenylation signal instead of the L1’s canonical polyadenylation signal (no. 1). The height of the orange bars indicates the frequency of L1 3′ transductions terminating at each polyadenylation signal; number of termination events is given above the bars.

Consensus sequences, generated from the LDI-PCR.py calls after filtering, provided us with complete inserted sequences for 35 out of 39 L1 insertions which were analysed to decipher the insertion mechanism and retrotransposition hallmarks (Table 1). 8/35 consensus sequences contained short alignment gaps, ranging from 3–10 bp, due to mismatches affecting the alignment. Most of these alignment gaps (7) arose from insertions supported by less reads indicating, as expected, that higher number of reads improves consensus accuracy. 29 out of 35 insertions involved a target-site duplication while 6 insertions involved a target-site deletion. We also detected 2 target-site duplications and 1 target-site deletion in insertions with incomplete sequence (Table 1). The size of L1 insertions ranged from 142 bp to 1124 bp with an average insertion size of 493 bp. All detected L1 insertions were heavily truncated at their 5′ end, the majority (~77%) to the extent that they were composed of the 3′ transduced region only, without any L1 sequence (also known as “orphan transductions”). We were able to locate the terminal sequence in all 35 insertions with complete inserted sequence. Variation in the 3′ most genomic coordinate (Table 1) of the L1 3′ transduction suggested the use of more than one polyadenylation signal. By following the criteria explained in the methods section to determine which polyadenylation signal was preferred, we observed that more than 90% of the *TTC28* L1 3′ transductions used either the 6th or the 9th polyadenylation signal instead of the canonical polyadenylation signal (Fig. 4, Supplementary Table S2). Preference for the 9th polyadenylation signal is in agreement with polyadq prediction^23^ (Supplementary Table S2), a web-based polyadenylation signal prediction tool. However, the 6th polyadenylation signal was defined as a false signal by polyadq, even though it had the highest score among the ATTAAA polyadenylation signals (Supplementary Table S2).

Strand inversion of the inserted sequence due to twin-priming was observed in 16 out of 35 insertions for which complete inserted sequence was available. One more twin-priming event was observed in three insertions with incomplete sequence (Table 1). Furthermore, the point of inversion was identifiable in all 16 cases of twin-priming. We were able to resolve the 5′ junction for 29 out of 35 L1 insertions, as remaining 6 contained short alignment gaps as mentioned earlier. In addition we were also able to resolve 5′ junction of 4 insertions that had incomplete inserted sequence. Out of these total 33 insertions 28 of them showed microhomology of 1–13 bp at the 5′ junction. Microhomology of 1–5 bp was also observed in 10 out of 16 twin-priming inversion point.

**Figure 5:**
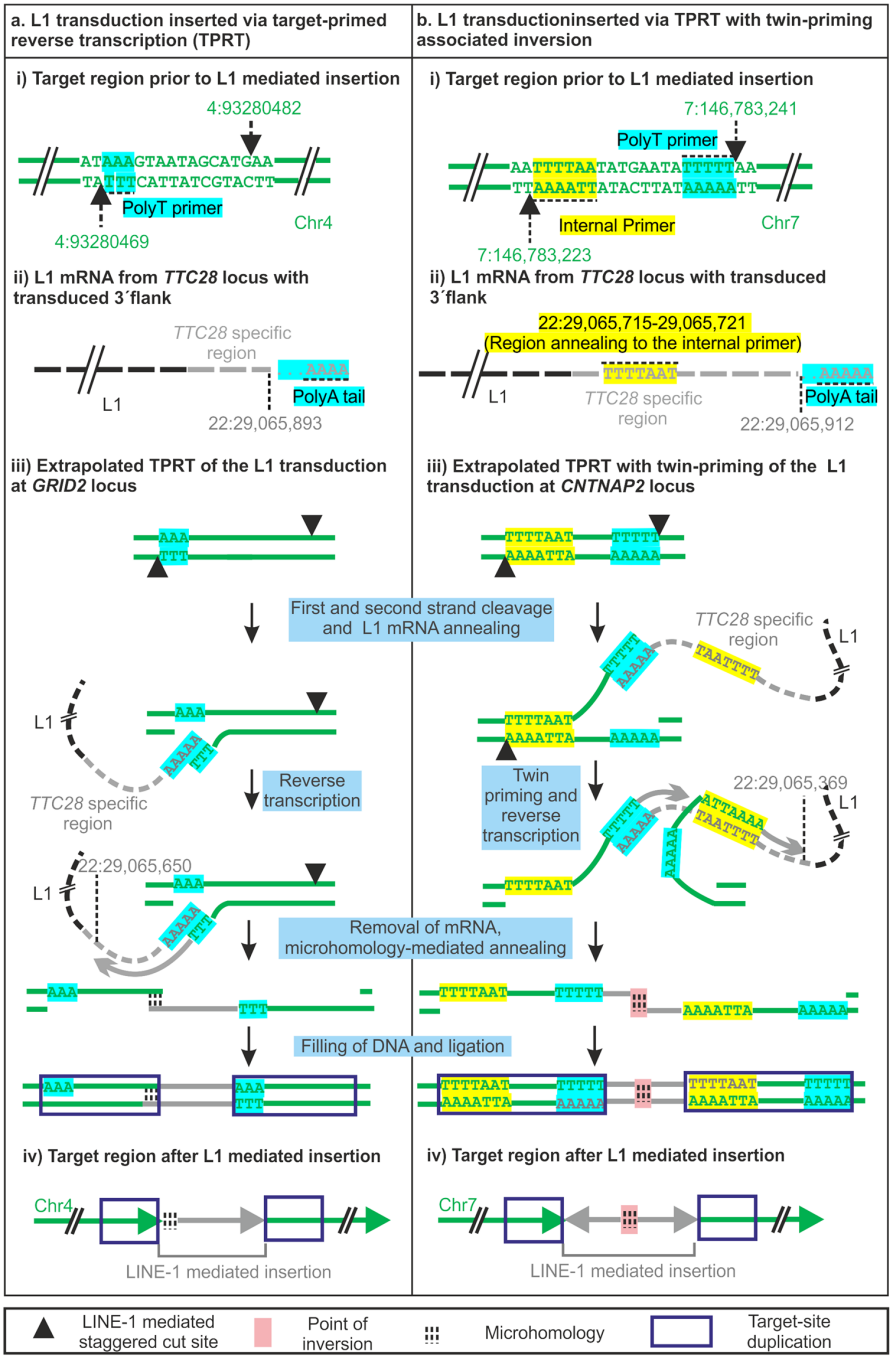
Extrapolated mechanism of L1 insertion at (**a**) *GRID2* locus and (**b**) *CNTNAP2* locus In both (**a**) and (**b**) (i) shows locations of staggered double-stranded cleavage in the target region with PolyT primer in the 3′ overhang and an internal primer (when used) in the 5′ overhang (**b**). (ii) Extrapolated L1 mRNA with the *TTC28* 3′ unique tag. Regions complementary to the polyT primer and internal primer at target site is highlighted by the same colour scheme. (iii) Schematic representation of the TPRT (**a**) and twin-priming (**b**) mechanism of *TTC28* specific 3′ transduction in the *GRID2* (**a**) and *CNTNAP2* (**b**) locus. Figures are not drawn to scale.

We then used information on the inserted sequences to better understand the integration process of the L1-derived sequence. To extrapolate the stepwise mechanism of L1 insertion, we selected two insertions displaying two different well-characterized modes of insertion: (a) TPRT, exemplified by the L1 insertion at *GRID2* locus on chromosome 4 and (b) TPRT with twin-priming, exemplified by the L1 insertion at *CNTNAP2* locus in chromosome 7 (Supplementary Fig. S3, Fig. 5). L1-transduced sequence was inserted on the “+” strand of *GRID2* target locus and on the “−” strand of *CNTNAP2* locus (Supplementary Fig. S3). Target site duplication (TSD) observed at both loci indicated L1 endonuclease mediated staggered double-stranded cleavage in the target region (Fig. 5ai,bi). This staggered double-strand cleavage at both target loci generated a T-rich overhang. We infer that these T-rich overhangs produced on the “−” and “+” strand of *GRID2* and *CNTNAP2* target loci, respectively, annealed to the polyA tail at the end of the L1 mRNA (Fig. 5aii,bii) and were used as a polyT primer for reverse transcription Fig. 5ai,bi; stepwise mechanism illustrated in Fig. 5aiii,biii). In addition to polyT priming, reverse transcription at the *CNTNAP2* target locus most likely also used as an internal primer the other overhang generated, causing an inversion of the inserted sequence (Fig. 5biii). Upon close examination we found that the region of inversion (22:29,065,715–29,065,721) did in fact show nucleotide complementarity with the 5′ overhang generated by ORF2p on the reverse strand (7:146,783,223) (Fig. 5bii,biii). This twin-priming (first by a polyT primer and then by an internal primer) at the *CNTNAP2* locus led to reverse-transcription at two different locations causing strand inversion (Fig. 5biii and biv). We also observed a deletion of 3 base pairs (22:29,065,722–29,065,724) at the inversion site and microhomology of 3 base pairs between the reverse transcribed sequence produced by the polyT primer and the internal primer at the point of inversion (Fig. 5b). Microhomology was also observed at the 5′ junction of L1 insertion at the *GRID2* target locus. Thus, LDI-PCR/Nanopore sequencing provided complete information for most of the somatic L1 insertions, enabling us to analyse the insertion process in great detail.

### Comparison of the local assembly of WGS data and the consensus sequences generated by LDI-PCR/Nanopore sequencing

In order to interpret the advantages and disadvantages of long read sequencing, we compared the local assembly of paired-end read data to the LDI-PCR/Nanopore consensus sequences of those insertions in tumour sample c985T that were detected by both methods. After local assembly, we were able to reconstruct four out of seven insertions into one contiguous fragment (contig) (Supplementary Table S3) with defined parameters (Supplementary Table S4). However, the remaining three insertions were represented in two contigs and therefore the sequence in between was lost. The length of missed sequences, ranging from 88 bp to 614 bp, was estimated based on the genomic coordinates of both insertion junctions. However, in cases where the twin priming point of inversion was located within the missed sequence, estimation of the insertion size was not possible, as shown for chr7:152661937 (Supplementary Table S3). All insertions with missing sequence information were longer than 400 bp and were the longest insertions based on the consensus sequence analysis of Nanopore data. The deviation of the estimated length of the insertions ranged from 0–7 bp and 10/14 target coordinates were consistently predicted by both analysis (Supplementary Table S3). The remaining four target coordinates were located right after a tract of Ns where the consensus polyA/T junction was predicted, henceforth, target sequence after the polyA/T was probably missed by the local assembly. In conclusion, local assembly of paired-end read data was of limited value in reconstructing full insertions but also interpreting polyA/T tails in our study, thus hampering the elucidation of insertion features.

## Discussion

Due to the repetitive nature of L1 elements and their abundance in the human genome, it is possible to determine the lineage or source of the L1 insertions either (a) when the insertion contains signature single nucleotide polymorphisms associated with the source L1^10^ or (b) when it involves 3′ transduction of the unique flanking sequence^9,11^. Thus, in order to determine the activity of a particular source L1, one needs to scan the genome of interest for either of the aforementioned signs. Assessing the activity of a particular source L1, in particular when active in a tumour type, can be of great interest, as these L1 insertions can cause tumorigenic events or serve as a clinical biomarker^24^. However, the specific detection of L1 3′ transductions and/or sequencing the whole length of L1 insertions remains a great challenge. In this study we used a LDI-PCR based assay to study the activity of a source L1 at the *TTC28* locus, previously shown to be highly active in colorectal cancer, and tested it on two colorectal cancer samples with already published whole genome sequencing data^21^.

By applying LDI-PCR in conjunction with Nanopore sequencing to as low as 300 ng of tumour DNA per sample, we were able to detect 14 out of 15 previously identified *TTC28* L1 mediated 3′ transductions, and also discovered 25 3′ transductions not detected by WGS (Table 1). The read count difference between the two groups (WGS-detected versus not detected) indicated that these insertions could be subclonal events and thus not detectable with 40x sequencing. Furthermore, high coverage provided us with enough data to reconstruct accurate consensus sequences, which permitted analyses of full inserted sequence in 90% of the insertions. About 45% of the 3′ transductions analysed showed strand inversion due to twin-priming. We speculate that this high incidence of twin-priming in *TTC28* L1 3′ transduction is due to (a) the nature of nucleotide sequence downstream of the L1 3′ end, possibly due to many small stretches of Ts present which could complement with a stretch of As generated by the ORF2p endonuclease action on the target region which in turn can be used as the second or “internal” primer leading to twin-priming, or (b) detection of more L1 insertions with these inversion properties than by conventional methods due to the sequencing of the whole inserted sequence. We were also able to sequence the entire source *TTC28* L1 (Supplementary Fig. S4). In addition, we were able to identify that *TTC28* 3′ transductions terminated preferentially on two polyadenylation signals (Fig. 4), one predicted to be a true signal with the highest score (9th), and another defined as a false signal (6th) (Supplementary Table S2) by polyadq.

One insertion predicted by WGS was not identified in this study, however. LDI-PCR and Nanopore sequencing could have been hindered due to the formation of a secondary structure due to homology between the insertion polyT and a nearby (567 bp upstream) polyA present in the reference genome.

LDI-PCR/Nanopore success principally relies on (i) a careful selection of the restriction enzymes so as to produce PCR-amplifiable target templates and (ii) the design of multiple primer pairs covering several downstream polyadenylation signals predicted by tools such as polyadq. In our initial pilot LDI-PCR/Nanopore sequencing experiment using one restriction enzyme (*Sac*I) and two primer pairs on one sample (c985T), we were able to detect only 4 insertions out of 8 detected by WGS analysis. Detection sensitivity was substantially improved by updated sequencing chemistry, the use of two additional restriction enzymes and by designing more primers that covered additional polyadenylation signals in a well-dispersed manner.

LDI-PCR follows a similar targeting strategy as transduction-specific ATLAS (TS-ATLAS)^18^. However, LDI-PCR allows the amplification of both 5′ and 3′ junctions and sequencing the entire insertion simultaneously, which cannot be accomplished by any other L1 targeted approach or by WGS. The only region that remains unsequenced in a single read is the nucleotide bases in between the primer pairs, however this limitation can be minimized by reducing the distance between the primers and using more than one set of primers. Furthermore, the inverse PCR primers at the unique sequence enable the detection of L1 orphan transductions, which are not detected by other targeted sequencing techniques such as ATLAS, L1-seq, RC-seq or TIP-seq.^15–17,19^. Additionally, LDI-PCR/Nanopore sequencing is customizable for any full-length L1 allowing the implementation of this assay on a handful of “hot” L1 elements that contributes to a large fraction of 3′ transductions in a cancer genome^11^.

To conclude, we demonstrated that LDI-PCR/ Nanopore sequencing is suitable for sequencing the entire L1 insertion and for detecting highly subclonal events. Consequently, applying LDI-PCR in conjunction with Nanopore sequencing in larger sample sets and different tumour types enables a more detailed characterization of L1 insertions providing new insights into L1 biology and cancer genetics.

## Material and Methods

### Samples

The colorectal adenocarcinoma (CRC) samples utilized in this study were obtained from a population based series of 1042 CRCs previously described^25,26^. The tumours were fresh frozen and the corresponding normal tissues were obtained from blood (c985T) and from colon tissue (c368T). The study was reviewed and approved by the Ethics committee of the Hospital district of Helsinki and Uusimaa, Finland. A signed informed consent or authorization from the National Supervisory Authority for Welfare and Health was obtained for all the samples.

### LDI-PCR and Nanopore sequencing

#### Digestion and ligation of DNA

To detect insertions arising from *TTC28* L1, genomic DNA was separately digested by three restriction enzymes: *Sac*I, *Pst*I and *Nsi*I. *Sac*I and *Pst*I make a 5′ cut at ORF1 of the L1 and a 3′ cut downstream of the unique tag producing a native restriction fragment of 5.7 kb and 6.3 kb respectively, whereas *Nsi*I makes a 5′ cut 3.1 kb upstream of the intact L1 sequence and a 3′ cut downstream of the unique tag generating a native restriction fragment of ~10.2 kb (Fig. 1b, Supplementary Table S5). L1 retrotransposition usually involves a 5′ truncation, and the average L1 insert size including the 3′ transduced region is 1000 bp^11^. Therefore it is unlikely that the somatically acquired L1 insertion will contain the same *Sac*I or *Pst*I cut sites as the source region, hence increasing the likelihood that the target site restriction fragment is of different size compared to the native one. The infrequent cases of full-length somatic L1 insertion can be captured by the digestion library produced by *Nsi*I. At least one out of three enzymes always generated a predicted restriction fragment of less than 8.2 kb in all the WGS predicted targets (Supplementary Table S5). Digested DNA was then self-ligated using T4 DNA ligase (Thermo) to form circular templates for LDI-PCR.

#### Primer Design and Optimization

Inverse primers for LDI-PCR were designed on the unique tag, that is, the genomic region between the canonical polyadenylation signal of the L1 and the next strongest polyadenylation signal on its 3′ flanking region (Fig. 1b, Supplementary Table S2). Strength of polyadenylation signals at this region was estimated using polyadq scores^23^ (http://rulai.cshl.edu/tools/polyadq/polyadq_form.html). Primers were designed using Primer3 (http://primer3.ut.ee) and their specificity was checked using NCBI Primer-BLAST (available at http://www.ncbi.nlm.nih.gov/tools/primer-blast/). Since there were several polyadenylation signals at the 3′ flanking region of the L1 (Fig. 1b), we designed several primer pairs between the canonical polyadenylation signal (marked 1) and following downstream polyadenylation signals. Distance between the primers was kept as short as possible (≤51bp).

#### LDI-PCR

1.25 ng circular templates generated by restriction enzyme digestion and T4 ligation were used in LDI-PCR as eight replicates (Supplementary Fig. S5) as previously described^20^ using three primer pairs (Supplementary Table S1). These PCR products were then analysed on a 1% agarose gel and purified using NucleoSpin Gel and PCR Clean-up kit (Macherey-Nagel). Replicate reactions showing reproducible patterns of PCR amplification were pooled, and sequenced using MinION (Oxford Nanopore Technologies). Replicates that did not show reproducibile patterns were discarded and the reaction was repeated again.

#### Oxford Nanopore MinION™ sequencing

LDI-PCR products from 18 different reactions (with three different primer pairs, three different restriction enzymes and from two tumour samples) were pooled into nine different barcodes in equal molarity. Tape Station 2200 (Agilent Technologies) was used to estimate the relative molarity based on the fragment distribution in each reaction. Libraries were constructed according to the manufacturer’s instructions using SQK-LSK108 and EXP-NBD103 sequencing and barcoding kits (Oxford Nanopore Technologies). Equal molarity was preserved throughout the protocol. The MinION Flow cell (FLO-MIN106) was run for 6 hours using MinION Mk1B. The raw signal from MinION was basecalled with ONT Albacore Sequencing Pipeline Software (version 1.0.2). The reads passing base calling were aligned to GRCh37 genome reference augmented with viral and 1000 genomes decoy sequences. The alignment was performed with bwa mem v0.7.12 using option -x ont2d^27^.

#### LDI- PCR software

We separated the reads produced by different samples, PCR primers and restriction enzymes by using the sequencing barcodes and comparing the read mapping with restriction enzyme cut sites in the reference genome. To systematically detect the insertions, we developed the LDI-PCR.py software which identifies reads that display hallmark features of LDI-PCR products (Fig. 1b). Hallmarks of a LDI-PCR product in this experiment are: that it contains at least one alignment to *TTC28* L1 (22:29060420–29067335) and at least one alignment to a single target locus in the genome. The read can have multiple supplementary alignments to the target locus but only if located in close proximity of each other (maximum distance between alignments 100 kb). All considered alignments had to have mapping quality of at least 20. The insertion breakpoint was defined by the location where the alignment switched from *TTC28* L1 to the target locus and vice versa for each hallmark read. All insertion breakpoints were clustered together allowing a maximum gap of 3 kb from all reads defining the different insertion locations. The most frequent genomic coordinate was called as the insertion breakpoint defining each LDI-PCR insertion call. The software is available at https://github.com/kpalin/LDI-PCR-call and https://github.com/kpalin/ampcorrect.

Furthermore, LDI-PCR insertion calls had to be supported by at least 5 reads and, to filter away random ligation products generated by LDI-PCR, the insertion breakpoint had to be located at least 35 bp from the closest corresponding restriction enzyme cut site. Moreover, due to barcoding ligation crosstalk produced by EXP-NBD103 barcoding kit (Oxford Nanopore Technologies), several insertion calls were present in both samples. In order to circumvent this issue, in cases where the insertion call was present in two samples, only the calls coming from the sample that contained at least 95% of the reads were included in further analyses. Insertions in mitochondria and unplaced sequence were filtered away. All insertions that fulfilled the abovementioned criteria in at least one reaction were defined as candidate insertions and selected further for consensus sequence analysis of the insertion characteristics. The reads from the candidate insertions were processed with ampCorrect, a Nanopore read correction method similar to nanocorrect^28^, to obtain accurate consensus sequences for the amplicons. Briefly, ampCorrect uses sumaclust^29^ (http://metabarcoding.org/sumatra) to cluster the reads, requiring 60% sequence similarity and poaV2 -do global^30^ to align multiple reads. The consensus sequence is treated as corrected sequence of the analysed amplicon. The processed sequences were aligned to the human reference genome using bwa mem with default parameters. The analysis of the insertion characteristics was performed on a consensus sequence which was constructed from 20 random candidate insertion call reads using UCSC BLAT (https://genome.ucsc.edu/cgi-bin/hgBlat) (Consensus sequence of all insertions analysed are provided in FASTA format in Supplementary dataset 1).

#### Determining the polyadenylation signal used for each L1 3′ transduction

Since the transcription termination and polyadenylation occurs 10–30 bp downstream of the selected polyadenylation signal^31^, we analysed how many 3′ coordinates of the L1 insertions were located within a 10–30 bp window downstream of each of the polyadenylation signal (stop signal window) located downstream of the L1 sequence (Supplementary Table S2). 9 out of 35 L1 insertion terminal sequence did not fall within any of the 8 defined windows, and were assigned to the closest available window (maximum distance was 9 bp) (Table 1 and Supplementary Table S2).

#### Statistical analysis

To test for differences in read counts, we used Wilcoxon rank-sum test with continuity correction in R version 3.3.2. Read counts refer to numbers of reads supporting each candidate insertion called by LDI-PCR.py. In cases where the same candidate insertion was detected in different reactions (three different restriction enzymes and three different primer pairs) the reaction with higher read count was used.

### Whole genome sequencing analysis

We utilized the WGS dataset described in Katainen *et al*.^21^ to select for LDI-PCR and Nanopore sequencing those colorectal cancer samples with a high number of somatically acquired insertions originating from *TTC28*. Structural variant breakpoints located at the 3′ end of the *TTC28* L1 were extracted to calculate the number of transductions. The 3′ end of the L1 was defined as GRCh37 coordinates 22:29065455–29066124. To examine whether the novel candidate insertions were detected by WGS or not, we performed a thorough visual inspection of the paired-end read data using BasePlayer^32^.

In order to compare LDI-PCR Nanopore sequencing results to WGS data we performed local assembly of the WGS data. We selected those chimeric reads and discordant read pairs that aligned within 1kb upstream and downstream the predicted insertion breakpoint, with the exception of one insertion (chr15:97602708) where, due to a long target site deletion, a 3kb window was used. The local assembly of the reads was performed using Velvet 1.2.10^33^. All hash lengths within default parameters (11,13,15,17,19,21,23,25,27,29,31) were tested and the hash length that produced the longest and most contiguous contig was selected for each insertion^33^ (Supplementary Table S4). We aligned the assembled contigs with UCSC Blat (https://genome.ucsc.edu/cgi-bin/hgBlat) to the GRCh37 genome reference.

### Validation of highly subclonal L1 insertions by conventional PCR

Two approaches were used to validate novel candidate insertions detected by LDI-PCR but not by WGS: (i) First, primer pairs were designed on the target genomic region across the insertion breakpoint and sequenced by Nanopore. The library was prepared as described in the section “Oxford Nanopore MinION™ sequencing”. This approach included all candidate subclonal insertions, however only 3/25 novel candidate insertions were validated (ii) Second, primers were designed based on the consensus sequence, with one primer at the target site and the other primer at the inserted sequence, followed by Sanger sequencing of the resulting PCR product; this was performed for 10 selected novel candidate insertions. Primer pairs were designed with primer3Plus (http://www.bioinformatics.nl/cgi-bin/primer3plus/primer3plus.cgi). Primer sequences are in Supplementary Table S6. Sanger sequencing was performed by the Biomedicum Sequencing Unit, Helsinki, on ABI Prism 3130xl Genetic Analyzer (Applied Biosystems) using BigDye Terminator v3.1 cycle sequencing kit (Applied Biosystems). Sequences were manually analysed using FinchTV v.1.4.0 (http://www.freewarefiles.com/FinchTV_program_17782.html).

### Data availability

The datasets generated during and analysed during the current study are available from the corresponding author on request.

## Electronic supplementary material

Supplementary Information

Supplementary dataset

## References

[1] Beck CR (2010). LINE-1 retrotransposition activity in human genomes. Cell.

[2] Burns KH (2017). Transposable elements in cancer. Nat Rev Cancer.

[3] Brouha B (2003). Hot L1s account for the bulk of retrotransposition in the human population. Proc Natl Acad Sci USA.

[4] Lander ES (2001). Initial sequencing and analysis of the human genome. Nature.

[5] Luan DD, Korman MH, Jakubczak JL, Eickbush TH (1993). Reverse transcription of R2Bm RNA is primed by a nick at the chromosomal target site: a mechanism for non-LTR retrotransposition. Cell.

[6] Ostertag EM, Kazazian HH (2001). Twin priming: a proposed mechanism for the creation of inversions in L1 retrotransposition. Genome Res.

[7] Lee E (2012). Landscape of somatic retrotransposition in human cancers. Science.

[8] Miki Y (1992). Disruption of the APC gene by a retrotransposal insertion of L1 sequence in a colon cancer. Cancer Res.

[9] Pitkanen E (2014). Frequent L1 retrotranspositions originating from *TTC28* in colorectal cancer. Oncotarget.

[10] Scott EC (2016). A hot L1 retrotransposon evades somatic repression and initiates human colorectal cancer. Genome Res.

[11] Tubio JM (2014). Mobile DNA in cancer. Extensive transduction of nonrepetitive DNA mediated by L1 retrotransposition in cancer genomes. Science.

[12] Penzkofer T (2017). L1Base 2: more retrotransposition-active LINE-1s, more mammalian genomes. Nucleic Acids Res.

[13] Wang J (2006). dbRIP: a highly integrated database of retrotransposon insertion polymorphisms in humans. Hum Mutat.

[14] Moran JV, DeBerardinis RJ, Kazazian HH (1999). Exon shuffling by L1 retrotransposition. Science.

[15] Badge RM, Alisch RS, Moran JV (2003). ATLAS: a system to selectively identify human-specific L1 insertions. Am J Hum Genet.

[16] Ewing AD, Kazazian HH (2010). High-throughput sequencing reveals extensive variation in human-specific L1 content in individual human genomes. Genome Res.

[17] Baillie JK (2011). Somatic retrotransposition alters the genetic landscape of the human brain. Nature.

[18] Macfarlane CM (2013). Transduction-specific ATLAS reveals a cohort of highly active L1 retrotransposons in human populations. Hum Mutat.

[19] Rodic N (2015). Retrotransposon insertions in the clonal evolution of pancreatic ductal adenocarcinoma. Nat Med.

[20] Pradhan B (2016). Detection and screening of chromosomal rearrangements in uterine leiomyomas by long-distance inverse PCR. Genes Chromosomes Cancer.

[21] Katainen R (2015). CTCF/cohesin-binding sites are frequently mutated in cancer. Nat Genet.

[22] Gilbert N, Lutz-Prigge S, Moran JV (2002). Genomic deletions created upon LINE-1 retrotransposition. Cell.

[23] Tabaska JE, Zhang MQ (1999). Detection of polyadenylation signals in human DNA sequences. Gene.

[24] Ardeljan D, Taylor MS, Ting DT, Burns KH (2017). The Human Long Interspersed Element-1 Retrotransposon: An Emerging Biomarker of Neoplasia. Clin Chem.

[25] Aaltonen LA (1998). Incidence of hereditary nonpolyposis colorectal cancer and the feasibility of molecular screening for the disease. N Engl J Med.

[26] Salovaara R (2000). Population-based molecular detection of hereditary nonpolyposis colorectal cancer. J Clin Oncol.

[27] Heng, L. Aligning sequence reads, clone sequences and assembly contigs with BWA-MEM. *arXiv* (2013).

[28] Loman NJ, Quick J, Simpson JT (2015). A complete bacterial genome assembled de novo using only nanopore sequencing data. Nat Methods.

[29] Mercier, C. B. F., Bonin, A. & Coissac, E. SUMATRA and SUMACLUST: fast and exact comparison and clustering of sequences. Programs Abstr SeqBio 27 (2013).

[30] Grasso C, Lee C (2004). Combining partial order alignment and progressive multiple sequence alignment increases alignment speed and scalability to very large alignment problems. Bioinformatics.

[31] Colgan DF, Manley JL (1997). Mechanism and regulation of mRNA polyadenylation. Genes Dev.

[32] Katainen, R. *et al*. BasePlayer: Versatile Analysis Software For Large-Scale Genomic Variant Discovery. *bioRxiv*, 10.1101/126482 (2017).

[33] Zerbino DR, Birney E (2008). Velvet: algorithms for de novo short read assembly using de Bruijn graphs. Genome Res.

