# Supplementary material for: Detection of subclonal L1 transductions in colorectal cancer by long-distance inverse-PCR and Nanopore sequencing
Source: Sci Rep. 2017 Nov 6;7:14521. doi: 10.1038/s41598-017-15076-3 (PMC5673974; doi:10.1038/s41598-017-15076-3)
Supplement: Supplementary file 1 — Supplementary Information [file 41598_2017_15076_MOESM1_ESM.pdf]

SUPPLEMENTARY INFORMATION:

Detection of subclonal L1 transductions in colorectal cancer by long-distance inverse-PCR and Nanopore sequencing

Barun Pradhan[1,2]†, Tatiana Cajuso[1,3]†, Riku Katainen[1,3], Päivi Sulo[1,3], Tomas Tanskanen[1,3], Outi Kilpivaara[1,3], Esa Pitkänen[1,3], Lauri A. Aaltonen[1,3,4], Liisa Kauppi[1,2]* and Kimmo Palin[1,3]*.

Supplementary table S1: Primers used for LDI-PCR

Primer Pair	Primer name	Sequence (5'-3')	Location
1	L1_001 (rev)	TTCACTAAGCATGTATGTGGAAAAC	chr22:29065758-29065782
	L1_002 (fwd)	CCCAAAATATACCCAATTACTGGCA	chr22:29065833-29065857
2	L1_048 (rev)	CAGTCACCTTTATTAGACATGTGG	chr22:29065330-29065353
	L1_049 (fwd)	TCCCAGAAGTGTGACCAGTTAG	chr22:29065405-29065426
3	L1_053 (rev)	TCTATGGCCATTTTATGGAATGAC	chr22:29066009-29066032
	L1_054 (fwd)	GCCAGATCAAGTAATCCTACTCAT	chr22:29066035-29066058

Supplementary table S2: Polyadq[1] strength scores of the polyadenylation signals of LINE-1 element in *TTC28* (#1) and subsequent following signals. The number in the first column refers to the polyadenylation signals depicted in **Figure 1b** and **Figure 4** with red lollipops.

S. No.	Polyadenylation signals	Location	Score	Prediction
1	AATAAA	Chr22:29065288	0.009	-
2	AATAAA	Chr22:29065340	0.219	-
3	AATAAA	Chr22:29065433	0.112	-
4	ATTAAA	Chr22:29065481	0.000	-
5	ATTAAA	Chr22:29065643	0.003	-
6	ATTAAA	Chr22:29065870	0.895	-
7	ATTAAA	Chr22:29065907	0.049	-
8	AATAAA	Chr22:29066078	0.282	+
9	AATAAA	Chr22:29066099	0.400	+

Supplementary table S3: Summary of characteristics of the insertions detected by local assembly of the paired-end read data in c985T. The length of missing sequence was estimated from genomic coordinates detected at the junctions of the insertions. TSM = Target-site modification; del. = deletion; dup. = duplication

Target co-ordinates		Contigs	Insertion co-ordinates (start-end)	TSM	TSM (bp)	Missed sequence (bp)
5´junction	3´ junction					
1:195769724	1:195769735	1	29065822-29066119	del.	10	0
4:93280482	4:93280483	1	29065653-29065889	no	-	0
4:155900401	4:155900410	2	29064889-29065912	del.	8	614
4:183051382	4:183051401	1	29065306-29065455	del.	18	0
7:146783241	7:146783223	2	29065372-29065909	dup.	18	88
7:152661937	7:152661940	2	29065691-29065889 & 29065510-29065295	del.	2	N/A
12:33708290	12:33708277	1	29065722-29066118	dup.	14	0

Supplementary table S4: Hash lengths used in the local assembly of the paired-end read data. All hash lengths within default parameters (11,13,15,17,19,21,23,25,27,29,31) were tested and the hash length that produced the longest and most contiguous contig was selected for each insertion (insertion length= 400, coverage cut off= 2 and expected coverage =10).

chr	Predicted breakpoint	Hash length
4	93280483	25
4	155900402	11
7	146783222	23
12	33708276	15
1	195769725	23
4	183051400	21
7	152661939	19
15	97602708	23

Supplementary table S5: Restriction fragment length made by all three restriction enzymes used in native (unique tag of *TTC28* L1) as well as target regions predicted by WGS analysis. Highlighted in grey predicted fragments longer than 15 kb and therefore less likely to be amplified.

Name of the sample	Location of the native and targets	Restriction fragment length (in base pairs)		
		***Sac*I (GAGCTC)**	***Pst*I (CTGCAG)**	***Nsi*I (ATGCAT)**
Present in all (Native)	chr22:29065288-29066104	5699	6344	10288
c985	chr1:195769724-195769735	>15kb	4990	817
	chr4:93280482-932804483	6870	9481	747
	chr4:155900401-155900410	>15kb	4080	2112
	chr4:183051382-183051401	3676	>15kb	5122
	chr7:146783223-146783241	2276	5147	4503
	chr7:152661937-152661940	10974	1405	>15kb
	chr12:33708277-33708290	4013	5041	8010
	chr15:97602700-97604688	>15kb	8828	8141
c368	chr1:115147190-115147209	11400	3093	7461
	chr2:182004550-182004552	>15kb	1418	3683
	chr2:229159060-229159060	>15kb	7085	7374
	chr6:70787198-70787200	3673	1493	9563
	chr6:133527432-133527432	>15kb	7436	6813
	chr21:10015974-10015974	>15kb	7039	3986

Supplementary table S6: Primers used for validation of novel L1 insertions detected by LDI-PCR/Nanopore sequencing in colorectal cancer samples c985T and c368T.

Primer name	Sequence (5´-3´)	Approach
16_26220797_F	TTCAAAGAGATATCTGCACTCCA	Nanopore validation
4_90987145_F	CTGTGTCATGGGACCAACAG	Nanopore validation
16_5902154_F	GAGCATTCCTTCCTGCTGTC	Nanopore validation
14_99070526_F	TGGAGCCTATGACCTTCTGTG	Nanopore validation
3_99147137_F	GGGCCAAACTCAGAATTTCA	Nanopore validation
6_74978246_F	TCAATGGGGCATATCACAGA	Nanopore validation
8_111856478_F	TGTCGCTATGGAATAATTTTAGATG	Nanopore validation
2_78612546_F	TTTCAAGCTTATTACACCAATCC	Nanopore validation
Y_15633117_F	CTTCAGGCCCTGTCTGACTC	Nanopore validation
8_114925191_F	TCAACATTCATACTTGTGGCATA	Nanopore validation
7_152870678_F	TTGAGGGATTTGTGATTGAGG	Nanopore validation

5_8665942_F	TGTGGTTATTTCCCCAGCTC	Nanopore validation
12_33100097_F	GATAAGAGGTCGGCACAAGG	Nanopore validation
2_129889240_F	TGTTTTGTTATTTGGAAAAGTAGGTG	Nanopore validation
14_79638933_F	CCTCTGCATAGGGAGCAAAC	Nanopore validation
18_1233989_F	TGTTTCCAGGAGTTTGTCCA	Nanopore validation
2_50947605_F	CATTTTCAGCCTGCACAAGA	Nanopore validation
10_107557376_F	AAGAAGCTACAAGCCCCTGA	Nanopore validation
5_83347384_F	AGACCTCCAGCAAACTCCAA	Nanopore validation
6_112763084_F	TCCCATCTCTTTCCCTCAGT	Nanopore validation
10_101386662_F	TCTTCCTGAAAAAGCCGTTG	Nanopore validation
8_107979184_F	TCAAAACAAAAACTCCAGTCCA	Nanopore validation
5_119565859_F	CCTAGAGTGCATTGGCCTGT	Nanopore validation
X_108351907_F	CCTGGGGCAACTCAATCTT	Nanopore validation
16_26220797_R	AGCACCCACTGCTGATCATT	Nanopore validation
4_90987145_R	CCAGCTCCCATAGATTTGGA	Nanopore validation
16_5902154_R	CAAGCATTGGTTGATCAGGA	Nanopore validation
14_99070526_R	ACCTCGCCATTGTAATGCTC	Nanopore validation
3_99147137_R	CACTCTTTTCTGTGCCCAATG	Nanopore validation
6_74978246_R	GGTGCATCTTCAAATGCTGA	Nanopore validation
8_111856478_R	CCTTCAATATTGCCTCTTTGC	Nanopore validation
2_78612546_R	GGTAGCCACTTGGAATAATTGG	Nanopore validation
Y_15633117_R	CAGAGCTGTGATCCACTCCA	Nanopore validation
8_114925191_R	TGCCTTGATCCAGACTTTTTC	Nanopore validation
7_152870678_R	TAAATGGCCAGAGCAGGAAG	Nanopore validation
5_8665942_R	TTGCCTAAAGCCTCAAAGTTTC	Nanopore validation
12_33100097_R	GATAAGAGGTCGGCACAAGG	Nanopore validation
2_129889240_R	GCACACAGTAATTTCTTTTCAGC	Nanopore validation
14_79638933_R	TGTGATAACAGCTTTGGAAGTGA	Nanopore validation
18_1233989_R	CACAAATCACTGAGGCAGAAA	Nanopore validation
2_50947605_R	TGCAGCAAGGAAGGAGAGTT	Nanopore validation
10_107557376_R	TGAGCCACAAGTGTCTGACC	Nanopore validation
5_83347384_R	TTTCTCTCTGGCTGCCCTTA	Nanopore validation
6_112763084_R	TCTTCAGAACCACCCAGAGA	Nanopore validation
10_101386662_R	CTGTCGCCTAGGCTGGAGT	Nanopore validation
8_107979184_R	AATGACCCAGCCTCTTCAGT	Nanopore validation
5_119565859_R	TCCACAAATCAAATGCAACAA	Nanopore validation
X_108351907_R	CTTCCTGATCCCTCTGCTTG	Nanopore validation
1_195769715_F	CCAAACATGTAGTAGTCTTGATTTGTA	Positive control
1_195769715_R	AAAAATTCAAGCATATGGAAAA	Positive control
15_97602708_F	tttccagaccccatcctTTA	Allele-specific PCR
15_97602708_R	GGCAGCTTCATGTTGACACA	Allele-specific PCR
Y_15633103_F	GGCCATTTTATGGAATGACAA	Allele-specific PCR
Y_15633103_R	TTTTCAATTTTGTTGGTTTTTCAA	Allele-specific PCR

8_114925178_F	ACTGAGAATGATGGTTTCCAA	Allele-specific PCR
8_114925178_R	GAGCTCAGTTAGCATTGCACA	Allele-specific PCR
7_152870685_F	TAGATAAGAACGTTTTTGTGAATTAAA	Allele-specific PCR
7_152870685_R	ATCCACCCCATCATCCAAT	Allele-specific PCR
5_8665942_F	ATAACAGCCCCAAGCAACAG	Allele-specific PCR
5_8665942_R	GGCCATTTTATGGAATGACAA	Allele-specific PCR
12_33100084_F	TGCTTGCATTAAAAATGTTAAGTT	Allele-specific PCR
12_33100084_R	CATTCTTTTGAACACCTTTGAATC	Allele-specific PCR
16_26220798_F	GGGCTGGGTAGATGTTGGTT	Allele-specific PCR
16_26220798_R	GGATTACTTGATCTGGCCATCTA	Allele-specific PCR
4_90987149_F	TCTTTTCTTCTTTTTCTCACAGTAAAT	Allele-specific PCR
4_90987149_R	TTTTGGCCCTAACTGGTCAC	Allele-specific PCR
16_5902158_F	GCCAGTAATTGGGTATATTTTGG	Allele-specific PCR
16_5902158_R	GCATTTGAGGCAGAGGAGAC	Allele-specific PCR
14_99070523_F	CATTTTTAATGCAAGCATATTAACC	Allele-specific PCR
14_99070523_R	AAGCTCTCCCTTTCTGCGTA	Allele-specific PCR
3_99147111_F	GGCAAAATAATCTCATAAGCACA	Allele-specific PCR
3_99147111_R	GCAGTGGGTGGTGATAGGAG	Allele-specific PCR

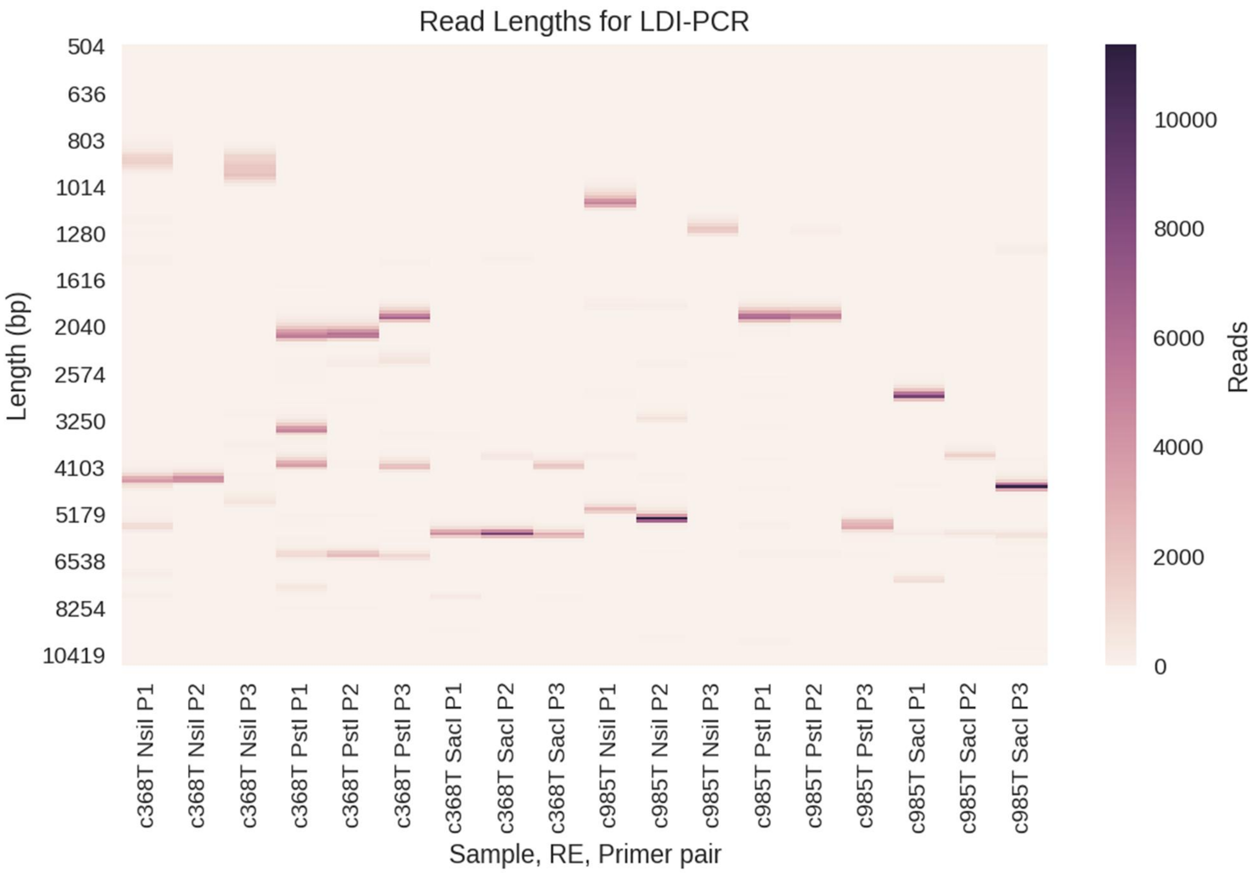

Supplementary figure S1: Read length histogram of LDI-PCR reads, Y-axis is split uniformly in log space to 200 histogram bins and bins are colored according to gradient. Read counts corresponding each sample, restriction enzyme and PCR primer pair are plotted separately. (Visualisation inspired by David Eccles https://github.com/gringer/bioinfscripts)

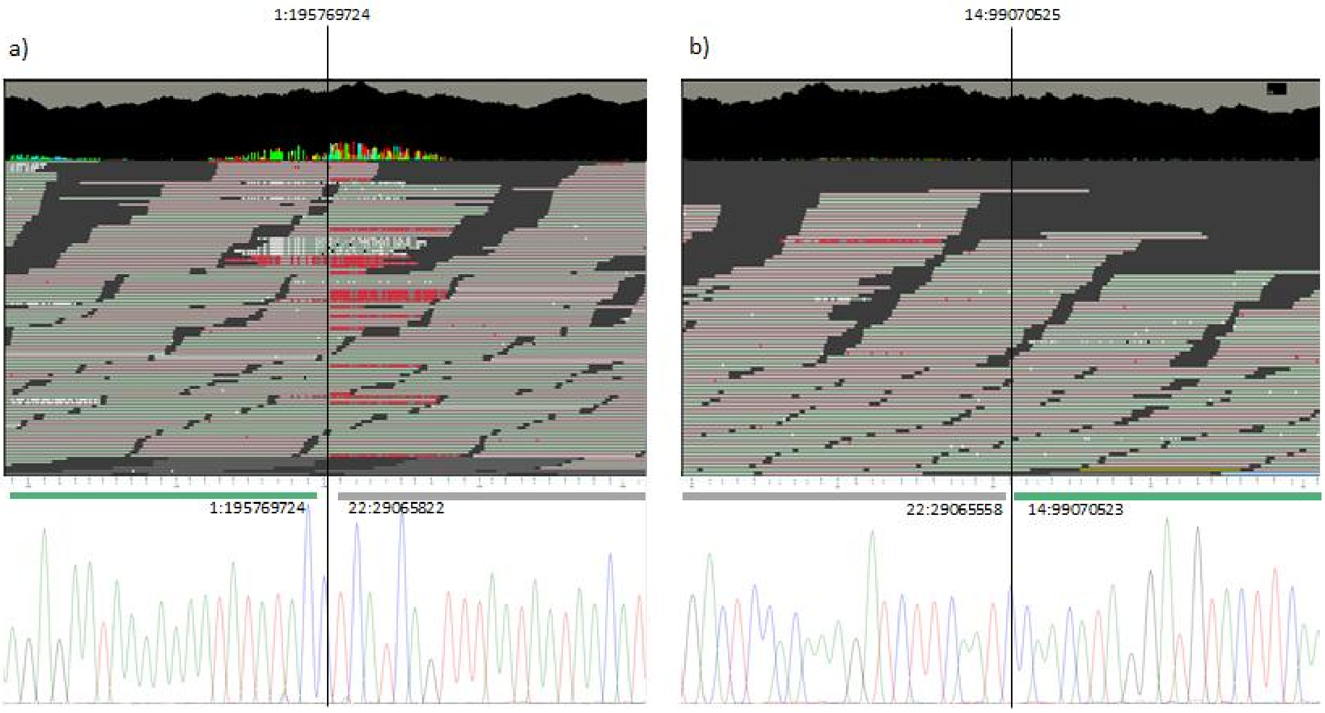

Supplementary figure S2: Example of one of the WGS-detected insertions (a) and one of the subclonal insertions not detected by WGS (b). Screenshots from BasePlayer[2] of the WGS data (top panels) show paired-end reads at the exact insertion breakpoint predicted by LDI-PCR/Nanopore and confirmed by Sanger sequencing. In the lower section of the figure; electropherograms of the junctions between the insertion and the target produced from allele-specific PCR and Sanger sequencing. a) Paired-end discordant reads aligning to the unique sequence of L1 *TTC28* can be observed in grey; also split reads encompassing the insertion breakpoint and the polyA. b) By WGS, only one discordant read aligning to 22:29065658 was observed, but no split reads encompassing the insertion breakpoint or a polyA/T were present (= not enough evidence to call an insertion).

Annotations used in FASTA sequences below:

Chromosome 22 (3´ transduction)

Chromosome 7 (Target)

Restriction enzyme cut site

Point of inversion

Microhomology

Primer binding site

a) Consensus sequence of LDI-PCR product from *Nsi*I digested template, amplified by primer pair 1 on c985T tumor sample. Target location identified as chr4:93280475 (*GRID2*)

```
gtgtatacttcgttcagttacgtattgctaaggttaaaggattcattccacggtaacaccagcacctcccaaaa
tatacccaattactggcaaaaaatgttattattaaactgatgaaaaaaaataaaaaaaaaaaaaaaataaaaaaa
aaaaaaaaagtaatagcatgaaactagttttggagtcatacagtcctaagagttaaattctagctccatactgaa
atgattgcatgaatgttggcaagttacttatctgacctataacttttcatctgttaatcagataattagatataa
tttatagggctgagtttaaggatgtaattcacaggaaaaagcccagcacaatgcacaggaaaagccagcacaatg
cctggcagtttgcatttactaaactatgtgttatgcttactctttctcccatcttacagaggtttgtgcatttca
atagagcatagcccctcaacactagggaatgcatactttttttttgcattatgtacttgcatgtatcaactaatt
gcaatttcatgtttttttttgaacaatagtaacatacgtctaaatacacagaaagccttacataggcaacatttt
tctgtatggaaaacaatgaatagagtcccttaaaatgtgtgaacaaatattgtagttaaatttttttagtagagg
aaagctgatatcctgtttggttggccttaactaactaccaaatttttaagtagtgaagttttgttctttgttct
tcttttcacgattttatggtccctcttccttcagtactcaccaacatgtagtgttctcatgtgacttctattctt
tctcttaccaagcatgcttccttcatctaatgatcagttggaaagcttaggaccactgtgatcaaaagattgaaa
aaataactttataaagtaatagcatgttaagttacttataatccaatttaaatgtaagttcaaataccttaacat
gttcacattacttttaattccacaaaaacgttcttatctatctttattttaagatgttttccacatacatgtagt
gaaaagtgctgcttgtccatcgggttttgtgtaacctttaacctgtatgcgtgactgt
```

(i) After Human BLAT Search (https://genome.ucsc.edu/cgi-bin/hgBlat)

chr22:29065832-29065893 **polyA** chr4:93280454-93280795atgcatchr4:93280043-93280482chr22:29065650-29065782

(ii) Reconstructing the LDI-PCR product for depiction of the insert before *Nsi*I digestion and T4 ligation

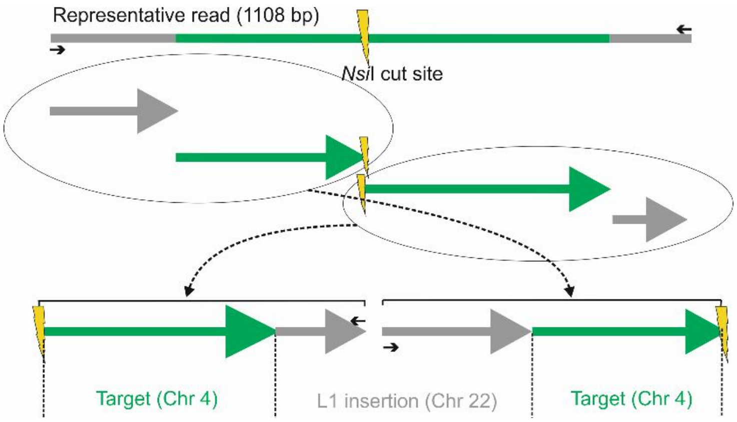

atgcatchr4:93280043-93280482chr22:29065650-29065782 **(Primer gap)**
chr22:29065832-29065893 **polyA** chr4:93280469-93280795atgcat

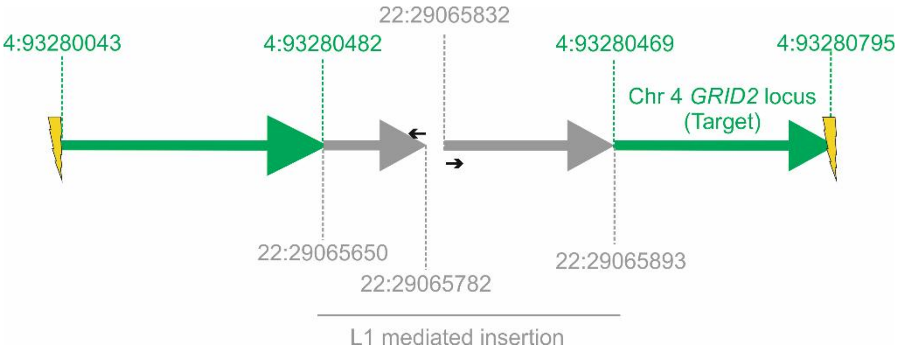

Target site duplication= 13 bp

Forward strand insertion

twin priming absent, microhomology present

Insertion= 29065650-29065893

Insertion length= 243

b) Consensus sequence of LDI-PCR product from *Sac*I digested template, amplified by primer pair 1 on c985T tumor sample. Target location identified as chr7: 146783223 (*CNTNAP2*)

ggaagttacgtattgctaaggttaaaagttgattcattccacacggtaacaccagcacctttcactaagcatgta
tgtggaaaacatcttaaaataaagatagataagaattttggtaacatggaaatttctgataggggaagaatattc
ccagaagtgtgaccagttagggccaaaataaaaggtacaatttacccaattattatacctaagtaccggccttat
taaatatataaacctcaacatcaaattgtactaaaaaatcaggaaactaagctcctagtagagaactggtcaaag
ggaagatctttgtggagtcagcagtttctctgtcctacaatttggcaaaataatctcataagcacaagaaggtt
aatatgtttgcattaaaaaatgttaagttacttataatccaatttaaatgtaagttcaaataatctttaaacatg
ttcacattactttttttttaatgatgatattttaaaggagcattttaatgtgaacgtagtgaaaatacaaaatc
atatttaaataggaaagaaagggatatctctgggctcaatgagttttgaaagaggagaggtttgaagaataaatg
agtttacagaaaataaacaaaaatatgaaaacaaagaagaaaatagatatagagaagcaaaagtataaaaatagc
aacatttaaaaattttatctatgctcttttgatacatccacagaaaatatgtttctcttttctttttcttttca
taaaatttatttttatttaatttttttaaattacttttgagacagacagatttctctatcacccagaatggag
tgcaatggcatgatcatggctcatggcagcccagtttcctggctcagtcaataatcccacctcagcctcctaagt
agctgaaacaggcatgcaccaccatatgtagctaatatttttttaaattatttctgtggagacagggtctctatgt
tgcccaggctggctcaaactcctgggctccagcgattctccaccccagcctcccaaatcgctggaattataagca
tgaccacctagtctggccatacagaagatacataatcttagacccccttcctcatgtaacatgtagcactttctct
aactatatcaaataatgtctgggaaacagcaattttaaatctttagtatggtataattattgtcaaagcaaaaca
agatgaaacttcagaaaagtctagtacagagcttcaacgaattcaagttaagttttacacctgttacccctgata
ctttagaaaagttttatgcttgccctctattgtgcttagagagaatgttgtggtttgtaggaaaatagacactgg
taagtgtaactgtatctgaattttgcacacttcaccagtgtgtctggttattatctacatggtggaccttcagtg
actgaggacctctcttccattcaagctgtcttttattaaccgttctctggggcctcaagttcctctgcagcattt
attttgagttaagtattgttcccattattgtcttagaaacgttaagtatcattagatacagtatctcattgtgga
ttatgttatcagattctttgcaatgtgtctctttaacaaaaggatattgagtggcagagtggggaggaaaatcac
tgaaaaaaagaggacaccgaagagacacatgaattttctttgttctgtttttttaaccagtggtcatccctagc
aaagatgtgtgtaagtgcattaaaaacatttgtgtgtagcgaaagtggtgaatttctctatgcaacatatttaaa

aagtcattgtttgtacctcaatttgcatggctatttggccctataaatattggctcttgtctaatgacattttct
atttcacttttaatagaatttgctgtaaaatggatactacagaaacagcattttattttcattctttggtaaaat
catttccacccaaagtgggggagctctgcctcctgtcagatgagtgcccgcattagattctcataggagcacta
accctactgtgaactgcacatgcaagggatctaggatgaacactcatgagaatctagtgcctgatgatctgaagt
gaacaacttcatcccaaagcccacccccaagcccaacctgtccgtgggaaaaaaattgtctccaaaacactgggc
cctggtgccagaaaggttgagatcgctgccataggtgatatctctcctagaaccatacacctctggttttaagag
ctaaaaaacatacagacttattctgtaagtgtaaacattttactgtgtgttacctggccaattcttcacagctga
tcattgtatattttaaattctcttcatgtctcatcgttttattagcgtgtttcttcccattatgccattctgaaa
tcctttttacaagttgtcctctggtccagtgatctggtgatacaaaatgattgatagcaataaacatatcatttgc
atctgcaacccaaatgtcctgtgtgttcctgtagaaattatgtagctaagacacgcacacgaaagctctgtaagt
caaagagtagcgtcgtgtttttccaaataaatttttttctttaaaccttgagcctcattgaagcacaacttgaaat
ggtagaaaacccagtggggcatgtcaccaaaagcagtttcctaccgcagcttccaagagaacttcattagtgttt
tcaacctgaatactaatcaatggcataaactctaaggaaattttaatatgaatatttttttttttttttttttt
tttaatccagtaataaacctttatttttctcatcagtttaataataacattttttgccagtaattgggtatattt
tgggaggtgctggtgttactgtggaatgaatcctttaacctagcaatacgtaactgaacgaagtacaatgt

(i) After Human BLAT Search (https://genome.ucsc.edu/cgi-bin/hgBlat)

chr22:29065782-29065725(-)chr22:29065369-29065721chr7:146783223-146784742 gagctc chr7:146782455-146783241 **polyT** chr22:29065912-29065832(-)

(ii) Reconstructing the LDI-PCR product for depiction of the insert before *Sac*I digestion and T4 ligation

gagctc chr7:146782455-146783241 **polyT** chr22:29065912-29065832(-) (Primer gap) chr22:29065782-29065725(-)chr22:29065369-29065721chr7:146783223-146784742 gagctc

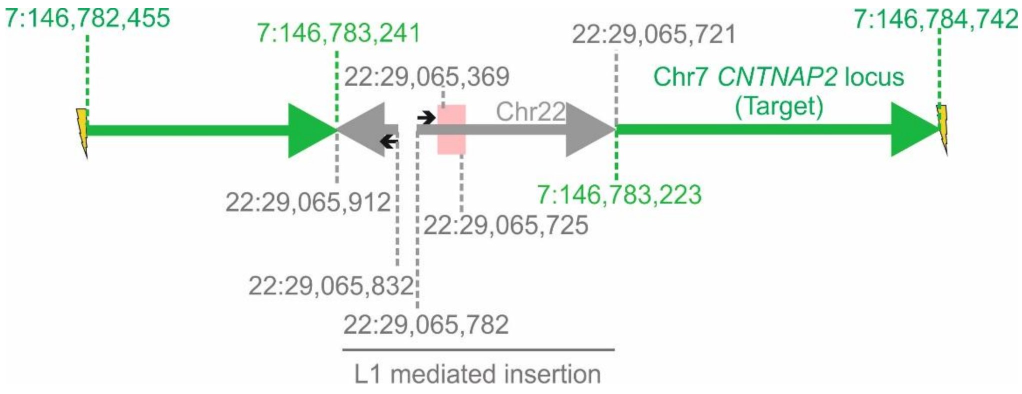

Target site duplication= 17 bp

Reverse strand insertion

twin priming present, microhomology present

deletion due to twin priming = 6 bp

Insertion= 29065369-29065912

Insertion length= 536

Supplementary figure S3: *TTC28* LINE-1 mediated 3´ transduction at (a) *GRID2* locus and (b) *CNTNAP2* locus in chromosome 4 and 7 respectively detected by LDI-PCR/Nanopore sequencing. For both cases consensus LDI-PCR product read generated by Nanopore sequencing with complete sequence information was used. (i) shows the genomic co-ordinates of the read and all junctions after its alignment with human reference genome hg19 using BLAT (ii) genomic co-ordinates from (i) were reconstructed to remove alteration produced by digestion, self-ligation and inverse PCR is shown along with schematic representation of the insertion.

Annotations used in the FASTA sequence below:
Chromosome 22
Restriction enzyme cut site
Primer binding site

Consensus sequence of LDI-PCR product from *Nsi*I digested template, amplified by primer pair 1 on c985T tumor sample that shows full length *TTC28* L1 at its native location

TTTTCGTTAAAGGATTCATTCCCAGGTAACACCAGCACCTGCCAGATCAAGTAATCCTACTCATTTTACCTGTATTACTGAAA
ATAAAAATGGGGGGGGCAAATAGGAACTTTAATATCTTTAATGATGTTTTAAGGTTGAACTTTATTTCTGACATTTTCTACAC
CTCTAATGAAATACATTTCACATATCGTAAACTTACCCATTAAAGAATCAGTTCAGTGTCTTTTGGTGATTTATTCAGAGTTT
GCAACCATTACCACAATCTAATTTTAGAATAGTCCTCATCACCCATACCCATCAACAGTCATTCCCTCCTCCTCCCCACCCAA
GCTTGTTCAGTGACAAACTAATCTTCTTTCCCTCTCTATGCATTTTTTAACAGCAAAAAAATATTTTCGGAAGTATATCTTTA
TTTCACAAACCTGGTCACAAAACTAAAATTCTGAATCAAAACACAACTTATAGTACCCAACACATTTCTCTTTAACTTTTATT
TGTACTATGAATTGATTCTTCCTCATCAATAACCAAGGAATATACAAAGTAGTGATTTTCTTTATCATTTCTTTCATCAGCAC
AGTAAAAGGAAATTTTTTTCTTTTTTAGAGACGGAGTCGCTCTGTTACTTGAACTGAATGCAGTGGCACCATCTCAATCACTGC
AACCTCTGCCCTCCCAGGTTCAAGTGATTCTTCTGCCTCAGCCTCCCAAGTAGCTGGGACTGCAGCAATTTTACACCACCACA
CCGGCTAATTTTTTTGTTTTTTTGTTTTGTTTTGGTTGGTTTGGTTTGGTTTTGGTTTTAGGATGGAGTCTCACTCTGTCCTC
AGACTGGAGTACAGTGGCGCTATTTCGGCTCACTGCAGCCTCCACCTCCTGGTTTCAAGGGATTCTTCTGTCTCAGCCTCCCA
GGTAGCTGATACTGGGCATGTGCCACCACACCCGGCTAATTTTTGTGTTTTCAGTAGAGACGGGGTTTCTATTATTAGCCAGG
CTGGTCTCAGTGCTCCTGACCTCAGGTGATCCACCACGTTGGCTTCCCAAAGTGCTGGGATTACAGGCATGAGCCACCGCGGC
TGGCCAATTTTGTATTTTTTAGTAGAGATGGGTTTCACCATATTGGCCAGAATGGTCTCGAACTCCTGACCTCATGATCTGAG
AAAGGAAAAAAATTTTAAATATAATTCCCTTTATTTCAAAATTGCACTTGAGGAAATTAGAATCATTCTTTTGCTATCACCAA
AAACTACTGCTTTTAAGGCCAGCCGCAGTGGCTTACACCTGTAATCCCAGCACTTTGGGAGGCCAGGGTGAGCGGATCACCTG
AGGTCAGGAGTTCGAGACCAGCCTGACCAACATGGAAACCCCGTCTCTACTAAAAATACAAAATTAGCCGTGTGTGGTGGCTG
CCTGTAATCCCAACTACCCAGAGCTAAGGCAGGAGAATCCCTTGAACGAACCCAGGAGGTGGAGGTTGCGGTAGTCGAGATCA
TTTCGTGTACTCCAGCCTGGGCAACAGAGTACAGCTCCATCTCAGAAAAAAAAAAAACAATTGTTGTTATATAGCCTCATTCCT
TATAATCCCTTCTTCATTATTTAGTTTCATCATCCAAGAAACATTTATATCCAGGTACTACATTAAGTATTGGTTAATAACAG
CTTTATAACATTCATTTTATTCATTTATTTTTTGAGACTGGGTTTCATTCTGTGGCCCAGAGCTACAGTCTGTCTTATAGCTC
ACTGTAATTTGGAACTACTGGGTTCAAGGGTCTTCCTGCCTCAGCCTCCTGAGTAGCTGGGACTACAGGTACATGCCACCATA
CCCGGCCATTTGACTGATTGATAGACAGATGGTTTTGTTTTTTTTTTTTTGAGACCAGAGTCTCACTCTGTCACCAGGCTCAG
GTGCAGTGGCACGATCTCATCACTGCAACCTCTGCCTCCCAGGTTCAAGCAATTATCCTACCTCAGCTTCCCAAGTAGCTGGG
ATTACAAGGAGCATCACCACACCCAGCTAATTTTGTATTTTAGTGGAGATGGGGGGTCTCACCATGTTAGCAAAAGCTGGTCT
CGAACTCCTGACCTCAGGTGATCCACCTGCCTCAGCCTCCCAAAGTTCTGGGATTACAGGCATGAGCCACTGCACCTAGCTAA
AAAATATATTTTAGAGACTGGGTCTCACACTATGTCACCCAGAGTAGTCTTGAACTCCCTGACCTCAAGCAATCCTCCTACTT
CAGCTACCAAATAGCTGAAATACAGAAAGAACAACCATGCCTGGCTCAGTTTTGTAACTTTCAATACATAAAAATTACACTGC

TCACATTGTAATTAATTTCATCTTTCATTAAATTAATAGATAATAAAATTTGATAATAAACTTAATGTCAAATGCTGCTTCAG
TCAGTATTATAGAGATAAATTCAGAATGGTGTGCCAACATAACCTTAAACTCACAATAAAAACTGGGTTTTAACAATATCCAC
AGGAAATTATGTTATTTTCTGGCAACAAGTCTAACGTTGGTAGCAAAAAAAGGTAGTTATAATTGATCACTATTTGTAGTGAT
CCATACAAATCAAAGACAGTCTAAGGCAAATGGTTAGAACAGATTCAGAATGACTGCTAAAATTAGAATCAAGGCAATTCAGT
TTCCCTAGCTGGATGGTATTGATAAAGTTACTTAACCTCTCATACTACACTTGTCTCCATATGTAAGATGGGGATAATGACCT
ACTTCTTAAGTTGTGAAATTAAGAATTAGTTAATATATGTAAAGAGTATTTAAACGAAGTACTGACACAGAGTACACACAATA
TAAATGTAGTCTAGCTATAATCTAAACACCTATATACTTACACAGAAGGGAAAGGTGCTTTCCACGATACTCTATAATTTATA
TGGTTCTTCCAAAATAAGCCTAATGAAAATTTTATTTTTCATAAAATATATTTGGAATTTGTAGCCTAGCCAGACAATTATTA
TTAAATGGTTTTCATAAATCTTTGTGAATATTAAGGAAGCAATGGATTCAACAAAAGCATTTAATGAGCGGTGGGCAATATTC
AAAACTCCTGTTGCTACACAAGCCAAAAAAACAGACAGGGATCAAGACAACTCAAAACAAAATATTACACACAGAACAGGAAA
CAACTGTACAGTATCAGGAAATGCAATACACTTTTTCTAGTGATAAGAAACTTCCACCGGGCGCAGAGTGGCTCACACCTGTA
ATCTGGGAGGCTGAGGCAGGCTGATCTCGAGGTCAGGAGGTCAAGGACCATCCTGGCTAACACAGTGAAACCCATCTGTACTA
AAATACAATAAATTAGCCAGGCGTGGTGGCGAACGCCTGTCCCAGCTACTCGGAGTGAGATGGGAGAATGGCGTGAACCAGGA
GGCAGAGATTGCAGTGAGCCGAGATAGTGCCGCTGCACTCCAGCCTGGGAGACAGAGCGAACTCCGTCTCAAAAAAAAAAAAA
AAGAAAGAAGAAGAAAAAGAGAGGGAGGAGCCAGATGGCCGAATAGGAACAGCTCGGTCTACAGCTCTAGCAGTAGGCAGGCA
CAAGAGACTTGGGTGATTTCTGCATTTCCATCTGAATGAGGTGCCGGGTTCATCTCACTAGGGAGTGCCAGACGGTGGGCGCA
GGCCAGTGTGTGTGCACCGTGCGCAGGCAAAGCAGGGCGAGGCATTGCCTCCTGGGAAGCGCAAAGGGGTCCAGAGTTCCTTT
CCGGGAGTCAAAAGAAAGGGGTGACGGACGCACCTGGGAAAATCAGGTCCTCCACGCGAATGTGCGCTTTTCAGACCGGCAGA
AACGGCACCACGAGACATATCCCACACCTGGCTCAGAGGGTCCTACGCCCGGAATCTCGCTGATTGCTAGCACAGCAGTCTGA
GATCAAACTGCAAGGCGGCAGCCAGGCTGGGAGGGGCGCCCCGCCGTGCAGGCTTGCTTAGATGGCTCAAAGCAGCCGGGAAG
CTCCAACTGGAGTGGAGCCCACCACAGCTCAAGGAGGCCTGCCTGCCTGTAGGCTCTCCACCTCACGGAGACAGGGCACAGAC
AAACAAAAGACAGCAGTAACCTCTGCAGACTTAAGTGTCCCTGTCACAGACAGCTTTGAAGAGAGCAGTGGTTCTCCCAGCAC
GCAGCTGGAATCTGAGAACAGGCAGACTGCCTCCTCAAGTGGGTCCCTGACCCCCTGACCCCCCGAGCAGCCTAACTGGGAGG
CACCCCCCAGCACAGGGGCACACTGACACCTCACACGGCCAGGGGTATTCAACAGACCTGCAGCTGAGGGTCCTGTCATTAGA
AGAAAACTAACAACCAAGAAAGGACATCTACACGAAAACCCATCTGTACATCACCATCATCAAGACCAAGTAGATAAAACCAC
AAAGATGGAAAAAAACAGAACAGAAAACTGGAAACTCTAAAACGCAGAGCCTCTCCTCCTCCAAGGAACGCAGTTCCTCACCA
GCAACAGAACAAAGCTGGGTGGAGAATGATTTTGACAGGCTGAGAGAAGGCTGAACGATCGATTATACTCCTGACTGCAGGGA
GGACATTCAAACCAAAGACAAAGAAATTGAAAACTTTGAAAAAATTTAGAAGAATGTATAACTGGAATAACCAGCTGAAAGTG
CTTAAAGGGGCTGAGTGGAGCTGAAAACCAAGGCTCGAGAACTACGTGAAGAATGAAGCCTCAGGACCGATGCGATCAACTGG
AAAAGATATCAACAATGGAAGATGGAATGAATGAAATGAAGCGAAGAAGGGAAGTTTACAGAGAGAAAAAGGAATAAAAAAGA
AATGAGCAAAGCCTCTCCAAGAAATATGGGACTATGTGAAAAGACCAAATCTGCATCTGATTGGTGCCTGAAAGTGATGTGGA
GAATGGAACCAAGTTGAAAACATACAGGATATTATCAAGGAGGAGAACTGTCTAGCAAAGGCGAATAACGTTCAGATTCAGGA
GGAAATTACAGAGAACACTACAAAGATACTCCTCGAGAAGAGCAACTCAAGACACATAATTGTCAAGATTCACCAAAGTTGGA
AATGAAGGAAAAAAAATGTTAAGGAGCAGCCAGAGAAAGGTCAGGTTACCCTCAAAGGAAAGCCATCAGACTAACAGCGGATC
TCTCAGCAGAAACCTACAAGCCAGAAGAGAGTGGGGCCAATATTCAGCATTCTTAAGAAAAGAATTTTCAACCCAGAATTTCA
TATCCAGCAAACTAAACTACATAAGTGTGAAGGAGAAATAAAATACTTTATAGACAAGCAAATGCTGAGAGATTTTGTCACCA
CCAGGCCTGCCCTAAAAAAAAAACTCCTGAAGAAGCTAAACATGGAAAAGTGGCAGTACCAACTAGCTGCAAAATCTGTATGC
CAAAATGCAAGACCATCGAGACTAGGAAGAAACTGCATCAACTAATGAGCAAATCACCAGCTAACATCTATTGTGGATCAAAT
TCACACATAACAATATTAACTTTAAGCTAAATGGACTAAATTCTGCAATTAAAAGACACAGACTGGCAAGTTGGATGAAGTCA
AGACCCATCAGTGTCCTTGTATTCAGAAACCCATCACGTGCAGAGACACACATAGGCTCAAATAAAGGATGGAGGAAGATCTG
CAAGCCAATGGAAAACAAAAAAAGGCAGGAAGTTGCAATCCTAGTCTCTGATAAAACAGACTTTAAACCAGCAAAAAGATCAA
AAGAGAGACAAAGAAGGCCATTACATAATGGTAAAAGGGATCAATTCAACAAGAGAATAACTATCCTAAATATTTATGCACCC
AATACAGGAGCACCCAATTCATAAGCAAGTCCTCTCAGTGACCTACAAAGAGACTTAGACTCCCACATTATGTGGGAGACTTT
AACACTACCTGTCAACGTGGGCAGATCCAGCAGACAGAAAGTCAATGAGATGCCCAGAATTGAACTCAGCTCTGCCAAGCAGA
CCTAATAGTATCTAGAACTCTCCACCCCAAATCCAGCAAAATATACATTTTTCAGCACCACGCCACACCTATTCCAAAAATTG
ACCACATGGTTGGAAGTAAAAGCTCTCCTCAGCAAATGTAAAAAGAACAGAAATTATAAACTATCTCTCAGACCTGAGTGCAA
TCAAGCTAGACTCAGGATTAAGTCTCACTCAAAGCCGCTCAGCTTCATGGAAACTGAACAGCCTGCTCTGAATGACTGCTGGG
TACATAGCAGAAATGAAGGCAGAAATAAAGGTGTTCTTTGAAACCAGCGAGAACAAGACACCACATGCAGAATCTCTGGGACG
CATTCCAGAAGCAGTGTGTAGGGGAAATTTGCTAGCACTAAGTACTTGCACCACAAGAGAAAGCTGAGGAAAGTCAAAGTGAC
CCTAATCTGAATTAAAAGAACTAGAAAAGCAAAGGCAAACACATTCAAAAGCTAGCAGAGGCAAGAAATAGCTAAAATCAGAG

CCAAATTGAAACTAGAATTTGCAAAAAAACCCTTCAAAAATCAATGAATCCAGGAGCTGGTTTGAAAGTCAACAAAATTGATG
GGCCATGTAGAGGACTAATAAAAGAAAAAGAGAAGAATCAAATAGACACATTAAAATGATAAAGGGGGAGGTTATCACCACCA
GTCCACAGAAGCCAAACTGCATCAGAGAATACTACCAAACACTCTACACCAAATAAACTAGAAAATCTAGAAGAAATGGAATA
CATTCCTCAACATACATCTCTCCCAAGACTAAACCAGGGAAGAGGGCAGATCTCTGAATATAGACCAATAACAGGCTCTGAAA
TTGTGGCAATGATGTGGTTACCAACCAAAAGAATTCAGGACAGATGGATTACACTGCAAGGTCTACCAGAGAGGTACAAGGAG
GAACTGGTACCATTCATTACAAAACTATTCAATCAATAGAAAAGAGAATCCTCCTAACTCATTTTTTTATGAGGCCAAGCATC
ATTCTGATACCAAAGCCGGGTAGAGACACAACCAAAAGAGAATTTGGAGCCAATATCCTTGATGAACATTGACGCAAAATCAT
AAGCCAAATACTGGCAAACCAGATCCAGCCTTTTTTTTATCAAAAAAGCATCCCACCATGATCAGTAGACTATTCTGGGATGC
AAGGCTGAGTTCAACTCACAAATCAATAAATGTAATCCAGCATATAAACAGAGCCAAAGACAAAAAACCACTGATTATCTCAA
TAGATGCAGAAAAAACCTTTGACAAAAGATTCAAAGCAACCCTTTAATATGAAGCTTCTCAATAGAATTGGGTATTGATGGGA
CGTATTTCAAATAACAGAGCTATCTATGATGACAAACTACAGCCAATATCATACTGAAATGGTAAAACTGGAAGCATTCCTTT
GAAAACTGGCACAGACAGGGATGCCTATCTCACCGCTCTATTCAACATAGTGTGGAAGTTCTGGCCAGGGCAATCAAGGCAGG
AAGGAAATAAAAGGGTATTCAATTGGAAAAGAGGAGAGGTCAAAATTGTCCTGTTTTAGACATGATTGTTTTATCTAGAAAAG
ACCCCATCGTCTCATTGAAATATAGCCAAGCTGATAAGCAACTTCAGCAAAGTCTCAGGATACAAAATCAATGTACAAAAATC
ACAAAGCATTCTTATACACCAACAACAGACAAAGACAGAGAGCCAAATCATGGGTGAACTCCATTTCACAGGTAGCTGAAGAC
CAAATACCTGGGAATCCAACACAAGGAATTCTTGGGACTCTTCAAAGGGAGAACTACAAAACCACTGCTCAAGGAAATAAAGA
GAGGACACAAACAAATGGAAAGAACATTTCCATGCTCATGGGTAGGAAGAATCCAATATCGTGAAAATGGCCATACTGCCGAG
GTAATTTACAGATTCATGCCATCCATCTATCAAGCTACCAATGACTTTCTTCTGGGGTGGAAAAACTACTTTAGAAAGTTTCA
TATGGAACCAAAAAGGCCCGCATTGCCAAGGTGTCCCTAGAAAACCAAAGAACAAAGCTGGAAGCATCCACACTGCCTGACTT
CCAAACTATACTACAAGGCTACAATGCCAAAACAGCATGGTACTGGCACCCAAAACAGAGATATAGATCAATGGAACTAGA
CAGGCCTCCAGAAATAATGCCCATATCTACAGCCTCTGATCTTTGATATGGAAGCCTAGAAGAAACAAGCAATGGGGGAAAGG
ATTCCCTATTTAATAAATGGTGCTGGGAAAACTGGCTAGCCATGTAGAAAGCTGAAGCGGATCCAGCAACACCTTATGCAAAA
ATCAAATTCAAGATGGATTAAAGATTTAAACGATTAAACCTAAAACCATAAAAACCCTAGAAGAAAACTAGAGCATTACCATT
CAGGACATAGGCATGGGCAAGGACTTCCATGTCCAAAACACCAAAAGCAATGGCAACAAAACCAAGAATTGACAAATGGGACT
AATTCAAACCTAAAAGGCTTCTGCACAGCAAAAGAAAAGAAACTACCATCAGAGTGAACAGGCAACCTACAACATGGGAGAAA
ATTTACCATGACCTACTCTCATCTGACAAAGGGCTAATGTCCCAGAATCTACAATGAGCTCAAAACAAATTTACAAGAAAAAC
AAAACAATAATAAAAGTGGGCGAGGACATGATGTAAGACACTTCTCAAAAGAAGACACATGCAGCCAAAAAAACATGAAGAAA
TGCTCATCATCACTATTGTTACATCAGAGAAATGCAAATCAAAACCGCATGAGATGTCATCACACCAGTTAGTGGCAATCATT
AAAGTCAGGAAACCAGCAGGTGCTGGAAGGGATGCGGAGAAATAGGAACACTTTTTTACACTGTTGGTGGGACTGTAAACTAG
TTCAAACCATTGTGGAAGTCAGTGGTGATTCCTCAGGGATCTAGAACTAGAAATACCAACAGGCCCAGCCATCCCATTACTGG
GTATATACCCAAATGAGTATAAATCAATGTAACTATAAAGACACACATGCACACGTATGTTTATTTGCGGCACTATTTCTATG
TAAGACTTAAGAATAGCCAGAAATGTCAACAATGATGACGGATTAGAAAATGTGGCTGCCACACCATTAAGAATACTATACAG
CCATAAAATGATGAGTTCCATATCTTTGTAGGGACATTGGATAGAAATTGGAAACCATCAAATTCTCAGAAACTATCACAAGA
ACAAAAAAATAACACCGCATATTCTCACTCATAGGTGGAATTGGAGGGGCAATGAATCACATGGACTGGGAAGGGGAACCTCA
CACTCAGGGACTGTGGTGGGGAGTAGGAGGAGGGGGGAGGACAGTGGAGATATACCTAATGCTAGATGACACGTGATTACAGC
ACCAGCATAGCATGTAGCCACATATGTAACCTGCACAATGTGCACATGTACCCTAAAACTTAGGTATAATAAAAAAAAAAAAA
GAAAGAAAAAAAAAGCTTCACATGGGGCCACATGTCTAATAAAGAGTGACTGAATCCCAAAACCTCTTAGTGGCGCAAAATTC
CTGATAGGGGAAGAATATTCCCAGAAGTGCTTGACCAGTTAGACCCAAAATAAAAGTACAAATTTACCCAATTAGTATACCCT
AGTACTGACCTTATTAAATATATGTTCCTCAATACAAATTGTACTAAAATCCAGGAAACTAAGCTCCTAGTAGAGAACTGGGT
CAAGGAAGATCTTTGTAGGTCCGGCAGTTTTTCCTCTGTCTGAATTTGGCAAAAATAATCTCTGTAAAGCACAAGAAGGTTAA
TAATAGCACATTAAAATGTTAAGTTACTTGTCAATTTAAATATGAGTTCAAATATAGCATGTTCTGATACTTTTTATTCAAAA
ACGTTCTTATCTATCTTTGTACACAGATGTTTTCCACATACATACTTAGTAGATTTTCTTTTGGAGATATGAAGGTCTACAAA
TCTTTATTCTACTATTTCCCCAAAAATATACCCAAATTACTGGCAAAAAAAATATTTATTATTAAACTGATGAAAATATAAAG
GTTTATTACTGGGATAAATAAGTTTTCCATATGCTTGAATTTTTTTATACACACAGTATATACTCTATTATATGTATACACAG
AATACAACTCTATCATTGAACTTGGTTTGTCATTCCAT

chr22:29066035-29066380 atgcat chr22:29056087-29065692 (4bp gap) chr22:29065696-29066018

atgcat chr22:29056087-29065692 **(4bp gap)** chr22:29065696-29066018 **(Primer gap)**

`chr22:29066035-29066380 atgcat`

Supplementary figure S4: Consensus sequence analysis of the native LDI-PCR product containing full length *TTC28* L1.

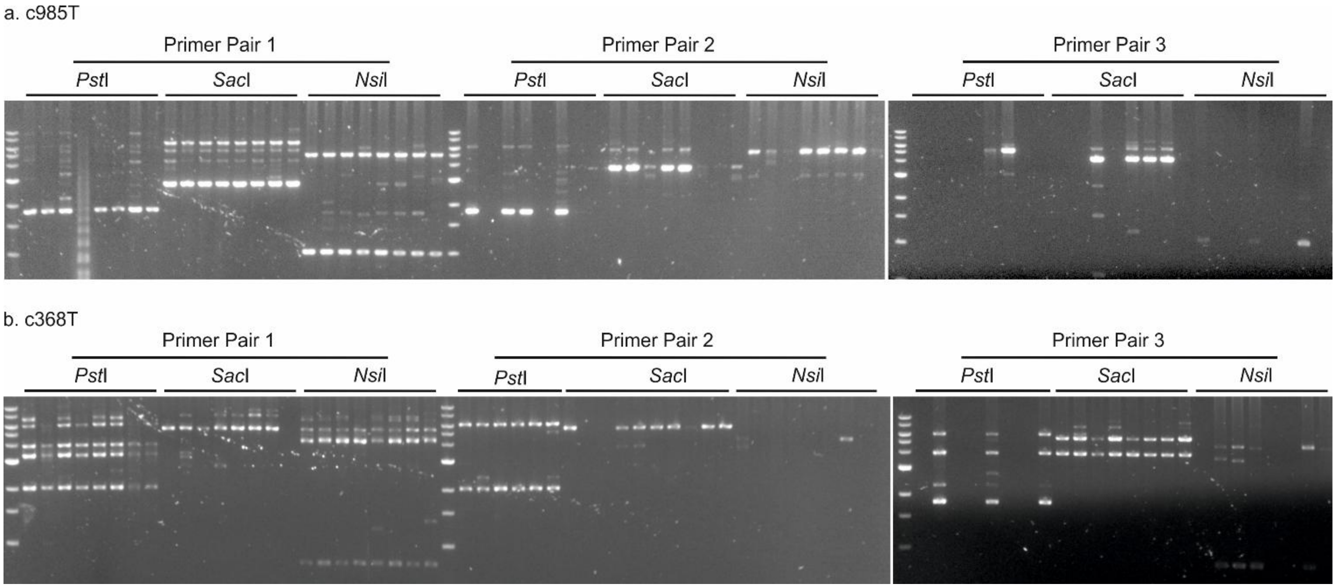

Supplementary figure S5. LDI-PCR on two colorectal tumor DNA (c985T and c368T) digested with three different enzymes (*Pst*I, *Sac*I and *Nsi*I) using three different inverse primer pairs (8 replicates for each case) gave - a native product of known size and tumor specific products of different sizes representing various putative 3´ transduction targets. All the replicates of each reaction that showed uniform PCR product band pattern in the agarose gel were pooled and processed for Nanopore sequencing. The replicates that did not show uniform PCR product were repeated (‘M’ denotes 1 kb ladder (NEB) and reaction without any template was run on ‘-’ lane as control)

References:

1 Tabaska, J. E. & Zhang, M. Q. Detection of polyadenylation signals in human DNA sequences. *Gene* **231**, 77-86 (1999).

2 Katainen, R. *et al.* BasePlayer: Versatile Analysis Software For Large-Scale Genomic Variant Discovery. *bioRxiv*, doi:10.1101/126482 (2017).
