# Supplementary material for: Detection of subclonal L1 transductions in colorectal cancer by long-distance inverse-PCR and Nanopore sequencing
Source: Sci Rep. 2017 Nov 6;7:14521. doi: 10.1038/s41598-017-15076-3 (PMC5673974; doi:10.1038/s41598-017-15076-3)
Supplement: Supplementary file 2 — Supplementary dataset [file 41598_2017_15076_MOESM2_ESM.doc]

Annotations used:

L1 insertion (+ strand)

L1 insertion (- strand)

Target (always + strand, when not Reverse complement (RC) is used)

Restriction enzyme used

Microhomology

Primer gap means sequence between the primer pair that remains unsequenced

PolyT is observed in –strand insertion and PolyA in + strand insertion

In cases of twin priming some sequence can be deleted, this has been taken into account when calculating the insertion length. The inserted sequence co-ordinates takes into consideration 5´most and 3´most co-ordinates of TTC28 region, regardless of junctions (because due to twin priming the 5´most co-ordinate can be in the middle instead of junction)

Deduction of different variables in Table 1 was noted for all consensus sequence like shown in the first consensus sequence representing insertion at chr1:195769724

1) For 3´ transductions in c985T sample

Consensus sequence for insertion at chr1:195769724 (Twin Priming)

gaagtatgcttcgttacgttgtattgctaaggttaaaggttcattccacggtaacaccagcacctgccagatcaagtaatcctactcattttaccctgtattactgaaaaaaaaatggggggggggcaaataaagactttaatatctttaaaaaaaaaaaaaaaaaaaaaaaaaaaaaaaaaaaaaaaaaaaaaatatatacctgccatacaacccaacaattccactcctaggtatctaccatagagatgaaaactcatgtttccgtaaagatttgtacactgatgtttatatttcctattggtaatagacaaaattacaaacaaacatccataaacaggtaaatcaataaacaaacagtggaacatgcattcacaaggagatgatttgaaatcagaaacttatgtttaaaaggaaagcagagcataaagtttggaaatttgcagcctgatcacggggtaggaaagaaaagacccattttctggggagaaattcaaacctgctgcagaaatctgaaatatactgcagaaaccctgataaacccatcagatttcgtgaggcttattcactaccatgagaacagtatgtgagaaatcgtccgtatgattcaaattatcttacaccaggtccctcccgacatgtgggaattatgggagtacaattcaagatgagatttgggtggggacacaaagccaaaccatatcatatatgtatatatatatatcactgtatataatttaaacagctaatataaagaaatggaaatgcaaaagaggaagagaagaaaaaaataatggtaaacaaaaacataaaatgattagaactaagaataagtccaaacatgtagtagtcttgatttgtatgactagattaagtgtcttattataaaacaaatgctcacgtttgatactaaaattatataactttatggtatttgcaagatacatctaaaacagaacacatagaaataaatttgctggaaataaagagaataaaaaaatatatacctcatcagtttaataataacattttttgccagtaattgggtatattttttgggaaatagtagaaaatataaaggtttattactggattaaataagtttttccatatgcttgaattttttatacacacagtatatactctattatatgtatgcacagaatacaactctatcagtattgaacttagtttgtcattccataaaatggccatagaaggtgctggtgttaccgtgggaatgaatccttttaaccttagcaatacgtaacttt

chr22:29066035-29066121 polyA chr1:195769709-195769897 atgcat chr1:195769075-195769724 chr22:29065883-29065826(-)chr22:29065882-29066032

atgcat chr1:195769075-195769724 chr22:29065883-29065826(-)chr22:29065882-29066032 (Primer gap) chr22:29066035-29066121 polyA chr1:195769709-195769897 atgcat

Target site duplication= 16 bp

Forward strand insertion

Twin priming present, microhomology present

Deletion due to twin priming = 0bp

Insertion: 29065826-29066121

Insertion length=295

Consensus sequence for insertion at chr4:93280482

Gtgtatacttcgttcagttacgtattgctaaggttaaaaggattcattccacggtaacaccagcacctcccaaaatatacccaattactggcaaaaaatgttattattaaactgatgaaaaaaaataaaaaaaaaaaaaaaataaaaaaaaaaaaaaaagtaatagcatgaaactagttttggagtcatacagtcctaagagttaaattctagctccatactgaaatgattgcatgaatgttggcaagttacttatctgacctataacttttcatctgttaatcagataattagatataatttatagggctgagtttaaggatgtaattcacaggaaaaagcccagcacaatgcacaggaaaagccagcacaatgcctggcagtttgcatttactaaactatgtgttatgcttactctttctcccatcttacagaggtttgtgcatttcaatagagcatagcccctcaacactagggaatgcatactttttttttgcattatgtacttgcatgtatcaactaattgcaatttcatgtttttttttgaacaatagtaacatacgtctaaatacacagaaagccttacataggcaacatttttctgtatggaaaacaatgaatagagtcccttaaaatgtgtgaacaaatattgtagttaaatttttttagtagaggaaagctgatatcctgtttggttggccttaactaactaccaaatttttaagtagtgaagtttttgttctttgttcttcttttcacgattttatggtccctcttccttcagtactcaccaacatgtagtgttctcatgtgacttctattctttctcttaccaagcatgcttccttcatctaatgatcagttggaaagcttaggaccactgtgatcaaaagattgaaaaaataactttataaagtaatagcatgttaagttacttataatccaatttaaatgtaagttcaaataccttaacatgttcacattacttttaattccacaaaaacgttcttatctatctttattttaagatgttttccacatacatgtagtgaaaagtgctgcttgtccatcgggttttgtgtaacctttaacctgtatgcgtgactgt

chr22:29065832-29065893 polyA chr4:93280454-93280795atgcatchr4:93280043-93280482chr22:29065650-29065782

atgcatchr4:93280043-93280482chr22:29065650-29065782 (Primer gap) chr22:29065832-29065893 polyA chr4:93280454-93280795atgcat

Consensus sequence for insertion at chr4:155900401(Twin Priming)

ttgttgtgcttcgttcagttacgtattgctaaggttaagactactttctgcctttgcgagaacaacacctcagtcacctttattagacatgtggccccatgtgaagtttcttttttcttttcttttttttttattatactctaagttttagggtacatgtgcacattgtgcaggttagttacatatgtatacatgtgccatgctggtgcgctgcacccactaatgtgtcatctagcattgggtatatctcccaatgctatccctcccccctcccctaccccaccacagtccccagagtgtgatattccccttcctgtgtccatgtgatctcattgttcaattcccacctatggtgagaatatgcggtgtttggttttttgttcttgcgatagtttactgagaatgatggtttccaatttcatccatgtccctacaaggatatgaactcatcatttttatggctgcatagtattccatggtgtatatgtgccacattttcttaatccagtctatcattgttggacatttggaagagagtctacaaatctttattctactatttcccaaaatatacccaattactggcaaaaaatgttattattaaactgatgagaaaatataaaggtttattactggattaaaaaaaaaaaaaaaaaaaaaaaaaaaaaaaaaaaaaaaaaaaaaaaaaaaaaaaaaaaaaagtgagtggagaagagtgagtaggtcatgaaaccttcaacaatttggaacctgactgctaggctagttagtccaaattgagttcccagtgtgtatcctgacatgatgcttggtcttcacatagaatggattcttggctaaaatctgacattcttacagattaggattttctaaagatttaagattgttaaaaccaattcctgggagacagagttaggagcactttatgctctcatggcttcctgattttgtctccatattatatatggaattaaaatttccttttgcctgttctttccacttctgtatagactctatgaagaaaaagaactgagttttcctctattgtatctacagtagctagaaaaaatatattgataaatatatttggatatattaatattttataaacaatgcagatgaagaaattaaaattttatattgagcagtaatttttaaaggctaccaacaagcatttgctggctaaaactatttgagcatcaatgcctaagttggtggcttttagaaattttcctctgtaactcacaaaatatatactgtgatatagtttacacaatcatgcacaggcatataaaactaaaaaacacttttatgaaatcattcatgtacttactacttgtgtttcctctgatatttcctattctatttaattttattaaaggaaaatcatgagtggacctacaaaatcaatattgcccctcacaagtgtcatgaccagcagtcaaaatgctttcttggtgcatattgtccattgctatcttgaaaaaaaccccgatgtgctagggaaaaaaacatgcatcctacagtgagtttcccgcaggaagtatagccaaaatatttctaagtgagagaacagcttacctatcctttgctctcaagatcttgactctatggtatcttcagaatggaaatttttgttcagtttaaattatcaaacaattcaaattgtagagatgagaaagacctattttcatagtgaactgccacattttctcatggtttattatttgactcagaattctcaggcagcaatttaaaaaacagtgtttattttttaatctactttgatctctagtgatatcagcatagttatgataattatcaaaatggttactaaaatatatactgcaggagagtgtagtggacactgtggtgtgccacccacattcttcttcagggagaaggcctccctatccccagcccctagaagatttgttgttgacagctcacggctgtgtcactttcaggaaactgcccaagttaagacatcatcaaccaaagttacagttccttcctgggaatagcccacattcagtgcctgattgttaatgacagaggtgtatacaagccaagacactgtgtacacagtgggaaattcttactgtggaattttttttttaattatactttaagttctagggtacatgtgcacaacgtgcagctttgttacgtaatatgtatacatgtgccatgttggtgtgctgtacccattaactcatcatttacattaggtgtatctcctaatgctatccctcacccctccccagccccatgacaggccctggtgtctgtcccagctctagagcacctggtggattggttgagcccgtacagtgtaacctctttttctatctaaaccagtttctgtcctccctcacaggtgctggtcctgagactgctacctgattaccattttgcacataaatgtcagtctgcttcctggggctgacttgagacagcatctataatacaatacaactgtgttctgaactggagtctcgctctgtagcccaggctggagtgcagtgatgtgatctcagctcactgcaacctccacctcccgagtcctggttcaagcaattctcctgcctcagcctcctgagtagctgggattacaggtgcgtgccactatgcccagctaattttttgtattttttagtacagacagagtttcaccatgttggccaggctggtcttgaactcctgaccttgtgatctgcccgccttggcctcccaaagtgctgggattacagacatgagccaccatgcccggccatttcttttttgtcttcagaaaaaagtgagtggactctcttacatctctaaaaagaaaattcactaagcatgtatgtggaaaacatcttaaaataaagatagataagaacgttttgtgaattaaaaaagtaatgtgtgaacatgttaaggtatttgaacttacatttaaattggattataagtaacttaacattttttaatgcaagcatattaaccttcttgtgcttatgagattattttgccaaattgtaggacagagagctgctgactccacaaagatcttccctttgaccagttctctactaggagcttagtttcctgattttttagtacaatttgatattgagggtatatatttaataaggccgggtacttaggtataataattgggtaaattgtaccttttattttggccctaactggtcacacttctgggaaggtgctgttctcgcaaaggcagaaagtagtcttaaccttagcaatacgtaacttcc

chr22:29065354-29064889(-)chr22:29065801-29065912(+) polyA chr4:155900387-155901220 atgcat chr4:155899103-155900401chr22: 29065809-29065404(-)

atgcat chr4:155899103-155900401chr22: 29065809-29065404(-)(Primer gap) chr22:29065354-29064889(-)chr22:29065801-29065912(+) polyA chr4:155900387-155901220 atgcat

Consensus sequence for insertion at chr4:183051382

ttgttacttcgttcagttacgtattgctaaggttaaaggttacacaaaccctggacaagcagcacctcagtcacctttattagacatgtggccccatgtgaagtttctttttcttattaaaatactatacagcaaagtaatttgtataggtgaacgtgacacaaattccattagtggtattgtcaccaaaatttgatatctaaaagaaaactcatagcagatcaatactagtaagataaatggatcaaacttacacacacaaatcacatttcttttttataagctgattaatacttagcaatacataattaacagttacaaaaacattgtactatgtgtttaagtgacagtgcaataaattaaggcttcacatcaaaccccatgtaggcccacttttggagtgtctacttccctagtcagagacgttaccctcagtcagtgtgcacattttcattaccaagtaaaaagaagtagttgttgaatgaactggaatggctcaacataacatattaccacaagaagaaaagaaccatttataaatcctgatcctgtatgccgtttttttttcatctttggatctggacagttggtattagataaatgtttaagacaaagccgaacgattttagcatcatttcacacaaatcattacattatatttattttgaattttttctacgtaagctgaactagaagaaaataattggttagataaatgaagatattaataataaactctaacactctaaagtttaaattctatctcatttgattctcttaatatcttaagagaagcagatagagactattacttcttgtgtggcagatttaaccccaggatgacatgaaattgttatttgataacagttcagtttgttggtgataatggtgacccttggtgtttcaagtgggttttttggccaagtcatctgcacaagtgaatctaaactaattatttcaatagacctttcttatataaactgtgttaacgtaaacactgtagaaactctaaatgtagtggggaggttggggtgtgcagaacagacaaagagattgctttctacaatttaaccctgaacatatggcatgtccatacatgtaaaattaatgcccagtacaagcaatgatttcatagtttatcagtgattgcagatcacagcaaaatacgtttcgccacactactatggatctgttagaagataaacctaaatggataaaatgagctctgttctcttggagcagaacaaagtttactgaaagactcttttttcaaaaatggagttatcaagcaagcaatggttttgcaattttgtttttcaaatgtatagtctatatagcatgcaaactagcatattcagaaaagtatataaacttgagataaaaaaagtcataatattcttgttttcatttgtcaattttcttttgtaaagtatattttactgtgatgatcaaatttagacattaagaattaaatataaattcagcaggatgtgtataataaataaatcaaaaaaagttattaaatgaatatttgctttaggtgaaaaaaaaatttctttataggaagtttaataggtttgtttagtattatagtactactttataatatagttcattggtccacattaccaaataatattattaatgatgtttcacttttttttgttgttttggtcaaaggataaatattttatgtaaaatctgtggaaaaaaacgcagaaaccctattttatatggtaatccattttcaattttaatggatgttatcttatccttttttttttctatgtaggcatttttttgagtatcttcttgtagttatgctattagcatatgaactattaataaaatttgagttttgtacttgtttctttctttcttttcttattttttacttttttttttaagagacgggtcttgctatgttgcccaggttggtctttaactcctgcactcaagtgatcctccactacagcttcccaaagtgctgggattacaaacatgagctactgatgctgggattacaggcttcagccaccagcgcctggcctgtacttgtttcttttcaacaattttaactagtgcttctattgttccttttttcctattaagattcaagttttctttaagatgaacaattccttattaacttactgttcttttcttcgtgtcctaaaatgacacatcatcctgtggtataacactaagaacgaagttagtaaatttgagaattttgaaaatcctcatttgaaatacacaactggaattgaatattttctcctaaatttatcattaaataggtaataattgttacttttcatttcatcactggcactgtattaaaccctgaaacaactcaaaaaatcaacttcagaaattacctttgatttgacattacagaagttggtttgttattactatcagtttcacaacattgacaattaacttgagcaaaatgtaatagacttttttttctctcatattttcatactcaaattaattctgtcatcacaattattcacattcaaaaaaaaatttaaagcaggtcacgagggaagaaaatgccttaatttgaggccacgcagaagatctgtggcaacaaagcatgggaaaaggcgggaatagggttacatcctgaagcaacagacatctagcgctgtgcaaagtatatgactattaagtcgttttaaattcacataatatcatctgaaaaaaattgctaatgcacatagtcacaaacagcagtcagcagtagatagattgtgttaagtataaatgatttgaattactacctacatatatttagcttagaatatttataatacatacatggaaatctatatctcataatatataataaaagagaaggaaaatgtagaaacctggtattttttagagtatcacaaatctgtgttgtcaaatatgaatcaatttactcttcaaattcctgcttcctgaatgaagaagaatcaaatttagaaggcgtagtattatatatgatcaaaatgttacaaagcatagtaactgaaattattaaacatatcagcagaaccacctggcttgaagagactataatagatcaccatcaagaaaaaaacagaaattaaaagctttaggtgtcgaaggataggtaattaggaaagtataatcaaaagtggattccacaaaggttagtgctgtactttttgttgctgaaatcaacctttggcaattgatatataagtaatcaaattaaagttttccatcttacactgattcttttataggtaaaagtatgcattaggtaaataataaattaagaaaaagaaggtcaatgttcaattgcagtgttttaaaagcatcttccttagattataagcacccaagagacaattagcttaaaactataacactatagtaggagaaagagatctagatacacagacacacatacatacacaaatacataagtatatgtgagggtatctcaaatacagtatacaaatctatattgagttggctactattgttcttactgtaaagtttctaatgtagtcataaaattacaaggaagttaaaacgtcagaatagtttaccactcaaattagattgtgatattttatctaaatctaggcaaagccaactctttttaaaaatactcttcaggagagggtagtctcagatatcataactacaaaaaataatgtgcaatttaatttaaatctagatttttatatttagagacaatttagttatcgagttaaagaaattatgacatttctttttttttttttttttttttttttttttttttttttttttttttttaattgtaccttttattttggccctaactggtcacacttctgggaaggtgctgcttgtccagggtttgtgtaaccttttaaccttaacaatgcgtaactta

chr22:29065354-29065306(-) chr4:183051401-183052516 gagctc chr4:183048835-183051382 polyT chr22:29065448-29065404(-)

gagctc chr4:183048835-183051382 polyT chr22:29065448-29065404(-)(Primer gap) chr22:29065354-29065306(-) chr4:183051401-183052516 gagctc

Consensus sequence for insertion at chr7:146783241 (Twin Priming)

acattgtacttcgttcagttacgtattgctaggttaaaggattcattccacagtaacaccagcacctcccaaaatatacccaattactggcaaaaaatgttattattaaactgatgagaaaaataaaggtttattactggattaaaaaaaaaaaaaaaaaaaaaaaatattcatattaaaatttccttagagtttatgccattgattagtattcaggttgaaaacactaatgaagttctcttggaagctgcggtaggaaactgcttttggtgacatgccccactgggttttctaccatttcaagttgtgcttcaatgaggctcaaggtttaaagaaaaaatttatttggaaaaacacgacgctactctttgacttacagagctttcgtgtgcgtgtcttagctacataatttctacaggaacacacaggacatttgggttgcagatgcaaatgatatgtttattgctatcaatcattttgtatcaccagatcactggaccagaggacaacttgtaaaaggatttcagaatggcataatgggaagaaacacgctaataaaacgatgagacatgaagagaatttaaaatatacaatgatcagctgtgaagaattggccaggtaacacacagtaaaatgtttacacttacagaataagtctgtatgttttttagctcttaaaaccagaggtgtatggttctaggagagatatcacctatggcagcgatctcaacctttctggcaccagggcccagtgttttggagacaatttttttcccacggacaggttgggcttgggggtgggctttgggatgaagttgttcacttcagatcatcaggcactagattctcatgagtgttcatcctagatcccttgcatgtgcagttcacagtagggttagtgctcctatgagaatctaatgcgggcactcatctgacaggaggcagagctccccccactttgggtggaaatgattttaccaaagaatgaaaataaaatgctgtttctgtagtatccattttacagcaaattctattaaaagtgaaatagaaaatgtcattagacaagagccaatatttatagggccaaatagccatgcaaattgaggtacaaacaatgactttttaaatatgttgcatagagaaattcaccactttcgctacacacaaatgtttttaatgcacttacacacatctttgctagggatgaccactggttaaaaaaaacagaacaaagaaaattcatgtgtctcttcggtgtcctctttttttcagtgattttcctccccactctgccactcaatatccttttgttaaagagacacattgcaaagaatctgataacataatccacaatgagatactgtatctaatgatacttaacgtttctaagacaataatgggaacaatacttaactcaaaataaatgctgcagaggaacttgaggccccagagaacggttaataaaagacagcttgaatggaagagaggtcctcagtcactgaaggtccaccatgtagataataaccagacacactggtgaagtgtgcaaaattcagatacagttacacttaccagtgtctattttcctacaaaccacaacattctctctaagcacaatagagggcaagcataaaacttttctaaagtatcaggggtaacaggtgtaaaacttaacttgaattcgttgaagctctgtactagacttttctgaagtttcatcttgttttgctttgacaataattataccatactaaagatttaaaattgctgtttcccagacattatttgatatagttagagaaagtgctacatgttacatgaggaaggggtctaagattatgtatcttctgtatggccagactaggtggtcatgcttataattccagcgatttgggaggctggggtggagaatcgctggagcccaggagtttgagccagcctgggcaacatagagaccctgtctccacagaaataatttaaaaaatattagctacatatggtggtgcatgcctgtttcagctacttaggaggctgaggtgggattattgactgagccaggaaactgggctgccatgagccatgatcatgccattgcactccattctgggtgatagagaaatctgtctgtctcaaaagtaatttaaaaaaattaaaataaaaaataaattttatgaaaagaaaaagaaaagagaaacatattttctgtggatgtatcaaaagagcatagataaaaatttttaaatgttgctatttttatacttttgcttctctatatctattttcttctttgttttcatatttttgtttattttctgtaaactcatttattcttcaaacctctcctctttcaaaactcattgagcccagagatatccctttctttcctatttaaatatgattttgtattttcactacgttcacattaaaatgctcctttaaaaatatcatcattaaaaaaaaagtaatgtgaacatgtttaaagattatttgaacttacatttaaattggattataagtaacttaacattttttaatgcaaacatattaaccttcttgtgcttatgagattattttgccaaaattgtaggacagagaaactgctgactccacaaagatcttccctttgaccagttctctactaggagcttagtttcctgattttttagtacaatttgatgttgaggtttatatatttaataaggccggtacttaggtataataattgggtaaattgtaccttttattttggccctaactggtcacacttctgggaatattcttcccctatcagaaatttccatgttaccaaaattcttatctatctttattttaagatgttttccacatacatgcttagtgaaaggtgctggtgttaccgtgtggaatgaatcaacttttaaccttagcaatacgtaacttcc

RC:

ggaagttacgtattgctaaggttaaaagttgattcattccacacggtaacaccagcacctttcactaagcatgtatgtggaaaacatcttaaaataaagatagataagaattttggtaacatggaaatttctgataggggaagaatattcccagaagtgtgaccagttagggccaaaataaaaggtacaatttacccaattattatacctaagtaccggccttattaaatatataaacctcaacatcaaattgtactaaaaaatcaggaaactaagctcctagtagagaactggtcaaagggaagatctttgtggagtcagcagtttctctgtcctacaattttggcaaaataatctcataagcacaagaaggttaatatgtttgcattaaaaaatgttaagttacttataatccaatttaaatgtaagttcaaataatctttaaacatgttcacattactttttttttaatgatgatatttttaaaggagcattttaatgtgaacgtagtgaaaatacaaaatcatatttaaataggaaagaaagggatatctctgggctcaatgagttttgaaagaggagaggtttgaagaataaatgagtttacagaaaataaacaaaaatatgaaaacaaagaagaaaatagatatagagaagcaaaagtataaaaatagcaacatttaaaaatttttatctatgctcttttgatacatccacagaaaatatgtttctcttttctttttcttttcataaaatttattttttattttaatttttttaaattacttttgagacagacagatttctctatcacccagaatggagtgcaatggcatgatcatggctcatggcagcccagtttcctggctcagtcaataatcccacctcagcctcctaagtagctgaaacaggcatgcaccaccatatgtagctaatattttttaaattatttctgtggagacagggtctctatgttgcccaggctggctcaaactcctgggctccagcgattctccaccccagcctcccaaatcgctggaattataagcatgaccacctagtctggccatacagaagatacataatcttagaccccttcctcatgtaacatgtagcactttctctaactatatcaaataatgtctgggaaacagcaattttaaatctttagtatggtataattattgtcaaagcaaaacaagatgaaacttcagaaaagtctagtacagagcttcaacgaattcaagttaagttttacacctgttacccctgatactttagaaaagttttatgcttgccctctattgtgcttagagagaatgttgtggtttgtaggaaaatagacactggtaagtgtaactgtatctgaattttgcacacttcaccagtgtgtctggttattatctacatggtggaccttcagtgactgaggacctctcttccattcaagctgtcttttattaaccgttctctggggcctcaagttcctctgcagcatttattttgagttaagtattgttcccattattgtcttagaaacgttaagtatcattagatacagtatctcattgtggattatgttatcagattctttgcaatgtgtctctttaacaaaaggatattgagtggcagagtggggaggaaaatcactgaaaaaaagaggacaccgaagagacacatgaattttctttgttctgttttttttaaccagtggtcatccctagcaaagatgtgtgtaagtgcattaaaaacatttgtgtgtagcgaaagtggtgaatttctctatgcaacatatttaaaaagtcattgtttgtacctcaatttgcatggctatttggccctataaatattggctcttgtctaatgacattttctatttcacttttaatagaatttgctgtaaaatggatactacagaaacagcattttattttcattctttggtaaaatcatttccacccaaagtggggggagctctgcctcctgtcagatgagtgcccgcattagattctcataggagcactaaccctactgtgaactgcacatgcaagggatctaggatgaacactcatgagaatctagtgcctgatgatctgaagtgaacaacttcatcccaaagcccacccccaagcccaacctgtccgtgggaaaaaaattgtctccaaaacactgggccctggtgccagaaaggttgagatcgctgccataggtgatatctctcctagaaccatacacctctggttttaagagctaaaaaacatacagacttattctgtaagtgtaaacattttactgtgtgttacctggccaattcttcacagctgatcattgtatattttaaattctcttcatgtctcatcgttttattagcgtgtttcttcccattatgccattctgaaatccttttacaagttgtcctctggtccagtgatctggtgatacaaaatgattgatagcaataaacatatcatttgcatctgcaacccaaatgtcctgtgtgttcctgtagaaattatgtagctaagacacgcacacgaaagctctgtaagtcaaagagtagcgtcgtgtttttccaaataaattttttctttaaaccttgagcctcattgaagcacaacttgaaatggtagaaaacccagtggggcatgtcaccaaaagcagtttcctaccgcagcttccaagagaacttcattagtgttttcaacctgaatactaatcaatggcataaactctaaggaaattttaatatgaatattttttttttttttttttttttttaatccagtaataaacctttatttttctcatcagtttaataataacattttttgccagtaattgggtatattttgggaggtgctggtgttactgtggaatgaatcctttaacctagcaatacgtaactgaacgaagtacaatgt

chr22: 29065782-29065725(-)chr22:29065369-29065718 chr7:146783223-146784742 gagctc chr7:146782455-146783241 polyT chr22: 29065912-29065832(-)

gagctc chr7:146782455-146783241 polyT chr22: 29065912-29065832(-) (Primer gap) chr22: 29065782-29065725(-)chr22:29065369-29065718chr7:146783223-146784742 gagctc

Consensus sequence for insertion at chr7:152661949 (Twin Priming)

cttgtacttcgttcagttactgtattgctaaaggttaaagttcattccacggtaacaccagcacctcagtcacctttattagacatgtgtttatgtgtggaagtttcttttctttctttttttttttattatacttataatccaatttaaatgtaagttcaaataccttaacatgttcacattacttttttaattcacaaaacgttcttatctatctttattttaagatgttttccacatacatgtagtgaattttcttttaggagatgtaagaggaatctacaaatctttattctactatttcccaaaatatacaattactggcaaaaatgttattattaaactgatgagaaaaaaaaaaaaaaaaaaaaaaaaaaaaaaaaaaaaaaaaaaaaaaaaaaatcaaatgacccactttttttaaaagtaggtaatagacttaaacagacaatttaccaaaaggatgtccaaaggccaaaagcatatgacaagttgtcagtatcattactcatcagataaatgcaaattaaagctacactgagttatcacacacccaccagaatgactaaaattaaagactgacagtaccaagtgttggtgaggatttggagcaactgcaggaaaaaaaagctcaggctcccacagattctacattatggtgagatgtatagttatttcattatacattacaatgtaatcataatagaaataaagtgcacaataaatgtaatgtgcttgaatcatcccaaaacatcccccccgaccccagtcgtccatgaaaaatttgtcttccacaaaatggtccttgatgccaaaaggttggggaccactggactagaagaatttccacttgaggggcacttactgccttgctatgagatgttaactgaagctacccttatcctaagaggaacagcaatgcccaaaagggttccatgataagacggacatggttttacataggatcttgatacctgggggaggcacagaggagatattcacaagcaggaggcctttttcccctaggaactgtgtgagctgctgctggatttacagtgcctgataaatgcctctcacgtgactgacaaagagctgtgtggttggtggaggcagctcaaagtaaatggacaacatcttgtttagaaagccgctactgtgattgaagatgagtcaggaaaatctttttccttttttgagctatttatagctgttatagctcaaaagaaatatggtatggtatccaccatcttgtctcaccactgcccgagacacagacgtggcttctgttagtaagtccctattaaacgtttcttccctaaggtggggaaaaagagtcctagacctacaggtatactaacttatcacaagttaatttccagcacaagtgccctggaagaccagcccagttaactggacacctgtgtgggacgtgataagcctcatcttccaatttgtacagcatataaaacttgattcaaattggatcatagacgtaactgtgaaaagtaaaaccttaaacctttttaggagaaacacagcagaatactcccatggggaggaaaatttcttacacagaacaaaaacctctaaacataaaagacaatattgacaaattcgactgcatcacaattaagaacttattttttgccccattctcagcaactaaaagaaacttttattcaccaaagataccactaagaaactgaaaggcaagacatagactaagagatacatatatttgacaaaggacctgtgtacacaatatgtaaaacttctaaatcattaagaaaaatcaatttgatattgaggaacatatatttaataaggccgggtacttaggtataataattgaattaaattgtaccttttattttggcccctaactggtcacacttctgggaaggtgctggtgttaccgtgagatgaatccttttaaccttagcaatacgtaactt

RC:

aagttacgtattgctaaggttaaaaggattcatctcacggtaacaccagcaccttcccagaagtgtgaccagttaggggccaaaataaaaggtacaatttaattcaattattatacctaagtacccggccttattaaatatatgttcctcaatatcaaattgatttttcttaatgatttagaagttttacatattgtgtacacaggtcctttgtcaaatatatgtatctcttagtctatgtcttgcctttcagtttcttagtggtatctttggtgaataaaagtttcttttagttgctgagaatggggcaaaaaataagttcttaattgtgatgcagtcgaatttgtcaatattgtcttttatgtttagaggtttttgttctgtgtaagaaattttcctccccatgggagtattctgctgtgtttctcctaaaaaggtttaaggttttacttttcacagttacgtctatgatccaatttgaatcaagttttatatgctgtacaaattggaagatgaggcttatcacgtcccacacaggtgtccagttaactgggctggtcttccagggcacttgtgctggaaattaacttgtgataagttagtatacctgtaggtctaggactctttttccccaccttagggaagaaacgtttaatagggacttactaacagaagccacgtctgtgtctcgggcagtggtgagacaagatggtggataccataccatatttcttttgagctataacagctataaatagctcaaaaaaggaaaaagattttcctgactcatcttcaatcacagtagcggctttctaaacaagatgttgtccatttactttgagctgcctccaccaaccacacagctctttgtcagtcacgtgagaggcatttatcaggcactgtaaatccagcagcagctcacacagttcctaggggaaaaaggcctcctgcttgtgaatatctcctctgtgcctcccccaggtatcaagatcctatgtaaaaccatgtccgtcttatcatggaacccttttgggcattgctgttcctcttaggataagggtagcttcagttaacatctcatagcaaggcagtaagtgcccctcaagtggaaattcttctagtccagtggtccccaaccttttggcatcaaggaccattttgtggaagacaaatttttcatggacgactggggtcgggggggatgttttgggatgattcaagcacattacatttattgtgcactttatttctattatgattacattgtaatgtataatgaaataactatacatctcaccataatgtagaatctgtgggagcctgagcttttttttcctgcagttgctccaaatcctcaccaacacttggtactgtcagtctttaattttagtcattctggtgggtgtgtgataactcagtgtagctttaatttgcatttatctgatgagtaatgatactgacaacttgtcatatgcttttggcctttggacatccttttggtaaattgtctgtttaagtctattacctacttttaaaaaaagtgggtcatttgatttttttttttttttttttttttttttttttttttttttttttttttttttctcatcagtttaataataacatttttgccagtaattgtatattttgggaaatagtagaataaagatttgtagattcctcttacatctcctaaaagaaaattcactacatgtatgtggaaaacatcttaaaataaagatagataagaacgttttgtgaattaaaaaagtaatgtgaacatgttaaggtatttgaacttacatttaaattggattataagtataataaaaaaaaaaagaaagaaaagaaacttccacacataaacacatgtctaataaaggtgactgaggtgctggtgttaccgtggaatgaactttaacctttagcaatacagtaactgaacgaagtacaag

chr22:29065404-29065510 (+) chr7:152661940-152663136 ctgcag chr7:152661726-152661949 polyt chr22: 29065887-29065659(-)chr22:29065283-29065354(+)

ctgcagchr7:152661726-152661949 polyT chr22: 29065887-29065659(-)chr22:29065283-29065354(+) (Primer gap) chr22:29065404-29065510 (+) chr7:152661940-152663136ctgcag

Consensus sequence for insertion at chr12:33708291 (Twin Priming)

ctgtgtacttcgttcagttacgtattgctaaggttaagactactttctgcctttgcgagaacagcacctcccaaaatatacccaattactgttcatgatttttttttaataagagtaattttgcatggaattgtgtatatactagatgtttattcactggcctttttttgcaatttaagtttatatagtcagaaccaatattgctttattaaaggaaacagattgttactcaatagatggttcagccttaatgtctatgattgtttaatttccttgatttcatttttccttaatttaattttgctcctctttcacattcaaatatgaattactgcatgaaacactacctaaagacaaaagatgccaagtcacatcattggataaagcaatatgaaacacaaaagtggtacgtgcctcactagggaattccctcaaaattagagacacaaagtgaatttgtcctagaaatgaagaatagagcacaaaacaggacgatagggtcttctttgattatgtttcaagtcaaagaatgaaaacagcaaaagtttaaaaatgagaatagcttttattactgaaacattttctcttttggcctgatttctacagtgatgctagatgcatttccttttgcagcttcatgtttactactaggtacagatgatgtttgaaactccgcagtgccaaaaccagaaatctctacaggcattgtggaatgcttatcctctggcctaccatattaattttcagccatcatattgctcatagagttatataattagtccatatttggaacaagacttaggcaattttgattacctctaagatttgttgagaaataatttaatcaaattcaacaaatataacaatatattaagtataaatgtttaagtagtgatagttggatatttggatttattaaagagaagacttattcatgacatttgttaaagtatgataaggtagccaattcgggggactatcaagataggtagaaagtcacaatggagttttacagaggaaagacattgagctccactccaaataaaaggaagagaaaagtgggaatgtatagtcaaggagtagggtgggtggggaggtcagtggacagaacattactaagaggatgtgatggttaatactgagtgtcaacttgattggattgaaggatgcaaagtattgatcctgggtgtgcctgtgagggtgttgccgaaggagagtaacatttgaatcagtgggctggtaagtttgacccacccttaatctgggttggcaccatctaatcaactgccagcatggccaggatataaagcaggcagaaaaatgtgaaaaggctagagtggcagcctcccagcctacatttttctcccatgctggatgcttcctgtccttgaacatcagactccaagttcttcagctttgggactcacactggcttcctcgctcgtcagcctgcagatgctcagcaccagcccgtgaaaccaaagccacaggggcatagctgcccctcttgcatcacctcttgcatcagcataacatagatgtgagacatgcagtctaggagatcattttggagctttaagatttgactgccctgctgaattttggacttccatggggcctgtagcccctttgttttagtcaatttcttccatttggaatggctgtatttacccaatgcctgtaccccccattgtatctgggaagtaacaatctgcttttgattttacaggctcatgggcacaagggacttgccttgtctcggatgagactttggattgtggacattttagttaatgctgaaatgagttaggactttgggggactgttgggaaggcatgattggtttgaaatgtgaggacatgagatttgggggaggggctggaatgatgtgttctggctgtatccccacccatatctcatttttgaattcccatgtgttatgggagggacccagtgggaggtaactgaatcatggggggcaggtctttccatgctgttctgtgatagtgaataagtctcatgagatatcatggttttttaaaagagctccctacaagctctctctctttgcccactgccatccatgtagatgtgacttgctcctccttgccttctgtgatgattgtgaggcctccccagccacgtggaagaggtccattaaatctctttcttttgtaaatttcccagtcttgggtatgtctttatcagcatcatgaatacgaactaatacaatattcaagtattctttgtgtacatatgctctgttttcatttctctcaggtaaatatttagaagtggaatgatgtatgtttaaaatttaagaaaaatgaaaccttgttttccaaatttgctatgcaattttacattcaaacagcagtgtgaataggtgtaacagtggctctgcatccttacaagatgtggtataatcattcttttacattttagctattctaataggtgtgcagtggtgtcttattagggttttttttcttctatttcctaatgtgtaatcatgttgagagcattttttgcatgtgcctatttactatttgcacatcttctatggtgaaatgtcagttcaaactgattggctttttttttttttgttttattgaattgtttgcattcttttttattgacttttgaagattatttgtatgttatactggatacaagttgtttatcagagatatgacttgaaaatatgttctatgaatctttaccttttatttcattctcttaactgtgtccggtagagtacagtgcttaattttgaagaagttcaaatcattaattattcttgtatagtttagctttggtttcatatctaagaaatattttctaaaacaatgtcacaaatattacttgtcgattttcttctaagtttttttaactttagattttacatttagtctatgatccatatttttaaacttttttgtaatatggtatttgatgtagagaggttcttttttattagcatatagaaacccaatttttttcagcatgatttgttaaaaacaactattcttttaccattgaattgtatttgcatttttaaaaaaacagttgatcatatatattaggatatatttctggactgcctattctactccagttatctattttcctactttaagatcaatgccatgttatctgtattactgacacattttaattcttgaagttaggtagtcaaaatcctccaaaattacatttttctccaaaattgtgttagctattctaggtactttgtattttcatttacactgaaaaaatcagtatgtcaattaaaaactaagctcattggatttccgtttagattgcactgaatcagtagaaaaactcgaagataactgacaatcttaacaagttaagttccctatccatggatgtggtacatttctccatttgattagccttgtttaattcctctcagcaatggtttatagtttttccgtgtataggtcttatacatttttgggtcaaatttatcaataaaatttaatatattttatgattttgaaaaatatacttttactagttgcaaattcaattcttcattataggtacatagaattactacctttttcctactatataacctgtattaagcaaccctgctaaacttacctattagtcttaatagattttttgtagattcaaagaccttttttgcataatcatatctagaacaaataaagatagttttatttcttactttccaattctgatgtattttatttcctgtttcctggctagaacttgtagcataatgttgaatagaagtgttgagattagacatctttaacataaggggaaagcaattagttaaacatgttgttaaacataaaggaaagcagttagctttttcattgtgaagtatgatgttagctacagattttttcatagatgcttttttcaatcagacataggaagtttctgtccccttttagtccttctattcctagtttactgaaggttttattagaaatgaatgttgaatttttttgcaaatgttttttcttcatttatttacatgattatgtgtttttttaaatgtttgagtcatttaaaacaatgaacaatttcaattaattttgaatgtttaaccaaccttgtctttctaggatatagtccattagtcataatataactttgtatttgatttgctaaagtatattggtttggtaaactgtcattttttctccccctccattcccctctatgtgcataattttaaaattgtgttataatgtaatataatattgtgtgtaatgggtgataaggaagagagaatctgatttgcttgttttgaggtcaagttttttatttgtttgcatattttaagcaatgcccttattttattctggaccagtgcttgaatcgctaagctgctaacaagttcagatacaatttattgtttgttactgtatgggctatctgggaatagagaaactattttattttagtatgccatattcaaaggagaaatgatgaaaagtttaagaacattttataaaaagataagtaaaatatttatttatatctttataggtgtgcagtggtatcttattgtggttttcatttgtatttccctaatgagtaatcatgttgagcattttttgcatgtgcctatttaccatttgtgcatcttctatggtgaaatgtcagttcaaattgattggctgttttgttttttattgaatttattgttttttttgttgtttgcattcttattaaattttgaagattcaatatctagatacatatcaatatctagataaatagaatggtgagtattaacaaactgatttaaaatcagacagactgtattttatggaatgttacaatacaggataacaaatctcaggcaatattataggcacaatttaaatatctaaataatgccatcatttaaatgttcataaagacatgaatgcaagtctcaagttgaagtagatgataccttaaggttacttatagttcaaagacctgtaatgccatgagttagtgaaaagagcaactaaatcctatgttaatgtaatagaaactcaatgtcttgctacatgaaatagatggtaattagataatgattataaagggaattgttctattgcatcataattttcatgattttttttttttttttttttttttttttttttttttttttttttttaaagatattaaagtctttatttgccccccattttattttcagtaatacaggggtaaaatgagtaggattacttgatctggccatctatggccatttatggaatgacaaactaagttcaatactgatagagttgtattctgtgtatacatataataaattatatactgtgtgtataaaaattcaagcatatggaaaaacttatttaatcagtaataaacctttatattttctcatcacaaaacgttcttatctatctttattttaagatgttttccacatacatgcttagtgaaaggtgctgttctcgcaaaggcagaaagtagtcttaaccttagcaatacgtaataa

chr22:29065832-29065854 chr12:33708277-33709645 ctgcag chr12:33704599-33708291 polyT chr22: 29066121-29065877(-)chr22:29065722-29065782

ctgcag chr12:33704599-33708291polyTchr22: 29066121-29065877(-)chr22:29065722-29065782(Primer gap) chr22:29065832-29065854chr12:33708277-33709645 ctgcag

Consensus sequence for insertion at chr2:78612537 (Twin Priming)

acagttgtgcttcgttcagtttacgtattgctagggttaaaggttacacaaaccctggacaaacagcacctttcactaagcatgtatgtggaaaacatcttaaaataaagatagatgattttgcaattattatacctaagtgccaggccttattaaatatatgttcctcaatatcaaattgtactaaaaatcagaaaactaagctcctagtagagaactggtcaaaggaagatctttgtgtggtcagcaacgtttcctctgtcctaatttggcaaaataatctcataagcacaagaggttaatatgctttgcattaaaaatgttaagttacttataatccaagatttaaatgtaagttcaaatgccttaacatgttcacattacttttttaattcacaagaaacgttcttagtttataagcttctatggttgtgataaatttcagccccacagactaacatacaaaccactgtgtgaaaaattattataactgttatacgttgtgcagaaaattattgttcaatacagagatataacatagtaaacaatggaatgactgagacaagagaggaccaatgaaacaaccactttcaaccttacttttatcatgtgacaaaatataataccacatgtctctttcagtacctggctacatggcttagatgacaaaacaacagtatcttaccttgggcaacactgaatctttgagaaaacgtggaaaataaacataaaaattatcaaagttatttagttataagaatagagctcttttgaagaaaagatgaagggcttaaaggttgtattatgatgagatggcaatgaatatgtgcataagataaacacgtttattaaatcacgcttgccaatgaaggttgtgaatgagcttctatcgaaatagcataaggttatctgcttatgaaagtaatgaggttaatacagtcttttgaaaatataatcctacttctaaatgcctgtaaaatggaaaccacactaactctgctgagttatgttaataactggtgaaagcgtcttattctgaagttacttcaaaaaccaattattccaagtgagctaccaggtgttgtggaaggaagttagtttggtttgggttttttttcaagtcacagctaggagtactcagagcttctgaaattaactatcactgcacatttgaatattttaattatacacttcaaaaaaatggcagagtcatttgactgaagtgcaatttattatgctctttatttagtacttaaatcaactatgaaatagcattttgaattaaaatcaatacactttgcattcactctgttcaaagaaaattgaagctaattataactctcaaattaaacaaatatggagactaacaaagcaattttagaatatattacattattagatgtgagataactatatttctatgcttctaaaaaaaaaaaaaaaaaaaacagaactaccagtataataaagaaaaggtgaaggaggtaggatagaattgaaataattctttgtgagttttgaagaataaatttccaaggaggattatgtgtgcaactcaataagtgccctttttttcaaggtcccctcttttactagtttacctgagaaaacagggagttgatttgtccatgtggttcctcctcagtctatgatttacagtgcatctctgattatttaaaccctaggcttccagctagtgttgttatgagtaaataactggaagttggagattacctacatttgaaatcatgctgccctcatcatttcaccccacaattattgggtgctggcaggagattactacacttccctgtaattggcttgatttctcttaaactacactattgaacaatcattgtttataaaagtctttgtaggtgctgctagtttgtcccacttcaatggaaaatgtaaatgacttccccaaactatataaaagtcaaactcatactccaccccgtctcctaataaaaatacaaattagccgggcgtggtgacgcagctccccagctatcgagaggctgaggcaggagagacagcttgaacccggccttgggcggaggtggcgaaacagccgaaatcgcgctgcactccagcctgcgcgacaagagaagattccgtctcaagaaaaaaaaacaaaaacttatactccacccctagtaatatcttatttcctttccttgcttccttgttaacactttacattatcagataatgtaaatgcttgtcaatgcagtgaaagtatattttactttcatgaagtcaatatcctttaatattggattaatattttattactgctgcctatttctaaaaatttattattaactttttgttgggttttttatattgtggtacagatgatgtcttttgttatgtataaagtgtgtgtgttatagtataatgtacatacgtattatgtgtgtgtatattttatatatgcatggcttatttcataacataatgtctcaagttacatccaagttgctgcacatattcaattctgtgagcctggagaacatccaggagtggtttgcattcttaacaaaagacctcagaagattctgattcaagctgatcattgaccacaatttgaaaattattgcacttgagtgttagcttgttaatttatgaacctgaaggcaagcaatatacactcatggatgataactacatggatatttaaaactgctagtttgttttggtgaaatcgtaccaggaggctatcagtgagtttcaagcttattacagaatcctataattaattttttttttttaagtaatatggttttcagtcctaaactgcattttaagacaaaatttttatttgatgaagggcaggaattggtcttgttttggatctttgtcttataactagagaacacagagggcatctgattcagggctggtctgcatacacatatgagaaaaattaaggtaaagacactgtatgccctaatctgttctagaagggttcgggtttacatcacatttgaatgtaaaagagatcaatctcttcccacattaagaaattgaaaggttggatttcacaaattgaatatcagggaaaaggtgaatagtttatcgtcttttttttttttttttttttttttttttttttttttttttttttttttttttttttttttttttttttttttttttttttttttaagatattaaagtctttatttaccccatttttattttcagttaatacaggtcacaaaataaattaggatgccagatctggccatctatggccatttaatggaatgacaaactaagttcaatactgataggttgtattctgtgtatactacatataatgtcagtatatactgtgtgtgtataaaaagttcaagcatatggaaaacttatttaatcagtaataataaacctttatattttctcatcagtttaataataattttttatgagtaattgggtatattttgggaggtgctgcttgtcaaggtttgtgtaaccttttaaccttaataacgtatca

chr22: 29065782-29065738(-) chr22:29065453-29065738 chr2:78612530-78614538 atgcat chr2:78611887-78612537 polyT chr22: 29066118-29065832(-)

atgcat chr2:78611887-78612537 polyT chr22: 29066118-29065832(-) (Primer gap) chr22: 29065782-29065738(-)chr22:29065453-29065738chr2:78612530-78614538 atgcat

Consensus sequence for insertion at chr3:99147126 (Twin Priming)

ctcattgtacttcgttcagttacgtattgctaaggttaaagactactttctgcctttgcgagaacagcacctttcactaagcatgtatgtggaaaacatcttaaaataaagatagataagaacgttttttgtgtggtggggtgggggagggagggatagcattgggagatatacctaatgctagatgacacattagtgggtgcagcgcaccagcataacacatgtatacatatgtaactaacctgcacaatgtgcacatgtaccctaaaacttaggagtataataaaaaaaaaaaaaaagaaagaaaaagagaacttcacatgggaccacatgtctaataaaggtgactgaatcccaaaattatattggtaacatggaaattcctgatagggaaagaatattcccagaagtgtgaccagttaaggggccaaaataaaagtacaattacccaattattatacctaagtacccggccttattaaaatatatgttcctcaatatcaaattgtactaaaaaatcaggaaactaagctcctagtagagaactggtcaagggaagatctttgtggagtcagcagtttcctctgtcctacaatttggcagaaataatctcatacgcacaagaaggttaatatgctgcacattaaaaatgttaagttacttataatccaatttaaatgtaaagttcacataccttaacatgttcacattacttttttaatgcaatctgtttctttatatatttattgaaaatggaaagaaaaatgcctaccttacatatttttttgtgacaattaaattagataacctacataaaggtatataataaacattgaatatatgtaatcattactattttttaacttttgacttcatctactcctatcaccacccactgccccgcaccttggagctatcctatacctgaggtaacaaacagatttcattacccaaaccaaattggtgaaagaggtaatgtttgttttcttgagcctacactaagaaaggattctaagcaaatcagactcagcgaaaacaatgttgaaatagatttgcaggagtctgccattgggctgaaaagagtgttagttattactgatactgtatactacattgtagctaatttgtaatacatgtggtccttgtatctcctacatccataaatttatcttctgcttcatctgtccaaaaatagttttattgttctctcattttcccttctacctgtcaaaatcttactcattccttaagacccatatcaaatgtaccccttgttataaacctgccctcctgagtactactaactgcagttttgaatcagtaatcatatttatctgagcttatataacataaaattattgccagccaattttactcaatctttaatcattaatttacttaaactttttaaataaaggatatttcagttaaggttttgttgatattatgcaaaaattcaacagtactaacagcactgattaattttcaaatcttggtcctttaggaggttgaatgagtttctcttttctttattcttttagcataagatcagagacctacttttaagggaggcctttctttgtaataaatactttgcatcatctctaaataaaaaaaaatttgcttgctgtatctcctgttcctttaacaactgctcagatgaatgcattcatgattattatatatctccatgactttattacgctctaaacttctaaagactagtgaacatgttctctagtataaaccataagagcctagaaacatgttcaacatttggttaataatttatcttaaggtctattggctgttgaacacaaatccttttttttactctcactgcattatgaaagactgtaattattttttaatgcaagatactacattgaattattagaaaatttagtattcgtaatatagagattaataagtctattaactaccacagtttgatcttattataagaagaattagtgggtgttgcttttctcaccatctattttcatgtctgtaggctacttttgttttagtgtctaattaccctcttttttgtgtctccttatctgccttggctgctttactaagttctgggagatacatgcattacctgccagctacacagaaatattcttcagaatgataacgttactgggtattactataatataaattctaattgatggttagagttgggaataaaaacatgaaatacctttcaccttgccatgctcaaaaatttcaatatgattaaaaaagaacacaggaattgaaatttgctaacctacatgctttcaacaaattggaagtattctactgtgtatcagccccagaggggctatcattctgacactttctcatcagaattaaactacaaattcaataataataatagtaataataacttagctaagtaggtcagactgctgagccctagcactaagtttatttatgactgggcagaattctctaccatgaaagaatgaaaacgacaataaatctaccttgtgcttaaaaaagtgagttaatgaaaaagagtatatctctgataacatcacattgttcttttaacttaaatcttgtaactttttctcatttctcccctccactgtacacatttcaatgtcattgggaaatgtgcccattttttgttatatcctggaactgtctagagttccattgtgtgtttgttgttttttttttttgactagaccaaacttagtaggttaaaccttatatatacaaattgatagataatctccatctgtatattccataagtgtctcaaacttaactcactctcttcctgctcatgtcaatttaagagggccaaactcagaatttcagaaatgttaccttttcttacagtcagccaagtctatgaatttcattttttgaagctataatttgattccattatctccttttactcatactccacttctctgccaacaaattgattgtcccatctcagtctccttagtttttcatatatttttcatcctgccaccagagttttcttcctacaaggcaaatctaatcaaactgttctgctcaaaaaacctccaatagcttcctttatgtacagaataaatttaaaaaattattaacatgaaattaaggcctggcttgtgactgatttcagtttacctccatgttgttttaaatgagattttagtatcataatgtgattaagaatgcaggttttagattttaaaagccctggtttaaactcttactcccacattttcgaataagggaccttggaaatggttcttaactctgcaatctgtttctttttttttttttttttttttttttttttttttttttttttttttttttttttttttttttttttcatcattaaagatattaaagtctttattgccccccccattttattttcagtaatacaggcgaaaatagagtgggattatgatcatctggccatctatggccatttatggaataacaaactaagttcaatactgatagagttgtattctgtgtatacatataatagagtatatactgtgtgtataaaaattcaagcatatggaaaaactttatttaatcagtaataaacctttatattttctcatcggtttaataataacattttttgccagtaattgggtatattttgggaggtgctgttctcataaagatagaaatagtcataatagtcagcaatacgtaactt

chr22: 29065782-29065726(-)chr22:29065138-29065721 chr3:99147111-99147799 ctgcag chr3:99145121-99147126 polyT chr22: 29066126-29065832(-)

ctgcag chr3:99145121-99147126 polyT chr22: 29066126-29065832(-) (Primer gap) chr22: 29065782-29065726(-)chr22:29065138-29065721 chr3:99147111-99147799 ctgcag

Consensus sequence for insertion at chr4:90987152

ttgtatgtacttcgttcagttacgtattgctaaggttaaagttcattccacagtaacaccagcacctcccaaaatatacccaattactggcaaaaatgtttattattaaactgatgagaaaatataaaggtttattactggattaaataagtttttccatatgcttgaattttttatacacagtatatactctattatatgtatacacagaatacaactctatcagtattgaacttagtttgtcattccataaaatggccatagatggccagatcaaataatcctactcattttacctgtattactgaaaataaaaatggggggggcaaaataaagactttaatatctttaaaaaaaaaaaaaaaaaaaaaaaaaaaaaaaaaaaaaaaaaaaaaaaaaaaaaataaaatcaattgctcaattttctatatcagtgaacaagaacactaaccaagaaaaaatgaaatttatgtttaatctatccatttagatcagatggtatctcagtcataagggaagcaaagagctcaggagttccagagcagcctgggcaacatgacaaaatccatctctaccaaaaacaaaaaaaattatcaggtgtggtgacgtgcacctgtcgtccaaactactcaggtagctgaggtgggaggattgtttgagcctgggaggtggaggttgcagtgagccgagatcatgccactgcactccaggctgggcgacagagcaagaccctgtctcaaagaaaaaaaaaaaaaaagaatgaaggtgggaggaagggagcaacaggacgaataacaaaagcacaatataaatatttattattaaaaagataaaagaggtaatagcacaaacataatgaaggctctctcagtagtgtgtaaattttgcttaaataaatgcctaagacgtggtgttcataggagacgctttccaaatttgaaaaaaaaaaaaatttctatcttttgtcttgggggatctgtcacatgtttgtagaaatacagtggcacttctgcaaacaaacctccccaccgtcacatctagccaggtgtgaatcacaaaaccagcatgtgactcccatcacaatggaaataaggaataaatgaggctattaaaatgtaaagtctcttaaatgaaccacgttaacattctcctcttcaaagacagtcttagttttgtaacatccaaacttatttttataactcacacttctttagaatatctcaatttgaatatcaatataagattcaaaataatctagtatctcccttcaccacatttttcacagtaaatatgaataatgcacatggagcattgaaatatttttttgctgcttcatagaatttagagtatggtcttatacaaaaaaaataacttaaagccttttgatatataattgttcatatcatcaattactggtatacacgtacacagagtaaagaagcttttccatatgaagggatttagaaacatgtaatactataaatctatacccttggaattgaaggaaagttctagccctgtgtctgcacctgtttctttatgggacagaatataaataactaatactgtcaagtttctgggggggtaaaataggatttttcctacattgacacaaataagctctaagaatatgtagcttttgtattagcttttctattagccatatgttctgtgtcccagctgttaagatctcctacagctgattaattatatttttttttaaaaaagtcaggcaatttacacctgtgataccatagatcacttaaggctaatgggctggaagtccttgcgatattagggatccttcagggtatgggtaggatgaggaaggaacataatctgtgcccataatctagcacagtttcctctttactaatgcttagggtttagataaatgtctcattaagaggtaaatgtctcattaaggttattagggttattaggttattaggggtttaggtaaatgtctcattaagaggaaatgcatttgtcagcaagggctgtaggctcatagggtatgattgcaaagacttttctgaaagcttagctttggataaatcttcagctagcctaggcagtgcatattctatcctgtgaaaacaattggtgaacactggtgaaaaaaaagtgtattagcccacaggccacagaggtgtttaaagaattgagcttatgtacatcaatgttcaataggaagtccagtttatttcatatatagggtcaacagttaaggaagaaactgggaaagaggctgtgtcatgggaccaacagggagggtcagtaaaaatattgagtaaagaaaagtttctttacaaggttttctgtttgtttgtttgttttgttttaaaactagttgattcaagtaatgcaattacctgattttatagctctaagacagttattttcactgtttaccaaatggattggggaattttgaagataacttaaaagccctgaacaaatgttttagaactttttcttaggtttcgatacaagagaggcatttgaagtaaccaccagtttcttcaatttctaagatatcttttcttcttttttctcacagtaaatgtgagttttttgtttcccttccttatagagttattaaaagcagtaaaagattatcagtgtccacaacttcagctgtggtcatcattgggaaaagctgtcataaactttaccttaataaaaaatcacttcacatggggccacatgtctaataaaggtgactgaatcccaaaattatattggtaacatggaaattcctgatagggaagaatattcccagaagtgtgaccagttagggccaaaataaaaggtacaatttacccaattattatacctaagtacccggccttattaaatatatgttcctcaatatcaaattgtactaaaaaatcagaaactaagctcctagtagagaactggtcaaagggaagatctttgtggagtcagcagtttcctctgtcctacaatttggcaaaataatctcataagcacaagaaggttaatatgcttgcattaaaaatgttaagttacttataatccaatttaaatgtaagttcaaataccttaacatgttcacattacttttttaattcacaaaacgttcttatctatctttattttaagatgttttccacatacatgcttagtgaaaggtgctggtgttaccgtgggaatgaatccttttaaccttagcaatacgtaacttcc

chr22:29065832-29066121 polyA chr4:90987149-90987271 gagctc chr4:90984996-90987152 chr22:29065287-29065782

gagctc chr4:90984996-90987152 chr22:29065287-29065782 (Primer gap) chr22:29065832-29066121 polyA chr4:90987149-90987271 gagctc

Consensus sequence for insertion at chr6:74978206

cttgtgcttcgttacgtattgctaaggttaaaggttacacaaaccctggacaagcagcaccttctatggccatttgaatggaatgacaaactaagttcaatactgatgagaggagttgtattctgtgtatacatataatgagtatatactgtgtgtataaaaaattcagcatatgaaaacatttaatcagtaataaacctttatattttctcatcagtttaataataacatttttttgccagtaattgggtatattttgggaaatagtagaataaagatttgtagactctcttacatctcctaaaagaaaattcacatataagcatgtatgtggaaaacatcttaaaataaagatagataagaacgtttttgtgaattaaaaaaagtaatgtaggaacatgttaaggtatttgaacttacattttaaattggaccataaagtaacttaacattttttaatgcaagcatattaaccttcttgtgcttattgagattattttgccaaattgtaggacagagaaactgctgactccacaaagatcttcctttgaccagttctctactagaacttagtttcctgggaatattaaattcttaaagacttcaaccttctggccaaaattgattgatactgaaagtaaatctgacctaagccatgacaaaggaaaccatttttctggatttggagacctgaatcaagaagtcatctggtggcaggagctctaatacataaaactgggaggtgtgaggtgccatgttttcaaccacaaaagcttctctactgcaactaaaagataaagccaaaagtagaagaagaaagtggaaatggagggcaagtcctgatttcactggagtcctggatgctcttgagaactaatcacataattgccctcagatttctgtattatgcccattagattaaccagaaattattcattgggatacaagaacagaaaactcaaacaacagcagcttcatttttgttttgtttgtttgccttttctttttttccattccgtgatgtcagagctggaagtaagggatgccatctaggactaagttacctccatcttccctgttctacacttagcatagaaagagcatttgtcataataaagagaatgttgcacctcttgagtttggtggtgagaccctgaaggcaatatatctgattttcaggcagaacagaataaagaatagcctaaagggcaaagtgtaaagccccttctctttttttgaaattttattttgagaatggggtcttcccagggactccccaccagagcacatggtcactcctagcttgcagaggctgaaatttctaattttctggtttttctaccttaagaatttggacctcatacattactagcacataggggaagatgttcaacattttgattgtgcagaaatgaaaaatatctactacccccctagtatgccttgactaatctatctgtcattaagcttgttgagtttctgaaacttatggggctaatttcttttatttatttctaagtttataatagtaacctcatgttatcttgtttctctgtcatcttgatatctcaactctcacacataaaagcaatagctaaaatgaacatcttaaacacaagagtagtaatatcttgagtggctattttttagtgagtgaaggaaggggcttaaatatagacccaaattttaatagcaaaggtttcttgtaagtctagctttctctggccttctcctcttataagactattaccttttcctctgaaaaggatgttatatataccatctagtggtgttttctaccacaagagaagaatgcgacatggtgtgtggtgcaaaggtgagcctccaacaaatgttagctctcactgttaaaatctgtctctgtgcctgagctcttctagcagctccatattctttttatccaattacatcttggacattttttgtgaacaccaccacatatgcaaactaaaaaactgttcatcatctttccaaaggtgggattttcttacaaatttcatgaaacacaatgaccgcagtcctgggcttggcattatgctataccattatgaaaagggcctcttcagccttctttcttttcttctcctcttacaaaccacttgtcctgtgacttgattttttaggctgcccatttttttctctgtcccaatacctggaatgcatgccttcctaggcctaacccaacttaacttgagacttggtcttctcattttttcatgcatatttggacctgttacatgcctctggccttcaatactcaacacaatagttcccaaaagattgcctgctcagagtagtcctcaatcctattcttcctgaattgtctgtaatgtagaattctaatttaccacctatagattttttactctctggtcacatacagccacaacccttcatcctttctcatgctcttgtttcatgtcaccttcattaatttccatatatgaccaacccaagaacaagctacttaatttgtagggcccagtataaaatgaaaatgttgaattttctatttagaaattattcagaatttaaagatagctacagcaagagtattaaatgaagtatggggcccttctgagtgtggaatcttgtgtgtgactccatgagtctcaggcccatgaagccagcctgattatgagtctattcaaggcttttttaccacacattttctcttactgaatgtactttctagctggtttctctgacagtagctctcctggttgtcttataaatatatattttttaatttccagtggttccccatccactagtcactcatctttactccctggttaaattacctatacttcctctgaaggaccatagctttcatagtctgtgccacataggagactgtcttggcaaaaatctactgtctggcatgtagtttttttctatgggaaggttgttggcctcctccatcataagatttggctctatttttatctctaatactcctgatatgtaatgcatttaatacctgccttgattgtctaaagggttttgagtctaattactttccgtatactatacaaagaaggcctttaactccagacatttaaatgactatttttttcaggaaaggtctgagaggcacttctgctcattctttgctttagaataagactgaccaagatcttacatgtatagtgtgcagagagagagagaaagaaaaatagaagaaaaaaaaactgaatatattcagttcctcctaaattggtgagtagatatcctggcttttttaatttattagtagtgtaggctttagatatatatgaatctgcctgtaaactcaaaaaatgcattccaagtgaaatgggcacagtcagttgcatattaatgaatttatggtttctattctcatctattagtgaagataactataagtggaatttataaaatgaacataaaagaaatgaatgaattagtaaagtcttatttcttcttatttattagaaattggatagtgtcacaaaaatgtgtattcaacaacattgggacttctatttatttattcaccatgtattaattactattagtggagattggaatttaaaattaataacatatggtctctgatttctgcatatcaagagaaataaaatcttatgtcttctagaagaagacatgggtcctttctctgatatcggaggtagtcagagtggtggcagcaacaagaaataaattatttttggaactgaacaaaaaaaaataacaagagcaaaggtgatgaaaagaccttcccaggtgatggaatacagccttcctctactccggagaaacttttgctgagaaacaagcaactgcagaatttgttaacaaaataaattgaactgctgtgttcagaggaattgaactgctatgtatccaatcgacttggattgaaagattcagtatttttgcttttctagaaattaccaaaatttatttatgtcatagaaacgtaatctctatttgcctcatatctattaaaggctggtgcctaaaaataaacactaaaattaaaaattaaaaaaagatgagaggattatctttctcttaactgtaataaaatgtgtagttcatgtcatagtgcacaaatcagactcctattactagttgctcctgtgtcttcctaaatcataattgagggctatgtttttatgtgaacccagatgcccctagttcaatgccttctttttttgcttcctcagccctttctccatctatatactttttctgagatgattaaattcttttttttttttttttttttttttttttttttttttttttttttttttttttttttttttttttttttttttttttttttttaagatattaaagtctttattgcccccattttatttttcagtaatacagggatggaaatggagtgggattacttgatctggcaggtaacatggtcgggttgatatggctgcaaccttaacaatacgtaaccc

RC:

gggttacgtattgttaaggttgcagccatatcaacccgaccatgttacctgccagatcaagtaatcccactccatttccatccctgtattactgaaaaataaaatgggggcaataaagactttaatatcttaaaaaaaaaaaaaaaaaaaaaaaaaaaaaaaaaaaaaaaaaaaaaaaaaaaaaaaaaaaaaaaaaaaaaaaaaaaaaaaaaagaatttaatcatctcagaaaaagtatatagatggagaaagggctgaggaagcaaaaaaagaaggcattgaactaggggcatctgggttcacataaaaacatagccctcaattatgatttaggaagacacaggagcaactagtaataggagtctgatttgtgcactatgacatgaactacacattttattacagttaagagaaagataatcctctcatctttttttaatttttaattttagtgtttatttttaggcaccagcctttaatagatatgaggcaaatagagattacgtttctatgacataaataaattttggtaatttctagaaaagcaaaaatactgaatctttcaatccaagtcgattggatacatagcagttcaattcctctgaacacagcagttcaatttattttgttaacaaattctgcagttgcttgtttctcagcaaaagtttctccggagtagaggaaggctgtattccatcacctgggaaggtcttttcatcacctttgctcttgttatttttttttgttcagttccaaaaataatttatttcttgttgctgccaccactctgactacctccgatatcagagaaaggacccatgtcttcttctagaagacataagattttatttctcttgatatgcagaaatcagagaccatatgttattaattttaaattccaatctccactaatagtaattaatacatggtgaataaataaatagaagtcccaatgttgttgaatacacatttttgtgacactatccaatttctaataaataagaagaaataagactttactaattcattcatttcttttatgttcattttataaattccacttatagttatcttcactaatagatgagaatagaaaccataaattcattaatatgcaactgactgtgcccatttcacttggaatgcattttttgagtttacaggcagattcatatatatctaaagcctacactactaataaattaaaaaagccaggatatctactcaccaatttaggaggaactgaatatattcagtttttttttcttctatttttctttctctctctctctgcacactatacatgtaagatcttggtcagtcttattctaaagcaaagaatgagcagaagtgcctctcagacctttcctgaaaaaaatagtcatttaaatgtctggagttaaaggccttctttgtatagtatacggaaagtaattagactcaaaaccctttagacaatcaaggcaggtattaaatgcattacatatcaggagtattagagataaaaatagagccaaatcttatgatggaggaggccaacaaccttcccatagaaaaaaactacatgccagacagtagatttttgccaagacagtctcctatgtggcacagactatgaaagctatggtccttcagaggaagtataggtaatttaaccagggagtaaagatgagtgactagtggatggggaaccactggaaattaaaaaatatatatttataagacaaccaggagagctactgtcagagaaaccagctagaaagtacattcagtaagagaaaatgtgtggtaaaaaagccttgaatagactcataatcaggctggcttcatgggcctgagactcatggagtcacacacaagattccacactcagaagggccccatacttcatttaatactcttgctgtagctatctttaaattctgaataatttctaaatagaaaattcaacattttcattttatactgggccctacaaattaagtagcttgttcttgggttggtcatatatggaaattaatgaaggtgacatgaaacaagagcatgagaaaggatgaagggttgtggctgtatgtgaccagagagtaaaaaatctataggtggtaaattagaattctacattacagacaattcaggaagaataggattgaggactactctgagcaggcaatcttttgggaactattgtgttgagtattgaaggccagaggcatgtaacaggtccaaatatgcatgaaaaaatgagaagaccaagtctcaagttaagttgggttaggcctaggaaggcatgcattccaggtattgggacagagaaaaaaatgggcagcctaaaaaatcaagtcacaggacaagtggtttgtaagaggagaagaaaagaaagaaggctgaagaggcccttttcataatggtatagcataatgccaagcccaggactgcggtcattgtgtttcatgaaatttgtaagaaaatcccacctttggaaagatgatgaacagttttttagtttgcatatgtggtggtgttcacaaaaaatgtccaagatgtaattggataaaaagaatatggagctgctagaagagctcaggcacagagacagattttaacagtgagagctaacatttgttggaggctcacctttgcaccacacaccatgtcgcattcttctcttgtggtagaaaacaccactagatggtatatataacatccttttcagaggaaaaggtaatagtcttataagaggagaaggccagagaaagctagacttacaagaaacctttgctattaaaatttgggtctatatttaagccccttccttcactcactaaaaaatagccactcaagatattactactcttgtgtttaagatgttcattttagctattgcttttatgtgtgagagttgagatatcaagatgacagagaaacaagataacatgaggttactattataaacttagaaataaataaaagaaattagccccataagtttcagaaactcaacaagcttaatgacagatagattagtcaaggcatactaggggggtagtagatatttttcatttctgcacaatcaaaatgttgaacatcttcccctatgtgctagtaatgtatgaggtccaaattcttaaggtagaaaaaccagaaaattagaaatttcagcctctgcaagctaggagtgaccatgtgctctggtggggagtccctgggaagaccccattctcaaaataaaatttcaaaaaaagagaaggggctttacactttgccctttaggctattctttattctgttctgcctgaaaatcagatatattgccttcagggtctcaccaccaaactcaagaggtgcaacattctctttattatgacaaatgctctttctatgctaagtgtagaacagggaagatggaggtaacttagtcctagatggcatcccttacttccagctctgacatcacggaatggaaaaaaagaaaaggcaaacaaacaaaacaaaaatgaagctgctgttgtttgagttttctgttcttgtatcccaatgaataatttctggttaatctaatgggcataatacagaaatctgagggcaattatgtgattagttctcaagagcatccaggactccagtgaaatcaggacttgccctccatttccactttcttcttctacttttggctttatcttttagttgcagtagagaagcttttgtggttgaaaacatggcacctcacacctcccagttttatgtattagagctcctgccaccagatgacttcttgattcaggtctccaaatccagaaaaatggtttcctttgtcatggcttaggtcagatttactttcagtatcaatcaattttggccagaaggttgaagtctttaagaatttaatattcccaggaaactaagttctagtagagaactggtcaaaggaagatctttgtggagtcagcagtttctctgtcctacaatttggcaaaataatctcaataagcacaagaaggttaatatgcttgcattaaaaaatgttaagttactttatggtccaatttaaaatgtaagttcaaataccttaacatgttcctacattactttttttaattcacaaaaacgttcttatctatctttattttaagatgttttccacatacatgcttatatgtgaattttcttttaggagatgtaagagagtctacaaatctttattctactatttcccaaaatatacccaattactggcaaaaaaatgttattattaaactgatgagaaaatataaaggtttattactgattaaatgttttcatatgctgaattttttatacacacagtatatactcattatatgtatacacagaatacaactcctctcatcagtattgaacttagtttgtcattccattcaaatggccatagaaggtgctgcttgtccagggtttgtgtaacctttaaccttagcaatacgtaacgaagcacaag

chr22:29066035-29066118 polyA chr6:74978187-74978614 ctgcag chr6:74974977-74978206 chr22:29065521-29066032

ctgcag chr6:74974977-74978206 chr22:29065521-29066032 (Primer gap) chr22:29066035-29066118 polyA chr6:74978187-74978614 ctgcag

Consensus sequence for insertion at chr8:111856478 (Twin Priming)

ttaattgtgctgcttcagttacgtattgctaaggttaaaggttacacaaaccctggacaaagcagcacctcccaaatatacttaattactggcaaaaatgttattattaaactgatgagaaaatataaaggtttattactggattaaataagttttccatatgtggattttttactacacacagtatatactctattatgtgttatacatggaatacaactctatcagtgatgaacttggtttgtcattcataaaatgtcatagatggcgtggaatcaaataatcagtactcattttacctgtattactgaaaataaataaaaatggatcaaaataaagacttaatatctttaaaaaaaaaaaaaaaaaaaaaaaaaaaaaaaaaaaaaaaaaaaaaaaaaaaaaaaaaaaaaaagaaaagggtcttcaactcttgttctgtggtgtgttttttccatttatttaaattaaaacatatattgtataataaaagaaaggtaataaatttatatttcagttttttaaaattttaagttacttaatgacactgcttactgtaaatacagagctcacatacatatgagagaacattattttggaaagatgaaagcattacattgtgatataatattaactacacaagtagttataaatattggtttatatattaaaaataaaaattgtgctatttctaagattgtcagtcataaaatgttctagttgatatcttgttaagacatctaaattattccatagcgacattaataggtactattattccacaactaacattaaactttttcagaaatacaataaattttttaataatgtaatttacataacagaatagctaagatactaaattccctctggtctatgagtctttacacattcaaaactgcatcttagaagaagaaagaaagttctaattttttgcatgtctcttctggtatatgtttgaaaaattactctttcaaatgtcaaatattggagttgacatggtgactcagtactgtaatcccagctactctggaggctgaggcaggaaaatctcttgagaccatgatggacaacatgggaagaccttgtatatacaagcttaaaaattagtgaggcatggtggcacatacctgtagcccccactactcaggagactgaggtgattggaccacttgcactcagaaagttcgaggctgcactgagctgttatcaaactactgcattccaacctggatgacagtgagacatcatctctaaataaatacaaaaagacctaaatatcaaatgttataaatacagccttgtttagtaaaatagaacagcctattttatttcaagttttttatatgtttttactactcttgcacatcaaataatgtgatttattattggctcttgttgtacgaacatgtagcattatcagccaagatctgcacacgttatgtttattggttgctcctcagtgtctaccttagagtctacctgacatatattcttctgacatgacagaataaataaatatttaaggtattcttttctacttgtttgaacattaatttagaattgattcttagagatgaaattcatctattttaatggtttggggaaccatatgtataacactggcagcagataattattgctgagaaatctaaaaaagaagaaaataggtcattaaattaatgaaattaaaggtgaaaaaaaaatagtaagtaaccaagaaaaattatattttatgttggcatctgaagtgtagtaatttaataatgaagtaatatcccaaaatgttcaatattttgattcaaacatctttaaaacaggcttctatattccgttttttttatatttgtttgttgctagcttgaaatgtatctaccttatttgatctcaacatggctgtcagattctacactaattgctctagttttgaattccttctcccattttatcctcagcactgctgtcaggagtaatgtttttatttatttaagaaaaatttttttagaaacgtagtctcactatgtttcccagcctgaatcttaaacttctgacctcaaatcatcctccccctttcagcccgtctggttgctggagttacaagtgtgagccaccacactcagctaaagtattattttttaaaacctaatttggacaagtcacaaattagaggctcaatgttttttttcttttttaaaattacaattaaaatgccaggatagattgtgtctttatattaaatcagttataaaatacacttaaaaaacttaattcagtatattcaagaatgtgtcaagtaacagcaaccaaagttgtcactaagaaattggtttcgcttacctacaatattagtaatacctaaaatgctaaatggggtatactcttgcaaaggaactgtataaaaggtatcaatggaatacaaggaaaatggcccaagctgttatcccaagtactgtgaaaaatcactcaccatccttacatcttgtgaaaaattgtccaaggatacatttttttgtagtctcctctacccactgatttcacagtatcaattcatagtgatgcactctttggctgtttatttcactgtgtctaatcttgcttcacaaacagagcacaatcctttattctactttctactagaatactcttagtatatgactgctatagatttattttaattctttattctttgaactttaggacaaaattggttatctctcctgatcattgctgggtaacttcttaaaaagtgacttctacaaatattttagtgtaaaacctgaaagtttatgattaatttattaaactctaccactcctaatatgtgttatgtccaaattaggctttaaaaatattatccatagctgagtgtggtggctcaattgtaattcagcaactctaataaattgaaagtacagctttcagcattttattatttttgaatataagataatctggctttaaaaaattatttatccttgcttttgcattttctgaatatcttcattgacttgtctctggtaaaaatttcaggagtaacactcacttgaaaatttaataatgatgacttatataatgttcacaaatctcattactatcatgttgacaaattctagtgatcaaactgttattcacaaaagcccgtatttttaagtgtgcttcttgatattcagctaaagtgaagaaatgcattttcataatgcagtttctgccttgcctaccatttattccaaattgcttggcttaatactaacacttttccaaatatggtaacatttacttacccaaactctatctcactctctctaataatatgttaatctaaaagaaagttttcacttctaactgaaaaaacaataaaatttctatgtttggaaatttgtttgtgataacattcttatcaagagatagctttttctaaattacctaattcattctagtaaataattttctttgtttctatcatatttagtatcagtatcataaaaatgatatatcacttttatgtttcattaatgttgtctcaaatttacctttttatagcacactatattttattttttattttttatttatttattttttttttgagatggagtctcacactgtctcctgggccgagtgcaatggcatgatctcagctcactgcagcctctgcctcccaggttcaagcaattcttctgcctcagcctcccaagtagttgggattacgggcaccactaccacgcccggctaatcttttgtattttagtagagacagggtttcattatgttggccagacttgtctggaactcctgaccttgtgatccgcccactcagcctcccaaagtgcaggattacaggtgtagaccctgcgcccacaccacactatgtttagaatgggagagcacaataaatacctattgagtctatggattaatgtaaaattaattacttaataaatggcaggtgagccatgaataaacaatataataatttgcgattactcaaacataatttcagtgatattattaaaaagatactaataatttgaatatatatattttttgacccacttacttaagactaatgatcaaatcactccccaaaagaaactatattaaacatcgtagtagctgctcagaaatgtgaacacttcaataatagacaataaaatagtcatatataatattagaattatggtcaatttctttggttatgaagttcagaaaaattacaagatattactacattttaaaatttaaaatatactatttttttgttattgttttacttattttgtgacttatgataattgtaattccttctaatagatttggcttatagtatttttaagtattctatttagctctattacagatatcttaagatttgcatttaacatgtagaaatacacattgatatatttattttttatattaatatattattttcttaagtttttacaattataaaacataatacccaagcataatctgaaaatatcacatcagaatgaagagaaacagaaaaaatttcatcaataaaattaaattcaataattattattgagatcaaattcatcaatattgcctctttgcttatttcaacctcactttatatcacaaaatatgttattcaatataccttatacataggtcacttttaattaaatttttatggccttttacaaccacttggagtagaactatctgaattttttaggcatatatgtttatgtttgtataaaaaaggtaaatgtatgtacatttgtatagagtttgtatttttatttcacgtaaatttttgttataaaatgtgaaattaacaaattaaatctaaatgtatgctccaacgttcatcatttggtaataggtctttgaaactatcagatattgctgaagtacataatatactatcctaacacaatgaagctgcttatctggaccaagattatcagacataaatatatttgacaagccaatgaaacaagttcaaatttcagctgtgtttcttttttatttctctctcttttcttccttctatttatctttttattcctctaaacaaactgtaaaatctatcttgacctctttatctctcatctatcattgctacataatcagacaaaattatttgtttggtaaaattcataatgtattcttcatgtttacacaatcaaaatttttgagacaagggtcttcaactctagttctaggatgaggaatcgccacactgacttccacaatggttgaactggtttgagtcccaccaacagtgtaaaatgtgttctatttctccgcatcctctccagcacctgttgtttcctgacttttttaatgattgccattctaactggtgtgagatgatatctccatagtggtttttgatttgcatttctctgatggccagtgatgatgagcatttcttcatgtgtttttttggctgcataaatgtcttcttttgagaagtgtctgttcatgtccttcgcccactttttttgatgggttgtttgtttttttcttgtaaatttgttgagagttcattgtagattctggatattagccctttgtcagatggagtaggttgcgaaaattttctcccatgttgtaggttgcctgttcactctgatggtagtttcttttgctgtgcagaagctctttagtttaattagatcccatttgtcaattttgtctttctttttttcaattcacaaaaacgttcttatctatctttattttaagatgttttccacatacattagtgaaaggtgctgcttgtca

RC:

tgacaagcagcacctttcactaatgtatgtggaaaacatcttaaaataaagatagataagaacgtttttgtgaattgaaaaaaagaaagacaaaattgacaaatgggatctaattaaactaaagagcttctgcacagcaaaagaaactaccatcagagtgaacaggcaacctacaacatgggagaaaattttcgcaacctactccatctgacaaagggctaatatccagaatctacaatgaactctcaacaaatttacaagaaaaaaacaaacaacccatcaaaaaaagtgggcgaaggacatgaacagacacttctcaaaagaagacatttatgcagccaaaaaaacacatgaagaaatgctcatcatcactggccatcagagaaatgcaaatcaaaaaccactatggagatatcatctcacaccagttagaatggcaatcattaaaaaagtcaggaaacaacaggtgctggagaggatgcggagaaatagaacacattttacactgttggtgggactcaaaccagttcaaccattgtggaagtcagtgtggcgattcctcatcctagaactagagttgaagacccttgtctcaaaaattttgattgtgtaaacatgaagaatacattatgaattttaccaaacaaataattttgtctgattatgtagcaatgatagatgagagataaagaggtcaagatagattttacagtttgtttagaggaataaaaagataaatagaaggaagaaaagagagagaaataaaaaagaaacacagctgaaatttgaacttgtttcattggcttgtcaaatatatttatgtctgataatcttggtccagataagcagcttcattgtgttaggatagtatattatgtacttcagcaatatctgatagtttcaaagacctattaccaaatgatgaacgttggagcatacatttagatttaatttgttaatttcacattttataacaaaaatttacgtgaaataaaaatacaaactctatacaaatgtacatacatttaccttttttatacaaacataaacatatatgcctaaaaaattcagatagttctactccaagtggttgtaaaaggccataaaaatttaattaaaagtgacctatgtataaggtatattgaataacatattttgtgatataaagtgaggttgaaataagcaaagaggcaatattgatgaatttgatctcaataataattattgaatttaattttattgatgaaattttttctgtttctcttcattctgatgtgatattttcagattatgcttgggtattatgttttataattgtaaaaacttaagaaaataatatattaatataaaaaataaatatatcaatgtgtatttctacatgttaaatgcaaatcttaagatatctgtaatagagctaaatagaatacttaaaaatactataagccaaatctattagaaggaattacaattatcataagtcacaaaataagtaaaacaataacaaaaaaatagtatattttaaattttaaaatgtagtaatatcttgtaatttttctgaacttcataaccaaagaaattgaccataattctaatattatatatgactattttattgtctattattgaagtgttcacatttctgagcagctactacgatgtttaatatagtttcttttggggagtgatttgatcattagtcttaagtaagtgggtcaaaaaatatatatattcaaattattagtatctttttaataatatcactgaaattatgtttgagtaatcgcaaattattatattgtttattcatggctcacctgccatttattaagtaattaattttacattaatccatagactcaataggtatttattgtgctctcccattctaaacatagtgtggtgtgggcgcagggtctacacctgtaatcctgcactttgggaggctgagtgggcggatcacaaggtcaggagttccagacaagtctggccaacataatgaaaccctgtctctactaaaatacaaaagattagccgggcgtggtagtggtgcccgtaatcccaactacttgggaggctgaggcagaagaattgcttgaacctgggaggcagaggctgcagtgagctgagatcatgccattgcactcggcccaggagacagtgtgagactccatctcaaaaaaaaaataaataaataaaaaataaaaaataaaatatagtgtgctataaaaaggtaaatttgagacaacattaatgaaacataaaagtgatatatcatttttatgatactgatactaaatatgatagaaacaaagaaaattatttactagaatgaattaggtaatttagaaaaagctatctcttgataagaatgttatcacaaacaaatttccaaacatagaaattttattgttttttcagttagaagtgaaaactttcttttagattaacatattattagagagagtgagatagagtttgggtaagtaaatgttaccatatttggaaaagtgttagtattaagccaagcaatttggaataaatggtaggcaaggcagaaactgcattatgaaaatgcatttcttcactttagctgaatatcaagaagcacacttaaaaatacgggcttttgtgaataacagtttgatcactagaatttgtcaacatgatagtaatgagatttgtgaacattatataagtcatcattattaaattttcaagtgagtgttactcctgaaatttttaccagagacaagtcaatgaagatattcagaaaatgcaaaagcaaggataaataattttttaaagccagattatcttatattcaaaaataataaaatgctgaaagctgtactttcaatttattagagttgctgaattacaattgagccaccacactcagctatggataatatttttaaagcctaatttggacataacacatattaggagtggtagagtttaataaattaatcataaactttcaggttttacactaaaatatttgtagaagtcactttttaagaagttacccagcaatgatcaggagagataaccaattttgtcctaaagttcaaagaataaagaattaaaataaatctatagcagtcatatactaagagtattctagtagaaagtagaataaaggattgtgctctgtttgtgaagcaagattagacacagtgaaataaacagccaaagagtgcatcactatgaattgatactgtgaaatcagtgggtagaggagactacaaaaaaatgtatccttggacaatttttcacaagatgtaaggatggtgagtgatttttcacagtacttgggataacagcttgggccattttccttgtattccattgataccttttatacagttcctttgcaagagtataccccatttagcattttaggtattactaatattgtaggtaagcgaaaccaatttcttagtgacaactttggttgctgttacttgacacattcttgaatatactgaattaagttttttaagtgtattttataactgatttaatataaagacacaatctatcctggcattttaattgtaattttaaaaaagaaaaaaaacattgagcctctaatttgtgacttgtccaaattaggttttaaaaaataatactttagctgagtgtggtggctcacacttgtaactccagcaaccagacgggctgaaagggggaggatgatttgaggtcagaagtttaagattcaggctgggaaacatagtgagactacgtttctaaaaaaatttttcttaaataaataaaaacattactcctgacagcagtgctgaggataaaatgggagaaggaattcaaaactagagcaattagtgtagaatctgacagccatgttgagatcaaataaggtagatacatttcaagctagcaacaaacaaatataaaaaaaacggaatatagaagcctgttttaaagatgtttgaatcaaaatattgaacattttgggatattacttcattattaaattactacacttcagatgccaacataaaatataatttttcttggttacttactatttttttttcacctttaatttcattaatttaatgacctattttcttcttttttagatttctcagcaataattatctgctgccagtgttatacatatggttccccaaaccattaaaatagatgaatttcatctctaagaatcaattctaaattaatgttcaaacaagtagaaaagaataccttaaatatttatttattctgtcatgtcagaagaatatatgtcaggtagactctaaggtagacactgaggagcaaccaataaacataacgtgtgcagatcttggctgataatgctacatgttcgtacaacaagagccaataataaatcacattatttgatgtgcaagagtagtaaaaacatataaaaaacttgaaataaaataggctgttctattttactaaacaaggctgtatttataacatttgatatttaggtctttttgtatttatttagagatgatgtctcactgtcatccaggttggaatgcagtagtttgataacagctcagtgcagcctcgaactttctgagtgcaagtggtccaatcacctcagtctcctgagtagtgggggctacaggtatgtgccaccatgcctcactaatttttaagcttgtatatacaaggtcttcccatgttgtccatcatggtctcaagagattttcctgcctcagcctccagagtagctgggattacagtactgagtcaccatgtcaactccaatatttgacatttgaaagagtaatttttcaaacatataccagaagagacatgcaaaaaattagaactttctttcttcttctaagatgcagttttgaatgtgtaaagactcatagaccagagggaatttagtatcttagctattctgttatgtaaattacattattaaaaaatttattgtatttctgaaaaagtttaatgttagttgtggaataatagtacctattaatgtcgctatggaataatttagatgtcttaacaagatatcaactagaacattttatgactgacaatcttagaaatagcacaatttttatttttaatatataaaccaatatttataactacttgtgtagttaatattatatcacaatgtaatgctttcatctttccaaaataatgttctctcatatgtatgtgagctctgtatttacagtaagcagtgtcattaagtaacttaaaattttaaaaaactgaaatataaatttattacctttcttttattatacaatatatgttttaatttaaataaatggaaaaaacacaccacagaacaagagttgaagacccttttctttttttttttttttttttttttttttttttttttttttttttttttttttttttttttttttaaagatattaagtctttattttgatccatttttatttattttcagtaatacaggtaaaatgagtactgattatttgattccacgccatctatgacattttatgaatgacaaaccaagttcatcactgatagagttgtattccatgtataacacataatagagtatatactgtgtgtagtaaaaaatccacatatggaaaacttatttaatccagtaataaacctttatattttctcatcagtttaataataacatttttgccagtaattaagtatatttgggaggtgctgctttgtccagggtttgtgtaacctttaaccttagcaatacgtaactgaagcagcacaattaa

chr22: 29065782-29065713(-) chr22:29064270-29064757 chr8:111856457-111858464 atgcat chr8:111853706-111856478 polyT chr22: 29066083-29065832(-)

atgcat chr8:111853706-111856478 polyT chr22: 29066083-29065832(-) (Primer gap) chr22: 29065782-29065713(-) chr22:29064270-29064757 chr8:111856457-111858464 atgcat

Consensus sequence for insertion at chr14:99070525

ctgcattgtgcttcgttcagttacgtattactaaggttaagactactttctgcctttgcgagaacagcacctttcactaagcatgtatgtggaaaacatcttaaaataagatatagataagaacgtttttgtgaattaaaaaagtaaatgtgaacatgttaaggtatttgaacttacatttaaattggattataagtaacttaacattttttaatgcaaacatattaaccttcttgtgcttatgagattattttgccaaattgtaggacagaaactgctgactccacaagatcttcaatcaacataggatgtacttcaactgatcaaaatggttttgaagaattacttgctatctaagggactttgttacttacaaaataacttttctaaagatctgtttggccagctatagatagaaagtacgcagaaaggagagctttgcttctaaaggttgaacttcaaaagttcagacatatttcctatcaacatggtttccaacaaacttttcaaacatgaaggccgtgacttggcactgaggtccagagagaacttcttctgagatggcttcagtgcattagagtgctggtcatgtgtggggcaggtgggggaaactaaatttaggagtctaagtggcaaatgcacaggcaagaacttcaccaggggtgagccaagtgggaagggatgccagttagaggagcattacaatggcgaggttcctagaaattaacaagacacctgtgcctgttttgagacattctccttagcagtgactctttgcttctagacttagactcaggtctttctcagctgtagggatatctcatcttcagtgattaataagctgacttcttgaacaacctagcaacacagcatgcactaagactcaggaagcagtactagatcaggagcatccctggaggatgccttctctgcccccaccccagttttctgggtgcctgcctctatggaagggatggtactcatggccagaactccaagtgtgacacagatgtctcctacatcctggagcagagtagggcagaatttgggcttactacatagataacctatctttttttttctgagcctcggtttccttgtctgtgaaacaggaataataacaacttctggagagggctctaatgagtaacagatgagaagaaatgtaggaagtagactataaattataaaaaacactactgagatgcaaattatcatgattatttataaggacccaagggttgtcccaagacgtgtttttccgcatctcagttcttccatctcttctttgggacacctcatctcccctaaatgctccctttgctgggatacagcctcccctgctggtctctaaattagcccatcctctaacacttctccttgagaatttcaattcctttaatccagtgaaataaagaagttcaggccatcatcttcaaagaataagaataaaatttgaggacaaaaattatacccattgggactgcttctgtagaaatactttggaattccttttcatgccctattaaataattaaaatgtttcttttctgcattcaatgttagttatttaaatcttagtcctaagctccattagcaactttattagcacaaaactttctttgccagttggattagttttatagattttttgcatagaaggagtgtacgcagacaaatacaagtgcgtggctatggttaacatcctacaatcctaggaaataggttgaatttaagctgacctgaccaatctcactccacttaatagcttattggcttaatcggtgtcttccatttgtgtcaggagctgttagcacatcaaaatcttttggtagattccagcagatgaaagtcacacctcacctcccaccccatcccagaatggatgctcaatgttaatgacttgagaatttcatagttaggcagaaaaacagagtcacaaatcttggggacagaagataatcatatacggcaataaagccacaagggccaagcaataaacctgattttggccattccttcagcctccctcctaggccatattcactcaaacttcacacctaacacactccctctaagataaacctggagaccgaggggagttgttagtgaaagaatctagtgtcaagatcagcatcaggtaagagaaagcaacagagagctcgtgacgcccaccctccagagccagttaggatgagggagactggctgtgcatgtccaattcctgtcatggaggccaggacaaaccacgaaccatgagggccccccaaggctgggaggcagggattgtctggaggctgctgcagagaggccatggacacaggcttcgggtggtcatccacaggcagtggattttaatctcttttttaaaacaacctagaaggaatttaatggttagccaacctgaatgtcattcagcaccagtaatgtgttggtgccaaataatcaacatcaccctgaagagttaccagaatgatgataaggaattccatgtacagagtcagagcctcatgggaggacacctgtcataagccttgggctggtgcttatttcttggcacaaagcattcaccatacatttcaaaacggaaaacaatcttttttgaagttctacacttcagaaagtaatgtgaatgtgactcaggtttaagatattactaaattccatgtaatcatcattatcctgtgtataaatatacacagaccacaaaataattttcacattgaaatgggttttaaggaacgtttcaggtaaaatgacataatcttcacattttggagcctatgaccttctgtgtgtttgattttccaattccaaacctctcttttggtgaattgctaaattgtctcggtaaacggattttaaaacctagctttgaaaactaccagctaagtcatctcaaatatctgtttatacctacacatatatttagcgggtctttttcctcctaaactcaaccctgtttaaatggaggtcagactgattgacatggaggttactgagtttactcacctctaagtaagatgtgatggtaacagattggatgaacacattttaagattccttgctgaatactgttttttgcggcacagaagaaatagcagcgatgggtgtacaataagaacaggtattgcataaattattccaacattcaagattcaacagaaacaaaatgcattttttcaagcactttaaacttcataaacttggtgtacattcaaaacattcttttttttttttttttttttttttttttttttttttttttttttttttttttttttttttttttttttttttttaatcagtaataaacctttatattttctcatcagtttaataataacattttttgccagtaattaggtatatattttgggaggtgctgttctcataaaggcagaaaaatagtcttaaccttagcaatgcgtaactata

chr22: 29065782-29065558(-)chr14:99070523-99072542 ctgcag chr14:99069584-99070525 polyT chr22: 29065912-29065832(-)

ctgcag chr14:99069584-99070525 polyT chr22: 29065912-29065832(-) (Primer gap) chr22: 29065782-29065558(-)chr14:99070523-99072542 ctgcag

Consensus sequence for insertion at chr16:26220799

acattgtattcgttcagttacgtattgctaggttagactactttctgcctttgcgagaacagcaccttctatggccattttatggaatgacaaactaagttcaatactgatagagttgtattctcaataatacaatacattgctaataattatagtcaccatgtggtacaatagatcttttgaacttattcctcccatctaactgaaattgtgcatttttttaaccaacatctacccagccccccaaccccacctctttcccagtctctggtagtcactattctacccactacttctgttagtgcaaatttttttttaattccatcgtcagtaagatcacatcatatttgtctttcatgcctggctttttttttcacttagtataatgtcctctaggttcatccatgttgtcacagataagagaaattttcttctattttagggtgaatggtatttaattgtgcatatataccacattttaaaaacctgttcgtctgttgatggacacaggttgatttcacattttggctattgtgaatagtgctgcaataaacattggagtgcagatatctctttgaaatactgatttcatttcctttggatatatacccagcagtgggattgctggttcatgtgaaatagttctattttaatttttttgagaacctccatactattttccttaatggctgttttcttaattttcttaatttacatttacaccaacagtacaccaggtctctgttttccccatatctctgcaatacttagctttcatcttttttgacactagctatcttaaaggtgtgaggtgatatttcattctggtttcaattttcattgctctagtgattagtgaggctgaacattttgatatacctgttggatatttgtatgtcttcctttgagaaatatttattcaaatcctttatccattttttaactgggttatgtattttcttactgttgagttgagctcgtacctggtgctttgccaaggtgtgccagaccgaatcagggctcactgcatgcctcagattatgccctatgctcatggctgtggggccagcctcccccgatcacataaatcactatgcggcaatctggctcccaagctggggagcctctgaaaactcacaacactttgcagagaagccgccagaattaggcataaatccacttcttgattggataagcaaacactccactgcatgaaatgatttactaaattacaatgcacagtgacagacttgggagattcagagcaagggcctgcttagcaattggaggcattctctaactccatggcttcctccacctctgcccttcctggcttcacctgtgacttctttccattcattcactcatttaatcatcattcattgggcacccacagcatgacaggtaaggtgcttacacataaattatctcgttaactttcatctatgcaacaactcgaaagtcagtatcacttgattttttctgttttgcagatgagggaactgaggatcaggaggttaagaaactccatatcacacagatagtaagtggtagagttgagattaaactccaaaaacatccatcaagccccaaaatttgtctactgtagactaaattcactgttgctgttttctttttgtctacaaatatctaactcttccacttcttctttttttttgcattttgttttcttttacttgtatctgtcagcatagggtaggttatgctgagggtgacataccccaacattctatggcaatgcagcagaagttgttgttattttttttttctagtgctttctcaacctaaatgtttcatgcagtccagctcagcatctccactatacatagtcacttaggctgacagactcagtcacttaggctgacagactgctctcctgcagtgctccgtgtggaactcgggggccatcttagtttctgcaagaggaaaagaaagtagaaagtttcacactcacagttaaatgccttcaactgaaaaggaacacaaactatttgacataacctgattatgtaattccacctgcccaaagcaggattaagtacaatcatccatgtgtccaaaggaaatgataatgttttattgtgaacattatacaacaccctcattctcctctttgtactcattcattcagcagatagttactgaacttatgcaatatgccagggcattcactgggctgaacattggcaataaagaggtgaagcaaaacacgtgcaacgtctgcactcaagaagctgacgttatgattagaacacgatgtattaatcaagtgaacgagcatgcaaataaactagaattgtgtcaagttctgtaaaaaaacaagcaaatggttttatgaaaacccataacaaaggtatgaactaaactaggaatcagagaaagcttttgtttcagtaaagcttttgattacaagcaacagaatccatctctagccaaattaaatagacagggaatttaattagaggatactgggtaactcacagactgaaatttacacagtctctgttctttccatttgaaatctggggcagtgtatttgtttggtggagttgtgtggtctcctttgtagctgcaagaagttctgaaaacaaaagtatctggccatctcagtttcaggtagcagaaagaatgacatacgtcctttgaagctactctctgagaagaaaaaaacagtggagttgagatttaaagaaataacagaagtagcaaaaggaataggaaggaggaatatatactttcctccacccctcatttataaaaattcaaatatttacaaaagtagaaaaataacatcataaaaccttgtgtacccatcacttttgcttcaactccatctgattttgtttacaccctcacccacttttctctgttgctacattttttttagaatacgtttttttaaagttctcagacattatgtaaatagtttatttgtgtatgattatgaatgataagaacttatttagaaaagaaatagaatcttgatgcaattatcacacttaaaaaaataacaataattatttgatatcaccaaatgcccagttagtacttacattttactgatggtcttaaaattgttacacaatttgattgagcagaactcaaatgaaatccatatattgtgtttgacttatatgtctcttgtctcctttaatagataggtcacccctgtctatgttcttccaacagacagataagaaggcatggatatggccagatgctgtggttcacacctataatcccagcactttgggaggcagaggcagaggcaggtggatcacgaggatgggagatcaagatcatcctggccaacatggtgaaaccccgcctctactaaaatataaaaaattagctaggcgtggtggcgagcgcctgtaatcccagctactcgggaggctgaggcaggagaatcgcttgaaccagggagtcagaggttgctgtgaggttgagatcgcagtactgcactacagcctgggtgacagaatgagactccgtctcaaaaaaaaaaaaaaaaaaagaaggcatggataccaagttagacatgaacatagatatatagatatagacataaatatagatacagagactgatatgacatagacataaacatgaacatagaagcagaaatagacatagagatagatataaacataaatatagacataggcatagacatgaacataatcatagttgcaggcatagacataaatataggcagctggagaaaaaaaactgagtcatttcttctagtgtcccacagtctgaattttgccagttgttttttttccactttttgtattttctgtggattaatgatagatccagagacttgatcagatgctacttcaaattttttggtaagacgatcatgggagatgcatacttccattagaaggtgtgcaagttctgactgtctccattttgtgataatagcacccactgctgatcattgactagctctattcattagaagtcgcaaaaaatagtgatatttgcattttattttttcctctttcattagctgggatacacaattaatatggaagagtcaggataaatcttaattctcccctttacttactaattttcaaacaatgagctggtttcctaatagcatttcctgacagtaaacaatgaatggttacttttttttagaatcatcatgaacttatgaatttaaacatacttatattttaatctactgcaggtagtattctgattgactctaattgtcctattttgggccagtggtgtcttattcatgttggtttcacatttttaacattttaaaaaattctaattgacaataattgtatgcatttattgtatacatttttttattacaaatgttgagatattaatactctatgagtgctaaattatatgcattgcttcacatacttactatttcttgaggtagaacatttacaatctactctcttgacaattttcttttttttttttttttttttttttttttttttttttttttttttaagatattaaagtctttatttgcccccccatttttttcagtaatacacagggtaaaatggagtaggattacttgatctggcgggttcagttctcgcaaaggcagaagtagtcttaaaaatacgtaacccc

RC:

ggggttacgtatttttaagactacttctgcctttgcgagaactgaacccgccagatcaagtaatcctactccattttaccctgtgtattactgaaaaaaatgggggggcaaataaagactttaatatcttaaaaaaaaaaaaaaaaaaaaaaaaaaaaaaaaaaaaaaaaaaaagaaaattgtcaagagagtagattgtaaatgttctacctcaagaaatagtaagtatgtgaagcaatgcatataatttagcactcatagagtattaatatctcaacatttgtaataaaaaaatgtatacaataaatgcatacaattattgtcaattagaattttttaaaatgttaaaaatgtgaaaccaacatgaataagacaccactggcccaaaataggacaattagagtcaatcagaatactacctgcagtagattaaaatataagtatgtttaaattcataagttcatgatgattctaaaaaaaagtaaccattcattgtttactgtcaggaaatgctattaggaaaccagctcattgtttgaaaattagtaagtaaaggggagaattaagatttatcctgactcttccatattaattgtgtatcccagctaatgaaagaggaaaaaataaaatgcaaatatcactattttttgcgacttctaatgaatagagctagtcaatgatcagcagtgggtgctattatcacaaaatggagacagtcagaacttgcacaccttctaatggaagtatgcatctcccatgatcgtcttaccaaaaaatttgaagtagcatctgatcaagtctctggatctatcattaatccacagaaaatacaaaaagtggaaaaaaaacaactggcaaaattcagactgtgggacactagaagaaatgactcagttttttttctccagctgcctatatttatgtctatgcctgcaactatgattatgttcatgtctatgcctatgtctatatttatgtttatatctatctctatgtctatttctgcttctatgttcatgtttatgtctatgtcatatcagtctctgtatctatatttatgtctatatctatatatctatgttcatgtctaacttggtatccatgccttctttttttttttttttttttgagacggagtctcattctgtcacccaggctgtagtgcagtactgcgatctcaacctcacagcaacctctgactccctggttcaagcgattctcctgcctcagcctcccgagtagctgggattacaggcgctcgccaccacgcctagctaattttttatattttagtagaggcggggtttcaccatgttggccaggatgatcttgatctcccatcctcgtgatccacctgcctctgcctctgcctcccaaagtgctgggattataggtgtgaaccacagcatctggccatatccatgccttcttatctgtctgttggaagaacatagacaggggtgacctatctattaaaggagacaagagacatataagtcaaacacaatatatggatttcatttgagttctgctcaatcaaattgtgtaacaattttaagaccatcagtaaaatgtaagtactaactgggcatttggtgatatcaaataattattgttatttttttaagtgtgataattgcatcaagattctatttcttttctaaataagttcttatcattcataatcatacacaaataaactatttacataatgtctgagaactttaaaaaaacgtattctaaaaaaaatgtagcaacagagaaaagtgggtgagggtgtaaacaaaatcagatggagttgaagcaaaagtgatgggtacacaaggttttatgatgttatttttctacttttgtaaatatttgaatttttataaatgaggggtggaggaaagtatatattcctccttcctattccttttgctacttctgttatttctttaaatctcaactccactgtttttttcttctcagagagtagcttcaaaggacgtatgtcattctttctgctacctgaaactgagatggccagatacttttgttttcagaacttcttgcagctacaaaggagaccacacaactccaccaaacaaatacactgccccagatttcaaatggaaagaacagagactgtgtaaatttcagtctgtgagttacccagtatcctctaattaaattccctgtctatttaatttggctagagatggattctgttgcttgtaatcaaaagctttactgaaacaaaagctttctctgattcctagtttagttcatacctttgttatgggttttcataaaaccatttgcttgtttttttacagaacttgacacaattctagtttatttgcatgctcgttcacttgattaatacatcgtgttctaatcataacgtcagcttcttgagtgcagacgttgcacgtgttttgcttcacctctttattgccaatgttcagcccagtgaatgccctggcatattgcataagttcagtaactatctgctgaatgaatgagtacaaagaggagaatgagggtgttgtataatgttcacaataaaacattatcatttcctttggacacatggatgattgtacttaatcctgctttgggcaggtggaattacataatcaggttatgtcaaatagtttgtgttccttttcagttgaaggcatttaactgtgagtgtgaaactttctactttcttttcctcttgcagaaactaagatggcccccgagttccacacggagcactgcaggagagcagtctgtcagcctaagtgactgagtctgtcagcctaagtgactatgtatagtggagatgctgagctggactgcatgaaacatttaggttgagaaagcactagaaaaaaaaaataacaacaacttctgctgcattgccatagaatgttggggtatgtcaccctcagcataacctaccctatgctgacagatacaagtaaaagaaaacaaaatgcaaaaaaaaagaagaagtggaagagttagatatttgtagacaaaaagaaaacagcaacagtgaatttagtctacagtagacaaattttggggcttgatggatgtttttggagtttaatctcaactctaccacttactatctgtgtgatatggagtttcttaacctcctgatcctcagttccctcatctgcaaaacagaaaaaatcaagtgatactgactttcgagttgttgcatagatgaaagttaacgagataatttatgtgtaagcaccttacctgtcatgctgtgggtgcccaatgaatgatgattaaatgagtgaatgaatggaaagaagtcacaggtgaagccaggaagggcagaggtggaggaagccatggagttagagaatgcctccaattgctaagcaggcccttgctctgaatctcccaagtctgtcactgtgcattgtaatttagtaaatcatttcatgcagtggagtgtttgcttatccaatcaagaagtggatttatgcctaattctggcggcttctctgcaaagtgttgtgagttttcagaggctccccagcttgggagccagattgccgcatagtgatttatgtgatcgggggaggctggccccacagccatgagcatagggcataatctgaggcatgcagtgagccctgattcggtctggcacaccttggcaaagcaccaggtacgagctcaactcaacagtaagaaaatacataacccagttaaaaaatggataaaggatttgaataaatatttctcaaaggaagacatacaaatatccaacaggtatatcaaaatgttcagcctcactaatcactagagcaatgaaaattgaaaccagaatgaaatatcacctcacacctttaagatagctagtgtcaaaaaagatgaaagctaagtattgcagagatatggggaaaacagagacctggtgtactgttggtgtaaatgtaaattaagaaaattaagaaaacagccattaaggaaaatagtatggaggttctcaaaaaaattaaaatagaactatttcacatgaaccagcaatcccactgctgggtatatatccaaaggaaatgaaatcagtatttcaaagagatatctgcactccaatgtttattgcagcactattcacaatagccaaaatgtgaaatcaacctgtgtccatcaacagacgaacaggtttttaaaatgtggtatatatgcacaattaaataccattcaccctaaaatagaagaaaatttctcttatctgtgacaacatggatgaacctagaggacattatactaagtgaaaaaaaaagccaggcatgaaagacaaatatgatgtgatcttactgacgatggaattaaaaaaaaatttgcactaacagaagtagtgggtagaatagtgactaccagagactgggaaagaggtggggttggggggctgggtagatgttggttaaaaaaatgcacaatttcagttagatgggaggaataagttcaaaagatctattgtaccacatggtgactataattattagcaatgtattgtattattgagaatacaactctatcagtattgaacttagtttgtcattccataaaatggccatagaaggtgctgttctcgcaaaggcagaaagtagtctaacctagcaatacgtaactgaacgaatacaatgt

chr22:29066035-29066118 polyA chr16:26220798-26224282 gagctc chr16:26219944-26220799 chr22:29065976-29066032

gagctc chr16:26219944-26220799 chr22:29065976-29066032 (Primer gap) chr22:29066035-29066118 polyA chr16:26220798-26224282 gagctc

Consensus sequence for insertion at chr16:5902138

ttgttgtacttcgttcagttacgtattgctaaggttaaaggttacacaaaccctggacaaacagcacctcaaaatatacccaattactggcaaaaatgttattaaactgatgaaaatataaaggtttattactggattaaaaaaaaaaaaaaaaaaaaaaaaaaaaaaaaaaaaaaaaaaaaaaaaaaaaaaaaagggaaaacccggttaactttactaaggatcattctgatcctctgcaggctgttctcagtacttttaacatataatgccaattgatccttatcacaccccatgatgtagatcctctcattgtccccgattttaaaacagagaggaaacagcctaactcatatggctcgtaagtggtgggctggagcttttaattcaggtgcctctaatgatggagcctctgctctcagccaatgttgtagggttcagaaactctctacacctggccaagacatcgtccttccttactcacccactgactcagcaacagacagttcctttaagacactcacccacagagacgctaaacttgcggttttcctgtcacctttgaacggacatgaacaatgactcttttttaaaaggacacacagtatttacagaccaaaatgaaaaggacagcaggaaggaatgctaagaacctttgatagcacagagatgacataatttatgtttcgccatagccatctctgctctcccattgcaatctcaaattgagaagaaacttggtaattgcttcagctgggctgggcctgctgttgaccgtttcttttttcttattacctcgtcctgcccagtgaccctgagcatttcatcttcttcggcagggctggtaaatcttaggccgtccctggcagtgcagagaatgccttggaaaacacacagaggaaaattaaggaggagggatgcagagaagaggcggtctagggaaaaggtcttcagacaaaatgcaagatgacggtgaaatgaacctccagtgtggcctttgcaggataaacgaaaagcacccactgcacactgggcagagcatcttgtctccagataggacgtgatcagtaacaccaagctgaatgcacactgatatccttcctgctctgtgtgaatacaaggtacaaccatgataataactagcgggaggttactaccgtcatagggtctggcttcacaacccagaattttgggctgattgtgagggagtaagtccacagcacagtgtaccaagggagaaataaagcaggcctggggtggggagtgcgtttgggggtgcactccacaggggaggctggaatcaacaaaacaagtttccaggtggccacagctcagcttagctcagaggatgcattgacctccatccggagccagcctgcagtccatggctggctgagacgatggtataaaaagtgcactttgcatgcttcagggtgtgcaagctcctggagctatccacattcagagcctcccatagcatccggctggggctcaacttcaactaaaccacatctttgctgcctcatgttccttccctcacctctagacaggcccttcctgtgagcacgcctgcaatcctcactgcttcaaaagccacatctcagctctggagaactcgacctaaggcaggggaggagggcatttgaggcagaggagacagcatgggcaaaggctaggaggcatgaacttgaaggcacgatcttggaagataaggaagctttcctgcaatatttattttaaagatgttttccacatacatgtagtgaaaggtgctgcttgtccagggtttgtgtaaccttttaaccttaatacgtaact

RC:

agttacgtattaaggttaaaaggttacacaaaccctggacaagcagcacctttcactacatgtatgtggaaaacatctttaaaataaatattgcaggaaagcttccttatcttccaagatcgtgccttcaagttcatgcctcctagcctttgcccatgctgtctcctctgcctcaaatgccctcctcccctgccttaggtcgagttctccagagctgagatgtggcttttgaagcagtgaggattgcaggcgtgctcacaggaagggcctgtctagaggtgagggaaggaacatgaggcagcaaagatgtggtttagttgaagttgagccccagccggatgctatgggaggctctgaatgtggatagctccaggagcttgcacaccctgaagcatgcaaagtgcactttttataccatcgtctcagccagccatggactgcaggctggctccggatggaggtcaatgcatcctctgagctaagctgagctgtggccacctggaaacttgttttgttgattccagcctcccctgtggagtgcacccccaaacgcactccccaccccaggcctgctttatttctcccttggtacactgtgctgtggacttactccctcacaatcagcccaaaattctgggttgtgaagccagaccctatgacggtagtaacctcccgctagttattatcatggttgtaccttgtattcacacagagcaggaaggatatcagtgtgcattcagcttggtgttactgatcacgtcctatctggagacaagatgctctgcccagtgtgcagtgggtgcttttcgtttatcctgcaaaggccacactggaggttcatttcaccgtcatcttgcattttgtctgaagaccttttccctagaccgcctcttctctgcatccctcctccttaattttcctctgtgtgttttccaaggcattctctgcactgccagggacggcctaagatttaccagccctgccgaagaagatgaaatgctcagggtcactgggcaggacgaggtaataagaaaaaagaaacggtcaacagcaggcccagcccagctgaagcaattaccaagtttcttctcaatttgagattgcaatgggagagcagagatggctatggcgaaacataaattatgtcatctctgtgctatcaaaggttcttagcattccttcctgctgtccttttcattttggtctgtaaatactgtgtgtccttttaaaaaagagtcattgttcatgtccgttcaaaggtgacaggaaaaccgcaagtttagcgtctctgtgggtgagtgtcttaaaggaactgtctgttgctgagtcagtgggtgagtaaggaaggacgatgtcttggccaggtgtagagagtttctgaaccctacaacattggctgagagcagaggctccatcattagaggcacctgaattaaaagctccagcccaccacttacgagccatatgagttaggctgtttcctctctgttttaaaatcggggacaatgagaggatctacatcatggggtgtgataaggatcaattggcattatatgttaaaagtactgagaacagcctgcagaggatcagaatgatccttagtaaagttaaccgggttttccctttttttttttttttttttttttttttttttttttttttttttttttttttttttttaatccagtaataaacctttatattttcatcagtttaataacatttttgccagtaattgggtatattttgaggtgctgtttgtccagggtttgtgtaacctttaaccttagcaatacgtaactgaacgaagtacaacaa

chr22: 29065773-29065747(-)chr16:5902158-5902542 atgcat chr16:5900972-5902138 polyT chr22: 29065912-29065835(-)

atgcat chr16:5900972-5902138 polyT chr22: 29065912-29065835(-) (Primer gap) chr22: 29065773-29065747(-)chr16:5902158-5902542 atgcat

2) For 3´ transductions in c368T sample

Consensus sequence for insertion at chr1:115147190

ctgttatgcttcgttcggtgcatattgctaaggttaaaaggattaattccacgtaactccagcacctttcactaagcatgtatgtggaaaacatcttaaaataaagatagataagaacgtttttgtgaattaaaaagtaatgtgaacatgttaaggtatttgaacttacatttaaattggattataagtaacttaacattttaattttatctctgtttctaggttgagtctcaaaatcaagtctttttcattttctgtccacttgggagcatttctgattgtatgtaaaatagaacttttttttttttttttttgtagaactgcaaactgaagagactctcaaatttgttttccttttgacactggggtattattccacgtttttttttaagccccacataggaaatgttctggttctgcttcttcctttcctctaccacatccttgacttgccacagtattcacatagcttcactaatggctgtgcccatttctcaaatactctgatttattttttgcaggtctaatgaatagaaatcagataaattagccccattacctcttgagttctggagggttggcatccagtcctttgaatcaggaaaacaaaattttgaataactttaagtctctcttccatgtctttggactgcttatagccatgatcatcctgaaaagataacaccataggtgacttagaattatactatgtaagtgagaataaataaaaaactcagccttgtctcaggaaaaaggtctctgataagcctggtagttgctgataaagcctcagcatggttttgtactgcttaactaaattaaaagtcaattctggaaatcactgaggatgcactgagaacagagttactaagaaaaaaaaaatcctgttttttacttagaaatggtatctgtggatatctcagcatctactgtaagagagattagtgaaaagtttctatcaagaggtaaaatcttaattcaggattcacacactaaatgagaacccagtatttttttatgaaccgtggggtataagataaaatccattcttcaatgtactgagaacaaaaacctagtcggctcaagacactttccaaacaataatcaaagctttgaaatggtagatgaatgttatttagaaaagatacggccagatgcactggcctgtaatcccagcactttgggagcccaaggtgggcgcatcacttgagccagggttggagaccagcctgggcaacatggtgaaaccccatctctacaaaaatagaaaagttggctgggtgtggtggcacatgcctgtagtcccagctactcagaggctgagaagtgagaggatttcttgagcctgggaggacgaggctgcagccttgacctcctggggctcaagtgatcctcccacctcaacctcccaaataactggaactacagatgtgtgccaccatgctataatcttttaattttgtgtgagagataagctcactatcttgacagggctggtcttaaacacctgcgttcaaataatccttctgtctcagcctcccaaagtgctgggattatagacatgagccactgtgcctggccttattttccttttgttgaatgcccaatttagtcaaacagatgaggtaagctacaaagactttgatacgttatttctcagttccaaattcttctcttttagttctgagaggctgtatagagcagcaataggcaaattatatccttaatatgtaaaaaattattaagaaaaatatcaactcattagaaacatgggcaaaagatatgtgttgataatttacaaaatgaaaacctaaatatacccaagaaagaaactcagactcaataatcaaaataagtccattttttttttttttctgagacggagtctcactctgtcaccaggctggagtgcagtggcgcaatctctgctcactgcaacctccgcctcctgggttcaagcaatgctcctgcctcagcctcccgagtagtgggactacaggcatgtgccatcacacccagctgattttttattttttagtagagacggggtttaactatgttggccaggctggtctcgaactcctgacctcaggtgatccacccgccttggcctgggattacaggcgtgagccacagcacctggccaagtcagttttttttttttttttagaactgggtctcactgaacagaacttaattttctgaagacatatttattatctaccttctttcctcctccctccttccttccttcctttattattattattattattttggacagggtcttgctctgttgcccaggctggagtactgtggcacaatcatagctcactgcaaccttgaactcctgggctcaagtgatcctcctgctctcagcctcccagtagctactgcacgccaccgtgtctggctagttcttttggtatttttgtagagacagggacttgctatgttgcttgggatggtctcaaacctggcctcaaatgatcctccaccttagcctcaaagtgctaggattacaggtgtgagccactgcacctggcctaaaatcaaacttgaacaatatatacatgtctgtatttggctaaaataaaagattggaagaaatatgacaaaatatttacagtgattacttttttgagtaaccagactgccagtgatctgtaatttctgcttttttatttactcttctgtgtttcccaaaatagtctagaataaacatatataacttgttgtgggaaaaagccagccacagagcagccaggccactatgaagctgctgtttataaaggtagcagtgcaactgacaacacaagagcatcaacagtgatggctgagagatgagagtgtcaatcaggctaaactgcttatgagagatagtagcttagtctgggaagcaacacagaagttgaagtgcagagatatttaagcaacagctgaggctattatcagaaggtcattaccatgtgatacggttcaatgaataaagcttttcaaactaaatggcatcatcaataattatagttttgaacaaagcaggcatctattatgttacaagcagattcagagattatgatagtttattaattaaaagtagtaatttcctctagatacctggaagaaatcctttaataacttcctttcttaatatactactttcccaaactggagcctcctttgtacaggttccacagaggaacataaaaagctgggctgtggattattctgttaatttgaagaagaaagagataaaagatgaagacactgctctgtttttttttttttttttttttttttttttttttttttttttttttttttttttttttttttttttttatttttctcatcagtttaataataacatttttgccagtaattgggtatattttgggaggtgctggtgttaccgtggaatgaatcctgggtaaccttagcaatacgtaactt

chr22: 29065782-29065647(-) chr1:115147187-115148357 ctgcag chr1:115145259-115147190 polyT chr22: 29065893-29065832(-)

ctgcag chr1:115145259-115147190 polyT chr22: 29065893-29065832(-) (Primer gap) chr22: 29065782-29065647(-) chr1:115147187-115148357 ctgcag

Consensus sequence for insertion at chr2:182004540 (Twin Priming)

ctgttacttcgttcagttattgctaaggattaacctggtaactgggactaaagactcagcacctttcataagcatgtatgtggaaaacatcttaaaataagaatagataagaacgttttttgtgaattaaaaaaagtaatgtgaacatgttaaggtatttgaacttacatttaaattggattataaaaaaaaaaaaaaaaagaaaagaaaaaaagaaacttcacatggggccacatgtctaataaaggtgactgaatcccaaaattatattggtaacatggaaattcctgataggggaagaatattcccaagtgtgacagttagggccaaaataaaaggtacaatttacccaattattacctaagtacccggccttattaaatatatgttcctcaatatcaaattgtactaaaaatcaggaaactaagctcctagtagagaactggtcaagggaagatctttgtggagtcagcagtttcctctccactacaatttggcaaaataatctcataagcacaagaaggttaatatgcttgcattaaaaaatgttaagttacttataatattcaatatttcttaagcatttgctttgtatacaagttttgcatatatccttatattaccactagattttaatcttcattttaaagagatgaagaaattgaggctcacacaacttaagcaagtttcacattacaaatctgataaacacagcctgaatttaaattactgttgtctcatgccatggtcctctctcattcatttctctatattgctccattttaacatttgctgtaatctcaacattaaagcattagtgcctcaagtttgttgtttatttaattctattgcttgacttgaaaaaatttgcttcaatctattgtcttttcctcttttcatgaggggatgctattgaggaagtttctgaatgattccattgtagttgaaaacataactatgtggctctctcaaactattctcctaatgagttaacactaaaatttatgtattgagcatctgctatatgccaagcactgtgctagatgctttatgaataaagatgtggtctttgccctatacctgcaggatttccaattacataatgcagaatcagaattctgggtgtcacatgaccgccagcatccatgggtgagttgcataacactcctctgtcatctagctcatgtgagaaagaaaattggaactgctgttcccaagaaatggcactcttcttggagaccttcagttacacaaagagcataatttccacattatcctctggcatactgtctgggataagtctatgataactttatgtgtaaattatatacacagttctgctaatgacaggcatacaagactgctttttttgatggtagtggtgcagaaaaacaaaatcaatttgttttccaatttttttataaaatgacacctgagctatctataataatcaacagagactctgagagttagatggtaggtaggtaattgcagttattatctaacagacagagtactggagttggcaagcaatgtcaaagggtgcagacttaccaaccacctcttaactggcttctttcaagtattttcattttaagtcgttcaaacttttaatttcatacttatctcagcttttcatcagagactgcactcttttttgcgtggtggtttaattttttacatatgaaagaacacttgcaaaggagacaaaaaaagtaagggaaaatgaatatttcatatacttattgtctctttgatgccaaaattccatatgtagtacatactgtttgccggatatgaaggataatttggaggataagacagtgttcttttctgtagaaactcacaattctctcattaacaagtctaaaacaggaaaactttttgtatcctggctatcaacgttatttggaagttttctttttagtttattaaattcaacaattttaaactaaataaaaatggaaactagaaaatagatgtagttattataatattcaatatttctttttttttttttttttttttttttttttttttttttttttttttttttttttttttttttttttttttttatatttctcatcagtttaataataacattttttgccagtaattgggtatattttggaaatcagcagaataaggattgtagactctcttgcatctctaaaagaataatttcactaagcatgtatgtggaaaacatcttaaaataaagatagatgagaacgtttttgtgaattaaaaagtaatgtgaacatgttaaggtttttagacttacatttaaattggattataaaaaaaaaaaaagaaagatcaaaacttcatattttaccagtaattgggtatattttgggaggtgctgtcgattccgttgtaagtcgtctgtttaaccttagcaataatgtaact

RC:

agttacattattgctaaggttaaacagacgacttacaacggaatcgacagcacctcccaaaatatacccaattactggtaaaatatgaagttttgatctttctttttttttttttataatccaatttaaatgtaagtctaaaaaccttaacatgttcacattactttttaattcacaaaaacgttctcatctatctttattttaagatgttttccacatacatgcttagtgaaattattcttttagagatgcaagagagtctacaatccttattctgctgatttccaaaatatacccaattactggcaaaaaatgttattattaaactgatgagaaatataaaaaaaaaaaaaaaaaaaaaaaaaaaaaaaaaaaaaaaaaaaaaaaaaaaaaaaaaaaaaaaaaaaaaaagaaatattgaatattataataactacatctattttctagtttccatttttatttagtttaaaattgttgaatttaataaactaaaaagaaaacttccaaataacgttgatagccaggatacaaaaagttttcctgttttagacttgttaatgagagaattgtgagtttctacagaaaagaacactgtcttatcctccaaattatccttcatatccggcaaacagtatgtactacatatggaattttggcatcaaagagacaataagtatatgaaatattcattttcccttactttttttgtctcctttgcaagtgttctttcatatgtaaaaaattaaaccaccacgcaaaaaagagtgcagtctctgatgaaaagctgagataagtatgaaattaaaagtttgaacgacttaaaatgaaaatacttgaaagaagccagttaagaggtggttggtaagtctgcaccctttgacattgcttgccaactccagtactctgtctgttagataataactgcaattacctacctaccatctaactctcagagtctctgttgattattatagatagctcaggtgtcattttataaaaaaattggaaaacaaattgattttgtttttctgcaccactaccatcaaaaaaagcagtcttgtatgcctgtcattagcagaactgtgtatataatttacacataaagttatcatagacttatcccagacagtatgccagaggataatgtggaaattatgctctttgtgtaactgaaggtctccaagaagagtgccatttcttgggaacagcagttccaattttctttctcacatgagctagatgacagaggagtgttatgcaactcacccatggatgctggcggtcatgtgacacccagaattctgattctgcattatgtaattggaaatcctgcaggtatagggcaaagaccacatctttattcataaagcatctagcacagtgcttggcatatagcagatgctcaatacataaattttagtgttaactcattaggagaatagtttgagagagccacatagttatgttttcaactacaatggaatcattcagaaacttcctcaatagcatcccctcatgaaaagaggaaaagacaatagattgaagcaaattttttcaagtcaagcaatagaattaaataaacaacaaacttgaggcactaatgctttaatgttgagattacagcaaatgttaaaatggagcaatatagagaaatgaatgagagaggaccatggcatgagacaacagtaatttaaattcaggctgtgtttatcagatttgtaatgtgaaacttgcttaagttgtgtgagcctcaatttcttcatctctttaaaatgaagattaaaatctagtggtaatataaggatatatgcaaaacttgtatacaaagcaaatgcttaagaaatattgaatattataagtaacttaacattttttaatgcaagcatattaaccttcttgtgcttatgagattattttgccaaattgtagtggagaggaaactgctgactccacaaagatcttcccttgaccagttctctactaggagcttagtttcctgatttttagtacaatttgatattgaggaacatatatttaataaggccgggtacttaggtaataattgggtaaattgtaccttttattttggccctaactgtcacacttgggaatattcttcccctatcaggaatttccatgttaccaatataattttgggattcagtcacctttattagacatgtggccccatgtgaagtttctttttttcttttctttttttttttttttttataatccaatttaaatgtaagttcaaataccttaacatgttcacattactttttttaattcacaaaaaacgttcttatctattcttattttaagatgttttccacatacatgcttatgaaaggtgctgagtctttagtcccagttaccaggttaatccttagcaataactgaacgaagtaacag

chr22:29065832-29065860 chr22:29065662-29065886 polya chr2:182004515-182005434 ctgcag chr2:182004009-182004540 chr22: 29065668-29065285(-) chr22:29065662-29065782

ctgcagchr2:182004009-182004540chr22: 29065668-29065285(-)chr22:29065662-29065782 (Primer gap) chr22:29065832-29065860 chr22:29065662-29065886 polyA chr2:182004515-182005434 ctgcag

Insertion length: exact length could not be determined because of duplications

Consensus sequence for insertion at chr2:229159082

actgtgcttcgttcagttacgtattgctaaaggttaacctggtaactgggacacaagactccagcacctttcactaagcatgtatgtggaaaacatcttaaaataaagatagataagaacgtttttgtgaattaaaaaaagtaaatgtgaacatgttaaggtatttgaacttacatttaaattggattataagtaacttaacattttttaatgcaagcatattaaccttcttgtgcttatgagattattttgccaaattgtaggacagagaaactgtttcttatcatacttaaggtttgtgttaatgataatgctggaggggggtataatggggcacacccaaccaaccccacatcccatcatggcctgaaccagtcttttgagttaaattttagggtgccctggatgaggagggagttaattcaaatggttttggggggggttgaattttattttttagtttacatacatatatatatatatataatgtaaaacctaaagtaaccactgaaaaagctgtacagagatatactcaaaacccctttaaataaatcaaaatgaattctaaaaaaagtttaaataacccataagaaagcagaaaaaaataggtaggaaatgaaagcagaagaataatataaaatttttaaaagaagacttatcttctagcatatgaataattacattatatacaaatggtctaaacagactgtgttaggctgaataatgccccccccagctttaagatgttcatgtctcaaatcctgaaaatgaaatattttgggttataagacaaggagacttaagattgcaagtggtagtaaagttgtcaattaagtgactttaaaatagaaacaccaggagggagagtcaagatggacaactggatgcagccaggaagagctcctcccactgagagaataggaccatcaagtagaccagcacacaagtgggagatccaaataggtcttcagaaagcactgactgagtgggcagaaggatgatgtagacctgggctgaaacggaaggaaggtgggaatccagcataaggttgccaagcaccagaactcatttctggccctgaatggctccctagggaaggggagcgagtgaaataggtgtgggacagcccacacctctgtaccctctgggatctagattcaggagaccacatgaccccatggatgtgtgagctggcacgaagaactgcccagagagttggcagagacagaattccagcctgcatggagaccagagggtcaggcacggaagtggctgcaatgaaacgtggcacatgggtgcccatccccccaaaaagctcaccatactcttctaggtagccctagccagtttggctgcttgacctggacagagcagggcagaagcaagacccagtggtatgctgtcatcaagagacccatctgacatgcaatgacacccataagctcaaagtaaagtgatggagtaagatctatcaggcaaacagaaaacaaagtgcagagattgctcttcttatttcagattaaacagacctcaaaacaacaatattcaaaaaagacaaaagaaaggcgttatgtaatgataaaggatgcagttcaacaagaagacatagctatcctaaatatatatacacctaacactggagtactcagattcataaaatgagttttgtagaagagacctatgaagggacttagataatcacacaataatagtgggggattattgagctctccattgtcattgagctcccacctcaagatcctagagaagaagaacaaaataaacccaaagcaaatggatcaacagcatttaaaacctctagcaagactggaaaaaaaaaaaaaagagaaaacacaaattacaggtattgggaagaaacagggatataaccacagacccttttaatgtagacattaacatgacgataagaaaatacaaacaactctatacatgtaaacttgataatttagttgaacaatttctcaaaattataaactactataacttacctaatatgaaattcatttgaaaatttctataaatattaagaaaattgaattaataatacaaagtaaacttccaaaaaaaaactttaaacctaagtggtttcattggagaattctatcaaaatttaaaaaagaattagcacaagttctatataatttatttcaagaaatataagagaaatgaacattttctaattattttttatgaatctagtattatcctggtaaaacaaatataatgaaaattacaattacaaactaataaaacctcataaagaagaaatggaaaattccttatcaaaatgtaatcaaatagaatggataatatataaaaaagagttaaacaccatcataaattgggattattatactgatacagatctggctcaatttttgaaaatcaatcaatataattcactagttaacagtcaactaagacaaatcacataatcatatatatctatacagaaaaagtatttctacaacatttaaaacatattcatgataaagacttccagaaagatagggatagagagtaacttatttaactcctaaacagcatctacagataatctacagctaacatggtatttaaaagttgaaaaatagaatactctctaacattggaaataaagttatagtgtctcttcttttcatccttactcaacatagtgctggaaattctagccagtgcaacaaaggaagaaaggggcagggctaggagcagggaggaaaatatacatgagaaatgaagaaataaaactgttttgatttcaagatgacatgattttctatgtagaaaattccacacatcttcaaaaactcccagaatcattaagttcaacaaatccacaagctatgaagctgacttacaaaaataaactgtatttcttttatatgctagcaatgaacaattggatagtgaaaattaaaaatatcatttacaattactcaaaaaaaatgaattagctataaatctaataaaatacacacaggatctacatcttgaaaattacaaattatgatgaaataaattaaagaactagataaatgaaaacaccatgtttgtggatcagaagactcaaagatatcaattatctccaaattaatatacataatttatgcataatgtgcataatttatgcagttcctataaaatttaaaaatgttgacaaggttgtgaagaaactcctcatcgctggtgagaatgcagaaatggtactgccactctagaaagcagtttagcagttttcaacaagactaaacatgcaactaccatgtaacccatcaattttactcctggccatttatcccagagaaataaaaactcatgttcacacacagtccttataggaatgtttgtagcagttttattaataataactaaaaaagtgaaaacaacccagatattcctcagtgggtgactgattaaacaaactgtttacattcatactatgaaatacggaatctctctgtattatttcttacaattgtacaagaatctacaattatctcaaagtaaagtattcttacaattttttgtaaggtctctatcattcttttattttctttaacatatggaatataattataataacttgtaatgtccttttctatttattctttcattttttccattttttttgattgattttctaggcattatagatcaattactttttacatatctttttttttaattgaattgtagatatggtgaactttaccattttgtgtgctggatatttttgtattcttataaatattttttggctgtgttctgggatacatttaagttacatgtaaataacttaatctttttttgacatttgctttaatattttgtccagatggaattagagccacctttcttctagtgctaattttgcccttatccttaggcaatactcttctgaatcctctactgctagtatcaaaatttgtgttgttattctacctcggctgatggggacaccaactattctggcctgttgtgatctctggctattgttctcacaaatcttttttgactgttccttcccccgactccactcagccttaggcagttttttgctcataggcacacatttagcagtactcagctgaagattaaagggcaaaacaatgtggatttttggcgctctcctctgtgcaattctctttgttcaagtactttcacctgcagcatattcaacatccttatagggagaaaaaaaactattatctcagtttacccttttgcaatctagttaagaacataggttttcataaaggtaagattaatctctagaatggctctctctgccatctttttgcaggatgctatacacaggtaggagagagggcagctaagagcgacagagtgcatacaatcaccaggcagtatggtcattgtttttttgtctcttttagttctcactgtgattctaggaataatatgtgaccatattttacttacatacaaaaattagaactagaatggtggaagtgaaaagcccaacttcatgagattttaagtggaagagctgaagccaagtcgtaacctatcttgatcagagtttaagcttttgttcactatcttggctgagtatagacacatgaggcttatgtgagtgaaagagaatttttattcaatagaatgtgaatacaaagattaaaatactcttcacaattctagaagaaaacagatattttttaatctcagtcttagaaaatgtgtcatgttgaaagtattgtgctatctttcctctcactgtcacattttctccatttacaacaccattatccacttggtatttatccatgaggcaaccaatagtctagccctgctttaccgtcgtttcacacttacctgtagcacagtcaagaacattgtggagagaaagaatctcgttttcattttactccattcaaaactaaattctgaactacagtttctttctctactgtcatgttttattttcaatttaaattagttttcttgctattgaaaatactaaggtctctttggcattaaaacgctcttaataactaactggctgtcagcagaatgaaatgggaatgaggatctgaggaactgaacccaactacctaggacttcagtccttctgcatgtcaataatcacctgcatgctagttaagaatgaaatacgcagctgggcacggtggctcatgcctgtaatcccagcactttgggaggctgaggcaggtggatcaactgaggtggggagttcgagaccagcctgaccaacatggagaaaccctgtctctactaaaaaatacaaaattagctgggtgtggtggtgcgggcctgtaatcccagctacttgggaggctgaggcaagagaatctattgaaactgggaggcagaggttgcggtgagccaagattgcaccatcacactccagcctgggctacaagagtgaaaccccatctcaaaaaattaaaaaaattaaaaaatttaaaaatttaaaaaaagaatgaaatatgcagtaagcttgggacttgtggtagatctagaaatctgcatttttgattagcatttatcaagtctgacgtaaatgactggaccatactttgaaaaatattcccttagagcaagggactgttaaaagataagagtttttaacagagaagctgagaatgctatctcatcgtggatgtttgaagagggctaaagctttgggaagagattagggatcacatgaatgtcctgatatgcacagtccattctacaaccatgaagatttacttaacttttcgagttgcagtcgtcttacctgagaggagaccttcaaatatgggacaagaggagccattaaatgaaggtgatgattcaggtgcttatggacatctttaagaaagggaacagagctgttgataactgaagtgaagacattaattaggcagatatagtgtacagaccagtgaaatgcacagtgggaaaatatagggtcagttcagtagcttaaataataagtagtccttttatactacataactccatagaaaggtaatctaatttctatcctcaagtggataaatggtagtgagcttagataatgatcttgaaaaaaatatatacaatttttcacttactatttattttagaggtattcaaaattaaaatgtgtgctcatgttttcagttaacaatataatatctaataaattgagacttattagcaataattatttttaaaatatgttttatatttttaaaaaatatttttaataactcatttaaaaacaatttttaatagaaattaagacattttctgaaagagagagaaataaaagaatttgtcagccttgagctatcctaaaaaaatggctaggctgttcacgggggctcatgcctgtaatcccagcactttggaaggccaaggcaggtgggtcacttgaggtcaggagtctgggaccagcctggccaagaggtgaaactccatctctactaataatgcaaaatttagccgggtgtggtggtggatgcctgtaatcccatctactcaggaggctgaggcatgaaattgcttgaacccaggaggcagaggttgcaatgacctgagatcatgccactatactacagcctgggcaacacagtgagactctgtctcagaaaaaaaaaaaaaaaaggctaaataatgttatctaaacaaacaaacaaaaccccaaaaactataaaaaagaaatcttggaacatcagaagagaaaagaacatggtatgcaaatatataagtaaatacaacaggctttccttttcctcttgaattttcaaaactatgtttgatggttgaatcaaaaattataagactggtatggttttaaatatacacaaaagaaatatttgatatcattgcattatgtgtgagaggagaggataagcaatgtaaaaggaggtaaagtttctatacttcattcaaacaggtaaaatgatgacataagtagactatgataagttatatatgtgaacccaaaatatctgaagacagatctcagtcaatttagaaagtttactttgccagtttaaggatgtacccatgacacagcctcaggaggtcctgatggcatgtacccaaggtggtaggactacagcttgcttttatacactttagtgaaacataatacattaatcaatacatgtaagatttacattggttttgatctggaagggtgagacaacctgaagtgaagggcttccatgtcataggcagatttgaacatattctgattggcaattggttgaaaagttattatctgtagaaaggaatgtctgggttgagaagatctaggttttgtcatacaaatgaagctcccaagaaggaggctttagaagatggaatggactgtaaatgtttctttttttttttttttttttttttttttttttttttttttttttttttttttttttttatccagtaataaacctttatattttctcatcagtttaataataacatttttttttgccagtaattgggtatattttgggaggtgctggagtcttgtgtcccagttaccaggttaaccttagcaatacgtaaactgca

chr22: 29065782-29065578(-)chr2:229159075-229162994 ctgcag chr2:229155905-229159082 polyT chr22: 29065908-29065832(-)

ctgcag chr2:229155905-229159082 polyT chr22: 29065908-29065832(-) (Primer gap) chr22: 29065782-29065578(-)chr2:229159075-229162994 ctgcag

Consensus sequence for insertion at chr6:70787202 (Twin Priming)

actgttgtacttcgttcagttacgtattgctaaggttaaaaagattcattccacggtaacaccagcaccttctatggccatttttatggaatgacaaactaagttcaatactgatagagttgtattctgtgtatacatataatagagtatatactgtgtgtataaaaattcaagcatatggaaaaacttatttaatcagtaataaacctttatattttctcatcagtttaataataacattttttgccagtaattgggtatattttgggaatctacaaatctttattctactaattttcaactttttaaaagaattatggcatcacatttacttttgcaatagcttctctcttgataattaaaatataatgtataagaaaaacaaaagtgctataaaattaaagttttgagaattatgtgccattacctaaggtaactcaaaatactatagagtttacaaagttgagggcttatttattggaaacgtaatttgctggctttcaaaaagtcattaggccataaaaatgtttcatgctccgaatgttttgcatttctgaaaatttatctgtttatatttttcaaattgattaataacttgtgattaattcttggttttggcagccatccacatatattcgctccatcccaaatggataaataactttctacttgacatttcaatgtagtaaacatatgaaatagttactaacgtgttcagtgtaatgttctgaataaagatcctgatgcaggtgtattagcatatcgccactttggcagaatgtgccttctccttttccattctctttaagtgtttctgtggctaagtaggtaatatttataagactccttcatagaacacctgtcaccagagtgcatttaaagtgcttccggcttaatcccataatatacctgagggctaagttatgataaaacagataaatttagactagagtcttgagtgatttggctgaagtcacaaatttgatcaattgctctcctgagaaaaagagcagttctgactctcctcctggtccctcactggccaggcattgctggtttcccaaatgttggtgcaggttatagggacagaatgtgtgctcatcacaatgtgtggagaccataaagggatcagctgtacctacctatatttgtcccttctagtgtgtggctttaattagaaaaaattaagttagtgattttttaagtaacttttttttaaaagttagtgatttggttaaattctcaatatccctattttaaaacatgcaaatctgggtttttttcagtgtgatacacaaaatagacaatgggtgtatattatgcagaacagttagaataaatagccctgcagtcatctttctcacggttgcatagacacattgttaacatctctcacataataaatacaccattcggactcagggtcctttctagtgttcaagaagtgcattgtctagtgatttgagagccaaaatgacacacctggtgtttggttgtgtttgagtaccataatacagtggtcacaacatacgttagccatactgttgtgaatttagaaaacacaatggataatgcttacatacatgtacatatatgtatatgtttgtgttggacataatagagatatcaaataaatatcaaattaatctacaataattcacacaaaaaaaagtcaagtaaaagttactggtttgaggaaaatgaaaaagtagtttctaaggtatttctgaagagattcaagagcgactctcaatactaccattcaagaattttagaaataaaaacacagtacaatctacgtacatcatataaaagataaattttcaacttttttttttttttttttttttttttttttttttttttttttttttaatacaggaattaagtacttgatctgcccccccattggttttattttggagtaatccttttaaccttaacaatacgtaactt

RC:

aagttacgtattgttaaggttaaaaggattactccaaaataaaaccaatgggggggcagatcaagtacttaattcctgtattaaaaaaaaaaaaaaaaaaaaaaaaaaaaaaaaaaaaaaaaaaaaaagttgaaaatttatcttttatatgatgtacgtagattgtactgtgtttttatttctaaaattcttgaatggtagtattgagagtcgctcttgaatctcttcagaaataccttagaaactactttttcattttcctcaaaccagtaacttttacttgacttttttttgtgtgaattattgtagattaatttgatatttatttgatatctctattatgtccaacacaaacatatacatatatgtacatgtatgtaagcattatccattgtgttttctaaattcacaacagtatggctaacgtatgttgtgaccactgtattatggtactcaaacacaaccaaacaccaggtgtgtcattttggctctcaaatcactagacaatgcacttcttgaacactagaaaggaccctgagtccgaatggtgtatttattatgtgagagatgttaacaatgtgtctatgcaaccgtgagaaagatgactgcagggctatttattctaactgttctgcataatatacacccattgtctattttgtgtatcacactgaaaaaaacccagatttgcatgttttaaaatagggatattgagaatttaaccaaatcactaacttttaaaaaaaagttacttaaaaaatcactaacttaattttttctaattaaagccacacactagaagggacaaatataggtaggtacagctgatccctttatggtctccacacattgtgatgagcacacattctgtccctataacctgcaccaacatttgggaaaccagcaatgcctggccagtgagggaccaggaggagagtcagaactgctctttttctcaggagagcaattgatcaaatttgtgacttcagccaaatcactcaagactctagtctaaatttatctgttttatcataacttagccctcaggtatattatgggattaagccggaagcactttaaatgcactctggtgacaggtgttctatgaaggagtcttataaatattacctacttagccacagaaacacttaaagagaatggaaaaggagaaggcacattctgccaaagtggcgatatgctaatacacctgcatcaggatctttattcagaacattacactgaacacgttagtaactatttcatatgtttactacattgaaatgtcaagtagaaagttatttatccatttgggatggagcgaatatatgtggatggctgccaaaaccaagaattaatcacaagttattaatcaatttgaaaaatataaacagataaattttcagaaatgcaaaacattcggagcatgaaacatttttatggcctaatgactttttgaaagccagcaaattacgtttccaataaataagccctcaactttgtaaactctatagtattttgagttaccttaggtaatggcacataattctcaaaactttaattttatagcacttttgtttttcttatacattatattttaattatcaagagagaagctattgcaaaagtaaatgtgatgccataattcttttaaaaagttgaaaattagtagaataaagatttgtagattcccaaaatatacccaattactggcaaaaaatgttattattaaactgatgagaaaatataaaggtttattactgattaaataagtttttccatatgcttgaatttttatacacacagtatatactctattatatgtatacacagaatacaactctatcagtattgaacttagtttgtcattccataaaaatggccatagaaggtgctggtgttaccgtggaatgaatctttttaaccttagcaatacgtaactgaacgaagtacaacagt

chr6:70787188-70787647ctgcagchr6:70786149-70787202chr22:29065829-29065808(-)chr22:29065831-29066032

ctgcagchr6:70786149-70787202chr22:29065829-29065808(-)chr22:29065831-29066032 (Primer gap) PolyA chr6:70787188-70787647ctgcag

Consensus sequence for insertion at chr6:133527459

ttggtatacttcgttcagttacgtattgctaaggttaaacagacgactacaaacggaatcgacagcacctttcactaagcatgtatgtggaaaacatcttaaaataaagatagataagaacgttttttgtgaattaaaaaagtaatgtgaacatgttaaggtatttgaactaacactgataaactttttataagtcctttctaaaattaatcaaaaattatacttttttagtatatgtgtccatattggggggtggcagcaggtttaataataaattggtaggaaaacatttatttagattcattcaagttataagtgcataatgaaaggaaaggaaatgtaatttagagccagaagttctatgtttaagcattgactgtagtctgtatcaacagtttgattttttgataaattattttctttgagcttcaggttcttaatctataaagtgcaggtaataataataacaataatcagctctacagagttgtaatagaaccaagtaaatgcaagaatgttaaagtgtatgtgtacatttgtaaaaatacttaatagaggattaatattctacactataaaaatggcttcttacttaatggagttaaaggagaaataaatgaccatgtaagtacagagggaaagtataaactaatttgttttttgcttcttttctgtttttttttattattaatttttcatacttacttttaaaagttgataagcaggaagactacttcattgtgcctttaataagtagatactagttgtgaagaggtaatgatggcaggatttaaggactgtgttctgtttgcacctatggctaactcagtgtggccaggttgaagtcctttactgctccgtaagtctggttgtttcaactatgaaataggagtagtcggcttagaaaagctgataacccacagagggggcccctgactctctgaaccacagagaggcatactctcctcggcctgggacccgccagacctcttagaggtcaggctcccagttgctggacacctgagctgatcctgggtgcccagtggccccaattcacttcctcggggctgtgggacttgggagaaaaggaaagttacttgctggcagccctctggcatgacatattttctctactacagatgtttcaggccccatggatgacagtacccattcttcatatttcacagggtgccttttgttacccttgtgtatccccttgataaaactttttttaaaattaaaatttgacatattttggtggcaacaatgttattctaaaatatacttttgaattagtttctcagaatacttctcagaatactctctagaatataagcagccctagctacaggggagaggtagggaggagacaaaaaaaaaaaatgttctggatttagttggcattttggttcttactttccaatacctgttacttttttaaaatagttaatttagatttgtaaaatgagttgagtatgggttgcacctaatgatattagttatggtctattttggttttgtggcatgtcccatagatatatgtcttgaatattcttaagtgaaaattaaaagggcatcccatcgtttgaaatatatcagtgaatttttcaacaatttcctttttttttagagtacaatccatacccctcagttcaattttattttctctaaatgttccattttctttcctccaaattctattgctttgtgtgttgtgttttctttcaagcatacctacaataaagccatctgttgtcaagggtcattcctccatgaatcatgctctgtcaaaattttgaggttataaatgttcagctaattgagcagtctgactagctgaatacttgtaagtgctttataatatctttcaacacttcaaaaccctgacccacttaaactccagagtcctagagcaaacaggacaagttattcacatgaattctcactctgataaaactaaaacatgttcatgaaatttcctcagaatttggaataaagtgaatgtagctctacttttaaatatttatttttgagctgtgtctacattatgccaaaagcaagtggttcgagtaaaaaaaaccttagccagttggccaattattttgaccaccctgtattaaaaattaaagagctaaattaacttgctttccccttgccaacttgattgggtgttttggcatcatggatacttttagtttcgaagaaaaaagtctcctctctctctgtctctcctctctctctctcgctctttcccccctctctccctgtttcccctgcctctctttccccttccaattactttctttttttttttgtgagtcttcacagagatgagacactgaaatgaatgcacaaatgcattgtgaacccactggaattcttgttcatgggcgtcaggagcagtgtacctgcatattgagcatatctctgctctaatgtgcatgttcaattgtctctcataagacttcatttataaaacataagttcaaaataaaagtcttgagaatttcaagatggagacacagagcattatactaagcgtggggatctgtgcacctgcacagaagatgcttgcccaggaagccggccctgtgggaagcacattctgcctatatccttcccattgctgccccagttatcctcattggtgggcaccagctggaagccatctccacagaattggcagataaatcttgccttgtcttttttcccattcccatggttcttccccacctccactcgactttttttttttttaagacaggctttctctttgtccccaggctagagtgcagtggcacaatcacagctcactgcagcctcaggaattcctggagctcatgagctcaagccatcctcctgcctcagcctcccgagtaactgggactacaggcacatgcaccactcctggctaatatttttattttatttatattttgttttttttgtagagacagagagtctcactatgttgccaaggctggcctcaaactcctggctgcaagcagtcctctcatctcagcctcagtagttcttcccacttttcaacattttttcctcttcaccccagaatcaggctcttcccgtactgactggaaatattcagcttcatctgtacctgcaaaaatgagttgtggtggtttgaggaaagactcccatgctagtagaccttcttcaggagatatttgggaaatagcaactctcatatgttgaagataaaagtattctgtcatgttttttggactgtgcgctgtgtttggaatcactcctatcaatataggtaatgaggagctgcaaaaaaattttaaaaatcggaaaaggaaatcaaaagttaggaaatatttcattttggcaaagtccttctggcatatagcatggagttcaattggaggaatttataataataaatcatagaggctagctaaaggaagccattgctacagccctttgtaaaatgacagtgttgcccaacactctggtactaaataaccctatgattcactgatttcacttatgcagtgggtatagcacagtcaagacttatttggttgacagcttcaggactaaggaaaatatctccccaaatagaagcagctgatggcagaaaggagacagaagccaggaatctctttaaggggcttgagagaagaggatgcctgagccagtaggcctcgtcttcacaaaatcacctggcagctgggaccctcaggaggtaacacaagcttgggggtggtgctggagcttgtcgtgttgaaaaacacagagactctcttgggatataagtttttcaagcctagcccatctctgcctcacttctagattggtttcttctgcggtgctttcccctccccaaatgactcaatctgaagatgggagaaagttggcagagaaacccatgactgttccctacaaaatctgcacatcctcatcagttcttaagagcttgctttcctgcccagatgactgaattaattgaattcttcattccatcctatttagatcaggaggaatcctattagcagaaacttttccatgtaaatacggacataatctacccaggttttctgtttttgttcttggttttgtttgggggatttttttttggttttcgttttggtttgtttgacagggtcgcactctctcacttaggctagattgcagtggtgtaattgtgactcagtgcagccttgacgtcctggagtcaattaagcctcctgcctcagccagtagctgcgaccacaggtgtgcatcaccatgcctagctaattttaaaattaacttttgtagagacagggttccactatgtcacccaggctggtcctgcactcttgggctaaagcaatcattccaccttagcctcccaaagtgctaggattacaggtgtgagccaccatgccgagcctgtttggggttttttttttgatttttttttgtttgtttggttcagtgtgtgtttgtgtgtgtgtgtgtgtgtgtgtgtgtgtgtgtgtgtgtgtctgtgaattatcttcatacagtgaatcagggaatttggagaaatttgagggatgagttacctatatccggcttgtgaaatttcttatttgaattaaagccatatgtggaagcctgagggttctgttgggctcttataccatacatttggattttaagaaagtttaggtactttgtcttaaataacaatgtcaggataaatgaaaacaaaactgtgaatcattatttttttgtttatgtcaatctttcttcttgttagcataatgtgaaaagtgctagaggaatatcaaatgcctgctacctcatttggaaattcccatgtaaatttcaagttaacattttttaaatgaataaatacaaataaactctgaaaggtaatttgtttttaattctactcttgaatgtgtaaaggtgtcaagtcactgttcataattggatgacctaaaaacacctttaaaagttatgttaaagagttaatgtatttagaaagacacagaatgtttttttcctctagaagctgatattctcgaatatagaagtaaaattaaaatttcagcagaatgaaaatgtattctattccatggaattattgaaatgtattttatcctatggaataaaatacatttgatggaataccgatgggattattccatcaattccacagaataaaaaaaaatgagccgggcgcggtggctcacgcctgtaatcccagcactttgggaggccgaggcgggcggatcacgaggtcaggagatcgagaccatcccggctaaaacggtgaaaccccgtctctactaaaaatacaaaaaaaattagccgggcgtagtggcgggcgcctgtagtcccagctacttgggaggctgaggcaggagaatggcgtgaacccgggaggcggagcttgcagtgagccgagatcccgccactgcactccagcctgggcgacagagcgagactccgtctcaaaaaaaaaaaaaaaaaaaaaaaaaaaaaaaaaatgaaagtcatacacttttgtgaaggttttctcaaaccatgcccccaaagatactgtaactctacattgctattagtgattagatagcagttgcaattattccatagtcaattctattcaaatatttcagaacactttcaaaatgttttaacttaaatacattcttttgactataccattttaaaatcaatattgatcaaaaaaagccctatgtatagttatttgacaaacaacacgacaaccaaataactgtagaggatctcacttcgaccacccaggcatcagtatctcaggacaccaggcttcatttttaaattagtttgccatatatgtctgaaatgtcttcttagtttacatgttctgtccagcccctatcaatgttccttagaaattcttaagattagtaagtgaatagacactcatcccatctctgtaagaatcacaggctctgatagacagaaatgacttcttgtctctctgtctctgtctcttcctctctctctttaatgaagcagctgcccactaaacttgatcccatatggtcctgtttgagaaaaagaagggaaaaattaaaatcagttctcactaactaaaggaaaattatatgtaaacaattgaaaggtcttttcctacttatggctgacatctctaccaaggtcttaattgcctgctgggcctaaaaaagggttatgtgttttttttaagctacatgtgctcctggccaaaggactctctgggacatgccatgagtggttttagatcaccagtaactcttgctattttagtctgtgtcaatacttgaagttgtaattaatttggatgatcctaaaatcataattatgacaattctgatatttgataagaataacttaccttcagttttggcagaagaaaatcatgctgcatttagcagaaaggcaaaaaaaagtctatgcagtgacaatgagtatctcacgtagaaacagaccacagaagccttgatagggcttaaattgtccttgtaagtaatttgtggactattacgcacttactagattaactgtgttctgcactgtattttctatcagtaataatgtatgtcgcatttattttgttttcttcttaactaagagttcattcgtggacaatatttgtagacaaagccaatttttgctttcttctatgtataaatgatatgtgcagccacagaatacaatatttcacttttgttctggactaaaccacagtaataaaacaaactagaatcattgttagattttctctcttgctcctgaggttttcctcttttaattcagtttatttgtaatgattacatttcttaaagggttttctgattcttcaaattataaaaagaaaaataatatatatacaagagggagaggtttgaggaaataatagtaaacatctaagatgttgaagttaggcttcttgtttggttaccagatcattcaaattatgaacactgaaaagggaaaaaggcttttgaatgtgtaaagaatgatctccgcaacactgataaacttttttttttttttttttttttttttttttttttttttttttttttttttttttttttttttttttttttttttttttctcatcagtttaataataacatttttttgccagtaattgggtatattttggaagtgctgtcgattccgtttaattctgtttacttagcaatacgtaactg

chr22: 29065782-29065683(-)chr6:133527443-133529634atgcatchr6:133522816-133527459 polyt chr22: 29065887-29065834(-)

atgcatchr6:133522816-133527459 polyT chr22: 29065887-29065834(-)(Primer gap) chr22: 29065782-29065683(-)chr6:133527443-133529634atgcat

Consensus sequence for insertion at chr8:88681299

ttggtatgcttcgttcagttacgtattgctaaggttaacctggtaactgggactgaagactcaagcacctcccaaaaatatacccaattactggcaaaaatgttattattaaactgatgaaaaaaaaaaaaaaaaaaaaaaaaaaaaaaaaaaaaaaaaaaaaaaaaaaaaaaaaaaaaaaagaaaatgttactgtaagctaaaatcatagcctcaaactgagatcaatttcacaggaacatatattaatgtatttattggtccaataaatccctaacacaaacatatgctggatatggtcagacactgataaagtttacagaacaaagatttagaaattttagtaggttataattgtaatatgttttaatgctgtaaagatgctgtttaaagcctcacataatgttagatagaaataatatacttttttctgttcaatgattaaattcagacatattttactttgcttggcagagtccacatctgtagtatattcagtcttggacgtgatattttaataaggggttggcaaactacaaatctttgaggtattatagaaaccagggtgtaatggtgctacatcaaaaaaacgtttgaaggaactgtttattttagcttgggaaggccagactaaaacattaatactggtattggataggctgtcataagtaaagggaataaacttgtactgtgatgcttcagagtaaaagataaagagaaggtgtttgtatgcttgtatattagtctcctagggctgccatacaaattatcacaagctggatggcttaaaaaaacaacagaaatttattctcttactgttctgggttggaagtccaaaagtgaggtgtgagcaagccatgctccctctgaaggctctggggaagaatgcttccttgactcttcctagtttttgtagttgccagaaatctttggcattcattggcttgcagttgcatctctctgatctctgcctctgttgccatatggcattctcctcaatgtcttcacatggccttcttataagggcatgaatcactgtgttaggacccattctaatccagcagtatgacctcattttaactagttacatttgcaagtcttgtacacacaactcttgttgtctacaatgataatggagcacctcatgaagcagtgagcttccagttatggagtcaattcaaacataagatgaaattcctaaaaggagtctctagcatgtagggctgaataactgaacttgacattatgggagaatgagaactacattaggtcttttatgagcctagtgaagtagatttcacctttcaaatattcagctgagattctatgatttgtaaagatgataagatgggcaagtgaaatatatacaagcaagatgaaaggatagtagtaagtaaggtttacaaccattttctctcctcttttttttcaatttaatattgacagtttccagatttgctgatggtggctgttatcttaatttcaaaattatttcactgagatgttcatgaattttttgaccatgaaattgcaagctgctagaatatggcttagtgtccacttaactgcatttttctgaactctgtatattactattaagattagggaaaaaaggttgacagtacttttaatgaagtccacctcagtgcagtcagtattcactgagggcccagcaagaacagtgaactgtcattaacataatttagtctttgtggagaagtgataattcaaaaaaacaaaagaaatgaaaaaattaaatcttcacaatgcctttttatacttttacaaaaaatgctttgatctcagactttcattaaacccttaatcaaatgtgtttccgtatttctgaccagaaatgcaacttatttcgttagttgtcatttcaagatgccttcagtctgtgtagttgctgcatacctacacctcacacattttcaatccagccctctgaagggctcggttaagtttagataagcagtgacttcagaagaaaatgaagtcattggcagaaaagttgttcccaacacagttgagtgtaggtattcctcaaagatattgactccctttcataaagtatcaagactacccacaaatatttgaagaaatcaacgaaggcttcataggacttttacctaatatagtttgaataattctggaaataacttataggcagaaggataaggagaatggaaagtaattatgaataaactccacagccagggtatattaataaaataattaataaaagaaacatgtctagctagggaattatttgggatcaagaaaccaccaaagtcatcaatgcttaaaagctgacttatttgtctagaggtcatttgaaacccaaacagttcactttgtttatggatttttctggattaagcatatcaatattctattttactttgccattctgatacatcccctttgtgctactctgaggtatttagcttccagagggtgttaaataccgtagcaaggttttaaaatacgtttctaaaatattcctttaactaacagagaacacttgacttagggaaaaaaaaaagaccaaagatgtacataaaacatatcttttaacatctgaaagatgaacacatgacaaaaagaatgtaaatatatattaattacagtctaattgagtaaaaaagtaaggatctcacaaattctgggatctttgttttgatcactgacttatcctcagtacctaaaagcagtgatgggcatatcataaacaataaatatttgtttaataaatgaataaaaccataagatgacatttcattttaacctgaaggtttttaataggcatgttcaaagttagagagctaagctgtctgctgtagtaagatttaaatattagaatagctgaccatttgactaacaaatatttaagtattaaatgagtagctcacaaaaatattatttaaggtaatttgaagcaattgtggaaatcttcaatttttaaaatgctattcctccagcattgacttagacactgacaggtgttattatacagcagaggtagagaatcatgacttccctgtgtggagaacatgttaagggaaagaaacagatagtttaagccctctgaataagacagcattgaatctaaaacttcaggtacaaagagaaaaagacagggtgacagggggtggagggcgggggagagttgctgaggcaaaatggtataagggaaagtgtgcagtttctaatccatacaggtttaagttgattctgatatattcaatttttttttctatagcctagagcaagctatatgttttcctagtttacttatctgtatgatgggaatcataattagtaatatcttaggcggtggtaacatgcattaaatgagataatctacataaaagcacttcacagaatcccagctacagaatgtaatctgcaaatattagcattcgatatcagcactgtaatatgcttgatttgagtaatggtatatacatacttttcagctctcatgccaaaatagcagaaggttgagtggttttccatgaccatctttgaaaattcttctccctcttggttttgggaaagccttgtcttgatgaactgacggaataaatctctcttaaatattacatattatgattttaatatattagccataattatgggtcaatgtttcaataacttgttttgttgagttgaaataataatggaatttataaatacagactacacaggatggaacttttggtcgagtcaaaactttaaatggataacttcattctcttcttccccccactatgcccatttaatgttttttattttcctttttgtgctactgtgaatattgagagataagcccttttcatgtgttgcatcacccaagcatataaaattggttttccccaaatatttgaggttctacaaaattcacattagcatttttataaagtacttttcttttaattaaacacagaaagtatatgctgtagaaaatttatttcacatgtaaataactcatttgtactcaactaaattgtcatttggtacagttttgatattcaaaactttctttaattttatggtgacttttattttaaataaattgtttcttttgttagccagaaattaatctgaatcctcaaggttcaatttcagatgtaaatgtcaaatcccaatgtttgacatcaacctttcaaatttcctccctcactctctacctacttaaaacatttatactatataatatcagattccataccaatcgagattattggacccaagttattaaagaatatactcaacaaaggttgcttatgatgctaccatattatattataataacagccatggtactccattttttttaccttgaccagtcttaaacatgtcttgtgtatcttatgtctccatcaaacacacacacacacacacggacacacatacaacacacactgacaaataaccaaaataaatttataaggaaatgatacctaaagttatcgtaggcaaatgttcctgatgttttttttttttctgttttatctattttttattctacttctatcccttctgtttttatgtaaaatatataatgcttgttgagaattaccaaattggttcaagatctactcattggtcacaacaggcattttaaagtgaatgaaaagcgagaccaataccagaccgcttgcttaacacatattgctaagcagcaaccaatccagttctttccatgcagatagacagctgttattcaagaaacaggaaagcctccttctgaataatggcactcaatagcatgtcagaatgtctgtggctaccagtggcacatttctgtcctaaagaccagactgtcttagaactctgcactcttccagctataagtaagggatagttcagtttatgcctgctctccccaaagttctatccagtttagcccttgttccatgttttcattttaaatatttataattagtaggcattttatgttattattaatagttgcaatagagtggtctgggacgacatgataatacaaagaaataggattctcaataagactcacatcaatctgagattttttatatagcatacagatactcaacaattttaaggatataagcaattgcaaatcactggcacagcaggaatggtccaggtcttccaaggcagcttcccaggtcctttcttgctatatagcagaatgcactctggggtcaaatatgcagtctctaagtgatgtgtgaggtcatatcacgcagaaggaaccattttggtcaggaaacaccttccattcatccttatataattacatatcttctgtaacatttctcaggaagccatatccataaatcaatagtttccaggtttgcttaccataagcaattctagatagataagacacatcctgctaaaagcattccaaagataaggactttccagcaaaaacaacatttttttgtttgccagtagggctacattaccatgtttacaaaggaaatgtttcagggtcaacctctttctctcttgggcatacctgccctgcgtaagagaaaatatagattgcagaccaaaatattaaccatttacttccccagatgtccttgatgggcttccattttttttatatcaggttcttccagagtcttgtctaatgacctgtgagatgtgtgcatcgtaacgggcctctcagtttaacactatgttaaataggaaagcctgcaaaagaaataactgggctctcttttcttgatgttttctaggtttagcattactaatacttctctttcaagaatggctgatgacgtgctctgcaactgaaaggctaggaaaagctaaaaccttctggtcttcagtggggggtggaaaaggtgttcaatgctactacaccttctagctatttggttgtacttgtgctgtgtgacccatgccaactctctgatgatatgcaaaaactgccgtatgtcttgtttttttggtgtcagtgggaaacgatgtcacgtttataagaataaatgctgagcagacctactttagattcatgtatacaacacaaactgtacatcttgctacagatgtttggtcatgtattccatggttacaatctaaaatttatttcattcttcactaaaatttattttgcaattagtttttggtaccatgagacctaaaaactatatatttaattacaacaaatataaacttttttacttctgaagaaaattgatatgtaatttgcctaattttttgtcatcatttaatacatttaaacatgcactaagagatgggggggttggagtagcaggacacagtgacatcacagaacacatgaagacaagattaactgaagaagcattaatgtctaattgaaaagttagtgaatgttttgagattatcttttaaaggtgagaatttagtactactgaagaattcatatttaataatactctgtgaagcagaattttcatttaagatcaaatgatagctttttttaattaattttgctcatttacttatgcatgtacaaattataaaacaattgctattaatttgatggcttcattagcaaaaaaatgagcataaacatttagatgatacaacaaattttatattactttcattagatgtttcaaaagaaaatctatttaattccaaaaattggcatttttcattcaattaattagatcaaataaaccttttagaatttcatttcatttaaagtaaatgtctaatattattgtaaatatgtttctgtaatagttacaaagtttatcattgaagataaaattgtttttcaatgataatataaacacaaaacttagtagtaaattcagcttcatgcataagatattattaataaatgagagaatgttacatgaaggtcctaattatatgttaaaatctgaaatattcaccaatataaatagaaaccatggtgtcaaaattgtacatattttttatctgtatactcacagtgactaaactacagaactaaactacaaatgaagctgatactgaatgcaaaaaatttcagaaaagagtattttgctagaattttctctctgctgttttgtcaacaattagaaaaaatacacatagctgagcctttgagatgttatcttgtaggtcaaagtgcctatgaattattaaattttttatgagtcttctaaattttagtttcattttgttcaaattaagttggaaatttgtaatcaaactatctgcaaaaagagaggtagcaaaaattttataacactctccatttttgaaggtttgagcaaactttcttgaaaacacatttgaaaatagacttttttagaaaactttttcaaaaaatattaaaattttagatttatcaaaaagcaagaaattgaataaattaaataagttctgaaatagtataaattaatcatgaaattctataatcgctttggaatagtttgattcttaggaagaaaagaggttgaggaacttctattttttaaatgtataagttgatcattatcagagaccaccccaatatttcatgtaggttcttttctattttgcctaaggtgtcagccggtctgagaaataaagggaaaagagtacaaaagaaatttttaaagctggttgtccaggagacatcatatgtcagcaggttccatgatgtgccccaagctgccaaaccaacaagttttttaattagtgattttcaaaggggaggagtgtacgaatagggtgtgggtcacagagatcacctgcttcacaaggcaaatgggcacagagcaagatcacaggactggggcaaaattaaaattgctaatgaagtttcaggcacattgtcattgataacatcttatcagaagacagggtttgagagcagacaaccggtctgactaaaatttactaggcaggaatttcctcatcctaataggcctgggagcattacaggagaccggggcttatttcatcccttatcagcagcaactgtataagacagacattcccagagtggccattttagagacctcccctaggacgcattctctttctcagggctgttccttgctgagagaaaaaaattgagtgatatttctcctattcgctttgtaagaagaaatatggctctgttccactaagctctcaggcagtcagacctaacggttatctcccttgttccctgaacatcgctgttatcctgttctttttttccaagatgcccagatttcatattgtttaaacacacatgctttaggaacaatttgtgcagttaacacaatcatcacagggtcctgagtcgacatacatcctcaacaggaagatgacaggattaagagattaaagtaaagaccaagcatgaaatcacaaagagtattgattggggaagtgataaatgtccatgaaatcttcacaatttatgttcagagattgcagtagagacaggtgtaagaaattataaaagtattaatttgaggaactaataaatgtccatgaaatcttcacaatttatgttcttctgccatggcttcagccagtccctccattctgggtccctgacttcctgcaacaaatcatctatactaaaaatgtgtaaaattgatgaaatctgtaatttttcagcatatacttttgacaaacaaaacattcaagagagtcacatatagagaaagcatatataatgaaatcttttttttaaacaggtcaaagagcaagtactgaaaatggtgacaaaagagtatctagaaaaacatttgatctaatattcaacatattcaatataaaacaaaattaaaattgtgatatgttatattaggcagaatttgttgagttgacaaggtacctatgaagaattattttctcaattagaaatattatagtttacaaggtaggatcaattgaaaattcaacaatttcaaaatatgtttatcatttttccaattttatgaaaatattaaaaaatcatgaggtcaaattgaaaaaaaactacatttattataaaaacaaaggaaattagagaaaaatacaaccaagcagttgatcaaataatatgaatatgtatgaaagataagtattctacttgtgttaatttttttaaaagttcaaaaaaatcagtaaagataaaaggatttgggctatagtgtacatgaatacgcaatcttttaaattttttattaataactctattttttaaaaatcaatttaaatagagtgatttaataggcctaccaataaagaacaatttgttggttatctaatatttcaaaacagtagtattaatattttttaaagcccttgttcactctcaaaaatttaccagttggaatatatagaatgtgcttaaatgtgttcatgcagactattctgtgaaaaatatgtttcactgtcataaacacaattatagagctaccttctatatactttttcttgacaattcataatgcagaggagtacagccattgttctggaaagtcctgcagagttatccttattaattttaacatattagctactatacagtgagacaccaagagaaaagctacagtggacattccccaaatatatttatatgcagaagccttaattttctctaaaataaatttaatatcttttaaaacactaataacctacactgcacaactttaggaaattttcttacaggacacaagttacaaaatataaaatttgcatttctcagaagacttctgaaaatagaagtctttgttgtttcgagacatggtctcactctgttgcccaggctagagtgcaatagcacctcaagtgatcactcctgcctgggctgggattgcaggcataaaccactgtatccagcctgaaaacagaaagtcttaacacagcaagattatttatgtaaatgctgtgaatgtaagaatatacaaaggcgagtgttaaaagggatcataaggatatagtttctttgaagccattgaaagaaggctactgtctgctctatttacagaaaaataaactaggatgaaatgagattcttgctcaggaaaacaaggtaaagtttatacacatcaggaaaatctaatcaatgtctgttgtgctttagccttttccttcgaagagagtggtcattataccaaaagtttaggacaaacaccacaaatagaaaattggaataccaggacaggtgagaatatatttactaaatctattaattattgttaccacagttgaaattgctgataaatcaaaacttttgtcggtaaatgttgaagaagagacaaagtttcatttagaaaccataaatttgtaagcaatctgttgagagaaaaaatctgtaatatcatatcaaatatagatggtttgtaaacattaaaaacattaaaattctgccacttattaatgatgacaaaataacttgattttattttaaattattattttattggcagattaatataatataggcattgctaaagttttgttgaacacaagctattctaggcagtttatgatcttctggtgaattatatgataaaatattgacagtattaatgttattttatggattcctctctggtaaaatagtttcctaaaagaatattgataaatttgttgatatttacttaaggaaatactatatttacatgccactgaactctgccatgattttgtttttacgaactttatctttcctgtttggcatttctatcagcaacataaatgaaaatatagatagcaggcttatcatattctcaagcaacacaaaattacaggaaagatcttatgcactggtattcaaaatccacccggccttattaaataccatgttcctcaatatcaaattgtactaaaaaaatccaggaaactaagctcctagtagagaactggtcaaaggaagatcttttgtggagtcagcagtttcctctgtcctaaatttggcaaaataatctcataagcacaagaaggttaatatgcttgcattaaaatgttaagttacttataatccaatttaaatgtaagttcaaataccttaacatgttcacattactttttttaattcacaaaacgttcttatctatctttatttttaagatgttttccacatacatgtgcttagtgaaggtgctggagtcttgtgtcccagttaccaggttaaccttagcaatacgtaactg

chr22:29065832-29065887 polyA chr8:88681304-88690491 ctgcag chr8:88679994-88681299 chr22:29065471-29065782

ctgcag chr8:88679994-88681299 chr22:29065471-29065782 (Primer gap) chr22:29065832-29065887 polyA chr8:88681304-88690491 ctgcag

Consensus sequence for insertion at chr12:128116403

ttggtgtacttcgttacgttgctaaggttaaacagacgactacaaacggaatcgacagcaccttctatggccattttatggaatgacaaactaagttcaatactgatagagttgtattctgtgtatacatataatagagtatatactgtgtgtaaaaaaaattcaagcatatggaaaaacttattttaatccagtaataaacctttatattttctcatcagtttaataataacattttttgccagtaattgggtatattttgggaaatagtagaataaagatttgtagactctcttacatctctaaaaagaaattcactaagcatgtatgtggaaaacatcttaaaataaagatagataagaacgtaccccagctcaggggccaaaccagactgaaactcagagccaaagttaatattccatgtctacttgatggttatccctgttccactattgggctgtatttgttcatggcttagatgtgtgtatgcgtgtatgtgtgtgtgtgtgtgtgtgtgtgtgtgtgtatgtatgtgggtgtctatgtatgtgtgtatatatggtgtgtatgtgtgtatgtcttggatgtttgcatatggtaactgtatgtgtactgtgtgtgtgtgtggttgtatgtgcatttatgtattatgtatggcatgtgcttgtgtgcttggatgagtgtctgatgtgtattatatatttatgtgtgtatgcacatgcatttgtatgtttgtatgtgcatatatgtttgtgtgtttaagtgttgcatgtgtgcatatcgagggagaaatttatatgggtccaagttccctggatgctgatgctgtggaacaggaagacctactcagacataagaaaaagtgcaaaatgaccctggattctgttttgctccctgaaggtttctgtagatgtgtcagctgggagactggtgcgtgcacttgccaagcccacagactctcagccatctctgcgtctcccaaaactgccagcatcacctttgacattttagcaaatgttattccatactctcaggatgaaaagagaagtcttggcaacccacggtttgaatacctggatgtgtctcagtagttcccctaagccaagcctgctctttcgccaaacccacaggcagccatgagtaaatggagtagtgcgtctctgtgcagctaagcctgtggagcagcgtgcttgcatgcttcaaagagtcgatagaataggaggcaaaatgctctcccatgccctgagcctttcttccctatcgacattacttcctacaactagaaaaaaacaccgttttcactaatagcttcatcctgttcttttctacgggaaaacaggagagccaagctgctggccaccgagagttttcccagatctcagcatgtatgtatagcttttagtataattggattcaaagaaagaaggaacagagacattttgaaataagattcagaaaagaaaaacactttgtgtttgggagtttatttacaaaatgcaaacactgcattgaaaatattttgatattgaagttcttaacagttactgaaaatttctttatgtatttagataatttatatgggttatggtgggagatgtttgttttttagaaaaaataaaatatatagaaaagtgtagaaaatagattaatgtaaacccctccccttgctggaaaccagcgggacactaatactttcttgacggaattaacaacccaggaggttttcaaacccatcagggacatattcctctcaccctaagaaggctgggatgttatccatagaaaaacaagccaatcttcgtgttttaggagagcttgaaaatatctctggctttaactgttgctttgggttatttatttagatgaaatattttaattttattttaggttcaggatcatctctttagacactgcagttcattggattgtgattcttcattttttctcaggtttgccattgactttcatcaggttaatataattttattaatgtcttttcttctctaaatgtttcttgccaattagaaaccatgggttatatttaccttgttttcctcaaacagaatatgttgcactccgaagaacataaaccaatatggaaaaatgaacatgaattattagattctgagatgcagcttttttggtgggtcctaagatatttgcctgaatcaagtaggatcttagtggatgaggtatccaaacttttgatctggaaaaacaatttcatcaaaatctggcataaagtccttccttttctccaggatgcagtggttcagatcttgaggcagatactagaactctgacatcatctcagaattctttcatttgcaaataatgcttcaggtacagaagtccaatgcttattgaagcatttacaaaatgctttgggtgcagaagtccaaatgcttcaataagaccatgcagcttaaaagatttggtatgtgagatgtcagttcctgtgctctagatcagtggtccctaatcttttgggaaccagacaacttttcatggacttgggattctggattcgatgcattcccatctatgtgttttagcttctgttgtctgtacttttagagtcctattcaaaaaatcattgcccaggcccatgtaaagaactttgttgactatacttcaataaagatgggaataaaataaaacacaatacaataaataaaataaactaagaagaagaagaaacaaataaaaaatgcaggctggacccatcagagttaaccaagtccattagcagtagggagaataattgcatcccatctcgacacgcattcaaactatccttttctcttcattttaatagcctcagtcctccccttctctgtctgtgtaagcatagcatatgtgccttcccatctaaacagccatttccccagttaaaaccacacttgactaatataacacatattctctactttacaaagtactaatttaaaaggatagtggaacattatgctaaaatattgactatagagttccatttatgtaatggatacagcatactgaaacactaaaatgtatattggaacaaggatgctttcaagtaaagcatgcaaaggtgtagattttaacgcacgaccatcgaactctgaaatatttaaatgaaaggggaggaggcatttcgagagacaaaggtaaaatgcactgcatttaaatatctggccacgtttgggatggttgctggtatcattggcatgttctagtctctctgttttccttgtgattttatttttcctggacttacttcagcagtgaaaagagttcatattcagcataagacatctttccaccacagggtgtcatatattgaaacatgaaaataaacagtgaaacattcttcaattcggggtgcaattattttattacacacacttatctcagcattgtaattttagccattcacaagcacattatgatatgggacagagccctgtccttgtcctcttttcccaaacttaggacttggtagaaaggatcagagtccacatttaggcattcagatggaaagaaacggttcattctgttgcatgtgctggttgccttactaggtttcatgcattcttcaggtttctctgtggggttacatacatacaccaagtagaactatgtaaaaaaaatagaggcggcctcagtggttgcatgcaggctttcggaggtgaataagctgatcctgcacttctagttacccagctgcttggtgtgcgggtggtagcaaacatctctatgatttggcaatgtttccatcaaataatttgaagttgatgattttaataaatctgaaagataaatatgaacttaaaaaagtgttaaaagcatgtagaattttaccttcaagagcattttatttattctcatagtagccaataaaatggtatttgcagatgcatatcccatagtgttaaagaaaggaagaattagagtatttctttttttttttttttttttttttttttttttttttttttttttttttttttttttaaagatattaaagtctttattgccccccattttttattttcagtaatacagggtaaaatgagtaggattacttgatctggcaggtgctgtcgattccgtttgtagtcgtctgtttaaccttagcaatacgtaactt

chr22:29065730-29066032(-) chr12:128116405-128117970 ctgcag chr12:128114360-128116403 polyT chr22:29066035-29066121(-)

ctgcag chr12:128114360-128116403 polyT chr22: 29066121-29066035(-) (Primer gap) chr22: 29066032-29065730(-) chr12:128116405-128117970 ctgcag

Consensus sequence for insertion at chr2:50947578

ctggtatacttcgttcagttacgtattgctaaggttaatagggaaacacgatagaatccgaacagcaccttctatggccatttatggaatgacaaactagttcaatactgatagagttgtattctgtgtatacatataatagagtatatactgtgtgtataaaaattcaaagcatatggaaaaacttatttaatcagtaataaacctttatattttctcatcagtttaataataacatttttttgccagtaactgctacttttatgccacatactgttggtatggtagctaggaaaaaaaatgcagtgcctgaaacaggtatttgcattcctatctacatcaaagggctgaatttttctttcatgggacttagtgttttccagaacacaaaagatccatgtagccacaatattacattttgcatttataaaccactcttcccagtttctctgagatctcctgcatgtccaaagacactcaagcacttcaccaaagattctctgttcctgttatcaagtattttaatacaagataacaattacagtttaagagaaatgctagaaaaaatgcacggaaagctcccaggtgccacttaactctccttccttgctgcattccctctcccacccatcactgaaacctgagccagccaattagatgaacagcttgtcactcctagatgcagtgcaatgtctgcatcttactgcagctgttaggactgacaattcaattcagtctgcaatcctacaacaaagactctctcgtctggttcctgggagtcagagattctccttgttcatctttgaacctcaaaaacaatagtttatcctggagcactcagaaggcttaaatctgtataaatagatagaaacatggaaattgtgaccctagtttagccactagatgactcagctggagtggactcttcggcttccaaggacagctcacatagctgctctctcagaatataccttaaattcccaatcacagagcagggtgccctaagaaccattcaatttcctaggcctgtgaacagcagatttttttgcttggcaggttggataatctacaacactctttcatatgtctgtcctgttataaaatcagctctagtttgtactgggctctagaaagcattagagctaagaacaggatcctgaagcatgtatttcagagataactaccctttactaacaacaaatatgtagcatacagattcctctgaggtgcagtaaccacttttgtatttatagttattaaagaagggaaaacaagaaatcaagggccacctcatttctcatataatgtgttctcctttgtgcctgacagacagacgaggactacatgaagcatgaatcctcaggatacccagtcactaattttttaaaaagtggaatgagtcctaagtgtcaaggggttttaagtaaagtgaaaattaaaaacagttaaaaggaagttatacttaccacttgctgtatttactggaccttgtggtatgttttttttcatacattattttattttacttcataaaaccaattaatttttcctcataaacatttgggatagatattttttactcatattttacaggtagagaaagttgatatacattaatatgggtgttagtatctgagagagtttaaacaacttcccgaaactagtgagtagcagagctgaaattaaaaacctcaactactacacacagaattatttaacagagttgaattgtgattttttgcatttctcgggaaatgtccttggtgaaaactctgagaagagaaaaaatgtctgaatttcttctgcaggaggtctctaggctcccactgctctttaaaatggtgatcataatataaagggatatacgtccaatgaatctagtattccatagactctatcaatcttttgcccaatctttgctattcttcaaaattttctgaagctataatttgggcatctcctatcatttactgcattatttctaggctagatgttagtggcaaaataattaaaaaaaatatggaaccccaaaattgagggtcttcacagcaaaaggaggtgataaacatacaattccccataatacaattaattctgatatatcagcactatgagcagagtactaagagatcataaagggaagaagtaatagcaaattgaaaacaatctaaatcagcaataaaatattggtcaggttaattatgatatatccacacaatggaataccacatagccattaaaatcatattgcagcagaatatttaatgatttggggatacgtttcatatacattgtttagtagacaagcaggctgtaaaataaaagcaatataatttcatttaaaaatatattggaagagtgagaacactttagcaaatgttaataatggttatattaatataaaaggttaccattattgcttctgcttttgctttttttatgttttgtatagtgaatatgcttttcttttgtaattaagaaaaacattcaatacagtttaaaactcaaatcatgttaacaaaagacttaacaacaacaaaatcaatttgaaattctttcagattgggaaaagtaaaattctaacacacttaagatttatttccacagatggcctcatgagctacaaatacctcactcataaatcgtcttgccttgaatttccactcagtgttctgatatcatgccagatgtgggatcttgccttggtccaactcctcctttgtaattttaaggcctctcacacttgggatgctgagctagagtcattctcctgcttggtagcttttgagagtgaacagatcacttcgctgtgtattgtcattcagttagtagatgtttctggctgcttcttcaacccttacaatatacctttcctaccaagaaatgtgtgacttctattctctcagccttggtgtaaatcctgaaagtgttaaggagaaaaaaaagagcaagaatcctccaagatctataattgcacaggccccaggaaagaagcataaagaaaacagtaactaaaacaaatgcagaataagacctgaaatacaatatttgaccacagtctaaaagtaaaagcttagaaactggtcaaggatgtcatgtcttgtcggttttgaagaactattattcaaagctttccatttaattatggccaaaatgcccaggtctgtttttctcaaaaatccagtgttctgatgactagctacattacccatgcatggtggcatacgcctgtaatcccagctacttgggaggctgaggcaggagaatcgcttgaaccgggaggcggaggttgcagtgagccaagatcacaccattgcactccagcccaggcgacagtgcaagactctgtctaaaaaaacaaaaacaacaacaacaacaataacaacaatcatttttcattggatttattcttgattgggattattagcataaagtatctgagaaattgcacataactcaatttctaggtctttatctctctcatcctgaaaggcttccttagaccaactttgccctacttaaatgctttccttatttcaagaccctgttcattcagtggctttcctggaatctctctgtcatcccaaatacactgcttggagcaacagaaaaggtaaagctagagagagccatcaactatgcccacagtagccctcagacgtgtacaaatacacacagatacatgtttaaagaacaaattacaaaactggtcaatcattaaactcagggtaacacattttcagcctgcacaagatacctagatgtttggctcatgcctttgctgtagatctgtccagcaagtggtctgattttggatcagaacatgcaatttctgtataactgacacacagatatatatattgtctttcaactttttttttttaaccacatcttgaaccttggaggttatgggaaacttcattttcagaaactccacaagtaaaatggtgcaaagtcatataaaaccagttctggcaatgtctctttcagttttatcctcctcaatgtaagctaaatcaatacattaacaaaggtcgggctgcatcttcagttaatttggactctttcctatttgcttccatctgtgaacaaacaaacatcagtcaataagggcgccccaacgtgcttctagctttttttttttttttaaagatgttaaagtctttgttgcccccattttattttcagtaatgagataaaatgggtgggattagcgatctggcgggtgctgttcagttctatcgttttttcctaatattaatgcgatgtagtggctt

chr22: 29066032-29065849(-) chr2:50947612-50950576 atgcat chr2:50946657-50947578 (Unmapped)

atgcat chr2:50946657-50947578 (Unmapped) chr22: 29066032-29065849(-) chr2:50947612-50950576 atgcat

Consensus sequence for insertion at chr2:129889238

ttggtattgcttcgttcagttacgtattgctaaggttaaacagacgactacaaacggaatcgacagcaccttctatggccattttatggaatgacaaactaagttcaatactgatagagttgtgtattctgtgtatacatataatagagtatactgtgttgcagtgaggaaaaactgtaatgtgggtacaaagttttagtgttcaggaaagattggctttgaccttaaaagaaagcagaggcattgggatttttctctcaaactatgttagctgtgttgaccattattaaaattaagtggcattcatttggattaagtagtaacaaaatgtgagactttctagtgattttgatcccaggctgtcccacagttgggccttcatgtgtgtactggaaaacaaactatgcacaagtgtgttgcactggtttgaagattctagtggtgaaagttacctaatcagttgtcagtactatatctagaaaccaatcttagaaatattttaaaaggataatactcttaaaatagctgaaaagaaattactgtgtgcttactttcactttgttcctgttttgctacataatatttaaatgaaaggagattatttatcctcatactgaatttccaagagtgatatttacttttaccagttttaaatggtgaagaaaaaaaattaattgtcatactttcataagtttggcatagggcctatacatatttgatgcttttgatcacagttctgtcactagaatgctagcaattagatatatgcaaggagtaacctaatcactttaaaaacagtgatttgaagtgctgcagcagttaggtgtgttctcgtgaatgtttgtgtgttgatatggtcacgggaaattagaattcaataaactgttattcttttgtaatactgtttggccccagattcagtggaatctgggtttgctattgaatgggaaagttggatggagttttatgtacacaggctgttatgctgttattctaagcaagatgaggcctgtgtagtatatgtaatattcttctgtagtactgtttagccctggggtggtttggcctttaaaaataaaaccgccctggaaactactttacccaaaattttcgttcacagccttcactgtatttttttacctatcaaggcaaacaaagtatagccctgtgaacatgttcatagactgatgagtttgtgttgctatctcatggctagagttccatggtaaaagctattagatctttatgtgtgtgaatacatagatatgtttatgtatatgtacatttattatgttatatgttgcacttaccaaattggcttataaatagagtgcccataaattaagtccaaaatagttttcaagttcacatgacttagtaaaatatttaataaacaagctggctttaaaattattggtaaaataaaagtagaatgtctcagaattgtcagcatacatttttttatctaggttttgtatttgtttctgatagatattttgaggtgtcagggtttggctcagaaggttataaaactataaaccagccaaaacagaacggtctttgtctgtgaaaacttttgataaataagactaatttaatattgttggttttaatgaaaacagctgaatcttctgagttattggcgcaaatacctgtacagttaactttaagtttcttacttaggtgaacatttgatattcacaggttagaaagtggttaacaaaagaaaatgatgcataagtaatataaactgctaaaaataaataaaatgtaactaggtaggtaagtgctgggtgaactttatatttcatttaaaatcttaaaatcattttttgatgctcactggatgtctgagtcattttccaattaagaaaaggctatgatatggggaaacatgtttctaaaaatttttttggaatggttctcatctataaaatgctaatatctcataggcagctcaggatttcttgcatcctaggttttcaccaaaatttaggttactagggataaaaattctagttaatatctaattctctattaaaatgtgccaaagaagatgtgttttttattgaaaaaaatattttctaattcagaagttatctgaaggttaactccaattataaacttgaaaaggttagttacttatgaaacaaggcagacaggaaccagcaagtagaggaggagatgtgaagaaagttatggatatgaagatgtatctttggtaatgaaggctctaaagaaaagagaataattttgtataagaatggatcttgtatgtatttttttgtcctaaagtaaaatgactggttagttttaaaaaaatttagaggcaaaagcaaaatcaaagtcataaacggtctgtgtaagtcaaatgtagttttattttcctgcttctctatgtgtcatattcatgcacatacagagaaaatagaacactgaaaaagtttaagtaataaaatattctttacaacctgatagaagaatttgagaaatttgactaattaacactgctcattgctaaagttcttagtcttgatgaagataaaataaaatatttgtaaaagaaatgcattagctgtttggcagttcttgtttaatatagttaagcatgaagccagatttagcatggggccaaatttttacatcaaacttgcttttttttaccatacttgttcttctgcatagatagtactagcattaaagcacttactggtcataatacctaaattgatttctcaattgcacaaaatatataagtggtatttgaggacttaagaccttaaattgtatatcaggaacaaaatatccatcatgtgtttttttaatattctgggtaacactgtagcctcaaagtaaactgagtagagaaaaaatttgttttcgttttttttttttttttttgcttctaacttgtatttattagctgttttgttctttgggttttacttatatatacatatataaccattgatgattttcgttttcaatggaaggcttctatttggttctatgaatagtcattttgtttctcatgcatttcaacaattcatcatttgttctgtttatctaaatttcctaagctacctttgtcaagctttcaaattgataatgacctaaaccatttaaattttgattggttttgcttacctctgatgatcttaagctataagagcttgaaggttctaaatgaaaaattacataaaacacttacatttaaaagttctgaacagaaatagtacattatatgttttgttatttggaaaagtaggtgatagttaaatggtgtttgtttcaaggtaattcaattcaatcaattatttgagtttgtttcacgtcttttttttttttttttttttttttttttttttttttttttttttttttttttttttttttttttttaaagatattaaagtctttattgccccccccatttttattttcagtaatacagaattaaaatgaggtgggattacttgatcagcaggtgctgtcgattccgtttatgatcgtctgtttaacctgtagcaatacgtaacccc

chr22: 29066032-29065945(-)chr2:129889240-129889895 ctgcag chr2:129886718-129889238 polyT chr22: 29066121-29066039(-)

ctgcag chr2:129886718-129889238 polyT chr22: 29066121-29066039(-) (Primer gap) chr22: 29066032-29065945(-)chr2:129889240-129889895 ctgcag

Consensus sequence for insertion at chr4:44621421

cttgttgtacttcgttacgtattgctaaggttaataggaaacacgatagaatcgaacagcacctcagtcacctttattagacatgtggccccatgtgaagtttcttttctttaatagacatgttaattagcacagaattttcttaagttcttggtctttctatttgggctgagacagttctactgacatccttcatggtgaagcaagaattccatttatttttttttttttaataaaaaaaaaaaaaaaataaaattcttgcttccaatctcaaaaataatctcaaccaagaaaaagaaacaaaagaaaaattaactctttattatctaataatcattaatagaccgtaacagtctaacgtttactacaatcctgccttcttttcaacatactcctaccttagaattcttctcatacttttgtaattatctcatattaattgattgcatattgtttatttttgtccactgccaatgtatttccattggggctgcaaccatcacctatcttattcaccatagtgaacaatgcccaatcacagtctcattcgtttgtttattcattaattgaataaataaatatttatctctattccttatcatgaactgttgcatatagacaaggtctttggcctcagagtgtttattttctgcggagttggggtaggatagaaagcaagtatcaaataaataacataatttcacataattgtaacagctactgcagatacatttcacaagtatataataaatttaaaaaggaaaatatcttcattccactcaaatccatacaatgtatacacctgttggaattggaacagacaatattttgtgtgaaatgttttatgcaaatgctaactagatataaatgtgcatcattcattttaaattacagttcatacatggacccaggagtcatacaggtaccactataatccacttagacattctggattcttctatcttattacagtattatttttcccttgtaggtaaaagcccattggccctgtctagtattggccaagatgaaaagaaaggataaaccctagtagaatgaaaagtagacacactactgcttgagttacagacagtgttatgtagtgattacgggctctggaatcaaacttcctttattcaaatcctagtcgttggctgtgtaacctttggcaagtgatttagcctctctgtaactcagtagcctcacctattaaatggaggtgatgataattgcacttcataaaattacagtgaggattaaacatgagttaataaatgtaaagcatctaggccaactcatgagtaaatcctcagtaaatatcaaaattgtgaaaacttactgcatgcattttctcttgtcatgattcttccaaaacatttgttagttaccaaagcattcaattgcagagacgaacaataatctacttaactaacaattgtaataaatttttagattttttccaattcttcactgttatatatttgcctaatttttcagattaaccttagaatcaatttgtcaactttgtaaaaaaatgcctcttggattttaactgagattatattaaacatacacatttatttggggagattctcttatgaagtccaatcttctcatccaggggatatagtttatctttctgtaaatttaaacctccttaaaataaacctctaatgttttgcaattattttcatataaatcatctgtatttctttgtacatatattctttggtatttcacaactcagtcattaggaactattaaatagaatgtattcttatttcttactgttatggaaatatttagtagtttatgccatttttttaatatatagaggctataatttttcatagtaaagaatatcaaacctttctgtgtatgactctgttttttatacatagaaaaacatcaacttacatcctgaatttagagcaattttttagtttatcttttttaaaagtaaaagcactttcaaaagaggaaaaaaaaaattaggtccataaggccgggcgcggtggcttacgcctgtaatcccagcactttgggagaccaaggcgggcggatcaggacttcaggagatcgagaccatcctgcctaacatggtgaaaccctgtctctactaaaatacaaaaaaaattagccggacgtggtggcgggcacctgtagtcccagctactcaggaggctgtggcaggagaatggcgtgaacccaggagatgaaacttgcagtgagccgagatcgcgccactgcactcagcctgagtgacagagcgagactctgcctcaaaaaaaaaaaaaaaaaaaaaaaaaaaaatccataatataagtatacttctaagcaaaggggtgattatagaaatcattacaataaatggaattctttttttttttttttttttttttttttttttttttttttttttttttttttttttttttttttttaatcagtaataaacctttatattttctcatcagtttaataataacatttttttgccagtaattgggtatattttggaaatagtagaataaaagatttgtagactctcttacatctcctaaagaaaattcactaagcatgtatgtggaaaacatcttaaaataaagatagataagaacgtttttgtgaattaaaaaaaaaatgtgtgaacatgttaaggtatttgaacttacatttaaattggattataagtaacttaacattttttaatgcaagcatattaaccttcttgtgcttatgagattattttgccaaattgtaggacagaggaaactgctgactccacaaagatcttccctttgaccagttctctactaggagcttagtttcctgatttttagtacaatttgatattgaggaacatatatttaataaggccgggtacttagagtataataattgaattaaattgtaccttttattttggccctaactggtcacacttctgggaaggtgctgttcggattctatcgtgtttcctattaaccttagcaatacgtaactga

chr22: 29065354-29065305(-)chr4: 44621515-44621406(-)chr4:44621524-44621912 ctgcag chr4:44619750-44621421 polyT chr22: 29065912-29065404(-)

ctgcag chr4:44619750-44621421 polyT chr22: 29065912-29065404(-) (Primer gap) chr22: 29065354-29065305(-)chr4: 44621515-44621406(-)chr4:44621524-44621912 ctgcag

Consensus sequence for insertion at chr5:8665955

Ctggtgtgcttcgttcagttacgtattgctaagttaatagggaaacacgatagaatcgaacagcaccttctatggccattttatggaatgacaaactaagttcaatactgatagagttgtattctgtgtatacatataatagagtatatactgtgtgtataaaaaattcaagcatatggaaaaacttatttaatccagtaataaacctttatattttctcatcagtttaataataacattttttgccagtaacaagactgtattcttacaaatacctgtttgcaagcttggttctggctggcatctaaggaacttggctatattataaagaatttcctacactgatataaaacttctcttaaatgataagagtagatcattgtgcctgggtgtgggtgaaaataatatgctctatgccaagcaactcttttctgttgcttggggctgttatgagaatcatcaaccctgcgtctttcaagtctttaatgatggcaccattctgtgcaatatttcaggaaaatgctgtgttgtttttttgcatttgctattttcttaggtagaggcagtgctaatggcttccttttgcctttccaccataataataagtcccatttcacaggtcagggaacaggaactctgccagcttcagtgtctattctggtatgcatcctgagctttctcaccatgttctggctagtcttaaattctatgctcattcatttcactcaccaagttgtgcctccattgctggatattcacactcagttgtcatccttaccttgagctcagtaagactgtattcttttttttttttttttttttttttttttttttttttttttttttttttttttttttttttttttttttttttttttttaaagataataaaatctttgttgcccccatttttattttcagtaatacagattaaaatgggtgggattatgatctggcgggtgctgttcggagttctatcgtgtttcctattaaccttaatgcgtaact

RC:

Agttacgcattaaggttaataggaaacacgatagaactccgaacagcacccgccagatcataatcccacccattttaatctgtattactgaaaataaaaatgggggcaacaaagattttattatctttaaaaaaaaaaaaaaaaaaaaaaaaaaaaaaaaaaaaaaaaaaaaaaaaaaaaaaaaaaaaaaaaaaaaaaaaaaaaaagaatacagtcttactgagctcaaggtaaggatgacaactgagtgtgaatatccagcaatggaggcacaacttggtgagtgaaatgaatgagcatagaatttaagactagccagaacatggtgagaaagctcaggatgcataccagaatagacactgaagctggcagagttcctgttccctgacctgtgaaatgggacttattattatggtggaaaggcaaaaggaagccattagcactgcctctacctaagaaaatagcaaatgcaaaaaaacaacacagcattttcctgaaatattgcacagaatggtgccatcattaaagacttgaaagacgcagggttgatgattctcataacagccccaagcaacagaaaagagttgcttggcatagagcatattattttcacccacacccaggcacaatgatctactcttatcatttaagagaagttttatatcagtgtaggaaattctttataatatagccaagttccttagatgccagccagaaccaagcttgcaaacaggtatttgtaagaatacagtcttgttactggcaaaaaatgttattattaaactgatgagaaaatataaaggtttattactggattaaataagtttttccatatgcttgaattttttatacacacagtatatactctattatatgtatacacagaatacaactctatcagtattgaacttagtttgtcattccataaaatggccatagaaggtgctgttcgattctatcgtgtttccctattaacttagcaatacgtaactgaacgaagcacaccag

chr22:29066035-29066091 polyA chr5:8665942-8666085 atgcat chr5:8665543-8665955 chr22:29065849-29066032

atgcat chr5:8665543-8665955 chr22:29065849-29066032 (Primer gap) chr22:29066035-29066091 polyA chr5:8665942-8666085 atgcat

Consensus sequence for insertion at chr5:83347372 (Twin priming)

cttgtacttcgttacgttacgcattgctaggttaacctggtaactgggacacaagactccagcaccttctatggccattttatggaatgacaaactaagttcaatactgatagagttgtattctgtgtatacatataatagagtatatactgtgtgtataaaaaattcaagcatatggaaaaacttatttaatcagtaatccacatacatgtagtgaattttcttttaggagatgtaagagagtctacaaatctttattctactatttcccaaaatatacccaattactggcaaaaaaaatgttattattaaactggttttagcttctttgcaatgggttcgaacttcctcctttagttcggagaagtttgatcatctgaagccttcttctctcaactcgtcaaagtcgttctctgtccagctttgtttccgttgctggtgaggagctgcgttcctttgcaggaggagaggtgctctgattttttaaattttcaattttttctattctgtttttccccatctttgtggttttatctacctttggtctttgatgatggtgacatatggggttttggtgtggatgtcctttctgtttgttagttttcgcttttaacattcaggaccctcagctgcaggtctgttggagtttgctggaggtctgctcagaccctgtttgcctgggtatcagcagcggaggctgcagaacagtggatattggtgaacagcaaatgttgctgtctgatcgttcctctggaggttttgtctcagaggggtacccagctgtgtggtgtcagtctgcccctactgggggggtgcctcccagataggctactcggggtcagcaacccacttgaggaagcagtctgtcccgttctcagatctcaggagtccatgctgggagaaccactactctcttcaaaagctgtcagacagggacatttaggtctgcagaggtttctactgccttttgttcggctatgccctgccccccccagaggtggagtctacagaggcaggcaggcctccttgagctgcggtgggcttcacccagttcaagcttcctggctgctttgtttaccttctcaagcctcagcaatgggaacacccctccccagcctcactgctgccttgcagtttgatctcaggctgctgggctagcaatgagcgaggctctgtgggcgtgggaccctctgagccaggcgcaggatataatctcctggtgtgccgtttgcacaagaccactggaaaacacaatatggggtggggtgaccggattttccaggtgccgtctgtcacagctttgcttggctatgaaaggggaattcctgaccccttgcacttcccaggtgaggcgatgcctcgccctgcttcaactcacgctcagtgctgcaccctgtgtctgtacccactgtctgacactccccagtgagatgaacctggtacctcagttggaaatgcagaaatcactcgtcttctgtgtcgctcacactgggagctgtagactggagctgttcctattctgccatgttggaactgcctcaaatctcatttttaacattctattgttttttattactctaatgtttcatctactcacttctataagatcaaaatgtctggaacttctcaagcaaatattcttgtgtgatttttttttttttttttttttttttgagatggagttcagctctgtcgcccaggctggagtgcagtggcgccatctcgactcactgcaagctccgcctcccgggttcacaccattctcctgcctcagcctcccatgtagctgggactacaggcgtgcgccaccatgcccggctaattttgtgtattttagtagagacggggtttcaccgtgttagccaggatggtctcgatctcctgacctcatgatccacccgtctcggcctcccaaagtgctgggattacaggcgtgagccaccgcgcccggccgtgtgatttttttttaagaggtgatcctaccctggtggttagctttcttgagttgctgctaatcataaaattgttatgtctatttctatcgggacatggcaaaaatgtgtgagggcctcaggcagccctaaacagaggcatttctcagacctctgtgtatgatggtttgtaaagactttaaaattcaactaatactttgagcaataaaacaccaatgtgttatctgaaagcctcccaaaggtcaaattttagttttaaactttcaaaattcttaccaatgttgaaattggaacattctgagccattattccatactcctaattggacattcctaactcatggttaggatagatattaatctttgttataaaaaaaaagtaacatttaactcacagagagatgtttttaggttaagactattagctccataggtagtttccaaacaaagaaagtcagaaattgtgatgactcttaaagtggattaaataaaacaaataacagaacagtggaagcatgcagtaggtactcagtattggatagatcccttcttttcctttctggtggattgtgtacctcaggagaacaatttgacaccaccaattttaatcaaaagtgaagatttgcatatatattcctcaaaatggaattttcaataaaataatgaaatgtaaaataatacattgttaaatcattgcaaactgtaggttgtgttgataaattatatatattggcattggcacataaaaactttgaaatgtctatatctaggtagcatcagatttttcagcttctttacccagtagtaataatttaaaagtgagtaatttatcatttgccaatcttctctactgaaactgtattttttttactaaaagcagttatgtgtttttgtgtctgttatcactggctttcaacagaaagattaatttcttttttacttctgctcagattagacaaattgaaattaaacattgttaaagatgcttaaagacattctatgaggagcagggacattgctaatgatcatgctattaattgtgaagaaatatgctgattcattttggttttgttcacggaactaaacaaactgcactcaaaagattaacttggattaactaaaaacttattttagtaaaaaaatactacaaaattttgttataatgaatacattgtgtatgtattcattgccgtattagtggtgactatagaaaatggataatttactattcataattaaatctactaaacgttttcagatttcataaaaggtaacataatataaaggactaacaggaaggaaaatgtcacaatcccacctaagaaatgatcttttggtctatttcataagacatttgctaatttaaggtagtctgcatagtatttcaaccaaattagtaagattaatagagttgttagactgaacattttagtgttgacctttatcaaaattgcgtctgaccctacagattttcatttcaatactaattttgtgaaaatgtataagaaatcaattgaatttaagctttcaaattaaataagccttatttaatttagtccatatcatcagattttaaaagaaattattctagacataaaatttataactgttaaagcattaacttattttgccttctgaacaatttatttacaggagaactaaaggaaaggttttcaaaaggttaagtgaccaccaggccactgaaacatttgtcaatatgaaggattattctgatttccaaatcacagttccaatatttctcatttatgaaggttcacctataaagaagatctaaaagaggtcaagtaatgaaacttgtatatgattgtgagacagcttccgttcttctatagtattgacatatgttggagtgtgctaagtgaatcgaaggatgctcacactttcctaggactataagtcatctttatgtatttttcattttttaaaaccacataccatgaataagaaatcttttttgattttataattttatgatgtcaaaggaatcccactatccaattagaaaaaaagaacttttataaacaaaacaaaattgaaagggacatttgtacagaaaatacttaagtgcaaacaaaagtgcagccaatatcaatgtagtataaggagtgcataatttgaggtacaaaatatagagatgagagggctgacaaattatatcaatactctgagacttcaatattctttctttcttttttatgtaaaatggaatatactaaaaaaaagatcagccccaacctatcttctccttttaaaattcctcacacaagtaaaattctggtgtctaaatcatgactggacgaatcaaagaaatcagaaaattatcaagacaccagctaatttcttcacaaagtggcacaaagtatgaagctaataaaagtcatgtaacaattattgatacaagatatagccatctaaccttcaaatagacctataaaaagttaacttttatacaatgaataacacatctacttaatactcaaaatattgcccctaccttttcatcccttctaattttgagtcatttatttggtttagtatcttacatctctctaaaaacagcttagaaaacaaaaactccataagacaaggaagtaagatatatagtaagtgattattaaatacttacttaatatttaataagtaagtgcttgcatatagaaaatcatgaaaaaggcccaaagtaatagtttctggtgccaaagagaagaaatagattaaccccagatacaggtacaatctcagaaaagacaagctctaattaatttgcatttaaaaatgcatggaacctttatggtttggaggttattttaaatgactttatactttttttgaattaaaataagtatatttggggcacctgttataacaaaacacaatgtcaggtgcaaggaactgaagacatattcagtattatgtctgcctgcatgtatgattactgatgccataattgtcattattcaaaattcaaagcaagaaacagaagtgtaggatgttgaatgcttctgcttaaattgattattgaaaccattgaaaaaccgacagaaattaaaaagaatttcatgtgcacagcagacctgctcctgttttatgataatgcaaattagatgacttctgattcctgtgtgtgtgcaaatgaattttcgtttgtggcgtattagttatctttgtagcaacaaagcatagctgtaagaatggtaaccattctcttctaaaaatatgaactgaaaatatgccaatgagtaaaatttagaacttgggaatactgtttcaaatattatgagcatttgtaaaggcttttaaataaaattagaatcaattgtaatcagtaatctcattttgtctttaaagcagccctctccgtgagctaagttatgtttgcttttataatgaagagtcagagaagcttgttaagattgcagagccagtcagtggtctaaaggggagttcagcacttgtcaggaagcctatgagatgattttaccacagtattttctagactattataatgattggaagtgactcattttttttcggggatatctgttggattaggaagcctctccaatgcccagtcctgaccagcatagctctggtcacttatcttgtcttgaataagagctcttgtaaggcaggcctgatggtgacaaaatctctcagcatttgcttgtctgtaagattttatttctccttcacttatgaagcttagtttggctgatatgaaattctgggttgaaaattcttttctttaagaatgttaaatattatttactctcttctggcttgtagagtttctgccgagatccgctgttagtctgatgggcttccctttgtgggtaaccccacctttctctctggctgcccttaacgtttttccttcatttcatctttggtgaatctgacaattatgtgtcttgtagttgctcttctcgaggagtatctttgtggcattctctgtatttcctgaatttgaatgttggtctgctttgctaggttggggaagttctcctggatgatatcctgcagactgttttccaacttggttccattctccccgtcactttcaggtacaccaatcagacgtagatttggtcttttcacatagtccatatttcttgggctttgttcgtttcttttattctttttctctaaacttctcttctccttcatttcattcatttgatcttcagtcactgatacctttcttccagttgatcaaatcagctagtgaagcttgtgcatttaatcacgtagttctcatgccatggtattcagctccatcaggtctttaaggacttctttgcgttagttattctagttagccatttgtttaatcattttcaaggttttagcttcttttttttttttttttttttttttttttttttttttttttttttttttttttttttttttttttttttttttttttaaagatattaaagtctttatttgccccatttttattttcagtaatacagggaattaaaatggtaggattacttgatctggcaggtgctggagtcttgtgtcccagttaccgttaaccttagcaatacgtaacca

RC:

tggttacgtattgctaaggttaacggtaactgggacacaagactccagcacctgccagatcaagtaatcctaccattttaattccctgtattactgaaaataaaaatggggcaaataaagactttaatatctttaaaaaaaaaaaaaaaaaaaaaaaaaaaaaaaaaaaaaaaaaaaaaaaaaaaaaaaaaaaaaaaaaaaaaaaaaaaagaagctaaaaccttgaaaatgattaaacaaatggctaactagaataactaacgcaaagaagtccttaaagacctgatggagctgaataccatggcatgagaactacgtgattaaatgcacaagcttcactagctgatttgatcaactggaagaaaggtatcagtgactgaagatcaaatgaatgaaatgaaggagaagagaagtttagagaaaaagaataaaagaaacgaacaaagcccaagaaatatggactatgtgaaaagaccaaatctacgtctgattggtgtacctgaaagtgacggggagaatggaaccaagttggaaaacagtctgcaggatatcatccaggagaacttccccaacctagcaaagcagaccaacattcaaattcaggaaatacagagaatgccacaaagatactcctcgagaagagcaactacaagacacataattgtcagattcaccaaagatgaaatgaaggaaaaacgttaagggcagccagagagaaaggtggggttacccacaaagggaagcccatcagactaacagcggatctcggcagaaactctacaagccagaagagagtaaataatatttaacattcttaaagaaaagaattttcaacccagaatttcatatcagccaaactaagcttcataagtgaaggagaaataaaatcttacagacaagcaaatgctgagagattttgtcaccatcaggcctgccttacaagagctcttattcaagacaagataagtgaccagagctatgctggtcaggactgggcattggagaggcttcctaatccaacagatatccccgaaaaaaaatgagtcacttccaatcattataatagtctagaaaatactgtggtaaaatcatctcataggcttcctgacaagtgctgaactcccctttagaccactgactggctctgcaatcttaacaagcttctctgactcttcattataaaagcaaacataacttagctcacggagagggctgctttaaagacaaaatgagattactgattacaattgattctaattttatttaaaagcctttacaaatgctcataatatttgaaacagtattcccaagttctaaattttactcattggcatattttcagttcatatttttagaagagaatggttaccattcttacagctatgctttgttgctacaaagataactaatacgccacaaacgaaaattcatttgcacacacacaggaatcagaagtcatctaatttgcattatcataaaacaggagcaggtctgctgtgcacatgaaattctttttaatttctgtcggtttttcaatggtttcaataatcaatttaagcagaagcattcaacatcctacacttctgtttcttgctttgaattttgaataatgacaattatggcatcagtaatcatacatgcaggcagacataatactgaatatgtcttcagttccttgcacctgacattgtgttttgttataacaggtgccccaaatatacttattttaattcaaaaaaagtataaagtcatttaaaataacctccaaaccataaaggttccatgcatttttaaatgcaaattaattagagcttgtcttttctgagattgtacctgtatctggggttaatctatttcttctctttggcaccagaaactattactttgggcctttttcatgattttctatatgcaagcacttacttattaaatattaagtaagtatttaataatcacttactatatatcttacttccttgtcttatggagtttttgttttctaagctgtttttagagagatgtaagatactaaaccaaataaatgactcaaaattagaagggatgaaaaggtaggggcaatattttgagtattaagtagatgtgttattcattgtataaaagttaactttttataggtctatttgaaggttagatggctatatcttgtatcaataattgttacatgacttttattagcttcatactttgtgccactttgtgaagaaattagctggtgtcttgataattttctgatttctttgattcgtccagtcatgatttagacaccagaattttacttgtgtgaggaattttaaaaggagaagataggttggggctgatcttttttttagtatattccattttacataaaaaagaaagaaagaatattgaagtctcagagtattgatataatttgtcagccctctcatctctatattttgtacctcaaattatgcactccttatactacattgatattggctgcacttttgtttgcacttaagtattttctgtacaaatgtccctttcaattttgttttgtttataaaagttctttttttctaattggatagtgggattcctttgacatcataaaattataaaatcaaaaaagatttcttattcatggtatgtggttttaaaaaatgaaaaatacataaagatgacttatagtcctaggaaagtgtgagcatccttcgattcacttagcacactccaacatatgtcaatactatagaagaacggaagctgtctcacaatcatatacaagtttcattacttgacctcttttagatcttctttataggtgaaccttcataaatgagaaatattggaactgtgatttggaaatcagaataatccttcatattgacaaatgtttcagtggcctggtggtcacttaaccttttgaaaacctttcctttagttctcctgtaaataaattgttcagaaggcaaaataagttaatgctttaacagttataaattttatgtctagaataatttcttttaaaatctgatgatatggactaaattaaataaggcttatttaatttgaaagcttaaattcaattgatttcttatacattttcacaaaattagtattgaaatgaaaatctgtagggtcagacgcaattttgataaaggtcaacactaaaatgttcagtctaacaactctattaatcttactaatttggttgaaatactatgcagactaccttaaattagcaaatgtcttatgaaatagaccaaaagatcatttcttaggtgggattgtgacattttccttcctgttagtcctttatattatgttaccttttatgaaatctgaaaacgtttagtagatttaattatgaatagtaaattatccattttctatagtcaccactaatacggcaatgaatacatacacaatgtattcattataacaaaattttgtagtatttttttactaaaataagtttttagttaatccaagttaatcttttgagtgcagtttgtttagttccgtgaacaaaaccaaaatgaatcagcatatttcttcacaattaatagcatgatcattagcaatgtccctgctcctcatagaatgtctttaagcatctttaacaatgtttaatttcaatttgtctaatctgagcagaagtaaaaaagaaattaatctttctgttgaaagccagtgataacagacacaaaaacacataactgcttttagtaaaaaaaatacagtttcagtagagaagattggcaaatgataaattactcacttttaaattattactactgggtaaagaagctgaaaaatctgatgctacctagatatagacatttcaaagtttttatgtgccaatgccaatatatataatttatcaacacaacctacagtttgcaatgatttaacaatgtattattttacatttcattattttattgaaaattccattttgaggaatatatatgcaaatcttcacttttgattaaaattggtggtgtcaaattgttctcctgaggtacacaatccaccagaaaggaaaagaagggatctatccaatactgagtacctactgcatgcttccactgttctgttatttgttttatttaatccactttaagagtcatcacaatttctgactttctttgtttggaaactacctatggagctaatagtcttaacctaaaaacatctctctgtgagttaaatgttactttttttttataacaaagattaatatctatcctaaccatgagttaggaatgtccaattaggagtatggaataatggctcagaatgttccaatttcaacattggtaagaattttgaaagtttaaaactaaaatttgacctttgggaggctttcagataacacattggtgttttattgctcaaagtattagttgaattttaaagtctttacaaaccatcatacacagaggtctgagaaatgcctctgtttagggctgcctgaggccctcacacatttttgccatgtcccgatagaaatagacataacaattttatgattagcagcaactcaagaaagctaaccaccagggtaggatcacctcttaaaaaaaaatcacacggccgggcgcggtggctcacgcctgtaatcccagcactttgggaggccgagacgggtggatcatgaggtcaggagatcgagaccatcctggctaacacggtgaaaccccgtctctactaaaatacacaaaattagccgggcatggtggcgcacgcctgtagtcccagctacatgggaggctgaggcaggagaatggtgtgaacccgggaggcggagcttgcagtgagtcgagatggcgccactgcactccagcctgggcgacagagctgaactccatctcaaaaaaaaaaaaaaaaaaaaaaaaatcacacaagaatatttgcttgagaagttccagacattttgatcttatagaagtgagtagatgaaacattagagtaataaaaaacaatagaatgttaaaaatgagatttgaggcagttccaacatggcagaataggaacagctccagtctacagctcccagtgtgagcgacacagaagacgagtgatttctgcatttccaactgaggtaccaggttcatctcactggggagtgtcagacagtgggtacagacacagggtgcagcactgagcgtgagttgaagcagggcgaggcatcgcctcacctgggaagtgcaaggggtcaggaattcccctttcatagccaagcaaagctgtgacagacggcacctggaaaatccggtcaccccaccccatattgtgttttccagtggtcttgtgcaaacggcacaccaggagattatatcctgcgcctggctcagagggtcccacgcccacagagcctcgctcattgctagcccagcagcctgagatcaaactgcaaggcagcagtgaggctggggaggggtgttcccattgctgaggcttgagaaggtaaacaaagcagccaggaagcttgaactgggtgaagcccaccgcagctcaaggaggcctgcctgcctctgtagactccacctctggggggggcagggcatagccgaacaaaaggcagtagaaacctctgcagacctaaatgtccctgtctgacagcttttgaagagagtagtggttctcccagcatggactcctgagatctgagaacgggacagactgcttcctcaagtgggttgctgaccccgagtagcctatctgggaggcacccccccagtaggggcagactgacaccacacagctgggtacccctctgagacaaaacctccagaggaacgatcagacagcaacatttgctgttcaccaatatccactgttctgcagcctccgctgctgatacccaggcaaacagggtctgagcagacctccagcaaactccaacagacctgcagctgagggtcctgaatgttaaaagcgaaaactaacaaacagaaaggacatccacaccaaaaccccatatgtcaccatcatcaaagaccaaaggtagataaaaccacaaagatggggaaaaacagaatagaaaaaattgaaaatttaaaaaatcagagcacctctcctcctgcaaaggaacgcagctcctcaccagcaacggaaacaaagctggacagagaacgactttgacgagttgagagaagaaggcttcagatgatcaaacttctccgaactaaaggaggaagttcgaacccattgcaaagaagctaaaaccagtttaataataacattttttttgccagtaattgggtatattttgggaaatagtagaataaagatttgtagactctcttacatctcctaaaagaaaattcactacatgtatgtggattactgattaaataagtttttccatatgcttgaattttttatacacacagtatatactctattatatgtatacacagaatacaactctatcagtattgaacttagtttgtcattccataaaatggccatagaaggtgctggagtcttgtgtcccagttaccaggttaacctagcaatgcgtaacgtaacgaagtacaag

chr22:29066035-29066121 chr5:83347360-83348118 gagctc chr5:83342110-83347372 chr22:29065878-29065762(-)chr22:29065899-29066032

gagctc chr5:83342110-83347372 chr22:29065878-29065762(-)chr22:29065899-29066032 (Primer gap) chr22:29066035-29066121 chr5:83347360-83348118 gagctc

Consensus sequence for insertion at chr5:119565858 (Twin priming)

ctgttgtacttcgttcaggttacgtattgctaaggttaaacagacgactacaaacggaatcgacagcacctcccaaaatatacccaattactggcaaaaatgttattattaaactgatgagaaaatataaaggtttaaaaaaaaaaaaaaaaaaaaaaaaaaaaaaaaaaaaaaaaaaaaaaaaaaaaaaaaagaaaattatgtcatctgtgaacaaatagttttgtttattccttccctaatatgtatatgatttgaattgtcttttcttgtatcatttcaaataggtgtggcagatgatatccctaccttgttttctgatcttatggagaaaacacctagcttatcattatttagtattatgttaatgtagggtttttttgcagatgttctttatcatgttaaacaagttcctgtgtattccttctttgttataatgaataaagtattgcagactgaaaatgctttgtaaagatttattgatacgatcatatgattttttcctttagcctgttgataacagtgagttacattaattggtttcaaatcttgaccagtcttgtatacatgaaataaaccttacttggttataatatatgtaagtttttttagacattgttgcatttgatttgtggattttttttaaggattttttgcgtctatgagatattcatgagagatatgggtctctaattgccttccttgaaatgtatttgtcttggtttagtattagggtaatgccagtgttattgaataaattagaagtgtgctatctgcatcattttataaaagagattgtagaaggattgatataatttctacattaaacatttggtaaaattcactggagaaaccatttatgcctagtgtggtcttttttttgaaagctattaattattatataaaaatattaccaattatatgtatacatatatttacaaccatatatgaaaggtttatgtaaatcatatacatataaattatatatcaatatatatacaaattatatataaaattgtataaattaatatatatatacctttttttagattatcttttctctttgtgtaagttttattagtttttgtatatttttttaaggcataggtccatttcacggaagttatcaaatttgtagacataaagttttacatgacttttacttattatacttttaatgtcttgagatgagcagtgatggctatttcttatttctgatattagtaatttgtgtcttctctcctttttcttaaatagcctggctagagaacaatcaattttattggactttttaaaaaaataccttttgttttcttggagttttctagattttctcttttttttaattttattggattttgttctagtttttgttactttttttattatttcttttattttgcttgatttagactcagatgctctttttttctctagtttcctaaactggagagcttagattactgattttagattattcattaacttttgttttaaattcagaatttacatgtacaggtttgttatataggtaaacttgtgtcatgggtgtatgttgtacaattattttgtgatccaggtattaagcatagtacccattagttattttctgattctctcctccttctaccctccacccctccagtaggcccaatgtctgttgttgtcctctatgtgtcctcctcatttaacttccacttttaagtgagaacatgtgatatttgttttttctgttcatgcatccttctagtactgggttttttttccttcaagacaatgggatctcttttgacccagtgtgtgtctagaaatgttatctaggagctagagcctggaatgagggcctcactactctgcccagtgccctatcttgctgtggctgagcttgtatcaagatgcaaaacaaagtcctctttactctttgctcttatctccttaagcagaaggaaagatacattttcattgctatgagctgtactgcctacggtggggggggacgatggcagaagctctcctttggccactctgtcttgtatttccctaggttacatgccacccctagtcctttggctgttagcccagcccagcactaacagttgcctaggaattgtagtccttgtgtcctaggctgtctttcagtttacctaggaccctagagtgcattggcctgtggtgatgagggctgctgataccctgagttcagaccaatggggttggcaatttccctttggttaggtctggtccaaattctccctctgttcacaggcactggctgagccaagcatggctgtactctctgctatgatagggcagcactgaattcaatgtaaagtcccagtcactgcactctccctccccagatgcacagactctgtgctatagaagctaatcaaggatgggaaaagaagtaacattaatgatccaagactgtcttttcctatcctcctcaatgcctcttttaatgatacgaagttaaaaccagttactatcattgcctacctgatttttttggttcttgtggtggtgctttctgtgtgcagatatttgttaaaatttgatgttcctgcaaaaattacaagtggagtaggcttccattcctccatcttgctccaacctttcagttttttgtagacttttaaatattttctacatagaaaaaaattatgtcatcattaccttttattttggccctaactggagtacccggccttattaaatatatgttcctcaatatcaaattgtactaaaaatcagaaactaagctctagtagagaactggtcaaagggaagatctttgtggagtcagcagtttcctctgtcctacaatttggcaaaataatctcataagcacaagaaggttaatatgcttgcattaaaaatgttaagttacttataatcaatttaaatgtaaagttcaaataccttaacatgttcacattactttttttaattcacaaaacgttcttatctatctttattttagatgttttccacatacatgcttagtgaaaggtgctgtcgattccgtttgtagtcgtctgtttaaccttagcaatacgtaacctca

chr22:29065832-29065899 polyA chr5:119565843-119567380 atgcat chr5:119564928-119565858 chr22: 29065443-29065419(-)chr22:29065468-29065782

atgcat chr5:119564928-119565858 chr22: 29065443-29065419(-)chr22:29065468-29065782 (Primer gap) chr22:29065832-29065899 polyA chr5:119565843-119567380 atgcat

Consensus sequence for insertion at chr6:112763097 (Twin priming)

ttgttgtacttccgttcagttacgtattgctaaggttaacctggtaactgggacacaagactccagcacctttcactaagcatgtatgtggaaaacatcttaaaaataaagatagataagaacgtttttgtgaattaaaataaaaaaaaaaaaaaaagaaaaaaaagaaacttcacatggggccacatgtctaataaaggtgactgaatcccaaaattatattggtaacatggaaattctgataagggaagaatattcccaagtgtgaccagttagagggccaaaataaaaggtacaatttacccaattattatacctaagtacccggccttattaaatatatgttcctcaatatcaaattgtactaaaatcagaaataagctctagtagagaactggtcaaagaatatttgttttaaacctattggcttccagtagcagaaacgcaaagagattttctggcagtacattctgtacttttggttccttgcattttgggccaattctgaaatcaaaaggtgctggaaaatggtttgaggaagtgaaaggcagctctctagcaagtaatcaggcttcaggttggtgtgaacagcaaatgaatattcaattatttgtatgtcaattgcttgtctacccttccagtagaaatataacatcaaggtgtaataaaggcacaatttcatccagctttgctgactttaggtgttttcaatcataattgaaaacattcctttcaatcaaatcacagataccagcattattcaaagagtgaataatacaatagaaattccaaatattttgctaatccctttcaaaacaaataagccttctgatatttccacttaagttatgtattgtaacttaatataatataataatttaaatatggcttgggtaataaatataaatttaatcaacaatttttctttctactgagggaaagagatgggatgagaataaataatggtaattccctacactcagtttttttaccataagaaggatttgagcaggtaggtgactccttgaccacatttcccctaactttgcaacctgggcatcatcaactctactcattcaaacagggcattttttcaaattttcaattaaaaaaatatcttcgataaactcaagagatttcttaaaggatttttaacatctaccaaaaaagctcacggtgataacattttataagttgagattgctctttaactaagcccaaatatttcaagatttatgtcctaacattcaaatttcaagtaaccaatgtcacattctcaaagttaaaatgccaaaattttaaatggtggtttatagtgtactggtatgccacttttgactgtagtttctttaaaatgtttctaagataaagcttagtttttttttttttcttatgaaacaggatcttgttctcttgcctaggctggagtgcagtggtgcaatctggctcactgcagcagggctaaattctgcttcttgtggtggtttgcaaagtcactcaaacactgtaccttcatttattttctaattctaaaaatatttttctattttctaagttttacttttgtaataagaaaatagaatttcataagaaataaagatactgtgttaaaattctagcatttggtctcccaggacactttcattccactgagggacaactttcaatttttatacctaaatttgatccctctaggaagaaaatttcagccatttctaagattgaagtcaacagcttcaggaatggatttcccgttactataattgtttgggttcttctttcatctatgttggtatttgcatatataattgcaaataccagagatcaatcttaaaagcagtgcactctagtatttggtatgcaagtggaagattcactttattcactccatctccaaaggctgaggtacctggctaataggtggttaaagtgagaagatctgaaatgtggtctatcttccagaaccacccagagatcttttttaaaacctcctgggacactgtcaatgctactgaattagaaactcttaagaaggagtaaggaggacttactcaaagacctgacctgtgtgatttttaataccacctaagtgatgttagtaattgggcagttttgtgaatagctaattgttaattggccagtttgaaaccaggtaatattaggagaaagtgccactctaagatttaataatagtatgtggctttattttttatatatatcgtataaacatatgtggtatcctttacaatttccttagaatatttgttttttttttttttttttttttttttttttttttttttttttttttttttttttttagattaagatttaaagatctttattgccccccattttattttcagtaatacagggtaaaatggagtaggattacttgatctggccatctatggccatttatcagaatgacaaactaagttcaatactgatagagttgtattcatgatattgtatacatataatagagtatatactgtgtgtataaaaattcaaacatatggaaaaacttatttaatccagtaataaacctttatattttctcatcagtttaataataacattttttgccagtaattgggtatattttgggaggtgctggagtcttgtgtcccagttaccaggttaaccttaacaatacggcccc

RC:

ggggccgtattgttaaggttaacctggtaactgggacacaagactccagcacctcccaaaatatacccaattactggcaaaaaatgttattattaaactgatgagaaaatataaaggtttattactggattaaataagtttttccatatgtttgaatttttatacacacagtatatactctattatatgtatacaatatcatgaatacaactctatcagtattgaacttagtttgtcattctgataaatggccatagatggccagatcaagtaatcctactccattttaccctgtattactgaaaataaaatggggggcaataaagatctttaaatcttaatctaaaaaaaaaaaaaaaaaaaaaaaaaaaaaaaaaaaaaaaaaaaaaaaaaaaaaaaacaaatattctaaggaaattgtaaaggataccacatatgtttatacgatatatataaaaaataaagccacatactattattaaatcttagagtggcactttctcctaatattacctggtttcaaactggccaattaacaattagctattcacaaaactgcccaattactaacatcacttaggtggtattaaaaatcacacaggtcaggtctttgagtaagtcctccttactccttcttaagagtttctaattcagtagcattgacagtgtcccaggaggttttaaaaaagatctctgggtggttctggaagatagaccacatttcagatcttctcactttaaccacctattagccaggtacctcagcctttggagatggagtgaataaagtgaatcttccacttgcataccaaatactagagtgcactgcttttaagattgatctctggtatttgcaattatatatgcaaataccaacatagatgaaagaagaacccaaacaattatagtaacgggaaatccattcctgaagctgttgacttcaatcttagaaatggctgaaattttcttcctagagggatcaaatttaggtataaaaattgaaagttgtccctcagtggaatgaaagtgtcctgggagaccaaatgctagaattttaacacagtatctttatttcttatgaaattctattttcttattacaaaagtaaaacttagaaaatagaaaaatatttttagaattagaaaataaatgaaggtacagtgtttgagtgactttgcaaaccaccacaagaagcagaatttagccctgctgcagtgagccagattgcaccactgcactccagcctaggcaagagaacaagatcctgtttcataagaaaaaaaaaaaaactaagctttatcttagaaacattttaaagaaactacagtcaaaagtggcataccagtacactataaaccaccatttaaaattttggcattttaactttgagaatgtgacattggttacttgaaatttgaatgttaggacataaatcttgaaatatttgggcttagttaaagagcaatctcaacttataaaatgttatcaccgtgagcttttttggtagatgttaaaaatcctttaagaaatctcttgagtttatcgaagatatttttttaattgaaaatttgaaaaaatgccctgtttgaatgagtagagttgatgatgcccaggttgcaaagttaggggaaatgtggtcaaggagtcacctacctgctcaaatccttcttatggtaaaaaaactgagtgtagggaattaccattatttattctcatcccatctctttccctcagtagaaagaaaaattgttgattaaatttatatttattacccaagccatatttaaattattatattatattaagttacaatacataacttaagtggaaatatcagaaggcttatttgttttgaaagggattagcaaaatatttggaatttctattgtattattcactctttgaataatgctggtatctgtgatttgattgaaaggaatgttttcaattatgattgaaaacacctaaagtcagcaaagctggatgaaattgtgcctttattacaccttgatgttatatttctactggaagggtagacaagcaattgacatacaaataattgaatattcatttgctgttcacaccaacctgaagcctgattacttgctagagagctgcctttcacttcctcaaaccattttccagcaccttttgatttcagaattggcccaaaatgcaaggaaccaaaagtacagaatgtactgccagaaaatctctttgcgtttctgctactggaagccaataggtttaaaacaaatattctttgaccagttctctactagagcttatttctgattttagtacaatttgatattgaggaacatatatttaataaggccgggtacttaggtataataattgggtaaattgtaccttttattttggccctctaactggtcacacttgggaatattcttcccttatcagaatttccatgttaccaatataattttgggattcagtcacctttattagacatgtggccccatgtgaagtttcttttttttcttttttttttttttttattttaattcacaaaaacgttcttatctatctttatttttaagatgttttccacatacatgcttagtgaaaggtgctggagtcttgtgtcccagttaccaggttaaccttagcaatacgtaactgaacggaagtacaacaa

chr22:29065832-29066118 polyA chr6:112763084-112763907 ctgcag chr6:112762040-112763097 chr22: 29065556-29065288(-)chr22:29065715-29065782

ctgcag chr6:112762040-112763097 chr22: 29065556-29065288(-)chr22:29065715-29065782 (Primer gap) chr22:29065832-29066118 polyA chr6:112763084-112763907 ctgcag

Consensus sequence for insertion at chr7:152870668 (Twin priming)

ggtatgtactggtgactggacagttacgtattgctaggttaaacagacgactacaaacagaatcgacagcacctttcactaagcatgtatgtggaaaacatcttaaaataaagatagataagaacgtttttgtgaattaaaaaaaaagtaatgtgttcctcaatatcaaattgtactaaaatcaggaaactaagctcctagtagagaactggtcaaagggaagatctttgtggagtcagcagtttcctctgtgtccttatccaattctatggggttttaaggtttctcatcatttctgtgctaatctctcagggttgagtattggtctacatttcctgcaccacgaggatgcatggggattatggggattatggagattacaattcaggatgagattttgggtggggacacagccaaaccacatcaagagtttcagccaaggcagggcaataaatcaggcacggtcgatcagggaacactggtttcagagtgtaggataccaaatagcaacatcaactaaaaagactgaacgatgaaatgtcaagaatccaggccaaactgcctagagaatgagaagccagaattaaggtaacaaatatgacaatgcctggatggggagcaaagatattggtgggcagggattctctttttttttcttttgaggatttgtgattgaggtcatttacatatcaatattaaggacatgtgtaaaattagtgcttgttgctatcattggcaacaaatacatttgtgtatagggatttaggttttacttacattgtgaagtataattaaattatagacttttacagtaaatgtgaatctcagatcacaaattttaaaaatatctgaacttaatttgtattcaggccctcattctaccagtctcaataattttggtttcccttttcttcctttcctcaagattactaccatttctggggacgaagcattgactagggttgctcttcctgtcatcctctaaggcacattgctgaggatcagtcctcactgcctctcaatgtctttcccaaaccaggtcttctgctgcctctgaccctctctcacatggaaattgtcttttcccaacataggatctttttttttttttttttttttttttttttttttttttttttttttttttttttttttttttttttttttttttttttttttttttttttaatcagtaataaacctttatattttctcatcagtttaataataacattttttgccagtaattgggtatattttggaggtgctgtcgattccgtttgtagtcgtctgtttaaccttagcaatgcgtaactt

chr22: 29065782-29065705(-)chr22:29065491-29065590chr7:152870685-152870782 atgcat chr7:152869933-152870668 polyT chr22: 29065912-29065834(-)

atgcat chr7:152869933-152870668 polyT chr22: 29065912-29065834(-) (Primer gap) chr22: 29065782-29065705(-) chr22:29065491-29065590 chr7:152870685-152870782 atgcat

Consensus sequence for insertion at chr8:114925191

ttgttgtacttcgttcagttacgtattgctaaggttaaacagacgactacaaacggaatcgacagcacctcagtcacctttattagacatgtggccccatgtgaagtttctttttctttctttttttttttattatactctaagttttttagggtacatgtgcacattgtgcaggttagttacatatgtatacatgtgccatgctggtgcgctgcacccactaatgtgtcatctagcattaggtatatctcccaatgctatccctccccctcctcctaccccaccacagtccccagagtgtgatattccccttcctgtgtccatgtgatctcattgttcaattcccacctatggtgagaatatgcggtgtttggttttttgttcttgcgatagtttactgagaatgatggtttccaatttcatccatgtcctacaaaggatatgaactcatcattttttatggctgcatagtattccatggtgtatatgtgccacattttcttaatccagtctatcattgttggacatttgggttggttccaagtctttgctattgtgaatagtgccgcaataaacatacgtgtgcatgtgtctttatagcagcatgatttatactcatttgggtatatacccagtaatgggatggctgggtcaaatgcatcacgattattatccatcttaaagtaaaagcagaacttcaaacaaagatggagaaaatattttttttaattcttaacatcaatttcacagtgcattaaataaagttagttaccattcacctaattgaacattatgtcatgtaataatattaaaataagtgtatgtgaagtagctgcatactaataaatatttatgaacagcttttttatttaatgtgcaatgctaactgagctctttttaggggcaggcctggaggtgacaaaatctctcagcatttgcttgtctgtaaagtattttatttctccttcgcttatgaagcttagtttggctggatatgaaattctgggttgaaaattcttgtctttaagaatgttgaatattggccccactctcttctggcttgtagggtttctgccgagagatccgctgttagtctgatgggcttccctttgagggtaacctgacctttctctctggctgccctgaacatttttccttcatttcaactctggtgaatctgacaattatgtgtcttgggttgctcttctcgaggagtatctttgtggcgttctctgtatttcctgaatctgaacgttggcctgccttgctagattggggaagttctcctggataatatcctgcagagtgttttccaacttggttccattctccccatcactttcaggtacaccaatcagatgtagatttggtcttttcacatagtcccatatttcttggaggctttgctcgtttcttttttattcttttttttctctaaacttcccttctcgcttcattttcattcatttcatcttccattgctgataccctttcttccagttgatcgcatcggctcctgaggcttctgcattcttcacgtagttctcgagccttggtttcagctccatcagctcctttaagcacttctctgtattggttattctagttatacattcttctaatttttttttcaaagttttcaacttctttgcctttggtttgaatgtcctccgtagctcagagtaatttgatcgtctgaagccttcttctctcaactcgtcaaagtcattcttcatccagctttgttccgtttagaaaaagaaaaggcagattacttcaggcttaccaatttttgtatcctaataaaggtaaggctactctagatgttagaactggaaagttgtcagaaatatatatttcacccctaatagtatgaaagtcatctaactcaatatttaaccaagtaaagagataatgactcaactgcagagttaggtaaaattacagtaaaacctcttgagagtctatggacagaataataaccaaataaccaaagaagggcactgatgtataatgaaataacacaaagcaagaaaaatagattctaaggattaaatatatagaaggccagcaagctgtaggtgttagaaaaaaaagttcatcatctcactatctacaattacttataatttgtcctcccaagtaaaatacagcctcctgcaatataacattaactattgcagtatgcagctgtcctcctacccaaagggcctttaaatattagttcatgacttcaaaatctaggggctttaattatctcagacatggggacaaatcttccaatgtccacatcttttgatttaaattctaggaaagtctgactcattaaaaatgttgtctttcttctttcaggtattttataatttttttgccttgctgaagttttcttatgtgatatataccatgctcttttattctagtatgtgcttcattaatttgcctggacagtcaaatattgacaacaggggagttggtggtctctaaattctaagaacttgaattgatgataaaaccaaatataggaaaagaatgttgttttagttttagagcaaaatataagagatgtaagtagtctttgggtctttatttcaaagatcaatagttgtcttatttttttaaaatagaagaataaaagaggggtggaggagggcagctgcatctctaacaagtaactaagagcttttcttcatcctgagtaagcgttttctggactgaactactggttttggagtagctgaaggtgaaatgcaaattagtagaaaccgggaagcaatgctccagggaaaagctgagtataagagtctcaggtttgagaagcaaataattctttaagcattttgtgggtatcagattctggaaaatatttttctggactggttaagtacttcgatctgtaagcaagagacttggaatgaaggcaacaatacgtaaagagggattaaaggaattgtaaatctcaggtttccatatgcaaatgtcaacattcatactgtggcataatttttaaatggtttatataaaatatttttgtactctatgctcccaaatccttctgataagacctcaactattgcattccaagtattcatgtagatttcttgcttacctatgtgttcatgaacattttgattctaaagactttactcatctctacacctacaaatttagcgcctgtgcttgcctagattgagcactcagttagaagttagagaagataaatgaattaacaacaagcaaatcttcttgaaagagcaactgctggtatggaagatgccatagttttacggtgagtgggcttttgtccagaactataaaagcatcaaacaaagagacgaaccctaataaattgtagttttttattatccatcttttttttttttttttttttttttttttttttttttttttttttttttttttttttttctcatcagtttaataataacattttttgccagtaattgggtatattttggaaatagtagaataaagatttgtagactctcttacatctcctaaaagaaaattcactaagcatgtatgtggaaaacatcttaaaataaagatagataagaacgtttttgtgaattaaaaaaataatgtgaacatgttaaggtatttgaacttacatttaaattggattataagtaacttaacattttttaatgcaagcatattaaccttcttgtgcttatgagattattttgccaaattgtaggacagaggaaactgctgactcacaaaagatcttccctttgaccagttctctactaggagcttagtttcctgatttttagtacaatttgatattgaggaacatatatttaataaggccgaatacttaggtataataattgggtaaattgtaccttttattttggccctaactggtcacacttctgggaaggtgctgtcgattccgtttgtagtcgtctgtttaaccttagcaatacgtaactt

chr22:29065354-29064763(-)chr8:114925178-114925402 gagctc chr8:114922708-114925191 polyT chr22: 29065887-29065404(-)

gagctc chr8:114922708-114925191 polyT chr22: 29065887-29065404(-) (Primer gap) chr22:29065354-29064763(-)chr8:114925178-114925402 gagctc

Consensus sequence for insertion at chr8:107979180 (Twin priming)

ttggtatgcttcgtttcagttacatattgctaaagttaatgaggaaacacgatagaatccgaacagcacctgccagatgaataatcctactcatttaccctgtattactgaaaataaaaaatggggagacaaataaaactttcaatatctttaaaaaaaaaaaaaaaaaaaaaaaaaaaaaaaaaaaaaaaaaacaatgttgaattgaaaaagttgaataaaattgcataatatatgagtgacctataattatgtacctaattattacatggtatcaattttgaactgttacttgttttagaaactgcctttatgtagggattgctacgtatttgcgtaaatttattgtagaaagaaagaataataaatacatcagttacttctggaaaggtggtaaaggaatgattttgaaaagaatatgcacataactcaatgatgttgataacgttcctgtttttaacctcaacagtgggttcaccagtgttggaggttatactttctaaaatctcataaattatgtatatgtattggttattacataataatgcttaatcagagcttttgtttattattgatatactcttcataaattgttttgctaaaaatagtaaagaaggattcattcaataaattatattatagatgagtaaacataatagaagaatgaatttgaagccatatagatctggactgggtttttgttttttgaagcttactagcttgagctatatacttatgaacagataacttaagacctctgaatatcactttcaataccagcagtatttattataagtctgctatgtactaaagtaccattcaggaacactggagatacagtggtgaacaaaatagacacccatctcaggcgctgcataagagatgtatgtagaagaggttctatgggagccctgtaaagggctaaccatgaagagcagcagtagttgcttggtgtgagggtgtgcgtcagtaccttcttacaaaaaatttctgtggaagcatgtcatgagccttttctaacttgccatatgctattcttgcaacggtgcagcattttgcacaatattgggaaatttctttcagctttgtgagcaaaacaacacaataagacttgactatgtattcacagcctatatcctgggaatatgaaacacaagctgaggacatcagccatataacaaaaaaaaaatgaaataaaatatttgtcaaagagatatccacactgccatgttttattgtggcataatttgcaataataggatatggaattgacttcagtgtctttcagtggatgaatgaataaagaaaatgtggtttatatacacaatggaatatgcaatgggatattcagccttaaaaacaaaatctaccattttgacaacatggacattatgttcagtgaaataagccagacacagacaaacaaatacccccatgatctgcttatctgtagactctaaaaaatgttgaattcatagaaaagtagagtgtaagaatggtagttagcagagactggggggtgggaagaggttgggaaaatattgatcaaaatacaaaatatcagcgtggatgaccaaggttcagagatctattgtacaatatggtgactataattaacaacaagtaatattataatcttgaaaattgctaaagaagaataggttttcagtgttctcaccatgaaaaatatatatgtgaggtaaatgtatttgtaaattagctcaattttgctattccacagtgtctaaattttgaagccatcatgtacatgaaatgtataaattttgtcaaccttttaaagataaaaaaattattaggtatccaaatgtagtcaagtgttagagggaagatatatcatgaagaggttgtgtttactccaataatcaaaggtggcttaacattaaaatgttgtaaagtgtaaaccactatatttcaaggttaaatgagaaaaaaatatagtcgtctcaacagatgcagaaaaaagcattttctataatccaacacccttttccaacaaaactaaactaaatgcttaataaaacaggaatagaagacagttttcatgtatctgctaagaagcatttacaaggaaaaaaaaagaacctatagcaaatactaatgatgtaatgggggcattcaatttattatcatgaaaaagaaaagatggataccccaaatcactactacctctattcaacattatgctgaagatcccatccagtttgtaagaagaaaatactataaatatggaaggaaaagaaatacaggctgtccatattattaaaggtgatttgattgtatatatagaaacccatctaccaacaaaatatgagagtgtgagtatttagcaaggtgtctgactataacaatatgcaaattttgttgtgttactatacaacaactataaaactagaaaacattttttaggaattctttgtaatagcaaaaaatatgtaagtaaaatgaaatagatattttaagaggatctgtagaaactaaggaagaaaagataaaaaagctgttttttaaagacataaatatgaaaaggaaatatccatgttcatgggatgggataactcaatatcataaaaatgtcaccttaaattgaactacagatttaatttgtttccaatatctataaactcaatatcataaaatgtctccttaaatttctcctcaaaatgaactatagatttaatttttttctaataaggtttttttgaaaacttgagaagctaattctaaaatttatttgaaaaggcaaaggctcagtaatgggtaaagtattgaagaaggataggaaaacaaaattgtgctaccagatgcaagtttttattataaaagctatgtcaatgaatacagtgtcaattaataagcaaaaaacctatcagacactaggacagaattcccagtaaaatagagagagcccaaacagatctatacataacagaacctgatatatggcaaagatgatattacgatatctggagaaaggaagtaagctaataataattaaaaaatacaggtattatttgtttatataataaacaatattatatataggcatatacaggcatcaattccaggtaagatcaaaaatttaaagcaaagggaatattttaaaactttgagagcaaaatatagaaaataatctttgtgaactcaggaataagaaaatattaactataaattttttttaagcaatgctaaactcacctagattaatgggaagaacttctgcttatctaaagacaccataatataaagttacaaactatttgtagcttttatgaatcaacaaataattagtattcattaagtataatgaactcttccacattgagaataaaataacaacttcctctctcccctcaaaaaaaactagtaagtgattggccaaaccggcatttcagatcagagaaagtactaatgacccatgcctcttcggtaatcaaaagaagtacaaagtaggatcacaataagatatctttttttttgcattatactaaaattaaaaattgatatattgttggtaagaaatggattgatgaaaaaagatatacagctctatagtataagcataattggaaacatcttatttgaaaaaacgttcatctgtatacatctaaacacctaacttatctataacccagcaattttactcctagaaacaaagaaacctagagaaatttgcatgtgagacccaaagatatataccagagattttataattgtcaataataactgttgtcaataatagcaatagaaatgaaaatcaggtaacaaagtcaatttacacctttgaaaaaacgtattgattatagcatagtcacataatattattacaaattgtgaaaatgaatatattatagctactggtgacacaataaacaatgttgaactcatcattttttatggctgcatagtattccatggtgtatatgtaccacatttttcttaatcgatctatcattgttggacatttgggttggttccaagtctttgctattgtgaatagtgccgcaataaaacatacgtgtgcatgtgtctttatagcagcatgatttatactcatttgggtatatacccagtaatgggatggctggatcaaatggtatttctagttctagatcctgaggaatcatacactgacttccacaatggttgaactagtttacagtccaccaacagtgtaaaaagtgttcctatttctccgcatcctctccagcacctgtttgtttcctgactttttaatgattgccattctaactggtgtgagatgatatctcatagtggtttttgatttgcattttctctgatggccagtgatgatgagcatttcttcatgtgtttttggctgcataaaatgtcttctttttgaagtgtctgttcatgtccttacttcccacttttttgatggggttgtttgttgttttttttcttgtaaattgtttgagttcattgtagattctggatattagccctttgtcagatggagtaggttgcgaaaattttctcccatgttgtaggttgcctgttcctctgatggtttcttttactgtgcagaagctctttaaaagtttaattagatcccatttgtcaattttgtctttagtgaattttcttttaggagatgtaagagagtctacaaatctttattctactatttcccaaaatatacccaattactggcaaaaaaatgttattattaaactgatgagaaaatataaaggtttattactggattaaataagttttccatatgcttgaatttttatacacacagtatatgctctattatatgtatacacagaatacaactctatcagtgcagtgaacttagtttgtcattccataaaatggccatagaaggtgctgttcggattctatcgtgtttcctattaaccttaagcaatacgtagct

RC:

agctacgtattgcttaaggttaataggaaacacgatagaatccgaacagcaccttctatggccattttatggaatgacaaactaagttcactgcactgatagagttgtattctgtgtatacatataatagagcatatactgtgtgtataaaaattcaagcatatggaaaacttatttaatccagtaataaacctttatattttctcatcagtttaataataacatttttttgccagtaattgggtatattttgggaaatagtagaataaagatttgtagactctcttacatctcctaaaagaaaattcactaaagacaaaattgacaaatgggatctaattaaacttttaaagagcttctgcacagtaaaagaaaccatcagaggaacaggcaacctacaacatgggagaaaattttcgcaacctactccatctgacaaagggctaatatccagaatctacaatgaactcaaacaatttacaagaaaaaaaacaacaaacaaccccatcaaaaaagtgggaagtaaggacatgaacagacacttcaaaaagaagacattttatgcagccaaaaacacatgaagaaatgctcatcatcactggccatcagagaaaatgcaaatcaaaaaccactatgagatatcatctcacaccagttagaatggcaatcattaaaaagtcaggaaacaaacaggtgctggagaggatgcggagaaataggaacactttttacactgttggtggactgtaaactagttcaaccattgtggaagtcagtgtatgattcctcaggatctagaactagaaataccatttgatccagccatcccattactgggtatatacccaaatgagtataaatcatgctgctataaagacacatgcacacgtatgttttattgcggcactattcacaatagcaaagacttggaaccaacccaaatgtccaacaatgatagatcgattaagaaaaatgtggtacatatacaccatggaatactatgcagccataaaaaatgatgagttcaacattgtttattgtgtcaccagtagctataatatattcattttcacaatttgtaataatattatgtgactatgctataatcaatacgttttttcaaaggtgtaaattgactttgttacctgattttcatttctattgctattattgacaacagttattattgacaattataaaatctctggtatatatctttgggtctcacatgcaaatttctctaggtttctttgtttctaggagtaaaattgctgggttatagataagttaggtgtttagatgtatacagatgaacgttttttcaaataagatgtttccaattatgcttatactatagagctgtatatcttttttcatcaatccatttcttaccaacaatatatcaatttttaattttagtataatgcaaaaaaaaagatatcttattgtgatcctactttgtacttcttttgattaccgaagaggcatgggtcattagtactttctctgatctgaaatgccggtttggccaatcacttactagttttttttgaggggagagaggaagttgttattttattctcaatgtggaagagttcattatacttaatgaatactaattatttgttgattcataaaagctacaaatagtttgtaactttatattatggtgtctttagataagcagaagttcttcccattaatctaggtgagtttagcattgcttaaaaaaaatttatagttaatattttcttattcctgagttcacaaagattattttctatattttgctctcaaagttttaaaatattccctttgctttaaatttttgatcttacctggaattgatgcctgtatatgcctatatataatattgtttattatataaacaaataatacctgtattttttaattattattagcttacttcctttctccagatatcgtaatatcatctttgccatatatcaggttctgttatgtatagatctgtttgggctctctctattttactgggaattctgtcctagtgtctgataggttttttgcttattaattgacactgtattcattgacatagcttttataataaaaacttgcatctggtagcacaattttgttttcctatccttcttcaatactttacccattactgagcctttgccttttcaaataaattttagaattagcttctcaagttttcaaaaaaaccttattagaaaaaaattaaatctatagttcattttgaggagaaatttaaggagacattttatgatattgagtttatagatattggaaacaaattaaatctgtagttcaatttaaggtgacatttttatgatattgagttatcccatcccatgaacatggatatttccttttcatatttatgtctttaaaaaacagcttttttatcttttcttccttagtttctacagatcctcttaaaatatctatttcattttacttacatattttttgctattacaaagaattcctaaaaaatgttttctagttttatagttgttgtatagtaacacaacaaaatttgcatattgttatagtcagacaccttgctaaatactcacactctcatattttgttggtagatgggtttctatatatacaatcaaatcacctttaataatatggacagcctgtatttcttttccttccatatttatagtattttcttcttacaaactggatgggatcttcagcataatgttgaatagaggtagtagtgatttggggtatccatcttttctttttcatgataataaattgaatgcccccattacatcattagtatttgctataggttctttttttttccttgtaaatgcttcttagcagatacatgaaaactgtcttctattcctgttttattaagcatttagtttagttttgttggaaaagggtgttggattatagaaaatgcttttttctgcatctgttgagacgactatatttttttctcatttaaccttgaaatatagtggtttacactttacaacattttaatgttaagccacctttgattattggagtaaacacaacctcttcatgatatatcttccctctaacacttgactacatttggatacctaataatttttttatctttaaaaggttgacaaaatttatacatttcatgtacatgatggcttcaaaatttagacactgtggaatagcaaaattgagctaatttacaaatacatttacctcacatatatatttttcatggtgagaacactgaaaacctattcttctttagcaattttcaagattataatattacttgttgttaattatagtcaccatattgtacaatagatctctgaaccttggtcatccacgctgatattttgtattttgatcaatattttcccaacctcttcccaccccccagtctctgctaactaccattcttacactctacttttctatgaattcaacattttttagagtctacagataagcagatcatgggggtatttgtttgtctgtgtctggcttatttcactgaacataatgtccatgttgtcaaaatggtagattttgtttttaaggctgaatatcccattgcatattccattgtgtatataaaccacattttctttattcattcatccactgaaagacactgaagtcaattccatatcctattattgcaaattatgccacaataaaacatggcagtgtggatatctctttgacaaatattttatttcattttttttttgttatatggctgatgtcctcagcttgtgtttcatattcccaggatataggctgtgaatacatagtcaagtcttattgtgttgttttgctcacaaagctgaaagaaatttcccaatattgtgcaaaatgctgcaccgttgcaagaatagcatatggcaagttagaaaaggctcatgacatgcttccacagaaattttttgtaagaaggtactgacgcacaccctcacaccaagcaactactgctgctcttcatggttagccctttacagggctcccatagaacctcttctacatacatctcttatgcagcgcctgagatgggtgtctattttgttcaccactgtatctccagtgttcctgaatggtactttagtacatagcagacttataataaatactgctggtattgaaagtgatattcagaggtcttaagttatctgttcataagtatatagctcaagctagtaagcttcaaaaaacaaaaacccagtccagatctatatggcttcaaattcattcttctattatgtttactcatctataatataatttattgaatgaatccttctttactatttttagcaaaacaatttatgaagagtatatcaataataaacaaaagctctgattaagcattattatgtaataaccaatacatatacataatttatgagattttagaaagtataacctccaacactggtgaacccactgttgaggttaaaaacaggaacgttatcaacatcattgagttatgtgcatattcttttcaaaatcattcctttaccacctttccagaagtaactgatgtatttattattctttctttctacaataaatttacgcaaatacgtagcaatccctacataaaggcagtttctaaaacaagtaacagttcaaaattgataccatgtaataattaggtacataattataggtcactcatatattatgcaattttattcaactttttcaattcaacattgttttttttttttttttttttttttttttttttttttttttttaaagatattgaaagttttatttgtctccccattttttattttcagtaatacagggtaaatgagtaggattattcatctggcaggtgctgttcggattctatcgtgtttcctcattaactttagcaatatgtaactgaaacgaagcataccaa

chr22: 29066032-29065775(-)chr22:29064270-29064978 chr8:107979169-107981862 atgcat* chr8:107978214-107979180 polyT chr22: 29066121-29066035(-)

atgcat* chr8:107978214-107979180 polyT chr22: 29066121-29066035(-) (Primer gap) chr22: 29066032-29065775(-)chr22:29064270-29064978 chr8:107979169-107981862 atgcat*

Consensus sequence for insertion at chr10:101386670

tcggtgtcgcgatcggttcgatgtgggatcgggatcgggatcggacaggtccagatctggtcagtcggataagatcaggatagaaagtgactcgtcagacggacaaaagtggaatcaggatctgggaaagaaggactaacggtgggactgaaaactgccctctacttatattttcctcaataaatgaaattacaaattaaaaaaaaaaacactgctcactgtaccatatttatgtacacagaatatcccacatgggctgaaatttccattcttaaagccatgtttgcccttaacaggaatatatgtgtaagtcagggaaagacatgaagtctactgttatatcttgattgaaggctttgccagcacaaagttgtttgacaacctgggtgttttatgcacccagcagataaatgaataataagatgagaagtgagtcaggtcacacttttcttttgtaagataaaggacagattgagaaagggagaaaggaagaagaattgtttcaacggctttttttttttcggaagaggaattttaggaaagaattaatccagatgaggagtagcaaattaattcccttgaaacaatgtttccctaaataacccttggaaagtcttcctgtacacagtctcagtttgaaaactaatgagataaataatgatatctcctgatcctttcagtcctcactgcttaccccatctctgaattcctgtagctcttatgttctgtttcttcaccatttaggaatcagtttctttttaagtggctgtaaggttagcagtcttatttttcatgtgtggctgctggaatcaatctcattttatactacactcatatcacatgcccaggtatgatggcagttcagagaaggggagtagtgtaaagcccggacgcagcagtaggacctgttttgaaccttcaataatgagaatttgataagaacaaagggaaatgcattctaaggaagagcagcttctctaggatataaattcacctgctgtaacaaaagattcccattaacagcaattgcttaacaagctttctcatgtctgagcacaagtggtctgagggtgataggatgcctcatggtgttaaggacacaggtttcttctaacttttgctctgccaccagctaaagtacaggccttgtcctcatggccaaaaataattatcttctgggtctgtgttctgacgagaagggagaaaatgagcaaggagaacatatccttttcttttaaaggtacaacccaggtgtttcaaacatcactttccctcatcccactaactagaacttagtcacacctcaatgttgattgccagggaagctggggaaaaatatatgggctttagctgggaagccacatgcccaaattgaaacttgaggtttctattactaaagaaaaaaaaaataagtggtctcatagagagatgtagaaaattacaagacagacgggagaatgtgaaaaaaacaattgtactgggaatgagagggtgaagccagctggaggcttctgggtcaggtggggacttggagaacttttctgtctagctagaggattgtaaacacaacaatctgcgctctgtgtctagctaaagttgtaaacacaccaatcagcactctgtaaaatggaccaatcaatgctctgtaaaatggaccaatcagcaggatgtggggaacagggccaaataaaggggaataaaagctggccacccctagccagcagcagcaacctgctcaggtccccttccatgctgtggaagctttgttctttcactcttcagaataaatcttgctggttgctcactctttgggtcggcaatatctttatgagctataacacccactgcaaggtctgcggcttcaatcatgaagtcagcgagaccacaaacccactggaaggaagaaactctggatacatctgaacatctgaaggaacaaactccagacacgccatctttaagaactgtaacactcaccgcagtgtccgcggcttcattcttgaagtcccaagaccaagaatccaccagaaggaataaattccagacactggaatacaagaaaacatgactatagtacctgaggttgcaggtgcaggaaactgctgaggttttgtgaagaatatcaccaataaagttcccttgcctactttttttctttgagatagggtctcactctatcacccagactggagtgcagtggcccaatcttggctcactgcaacctctgcatcccgggttcaagtgattctcccacctcagcctcctgagtagctgggattacaggcacacgccaccacgcctggctaattttttgtattttagtagagacagggtttcaccatgttggccaggctgatctcaaactcctgacctcaagtgatctacctgcctcggtctcccaaagtgctgggattacaagcatgagccaccgtgcctggcctgcttacttttataattttttaggcaggtttagtgctggtggatgtgtacgacagtgtgtatgggtttgtgtgcaggttaggtgcttactattcaaagtgtggtcaaggtctagcagcatcagcatcacttgagcttgtcaaaaatgcagaatctaggtaggcgtggtggctcacctgtaatctcagcactttgggaagctgagacaagaggatatgagcctaggagtttctaagaccattttgggcaagataatagaccctgtctctctaaaagaaagagagaaagaaaaagaagagagaaaaaaagaaaaagaagaaagaaaaagaaaaaaaaagaaagaagaaagaaggaaaaaaaaaaaaaggaaggaaggagaaaggaagaaagaaaaaaagaagaaggaaggaaggaaggaaaaaggaaggaaggagaaaggaagaaagaaagagaagaggaaggaagaaagagaaggaaggaagggagggaggagggaggagaggaagaaagaatcttaggctaatgagaccctacagaactgcagtctgcattttaacaaattcttcaggtaacatcatttatcatcacagagtactgggctagtttatgctatggtgtggaataatcctaaaagctctgtgtcttgaaacaactaagatttatttctcactgatgctgagaagtctgtctggggagcaagcaggtaacaagtagagaactctgcttcatgtcattctcaccagggccccaggctgacagagtctccaccatttgaacaatcactagtcatgtggcagaggaggaaatgtgtgactcttgtgcttcctcgtatttcactggccaaaagcaaatcccagggccatgcctatcttcaaggaggtggaaaaaacatcctactgtgtacctggaaggaggagaatcaaaatactgctgaagctaggggatcctcgaggcaggcaggcctttcaaggggggttatgacaatacagatgtaatcagattcagggaaagtatatcaatacgaaggaaaggagagcataaacagaagaaacagttcagaatgatgatgattctgaatgggcagtcaaggaagactcagctttagagtctcatctattgggcaaataatgaagactttcatggaaaacgaacattttggaggaatgggttggggtggggagtggaaaggctgagcttggaatggacacgctgagtcagaaatcaaggtggatattcaccccttgtttatctttccaacttcgtgttgtattcctctcccactcactcaccatgctccagccacactggccttctttctgttcctcatgcaccaaccttgttctcaccttagggttgtcgtacctactgttccttctgctggcatgcccttctcaagtctgtcatgactggctcctgcctatcattcagaactcaccatacatgtcgtctcctcagaaaggccttccttggccatacaaatgtaaccacccagtcacactatatcataccaccctatttcaaaaggtcagcatagcatccaggctgacattttttgtgtgtatttttctctgtcccacctccccacctcatcattaaaagctccatgagaacatggactatgtctgtcctgtatccccagcttcaagtacacagttggtacacaataaacattactgaatgttcccatggaaatttcagttaaaagtatgcaataaggaaataaagatagggttccaatgaggacaactggttttggaatgtaaaaagttctctaaggtattaaagatgaccaagaagcattaaacgtttagggatgttccacttcaatggctagagtgtaagcgactgagatgtctaaaaaaaggtagagtggttttctgaagctagtaaaagtgccaatgtggtgtggcggcaagtgttatctctgttaagaaaaaaaacaggaataggaaaaagaccgatggatttggaaaggtcatttatgaccaatgaataaaagagtgaaaaatgtaaagagcttaaggagagaaaggacatagaggtggataggtcattagtggagtaatctggcagtaaagggaattagtactctagcaggattgaatgggttttgtgagacctattcatttctcaaactaaatatgctagaaaaaaggagagactgaacatgctgtggggagaagttccttttaaaaaaatttttttgagacaaagtcttgctttgtcacccaggctggagtgtggcatgaacacggctcactgcagcttcgacctcccctggcgcaagcaatcctccccaagtagctgagactacaggtgcacaccaccatacccagctaattttgaatttttttgtacttttttgtatttattattttgtagagacggggttttgccatgttgtccaggctggtctcgaactcctgagctcaggagtttgagacaatctgggcaacataatagactctgtctctactaaaattcaaaaaattagccggatgtattagtgcttaactatagtcccagctacttggggggacagaagcaggaggatcgcttgagccaggggttgaagctgcagtgagccatgatcacgctactgcactctagcctgagtgacaggcaatatcttgtctcaaaaaaaaaaaaacaaagatgaataaataaacaaatacataaataaaagtgtaattggattgtttatagctcaaaaggataaatgcttgaggggatggataccccattctccatgatgtgattatttcacattgcaggcctatattaaaacatctcagataccctataaatatgcacctactatgtatccaaaaaaagacttttttttatttttttattttgagattgtctcggtctgtcgcctaggctggagtgcggtggcatgatcttggctcactgcaacctctgcctccctggttcaagccattctcatgcctcagcctcccaagtagctgggattacaggcatgcaccaccatgccggctaattttttgcattttagtacagacagtgtttcactatgttggccaggctggtctcgaactcctggccttaagtgatctgcctgctgtggcctcccaaagtgctgggaggtgtgagccaccatatctggccataaaattaattatttttttaaaaaaaaaatacaaagggcagggttaaagaatagaaagtgacaagaaaggttaatatgcttgcattaaaaatgttaagttacttataatcaatttaaatgtaagttcaaataccttaacatgttcacattactttttttaattcacaaaaacgttcttatctatctttattttaagatgttttccacatacatgcttagtgaaaggtgctgttcggattctatcgtgtttcctattaaccttagcaatacgtaact

RC:

agttacgtattgctaaggttaataggaaacacgatagaatccgaacagcacctttcactaagcatgtatgtggaaaacatcttaaaataaagatagataagaacgtttttgtgaattaaaaaaagtaatgtgaacatgttaaggtatttgaacttacatttaaattgattataagtaacttaacatttttaatgcaagcatattaacctttcttgtcactttctattctttaaccctgccctttgtattttttttttaaaaaaataattaattttatggccagatatggtggctcacacctcccagcactttgggaggccacagcaggcagatcacttaaggccaggagttcgagaccagcctggccaacatagtgaaacactgtctgtactaaaatgcaaaaaattagccggcatggtggtgcatgcctgtaatcccagctacttgggaggctgaggcatgagaatggcttgaaccagggaggcagaggttgcagtgagccaagatcatgccaccgcactccagcctaggcgacagaccgagacaatctcaaaataaaaaaataaaaaaaagtctttttttggatacatagtaggtgcatatttatagggtatctgagatgttttaatataggcctgcaatgtgaaataatcacatcatggagaatggggtatccatcccctcaagcatttatccttttgagctataaacaatccaattacacttttatttatgtatttgtttatttattcatctttgtttttttttttttgagacaagatattgcctgtcactcaggctagagtgcagtagcgtgatcatggctcactgcagcttcaacccctggctcaagcgatcctcctgcttctgtccccccaagtagctgggactatagttaagcactaatacatccggctaattttttgaattttagtagagacagagtctattatgttgcccagattgtctcaaactcctgagctcaggagttcgagaccagcctggacaacatggcaaaaccccgtctctacaaaataataaatacaaaaaagtacaaaaaaattcaaaattagctgggtatggtggtgtgcacctgtagtctcagctacttggggaggattgcttgcgccaggggaggtcgaagctgcagtgagccgtgttcatgccacactccagcctgggtgacaaagcaagactttgtctcaaaaaaatttttttaaaaggaacttctccccacagcatgttcagtctctccttttttctagcatatttagtttgagaaatgaataggtctcacaaaacccattcaatcctgctagagtactaattccctttactgccagattactccactaatgacctatccacctctatgtcctttctctccttaagctctttacatttttcactcttttattcattggtcataaatgacctttccaaatccatcggtctttttcctattcctgttttttttcttaacagagataacacttgccgccacaccacattggcacttttactagcttcagaaaaccactctacctttttttagacatctcagtcgcttacactctagccattgaagtggaacatccctaaacgtttaatgcttcttggtcatctttaataccttagagaactttttacattccaaaaccagttgtcctcattggaaccctatctttatttccttattgcatacttttaactgaaatttccatgggaacattcagtaatgtttattgtgtaccaactgtgtacttgaagctggggatacaggacagacatagtccatgttctcatggagcttttaatgatgaggtggggaggtgggacagagaaaaatacacacaaaaaatgtcagcctggatgctatgctgaccttttgaaatagggtggtatgatatagtgtgactgggtggttacatttgtatggccaaggaaggcctttctgaggagacgacatgtatggtgagttctgaatgataggcaggagccagtcatgacagacttgagaagggcatgccagcagaaggaacagtaggtacgacaaccctaaggtgagaacaaggttggtgcatgaggaacagaaagaaggccagtgtggctggagcatggtgagtgagtgggagaggaatacaacacgaagttggaaagataaacaaggggtgaatatccaccttgatttctgactcagcgtgtccattccaagctcagcctttccactccccaccccaacccattcctccaaaatgttcgttttccatgaaagtcttcattatttgcccaatagatgagactctaaagctgagtcttccttgactgcccattcagaatcatcatcattctgaactgtttcttctgtttatgctctcctttccttcgtattgatatactttccctgaatctgattacatctgtattgtcataaccccccttgaaaggcctgcctgcctcgaggatcccctagcttcagcagtattttgattctcctccttccaggtacacagtaggatgttttttccacctccttgaagataggcatggccctgggatttgcttttggccagtgaaatacgaggaagcacaagagtcacacatttcctcctctgccacatgactagtgattgttcaaatggtggagactctgtcagcctggggccctggtgagaatgacatgaagcagagttctctacttgttacctgcttgctccccagacagacttctcagcatcagtgagaaataaatcttagttgtttcaagacacagagcttttaggattattccacaccatagcataaactagcccagtactctgtgatgataaatgatgttacctgaagaatttgttaaaatgcagactgcagttctgtagggtctcattagcctaagattctttcttcctctcctccctcctccctcccttccttccttctctttcttccttcctcttctctttctttcttcctttctccttccttcctttttccttccttccttccttcttctttttttctttcttcctttctccttccttcctttttttttttttccttctttcttctttctttttttttctttttctttcttctttttctttttttctctcttctttttctttctctctttcttttagagagacagggtctattatcttgcccaaaatggtcttagaaactcctaggctcatatcctcttgtctcagcttcccaaagtgctgagattacaggtgagccaccacgcctacctagattctgcatttttgacaagctcaagtgatgctgatgctgctagaccttgaccacactttgaatagtaagcacctaacctgcacacaaacccatacacactgtcgtacacatccaccagcactaaacctgcctaaaaaattataaaagtaagcaggccaggcacggtggctcatgcttgtaatcccagcactttgggagaccgaggcaggtagatcacttgaggtcaggagtttgagatcagcctggccaacatggtgaaaccctgtctctactaaaatacaaaaaattagccaggcgtggtggcgtgtgcctgtaatcccagctactcaggaggctgaggtgggagaatcacttgaacccgggatgcagaggttgcagtgagccaagattgggccactgcactccagtctgggtgatagagtgagaccctatctcaaagaaaaaaagtaggcaagggaactttattggtgatattcttcacaaaacctcagcagtttcctgcacctgcaacctcaggtactatagtcatgttttcttgtattccagtgtctggaatttattccttctggtggattcttggtcttgggacttcaagaatgaagccgcggacactgcggtgagtgttacagttcttaaagatggcgtgtctggagtttgttccttcagatgttcagatgtatccagagtttcttccttccagtgggtttgtggtctcgctgacttcatgattgaagccgcagaccttgcagtgggtgttatagctcataaagatattgccgacccaaagagtgagcaaccagcaagatttattctgaagagtgaaagaacaaagcttccacagcatggaaggggacctgagcaggttgctgctgctggctaggggtggccagcttttattcccctttatttggccctgttccccacatcctgctgattggtccattttacagagcattgattggtccattttacagagtgctgattggtgtgtttacaactttagctagacacagagcgcagattgttgtgtttacaatcctctagctagacagaaaagttctccaagtccccacctgacccagaagcctccagctggcttcaccctctcattcccagtacaattgtttttttcacattctcccgtctgtcttgtaattttctacatctctctatgagaccacttattttttttttctttagtaatagaaacctcaagtttcaatttgggcatgtggcttcccagctaaagcccatatatttttccccagcttccctggcaatcaacattgaggtgtgactaagttctagttagtgggatgagggaaagtgatgtttgaaacacctgggttgtacctttaaaagaaaaggatatgttctccttgctcattttctcccttctcgtcagaacacagacccagaagataattatttttggccatgaggacaaggcctgtactttagctggtggcagagcaaaagttagaagaaacctgtgtccttaacaccatgaggcatcctatcaccctcagaccacttgtgctcagacatgagaaagcttgttaagcaattgctgttaatgggaatcttttgttacagcaggtgaatttatatcctagagaagctgctcttccttagaatgcatttccctttgttcttatcaaattctcattattgaaggttcaaaacaggtcctactgctgcgtccgggctttacactactccccttctctgaactgccatcatacctgggcatgtgatatgagtgtagtataaaatgagattgattccagcagccacacatgaaaaataagactgctaaccttacagccacttaaaaagaaactgattcctaaatggtgaagaaacagaacataagagctacaggaattcagagatggggtaagcagtgaggactgaaaggatcaggagatatcattatttatctcattagttttcaaactgagactgtgtacaggaagactttccaagggttatttagggaaacattgtttcaagggaattaatttgctactcctcatctggattaattctttcctaaaattcctcttccgaaaaaaaaaaagccgttgaaacaattcttcttcctttctccctttctcaatctgtcctttatcttacaaaagaaaagtgtgacctgactcacttctcatcttattattcatttatctgctgggtgcataaaacacccaggttgtcaaacaactttgtgctggcaaagccttcaatcaagatataacagtagacttcatgtctttccctgacttacacatatattcctgttaagggcaaacatggctttaagaatggaaatttcagcccatgtgggatattctgtgtacataaatatggtacagtgagcagtgtttttttttttaatttgtaatttcatttattgaggaaaatataagtagagggcagttttcagtcccaccgttagtccttctttcccagatcctgattccacttttgtccgtctgacgagtcactttctatcctgatcttatccgactgaccagatctggacctgtccgatcccgatcccgatcccacatcgaaccgatcgcgacaccga

chr22: 29065782-29065621(-)chr10:101386662-101387432 ctgcag chr10:101381857-101386670 (Unmapped)

ctgcag chr10:101381857-101386670 (Unmapped) (Primer gap) chr22: 29065782-29065621(-)chr10:101386662-101387432 ctgcag

Consensus sequence for insertion at chr10:107557372

ttgttgtacttcgttcagttacgtattgctaaggttaatgggaaacacgatagaatccgaacagcaccttctatggccattttatggaatgacaaactaagttcaatactgatagagttgtattctgtgtatacatataatagagtatatactgtgtgtataaaaaattcaaacatatggaaaaacttatcaacagcagcacaaatccataacaattaattctgtacctagccccggaaccacaggtacacccagtacaatgtgtagcaatagttaaaacttctggaaagaaaacaaacttctaaggttgcctcataatcaggtctctcctctaacatgattttgaatgacacatataaaaaagaatgacctctagtggttagatgccaaacctacagtgctgtaattaagaaccagatcttaatgggtggggggtgggactgtattcaagcagttattatacaaagcagcatagagcatcctaatagagaacacacaccacccaactttctctcttataacttatgattatactggaaaggcatcaaaagttcgcatatctcatatagaaacctagaattagatcaggggcttgtagcttctttctcaatatgacactatacacagtatttgctcatacttctgcttaaaattcccagttactagacacattgtactggtaatttatctttattttcttcctgcataataataaactctgtttttctttgagaataatttttcccatgtgcctaatttagggaaagggagtgggagataagagcatattattctacatctcagtgtaaaaaaaagtgaaatgtggacaatcacttcctcttcccttgcagctagaatctaagctcatgaactagagtaaatagtcatcttcctccccagtaattcaaatctggaatgagggttggtgattgatagtggcagttaggtacatgcccgaaggaagtatctagaagtagaggcaatgtctagcagggttttgcagtggttatgtccaatgcatgtaatgtcactgaactgtacacttaaaatagtttaaatgagagattttttgttatatataacaaaatctactacaaaaaattgatcgacttcactctcttatttggaaaatgatgaaacctagacagaattttcctttgggccttgtcccaggcataagcatgttatgatctcaatagagattcattgggggtggaaacacagttaaatcaccctgctccctgctgtgttgtgaccacttgggaaaaagaaaggaagagtctaatggggattaacttcctggcaaaagctggccagagaactcagcaagtcctcccacttgcctccttaatctaaacttgagtaagcccagacttaagccaagaatggcaggcagagaatttggaactaagacagcaatgacctcctggatcccagaagcaaactctgttggatttgcttatcacatggagtaaggcaaactatctctttctaagagtaggatacccaagagacccaagaggaaaaaaaagaacaaggggaccaggaggaagatgaccccattgtcttggtatcagatagtggctctctctgcctgccactctaataaagcttcacagatgtcaggttctctccaggctcaatcatcacacaaggagctcccacacaccacaatcaatgaggagagacatgtaaaagttgaaggggtactgtaaataaagagaaaataaagattcctgttgaaaaaaaaattttattttttatttgctatcactttattattatactttaagttctagggtacatgtgcacaacgtgcaggtttgttacataatatacacatgtgccatgttggtgtgctgcacccattaactcgtcatttacattaggtatatctcctaatgctatccctcccctccgcaaccccacaaccgaggccccggtgtggtgttccccttcctgtgtcaagtgttctcattgttcaattcccacctgagtgagaacatgtggtgtttggttttttgtccttgtgatggtttgccgagaatgatggtttccagcttcatccatgtccctacaaaggacatgaactcatcctttttatggctgcatagtattccatggtgtatatgtgcctcaacaggtgctggagaggatgtggcaaaaaaaacagcctctaacctttccttttcctgtgccttctcagatatacagcaaaccttttacttcttattaacacacgatgttggggaacattgtcttttcttctttaccctgtagcccatcatcttctgtgggcaccaacaatttcccatgtttactcgaaggctgttcacataaagtgaaaccatttacactgcacgctgtaaaggaaggaggttatggcccttggatccaagctgaataattcagacacagtctcattcctacacatcactctcccttatttaaccctgcattgttattttttatttttttttttttgagagagtcttgctctgttgcccaggctggagtgcaatggtgcaatctcggctcactgcaacctctgcttcctgggttcaaatgattctcctgcctcagcctccccagtagctgggactacaggtgttcgccaccatgcctggctaatttttgtattttttttagttgtgatggggttttaccatgttggtcaggctggtcttgaactctgacctcaaatgatctgcccacctcggcctcccaaagtgctgagcttgcggatatgagccaccgcacccagcctcctgcattgttgactaattgatttatatgtctatgacccacctatcctgagccccaactttagagcaagatcctaaaggggagcatagcatgttggttggctgtcgagaccttctttccacaacagtccatgccagcacaccctgtggattccataaatgcctatgctctgatccatttattaagtttctgtgcatgcctagggcagggcagtcccttgccctgaacccattatttaataacaatctatagattccgagctgctggtggcagggatgctttctcacaggtcccatgggctttggggggaagattttcaagaagattaggagatgaatttgttttgaaagacagtacagagttagccttgactgcaaattggacattttattgcatttgtctgatggataatcacagataaagactttgatctttatcccatggcaacaataagaaaaggcaggaactctgtgtactatcttttatttcttttctctgcagttctgggaaaattgagcctatttaaatgctcagaagagtctctccttgtgctcttaaatgttgctgggcgttaaccactctgtctttgtccccgacaataccatgataagatggagatggggagggactgtgaagctcttctctctcagcagaaagactaatggatggggaagaataagaatgttgaaaggaaagaaaaggaagaatagaggattaaatgctgagtgtcatggaaaagatcaagggaagaattacaacagcagtgcagcatggccgtcccaatgaatgcaaagagatcagattggggctttattcttcagacacaagatgctgatggtctgtggccccagattgtcaacatcaggacctttactcttcagatagattctccccatgatcaaaccaagtgaagcattccacaattaaacttgtgaactgactcaaaaaaaaaaaaaaatgggcatgtggagaagctgaaatggaattcagggagtttgatgagtaccagatgaagcttcttccctccacgatgtatttaactgactccactggctatagaatcactatgtttgttgatcatgtttatcaagggcagaaagtaactttcactataaaatgaagactataataactcttatttatccttacaaaggagttggccaaatgccagttccttcaaggcttgaatgaagtctggtgcactagagcggatgtgtgcaggtggtacacataggtaccagggtgcaagcacacagagcccaccattctaggcagctgggctatgctgctgagccacaagtgtctgaccctatttggacaaagagacttaaatgtagacattatttaccaccatgtccaaaggggattacatgcagaatcatttcattttctatgttaacttgtgggatactttatatgattagttcaagtctaatacatttgtagtcttcattctgtctttatttccccgtgttttaatgtaaccataacattgtaactagcctggacctcatgacttttggagtcttatgatgcctgtaaatatgatgtgcgcatgtgcacgcatgcatggatgaacaaccaggatcttaaagtggagattctgtgctgaaaggtcagttacctggaatgtagaatgattatggtttgtctgtgcttgttattttcttttttttttttttttttttttttttttttttttttttttttttttttttttttttttttttttttttttttttttttttttttttttttttttcacattctgaatcttcaggtcttcagatcaagatcttggaatctttgttatctggcgggttattttcagttctatcgtgtttcccctaaccatgggcaatgcaatgatgaatcta

RC:

tagattcatcattgcattgcccatggttaggggaaacacgatagaactgaaaataacccgccagataacaaagattccaagatcttgatctgaagacctgaagattcagaatgtgaaaaaaaaaaaaaaaaaaaaaaaaaaaaaaaaaaaaaaaaaaaaaaaaaaaaaaaaaaaaaaaaaaaaaaaaaaaaaaaaaaaaaaaaaaaaaagaaaataacaagcacagacaaaccataatcattctacattccaggtaactgacctttcagcacagaatctccactttaagatcctggttgttcatccatgcatgcgtgcacatgcgcacatcatatttacaggcatcataagactccaaaagtcatgaggtccaggctagttacaatgttatggttacattaaaacacggggaaataaagacagaatgaagactacaaatgtattagacttgaactaatcatataaagtatcccacaagttaacatagaaaatgaaatgattctgcatgtaatcccctttggacatggtggtaaataatgtctacatttaagtctctttgtccaaatagggtcagacacttgtggctcagcagcatagcccagctgcctagaatggtgggctctgtgtgcttgcaccctggtacctatgtgtaccacctgcacacatccgctctagtgcaccagacttcattcaagccttgaaggaactggcatttggccaactcctttgtaaggataaataagagttattatagtcttcattttatagtgaaagttactttctgcccttgataaacatgatcaacaaacatagtgattctatagccagtggagtcagttaaatacatcgtggagggaagaagcttcatctggtactcatcaaactccctgaattccatttcagcttctccacatgcccatttttttttttttttgagtcagttcacaagtttaattgtggaatgcttcacttggtttgatcatggggagaatctatctgaagagtaaaggtcctgatgttgacaatctggggccacagaccatcagcatcttgtgtctgaagaataaagccccaatctgatctctttgcattcattgggacggccatgctgcactgctgttgtaattcttcccttgatcttttccatgacactcagcatttaatcctctattcttccttttctttcctttcaacattcttattcttccccatccattagtctttctgctgagagagaagagcttcacagtccctccccatctccatcttatcatggtattgtcggggacaaagacagagtggttaacgcccagcaacatttaagagcacaaggagagactcttctgagcatttaaataggctcaattttcccagaactgcagagaaaagaaataaaagatagtacacagagttcctgccttttcttattgttgccatgggataaagatcaaagtctttatctgtgattatccatcagacaaatgcaataaaatgtccaatttgcagtcaaggctaactctgtactgtctttcaaaacaaattcatctcctaatcttcttgaaaatcttccccccaaagcccatgggacctgtgagaaagcatccctgccaccagcagctcggaatctatagattgttattaaataatgggttcagggcaagggactgccctgccctaggcatgcacagaaacttaataaatggatcagagcataggcatttatggaatccacagggtgtgctggcatggactgttgtggaaagaaggtctcgacagccaaccaacatgctatgctcccctttaggatcttgctctaaagttggggctcaggataggtgggtcatagacatataaatcaattagtcaacaatgcaggaggctgggtgcggtggctcatatccgcaagctcagcactttgggaggccgaggtgggcagatcatttgaggtcagagttcaagaccagcctgaccaacatggtaaaaccccatcacaactaaaaaaaatacaaaaattagccaggcatggtggcgaacacctgtagtcccagctactggggaggctgaggcaggagaatcatttgaacccaggaagcagaggttgcagtgagccgagattgcaccattgcactccagcctgggcaacagagcaagactctctcaaaaaaaaaaaaataaaaaataacaatgcagggttaaataagggagagtgatgtgtaggaatgagactgtgtctgaattattcagcttggatccaagggccataacctccttcctttacagcgtgcagtgtaaatggtttcactttatgtgaacagccttcgagtaaacatgggaaattgttggtgcccacagaagatgatgggctacagggtaaagaagaaaagacaatgttccccaacatcgtgtgttaataagaagtaaaaggtttgctgtatatctgagaaggcacaggaaaaggaaaggttagaggctgttttttttgccacatcctctccagcacctgttgaggcacatatacaccatggaatactatgcagccataaaaaggatgagttcatgtcctttgtagggacatggatgaagctggaaaccatcattctcggcaaaccatcacaaggacaaaaaaccaaacaccacatgttctcactcaggtgggaattgaacaatgagaacacttgacacaggaaggggaacaccacaccggggcctcggttgtggggttgcggaggggagggatagcattaggagatatacctaatgtaaatgacgagttaatgggtgcagcacaccaacatggcacatgtgtatattatgtaacaaacctgcacgttgtgcacatgtaccctagaacttaaagtataataataaagtgatagcaaataaaaaataaaatttttttttcaacaggaatctttattttctctttatttacagtaccccttcaacttttacatgtctctcctcattgattgtggtgtgtgggagctccttgtgtgatgattgagcctggagagaacctgacatctgtgaagctttattagagtggcaggcagagagagccactatctgataccaagacaatggggtcatcttcctcctggtccccttgttcttttttttcctcttgggtctcttgggtatcctactcttagaaagagatagtttgccttactccatgtgataagcaaatccaacagagtttgcttctgggatccaggaggtcattgctgtcttagttccaaattctctgcctgccattcttggcttaagtctgggcttactcaagtttagattaaggaggcaagtgggaggacttgctgagttctctggccagcttttgccaggaagttaatccccattagactcttcctttctttttcccaagtggtcacaacacagcagggagcagggtgatttaactgtgtttccacccccaatgaatctctattgagatcataacatgcttatgcctgggacaaggcccaaaggaaaattctgtctaggtttcatcattttccaaataagagagtgaagtcgatcaattttttgtagtagattttgttatatataacaaaaaatctctcatttaaactattttaagtgtacagttcagtgacattacatgcattggacataaccactgcaaaaccctgctagacattgcctctacttctagatacttccttcgggcatgtacctaactgccactatcaatcaccaaccctcattccagatttgaattactggggaggaagatgactatttactctagttcatgagcttagattctagctgcaagggaagaggaagtgattgtccacatttcacttttttttacactgagatgtagaataatatgctcttatctcccactccctttccctaaattaggcacatgggaaaaattattctcaaagaaaaacagagtttattattatgcaggaagaaaataaagataaattaccagtacaatgtgtctagtaactgggaattttaagcagaagtatgagcaaatactgtgtatagtgtcatattgagaaagaagctacaagcccctgatctaattctaggtttctatatgagatatgcgaacttttgatgcctttccagtataatcataagttataagagagaaagttgggtggtgtgtgttctctattaggatgctctatgctgctttgtataataactgcttgaatacagtcccaccccccacccattaagatctggttcttaattacagcactgtaggtttggcatctaaccactagaggtcattcttttttatatgtgtcattcaaaatcatgttagaggagagacctgattatgaggcaaccttagaagtttgttttctttccagaagttttaactattgctacacattgtactgggtgtacctgtggttccggggctaggtacagaattaattgttatggatttgtgctgctgttgataagtttttccatatgtttgaattttttatacacacagtatatactctattatatgtatacacagaatacaactctatcagtattgaacttagtttgtcattccataaaatggccatagaaggtgctgttcggattctatcgtgtttcccattaaccttagcaatacgtaactgaacgaagtacaacaa

**chr10:107557435-107560841 atgcat chr10:107556545-107557372 chr22:29065912-29066032

atgcat chr10:107556545-107557372 chr22:29065912-29066032 (primer gap) **chr10:107557435-107560841 atgcat

Consensus sequence for insertion at chr12:33100097 (Twin priming)

cggtacttcgttcagttacgtattgctaaggttaatgaaacacgatagaatccgaacagcaccttctatggccatttatggaatgacaaactaagttcaatactgatagagttgtattctgtgtatacatataatagagtatatactgtgtgtataaaatttacccaattattatacctaagtacccggccttattaaatatatgttcctcaatatcaaattgtactaaaatcaggaaactaagctcctagtagagaactggtcaaagggaagatctttgtggagtcagcagtttcctctgtctacaatttggcaaaataatctcataagcacaagaaggttaatatgcttgcattaaaaatgttaagttacttataatcaaatttaaatgtaagttcaaatgccttaacatgttcacattacttttttaattcacaaaaacgtcctttactttcttaataaacgtgctttaactttactctgtggacttgcctcaaattctttcttgtgtgagacccaagaaccttctcttgggtctggattggaccctttccagtaacaatactacaagaaatgctggcttgacatgcagtagtttaaaaatgtatccataccatttgattcaaaaggtgttcaaaagaatgttttttaggaacactgaattttttttcagatcagatttcccactagatggtagtgttacaacatcaaaatccttcacaataatggcctagcagctctaattcaattccacagacatttactgagtgcctgctgtgtgtaagatactgtctaattctatgtaggctaatgactgctgaaacaaggatccaggccttttcattcccaagcaatgctgtttcccttataccctgcatacgcacttaaagaaagcaactgcccagtgtccttagctaagagtctttgcaaagctatatacttataaccttgcgaagaaaatgttctaaggtttatacacaagaaggcacatctgttttttgagaatgcaaagtaggtgcacaacagctccacagaaaccttaagaaaatgtttaatgagctcctagatcccaggaaggtggggagatagtctattctagaaagagttcagctcaggaaaaacagagcaggcctaaaagcagttataacacatatgattaatacatatactctgggttacagtgtgatgcattctcctaagtaaccaaggaacctctacttcaaggaatctcatggaaagaaattataacaattaacttattgggcatattgtggagggactgatgtcagatttaaaatttaaactttatttagtaggtggtaggaaactataattgctcttcatctcactctgatatgcagattcatttgttattatccatctttcctctctaaaatgtaaattccattgcagttgagactttgttttttgttttatcgctgttattgccagcacccagaacctggcacctgataaattcttaaatatttgttcattgttggatggattactcagaggttctcaaactttctttgttcgtagtgttctttgtgccttagagactttttatggcacctctaggcaaaacaaaaaagaaaacaaaaacaacacaaaattaaccgttctattgatttccaaacagtactgtacttgcttttttaaaagtcacagtaaccgtagaaactcagcttcacaaaaatttgacatcaatgaaaggaatgtagtgcgatctcatggtgaaactgatttactgtaggctagtagttcatgcagggcctgacagatgtcaagatatgcttcacctgaaaatttaaaatagcttgtagtagccatgcaagttttctgctgtgccctttggcaccttggtgcacaatttgaaataaaaatagtatttttgagtccctatgtctttactcttcgtctataggacatatttctatggtcctacaaaatttagtacttctagtgagatttggctttttttcagctgctctagtcgctgggcataagccattgaatatatcacaaagacaaaactgatttgtgtgttaaagagtaccaaaataggccaggcgtggtggctcacgcctgtaatcccaacattttgggaggctgaggtggatggatcacaaggtcaggagttggagactggcctggccaatatggtgaaaccccatctctactgaaaatataaaaattagccagacgtggtggtgcgcacctgtagtcccagctacttgggaggctgaggcaggagaattgcttgaacccaggaggtagaggttgcagtgaaccaagatcgtgccactgactccagcctgggcgacagagcgagactccatctcaaaaaaaaaaaaaaagtaccaaaataagtgcatgagatggatcaatttatcctgataaccataccagtgtaattcgatcaacatttttatctgatcttattacaacaatgtgtcatgtagaacactattctatgagatgctccaagaaaaaagagaccaatattcaaataaaggtgtgaaatctgcatattactgtatttgtcctcagtaaaaagtgacattaattataagtcttgttgttgatttaataacttttggagaaaagggtggggcgtggtggctcatacctgtaatcccagcactttgggaggcagaggccatcaaatcacctgagccctggagttcccagaccagcctaggcaacatagcaaaaccctctctttacaaaaaacacacaaaaaaaaaacttagctgggcatggtggtgtgcatttgtagtcccagctacttgggaagctgagttggggatgatcacctaagcctgggaagtcaagtctgcagtgaactgttattgtgcccactccagtgtgagtgacagaatgagaccatctaaaaaacaaacaaaaaaaacctttgagaagccgtatatttttttttagagggcttattttttttttactttttgggttaccaagttactcgaccacaatgcctttttcatatatcacctattcatatattgcaactccaatgatcatactgtcaagagacatttgaaccagagtgactccatattgaataggggctcagtaaatgaggctgagacctactgggctgcattcatagaaaggttaaggcattcttagtcacaggatgagataagaggtcggcacaaggtacaggtcataaagaccttgttgataaaacaggttgataaagaccttgttgataaaagaagctggctaaaacccaccaaaaccaagatggcgatgagactgacctctggtcatcctcactgctactactccaccagagaccatgacagtttacaaatgccatgggtcagaaattaccctatatggtctaaaagtggaaaaacctcagttcagggtgcccacctttctggaaaactcataaataaaccaccttgtttagcatataatcagaataaccataaaatggtcaaccaacagccgatggggctatctgcctaatcaggtaagccattctttattcctttactttcttttttttttttttttttttttttttttttttttttttttttttttttttttttttttttttttttttttttttttttttttttaagatattaaagtctttattgcccccattttattttcagtaatacaaggttatctggagtgggattacttgatctggcgggttgctgttcagttctatcgtgtttcctattattaaccttagcaatgcgtaaact

chr22: 29066032-29065937(-)chr22:29065445-29065733 chr12:33100084-33100830 atgcat chr12:33097745-33100097 polyT chr22: 29066118-29066035(-)

atgcat chr12:33097745-33100097 polyT chr22: 29066118-29066035(-) (Primer gap) chr22: 29066032-29065937(-)chr22:29065445-29065733 chr12:33100084-33100830 atgcat

Consensus sequence for insertion at chr14:79638932

ttgttgtacttcgtttcagtattgttaaaggttagctagggaaacacgatagaatcgaacagcaccttctatggccattttatggaatgacaaactaagttcaatactgatagagttgtattctgttcttaaactataaaaatgaataaatactatctaaatcaaaagactatggttttgagtggtgacaatttaatatcaaaagtaattcccactacatttctaagtaaaatctccctctggtgtctttttttaatttcccagtgttcttaaaattcctatggaagaataagtatctgaggatagccaagaaaaatataagtaagaagactttgagtaagaactatgctttataattctaatataaaaccattgtaataaaaataatgtatgaacctggcatgaaaagtgggcaaatagattaaaggacaagaattgtatggctcagaaattttccccagcatacatggaaatttaatcacttccaaagctgttatcacaatttagtggagagatggaaagtgttttttttggcaaatggtagaaagataaccaaccatccatttagaagaaaataatgttggtgcctatctcacatcataaacagaaataaattccaagtgaattgaagacttaaagtttaaaaaaattattaaaaatattggaagaaaatttggactagagatacaatctagtgattggttgaaacattgctaacaaaacaagaatctatatgctataatcttaagagaaaaaaggtcaacatataaaaagtttaagagaaatttaggccaggcacggtggctcaatcctgtaatctcagcactttgtaaggccaatgtgggaggatcacttgagcccaggagttggagaccagcctgggcgacatgatgaaccctatctttacacaggcacacacgcatagctgggcatggtggtgtgtgcctgtaatctcaactattcagaagactgaggtgggaggctgcttgagccaggaggtaaaaggctacagtgggcccgtgtgttcatgccccctgcactccagcctgggtgacagagcaagaccttgtctcaaaaaaaaaaaaaaaattacaataaaagatatcattgccaaattcaatatgtaaaatatatatttggaaatgtaataaaatgtgtccatgagaattacaagtagttataaccaaaacccaagtaactgtggcttggacaaattagatgtcttaatttttgacatgtaataagaagttggaaggtaggcaatcagacctcatacaatgactccatgtggtaatcacaaactgaggctcattctacctgtctccttagctgtcctgactatataggctttcatcctcatatttgtcacttcatgttcactgaatgaagcttctccaccttaaggcaggaaataaaagaaaacaggaaggtgaaagcaaaaaaatgtatatgtgggagctaagtttgtatcttttaaaagaaagagaccagaagtctcttctagtaaattatcagttatatatctttatccataaatgtgtctatcccagttgcaaggacaactggttaatatgttgttttgtaattgtttttgtttctctaatctgggcatatttgcaactttagcattagagttattttactatggggagaagacaatattaattccagaaggtggctagcagtgcctgccacacaaatgaaaggtagcgttaatgtctagactctacctaaaatttttgcaaattgataactgaggaaaacaacccaatagaaaaaattggtgataaatatgaataaacaattcacagaagaaaaaattcagattatccttaaaccaatgaaaagatgcccaacctccttcatcgtcagacaatttcaaatttaagtaacaatgagatgttttgtattcatatgattggcatgaatttaaagtgtggtaactcctattgccgtttgagatgtagaaaaaattttaaattgttacagctttttttttaaaagattaacttttgaatggcagtttctattgaaaagttttatatatatatatatatatatatatacacacatatatatgctttgatcagaaatcctaattatgagactctcatagaaataaaaataccagtatctagctatacatacccaatattgcatcatattttgcggtggtttaaaaaaaaaaaagaggaaaagattaagtaaaatgttcactaatagaaaaatggaattaagtaaattatagtaaatccctatcagggactattatgcagtcattttgcagaatggacttgatctgtcttcatctaaaagcagatgacttagaaatatttccacaatgtattgttaagtgaggaaatgagatagtaatggagaaagataaagaatgatgcaggttattaatctgggtggccagcgcttggagagaggaggggagcaataggggaatatggaatggggaaagcaaaagaaatcaaattttagatatgtacacaagtaaaataacttagaattcagatttccttttttttttttttttttctgaggagtctctctctgttgcccaggctggagtgcagtggtgccatctcggctcactgcaagctccatctcccgggttcacgccattctcctgcctcagcctcccgagtagctgggactacaggcgcccaccaccatatgcggctaattttttgtatttgtactagagacagggtttcactgtgttagccaggatgctctcgatctcctgacctcgtgatcccgcctgcctcggcctcccaaagtgctgggattacaggcatgagccaccgtgcccagcccagatttcttttttaaagacctgaaataaggaagagaaatcatagcataaactaagtttaacaagatcagtgtgggataaagaacagaagattgagaattttttttggagctgttcctgattagatgttagaagattttggttatcaccaatccttttcagaaaaattttataaacttacaagtatccattgcacgtgctttacatttgttttatatcaatatgactgtaaactttatagagatctttttaatacatgaataggacatcttattttttttaaggcaggtaaatgtgcttttctcacttcaaacaaattatgtccacaagctgaataatgagaaggtacttcatagtgtatcacactgccgaattacacgttcactgttttcatccttgaaaaccgtgggctgcttcagagtgaaacagtcttcactgcttataactgagttccaattttttcccagccatctgattcccaagcccagagcttcggcttctgggttctgttgctggcagtgaggaagtgggatagcacattgtttaaatcacagcacttctctgtttcctggctaagttacaaaacagtgttgtagtcgtgaaactgaatatcatcagcacacattgtggcatgcacagagcatgcacgactctgtagtagcgctctaggcaaacttatgatggcttttaaaagagaggtgttgttgcttcctttaggaagtttatgaactaaaaggaagacaaaaaccaaaccaaaccaaacagaaagcaatgcataatgacactatcctttggaatgcaggaaaaaaaattccaaatgacaatttgtcttaaatgtagacagtgctttaaaatatatgcctgatcataagagtggatatagtatttaagtgtgtcagaactttcttgctcttctaccttcctcctttttttggttattgttgaaaatatatgggggaaaaaatggcaaaagaaataggccctacatctgaattttaagttcctgttttttgagagaaagaaatattttgtttattttatttgtttttttttctttcccattagtaaaataattccaactaaggtaaagtttagtggaactaaactttttttactgtgtcacaactctgactttcaagagtaaaggaagaagtaaatgtaataccccagtacacctaaaaccaggattaatgttttactttatcttgcaacagtactaaaattaaaaacaaaacaagacacattttagagatatgatgacagtgagcatagaaaacctcttttattgatggagagcatattttaaaatatcttctcacctttagtctttgggttagtctcagtccctctgcatagagacaaacaatacatgaagaactatttgggaaattgctataaagaaacaataacttgctagggaaaaaaaacaattaactgtgaagcatgtatccctgaatctgaatcatggccataatacttattagacccccaaatttgagcttccattgccttacttgtagaatggagaaaaaattcctcttgaggtgtcagtaaagaagatgaatgtatcctcatgtttattctgatcagattattttatatttttgccttatggcatttacctttaacaattttgaatatctctgaaagaaaggagaaaggatggaaggtacgaagagggtgaaaaggaaggaaggaaggaaaggtctttgttttccttacctggcacaataaatattaataaatcacaatagatagcaaaatctaaaatacctaggagtacatttaacaaaatttatatttttttttttttttttttttttttttttttttttttttttttttttttttttttttttaaagatattaaagtctttgttgccccatttttattttcagtaatacaaggtaaaatggtgggattacttgatctggcaggtgctgttcggattctatcgtgtttcctattaaccttaataatacgtaaccc

chr22: 29066032-29065974(-)chr14:79638931-79642399 atgcat chr14:79637893-79638932 polyT chr22:29066121-29066035(-)

atgcat chr14:79637893-79638932 polyT chr22:29066121-29066035(-) (Primer gap) chr22: 29066032-29065974(-)chr14:79638931-79642399 atgcat

Consensus sequence for insertion at chr18:1233989 (Twin priming)

ttattacttcgttcagttacgtattgctaaggttaaacagacgactacaaacggaatcgacagcacctcccaaaaaatatacaattactggcaaaaaatgttattattaaactgatgaaaatataaaaggtttattactggattaaataagtttttccatatgcttgaagattttatatacacagtatatactctattatgtgtatacacagaatacaactctatcagtattgaacttggtttgtcattccataaaatggccatagatggccagatcaaataatcctctcattttaccctgtattactgaaataaaaatggggggaggcaaacaaaataaagactttaatatctttaaaaaaaaaaaaaaaaaaaaaaaaaaaaaaaaaaaaaataatgaaattaaggcaaaattaagaaattctttgaaactaaaggagaaagataaaccataccaggaatctctgccacacagctaaagcaagaattaagagaaatttgtaacactaagtgcccatatcaagaagagttacaagagatcacaaattggcaacatctaacatcacaacaaaagaaccagagaataatgggcatactaatcccaagctagcagaagacaagtaaccaaagtcagagctgaactgagaggagattgagactcagacaaccattcaaaagatcaataaatccaggagttggtttttttttttgaaaaaaattaataagatataaaggctagctagactaataaaagagaagatcaaaaataacacaattagaaatgacaaagaggatgttaccactgacacaaaaaaatacataaaataactatcagagactactataaacacttctatgcaaacaaactagaaaatctaacagaaatggacaaactcctgaaacatacaccttcccagactgaaccaggaagaaactgaatcctgaacagacaataacaagctccaaacttgaatcagtaatagcctaccaaccaaataacccatgaccagattctagcaggattctagcacatgtacaaagaagagctggtaccactccctactgaaactattcaaaaaaaaaaattgaaggaggatgctctcccaattcattcaatagaggctttcattatcctaataccaaaacctttcagataaacaacaaaaaaagaaaacttcaggtcaacatctttgatgagcattgatgcaaaaatcctcaacaaaatactaacaaaaccaaatccagctgcacatctaaaagctaatccaacatgatcaaaatgggctttatcctggtatgcaaggtttaggttcaacatacacaaatcaataaatgtgattcatcacaaaaaaacaaattcaatacaaaaaactacacaattatctcaatagatgcaaaaagaaaacttgtaaaattcaacatcccttatgttaaaaactctaataaactaggtattgaagaaacatacctcaaaataataaaagccatctatgacaagcccacactaacaacatacttaatgggcaaaaactgacacaagatgaggatgatatctctcaacactcctattcaatatggtattagaagtctgaccaggagaaatcagacaagagaatgaagtaaatggcatcaaaataggaagacaggcatcaaaataggaagtcaaactatcgctgtttgcagactacgtgattctatatctagaaaattccatagcaaataaaaaaaatccttaagctgttagagcaactacagcaatgatacattggataaagaaaatgttagcacatatacaccatgggaatactatgcagccataaaaaggatgagttcatgtcctttgcagggacatggatgaagctggagaaaccattatcctcaacaaactaacacaataacagaaaaccaaacactgcatgttctcactcataagtgggagttgaataatgagaacacttggacacaggaaggggaacatcacagaccagaataactattggcagatggagagctagggagggatagcattaggagaaatacttaatgtagatgatgggttgatggatgcagcaaaccataatggcttttatgtacatctatgtaacaaacctgcacattctgcacatgcattctactaatttacttgacattctgcaagtacagtacattttatatggcatatgttataatttgacaagattttttgataatattaacttccttaggcacattataagtcaatatgcaatgatataggaccgaatagtttccttgcctttatttttctgaatctggcatttaatagaataaatctgtggtgatgctcccagccttttatgtaggtaagcatatttatgccaggaccaatacaatttattttttaaaatatcaggtaagaattttcaaatataatcttaaattcatagtgaacattggaagtgtatgtaccaaaataagtttgaagtattctcaaatctcactgattgtgaagacctcaaaagaaagaggcaaatattgtatttagttacacagaaatataaagggtctttaatgtaaatatttcgattaaagatgccatggactgctaatttgtatccccttgatttacatgttgaggtcccagcttccaacgtgattttatttggaggtggggcctttgggagggagtaattatgtttagatgagctcattgaggatggagccttgtaggactagtgtccttataagatgaggaagagacaccagagctctctctcccccctgccccacaagtgagaagacacagtgagaaaggtggccatatgcaaactaagaagagccatatcattaagaactgaatgtattggcaccttgatacaaggacttccagctcccaaaactgtgagaagcaaatgtctgctatttaagccacccaatatgtcatattttgttatagcaatccaaactgactaaggaggactgagtgtcaaactgttcttgtggacaagtagaaaacagacttctagacctagatagatttctaagagccagaaataggtataagctagattgcaagagtgaaatagtttggatgtttgtccctgcccaaaaaatatcatgttgaaatataagtcctcaatgttggaagcagggcctggtgggaggtaattggatcattggggcagatttctcatgattggtttagcaccatcctcttcgttgctgtaactcgcaataagtaagttttcatgtgatcctggtttgtttaaaagtatgaggctcctcacctctcttattcagctttttgccatgtgacatggctgtttcccctttgccttccaccatgagtaaaagctcctgaggcctcctcagaagctgagcagatgccaacccgatgcttgtacaacctgcaaaagcataagccaattaaatctcttttttctttataaattacccagtcgcaggcatttcttcatagcaatgcaagaatggcaaaaaaatacataagattaaagcctgaaaccaaaggaaactcaaagactgcctggagtggtaacttccagaatggtgcatgaaaaactctgtgaatcctttccctaattaaacagccataactgataaaaattatagaagaaaaatcacttaaactcttgaatgctttggaattgtcctatagaagagcagggctatggagatttttttccccaggtggaggagcattctgtagagcagggaggttccacagcttatttcaaaggaactgactttgttttggtatagttcaataatattctgggcactattgaaaaaaaatagataagtaccaaagtacctggatttgtttttaatcaggcatggtagtaagatggaagacaccagagattggttgtcatgaaaataataagtttattatatttatagattcctggaaatgggaggcatggtacacaccaaacaaggaggccacacaggaaacactgggttgttcaggacctgaggaagcagggaagagatgtgactagagcctttattgtgtgttttctatgagggaaaagagtaagggatggtaaacaggcttaagattaaagttagtttaaataaatttcagtgagctctgggatataaaaatgtccctagacttctgatacttggctctgggatgattaggacagatgaatagtaggagtcagtttgagagccttctaagagaggtggttaggggtatggagctatgaattggctggtttgcatttgaaaagtgtgtgctcccacatgagttgtttactatctctagtaattgattaaccctggttatgggcattccctacaggatcaagcccccagatgaaaatgttggaatacagaaagacatagttaatgagctgtaagcaattaagaaagaggctggtagtaccaggagagctaaatgatagttagccagaagattaataaaagaatcagggaaagagatagttgttggagtttggaacaacctcaggctggcttcaaaagataacctggcaaaggggcccaaatttaattggatcagactatggagcagcaacttatgtccagggaattaatggaaataataacatgatcagccaccagttaatggaggctaaccactcagtgtgacgctaacagaaatatgtagcttactagagatatcagagaaagagatagtaaaaattgccttttaaagaactttcatcctaagatgtatatgtatatgcccaagattcccctctgagactgacatcagggaatgcacactggaaatagagattcatagaagtactccaactaagtaactaaacaaataaacaaacaaacaaaataacaacaacccatagccccaagaaagaaagaataattattcagagttcaatattatataaaatggccagttttcaacaaaaattatgagatgtgcatagaaacaaaaactgtaagaattgtagaaaaatttaaaaggcaatagtacctgcctttgagagaatctacacgttagactcaacagacaaatttcaagcaaatattaaaaatatgttcaaaggaactaaggaaccgtgccttaaaagaaaaaggtatgatgacaatgtttcatcatgtaagaatgtcaataaagaaaataaattacttttttaaaactgagtgaaaattatccaaagttgaaaactacaataaacaaagttaataatttaatagagggaagctcaatggtatgttttgaactggcaaaagaatgtgtgtgatgttgaagactgattattagagaatatgaaatatgaagaatatgaaaaacagaacgaatagaaatgaacagagtttcaaagaaatgtcagacaacgtttagtgcaacaacatagatatagtaggactacaggaaggagaggagaggaagaaggggtacaaaataaaaagacataatggagatatagtggctggtaactttctacatttaataaaaaacaaatattaatctacaaatccaagaactcaggaaacttcaaattaggataaatgcaaagatcatcacatcagaaaattatgttcaaaatctttgaaagacaatggcaaagaaaattctgaaagggaacaagagaaaaaacaacccatcacttcaagtaaactgtaatatttaatattaatatttaatatttgctgaaatatttgcagctgacttctcattagaaacaatggaggacagagcagtggaaagtcaaattcaaagtaaaataataaattgaaaggaaaaaaaaaaacctaatcgagaaataatcatatatcaaagaaaactaactttaaaaaaatgacagtgaaataaaagcatcttcaaataaacaaaaatggacagaatttgttgtaagcagatattccttgtaacaaacattaaagttctaaatgaaagtgaactcagaccatagttcaagtccgcatgataaaatagagtgccagtaaaagtacttgtttatgtaattatttttttttttttttttttttttttttgagacggaagtctcgctctgtcgccagagctggagtgcagtggtgcgatctcggctcacttcaagctccgcctcccgggttcacgccattctcctgcctcagcctcccagtagctgggactacaggcgcccgctaccacgcccggctaatttttttttgtattttttagtagagacggggtttcaccttgttagccaggatggtctcgatctccctgacctcgtgatccgcccacctcggcctcccaaagtgctgggattacaggcgtgagccaccgcgcccggcctgtaattattttaaaaactatatttgcaaatttcttaactgacttaaaagcaatctgtatgaataatatgtatataactgtattattgggcctgtaacacatagaaatgtgatatattagaaaatatcacaaataaggtgaattaagagcaaaacagtattggaataattaaatgataccaatagtacctggaattcactggaacaaatgaagagaactaaaaaggttaatataacaaggcataaatatttaattgctcttctttcttttctcagcttctttaaaggacataaaatttgtacaaagtaataattataacaatatatcattaggtttttttatatatatataatctagaaaacactaatttagtacaaagaagcagaagaataagaatgaacaaaacttctgagtaatatgggattataaaaaagaggccaaatctatgaatcactggtgtccctgaagagatagggaggaatagaagcaatttagacaatatatttcaggttatcatccactgagaacctcccaacctaactagagaagccagatttaaccaggaaatgtgaaaaacacctgcaaaatacttcacaagaagatcagccccaagacatataatcatcaagattcccaaaggtcagaaaaaaaaagttaatggcatctaagacagaaagatcatgtcacctacaagaagcacctcagactaacagtggacctctcagcagaaaccccacagagtgaagatatcaaggtccctatattcaacattcttaaagaagaaattccaacacagaattttgtatccagtcaaactaaacataagtgaaagaagacataagattctttatatagacaaacaaatgttacaggaactcatttaccccaccagacctgccttacagaaggctcctgaaagaagcactaaatatggaaaggaaatattattaccaatactacaaaaaattcacttaagtacacagaccacattgatactgtaaagcaaccacacaaacaaatctgcataacaaccaaacaacataatgactaggatcaaatccacacatatcaatactaaccttgaatttaaatggactaaatgccccaattaaagcacagcgtgtgaaagctggataaagaaaagcaagacccaatggtatgctgtcttcaaagagacccatctgacttgcaatgatatctatgagctcaaaataaaaagcattggaagagctaagaaagaagcaaatggaaagaaaaaaaaaaaacaatggttgcaatcctaatttttacacaaagcagacattaaaccaaaaagatcaaaaaaagacaaagaagggcgttacacaattggtaaggctgcaatttaacaagaagatgtgactatcctaaatatatgtgtgcactcacaggagggcatcagattcataatagcaaaagttcttagaaactatcagagagacttagactgccacacaaccccattgacagtattacacaaatcactgaggcagaaaagaattaataaacatattcaggacctgacctcaatactggacaaaatggttacagtagaaatccgcaaaactctgcccaaaaacaacagaatatacattcttctcatctgctgcaaggcacgtacttataaaatcgaccacccaattggacactaaaacattcttcagcaaatgtaaaagaaccaaaatcataccaaccactctctggacaacagcacaataaaaatagaaatcaagactaagaaaaatatgctcaaaaccatgcaattgcatagaaatttacaacctacttctgaatgactttttgcgggtaaataatgaaattaaattggattataagtaacttaacatttttaatgcaagcatattaaccttcttgtgcttatgagattattttgccaaattgttggacaagagaaactgctgactccacaaaagatcttacagcgatcattctctactagggcttagtttctgattttagtacaatttgttattgagaggtacatatatttaataaaaccgaaggtacttaggtataatagtgggtaagtgtactctttatttggccctaactggtcacacttctgggaatattcttcccctatcagagattcaatgtaagttcaaataccttaacatgttcacattacttttttttattctaaaaaacgttcttatctatctttattttaaagatgttttcccacatacattcttagtgaaaggtgctgtcgattccgtttgtagtcgatctgttttattagcaatgcataat

RC:

attatgcattgctaataaaacagatcgactacaaacggaatcgacagcacctttcactaagaatgtatgtgggaaaacatctttaaaataaagatagataagaacgttttttagaataaaaaaaagtaatgtgaacatgttaaggtatttgaacttacattgaatctctgataggggaagaatattcccagaagtgtgaccagttagggccaaataaagagtacacttacccactattatacctaagtaccttcggttttattaaatatatgtacctctcaataacaaattgtactaaaatcagaaactaagccctagtagagaatgatcgctgtaagatcttttgtggagtcagcagtttctcttgtccaacaatttggcaaaataatctcataagcacaagaaggttaatatgcttgcattaaaaatgttaagttacttataatccaatttaatttcattatttacccgcaaaaagtcattcagaagtaggttgtaaatttctatgcaattgcatggttttgagcatatttttcttagtcttgatttctatttttattgtgctgttgtccagagagtggttggtatgattttggttcttttacatttgctgaagaatgttttagtgtccaattgggtggtcgattttataagtacgtgccttgcagcagatgagaagaatgtatattctgttgtttttgggcagagttttgcggatttctactgtaaccattttgtccagtattgaggtcaggtcctgaatatgtttattaattcttttctgcctcagtgatttgtgtaatactgtcaatggggttgtgtggcagtctaagtctctctgatagtttctaagaacttttgctattatgaatctgatgccctcctgtgagtgcacacatatatttaggatagtcacatcttcttgttaaattgcagccttaccaattgtgtaacgcccttctttgtctttttttgatctttttggtttaatgtctgctttgtgtaaaaattaggattgcaaccattgttttttttttttctttccatttgcttctttcttagctcttccaatgctttttattttgagctcatagatatcattgcaagtcagatgggtctctttgaagacagcataccattgggtcttgcttttctttatccagctttcacacgctgtgctttaattggggcatttagtccatttaaattcaaggttagtattgatatgtgtggatttgatcctagtcattatgttgtttggttgttatgcagatttgtttgtgtggttgctttacagtatcaatgtggtctgtgtacttaagtgaattttttgtagtattggtaataatatttcctttccatatttagtgcttctttcaggagccttctgtaaggcaggtctggtggggtaaatgagttcctgtaacatttgtttgtctatataaagaatcttatgtcttctttcacttatgtttagtttgactggatacaaaattctgtgttggaatttcttctttaagaatgttgaatatagggaccttgatatcttcactctgtggggtttctgctgagaggtccactgttagtctgaggtgcttcttgtaggtgacatgatctttctgtcttagatgccattaactttttttttctgacctttgggaatcttgatgattatatgtcttggggctgatcttcttgtgaagtattttgcaggtgtttttcacatttcctggttaaatctggcttctctagttaggttgggaggttctcagtggatgataacctgaaatatattgtctaaattgcttctattcctccctatctcttcagggacaccagtgattcatagatttggcctcttttttataatcccatattactcagaagttttgttcattcttattcttctgcttctttgtactaaattagtgttttctagattatatatatataaaaaaacctaatgatatattgttataattattactttgtacaaattttatgtcctttaaagaagctgagaaaagaaagaagagcaattaaatatttatgccttgttatattaacctttttagttctcttcatttgttccagtgaattccaggtactattggtatcatttaattattccaatactgttttgctcttaattcaccttatttgtgatattttctaatatatcacatttctatgtgttacaggcccaataatacagttatatacatattattcatacagattgcttttaagtcagttaagaaatttgcaaatatagtttttaaaataattacaggccgggcgcggtggctcacgcctgtaatcccagcactttgggaggccgaggtgggcggatcacgaggtcagggagatcgagaccatcctggctaacaaggtgaaaccccgtctctactaaaaaatacaaaaaaaaattagccgggcgtggtagcgggcgcctgtagtcccagctactgggaggctgaggcaggagaatggcgtgaacccgggaggcggagcttgaagtgagccgagatcgcaccactgcactccagctctggcgacagagcgagacttccgtctcaaaaaaaaaaaaaaaaaaaaaaaaaaataattacataaacaagtacttttactggcactctattttatcatgcggacttgaactatggtctgagttcactttcatttagaactttaatgtttgttacaaggaatatctgcttacaacaaattctgtccatttttgtttatttgaagatgcttttatttcactgtcatttttttaaagttagttttctttgatatatgattatttctcgattaggtttttttttttcctttcaatttattattttactttgaatttgactttccactgctctgtcctccattgtttctaatgagaagtcagctgcaaatatttcagcaaatattaaatattaatattaaatattacagtttacttgaagtgatgggttgttttttctcttgttccctttcagaattttctttgccattgtctttcaaagattttgaacataattttctgatgtgatgatctttgcatttatcctaatttgaagtttcctgagttcttggatttgtagattaatatttgttttttattaaatgtagaaagttaccagccactatatctccattatgtctttttattttgtaccccttcttcctctcctctccttcctgtagtcctactatatctatgttgttgcactaaacgttgtctgacatttctttgaaactctgttcatttctattcgttctgtttttcatattcttcatatttcatattctctaataatcagtcttcaacatcacacacattcttttgccagttcaaaacataccattgagcttccctctattaaattattaactttgtttattgtagttttcaactttggataattttcactcagttttaaaaaagtaatttattttctttattgacattcttacatgatgaaacattgtcatcatacctttttcttttaaggcacggttccttagttcctttgaacatatttttaatatttgcttgaaatttgtctgttgagtctaacgtgtagattctctcaaaggcaggtactattgccttttaaatttttctacaattcttacagtttttgtttctatgcacatctcataatttttgttgaaaactggccattttatataatattgaactctgaataattattctttctttcttggggctatgggttgttgttattttgtttgtttgtttatttgtttagttacttagttggagtacttctatgaatctctatttccagtgtgcattccctgatgtcagtctcagaggggaatcttgggcatatacatatacatcttaggatgaaagttctttaaaaggcaatttttactatctctttctctgatatctctagtaagctacatatttctgttagcgtcacactgagtggttagcctccattaactggtggctgatcatgttattatttccattaattccctggacataagttgctgctccatagtctgatccaattaaatttgggcccctttgccaggttatcttttgaagccagcctgaggttgttccaaactccaacaactatctctttccctgattcttttattaatcttctggctaactatcatttagctctcctggtactaccagcctctttcttaattgcttacagctcattaactatgtctttctgtattccaacattttcatctgggggcttgatcctgtagggaatgcccataaccagggttaatcaattactagagatagtaaacaactcatgtgggagcacacacttttcaaatgcaaaccagccaattcatagctccatacccctaaccacctctcttagaaggctctcaaactgactcctactattcatctgtcctaatcatcccagagccaagtatcagaagtctagggacatttttatatcccagagctcactgaaatttatttaaactaactttaatcttaagcctgtttaccatcccttactcttttccctcatagaaaacacacaataaaggctctagtcacatctcttccctgcttcctcaggtcctgaacaacccagtgtttcctgtgtggcctccttgtttggtgtgtaccatgcctcccatttccaggaatctataaatataataaacttattattttcatgacaaccaatctctggtgtcttccatcttactaccatgcctgattaaaaacaaatccaggtactttggtacttatctattttttttcaatagtgcccagaatattattgaactataccaaaacaaagtcagttcctttgaaataagctgtggaacctccctgctctacagaatgctcctccacctggggaaaaaaatctccatagccctgctcttctataggacaattccaaagcattcaagagtttaagtgatttttcttctataatttttatcagttatggctgtttaattagggaaaggattcacagagtttttcatgcaccattctggaagttaccactccaggcagtctttgagtttcctttggtttcaggctttaatcttatgtatttttttgccattcttgcattgctatgaagaaatgcctgcgactgggtaatttataaagaaaaaagagatttaattggcttatgcttttgcaggttgtacaagcatcgggttggcatctgctcagcttctgaggaggcctcaggagcttttactcatggtggaaggcaaaggggaaacagccatgtcacatggcaaaaagctgaataagagaggtgaggagcctcatacttttaaacaaaccaggatcacatgaaaacttacttattgcgagttacagcaacgaagaggatggtgctaaaccaatcatgagaaatctgccccaatgatccaattacctcccaccaggccctgcttccaacattgaggacttatatttcaacatgatattttttgggcagggacaaacatccaaactatttcactcttgcaatctagcttatacctatttctggctcttagaaatctatctaggtctagaagtctgttttctacttgtccacaagaacagtttgacactcagtcctccttagtcagtttggattgctataacaaaatatgacatattgggtggcttaaatagcagacatttgcttctcacagttttgggagctggaagtccttgtatcaaggtgccaatacattcagttcttaatgatatggctcttcttagtttgcatatggccacctttctcactgtgtcttctcacttgtggggcaggggggagagagagctctggtgtctcttcctcatcttataaggacactagtcctacaaggctccatcctcaatgagctcatctaaacataattactccctcccaaaggccccacctccaaataaaatcacgttggaagctgggacctcaacatgtaaatcaaggggatacaaattagcagtccatggcatctttaatcgaaatatttacattaaagaccctttatatttctgtgtaactaaatacaatatttgcctctttcttttgaggtcttcacaatcagtgagatttgagaatacttcaaacttattttggtacatacacttccaatgttcactatgaatttaagattatatttgaaaattcttacctgatattttaaaaaataaattgtattggtcctggcataaatatgcttacctacataaaaggctgggagcatcaccacagatttattctattaaatgccagattcagaaaaataaaggcaaggaaactattcggtcctatatcattgcatattgacttataatgtgcctaaggaagttaatattatcaaaaaatcttgtcaaattataacatatgccatataaaatgtactgtacttgcagaatgtcaagtaaattagtagaatgcatgtgcagaatgtgcaggtttgttacatagatgtacataaaagccattatggtttgctgcatccatcaacccatcatctacattaagtatttctcctaatgctatccctccctagctctccatctgccaatagttattctggtctgtgatgttccccttcctgtgtccaagtgttctcattattcaactcccacttatgagtgagaacatgcagtgtttggttttctgttattgtgttagtttgttgaggataatggtttctccagcttcatccatgtccctgcaaaggacatgaactcatcctttttatggctgcatagtattcccatggtgtatatgtgctaacattttctttatccaatgtatcattgctgtagttgctctaacagcttaaggattttttttatttgctatggaattttctagatatagaatcacgtagtctgcaaacagcgatagtttgacttcctattttgatgcctgtcttcctattttgatgccatttacttcattctcttgtctgatttctcctggtcagacttctaataccatattgaataggagtgttgagagatatcatcctcatcttgtgtcagtttttgcccattaagtatgttgttagtgtgggcttgtcatagatggcttttattattttgaggtatgtttcttcaatacctagtttattagagtttttaacataagggatgttgaattttacaagttttctttttgcatctattgagataattgtgtagttttttgtattgaatttgtttttttgtgatgaatcacatttattgatttgtgtatgttgaacctaaaccttgcataccaggataaagcccattttgatcatgttggattagcttttagatgtgcagctggatttggttttgttagtattttgttgaggatttttgcatcaatgctcatcaaagatgttgacctgaagttttctttttttgttgtttatctgaaaggttttggtattaggataatgaaagcctctattgaatgaattgggagagcatcctccttcaatttttttttttgaatagtttcagtagggagtggtaccagctcttctttgtacatgtgctagaatcctgctagaatctggtcatgggttatttggttggtaggctattactgattcaagtttggagcttgttattgtctgttcaggattcagtttcttcctggttcagtctgggaaggtgtatgtttcaggagtttgtccatttctgttagattttctagtttgtttgcatagaagtgtttatagtagtctctgatagttattttatgtatttttttgtgtcagtggtaacatcctctttgtcatttctaattgtgttatttttgatcttctcttttattagtctagctagcctttatatcttattaatttttttcaaaaaaaaaaaccaactcctggatttattgatcttttgaatggttgtctgagtctcaatctcctctcagttcagctctgactttggttacttgtcttctgctagcttgggattagtatgcccattattctctggttcttttgttgtgatgttagatgttgccaatttgtgatctcttgtaactcttcttgatatgggcacttagtgttacaaatttctcttaattcttgctttagctgtgtggcagagattcctggtatggtttatctttctcctttagtttcaaagaatttcttaattttgccttaatttcattattttttttttttttttttttttttttttttttttttttaaagatattaaagtctttattttgtttgcctccccccatttttatttcagtaatacagggtaaaatgagaggattatttgatctggccatctatggccattttatggaatgacaaaccaagttcaatactgatagagttgtattctgtgtatacacataatagagtatatactgtgtatataaaatcttcaagcatatggaaaaacttatttaatccagtaataaaccttttatattttcatcagtttaataataacattttttgccagtaattgtatattttttgggaggtgctgtcgattccgtttgtagtcgtctgtttaaccttagcaatacgtaactgaacgaagtaataa

chr22: 29065782-29065677(-) chr22:29065387-29065677 chr18:1233975-1239597 (with 320bp deletion in between) atgcat chr18:1232259-1233989 polyT chr22: 29066121-29065836(-)

atgcat chr18:1232259-1233989 polyT chr22: 29066121-29065836(-) (Primer gap) chr22: 29065782-29065677(-) chr22:29065387-29065677 chr18:1233975-1239597 (with 320bp deletion in between) atgcat

Consensus sequence for insertion at chrX:108351909

cttgttgcttcgttacgtattgctaaggttaaacagacgactacaaacggaatcgacagcacctttcactaagcatgtatgtggaaaacatcttaaaaataaagatagataagaacgtttttttgtgaattaaaaagtaatgtgaacatgttaaggtatttgaacttacatttagaattggattataagtaacttaacatttttaatgcaagcatattaacaaccttcttgtgcttatgagattattttgccaaattggacagagagaaactgctgactccacaaagatcttccctttgaccagttctctactaggagcttagtttcctgattttttagtacaatttgatattgaggaacatatatttaataaggccgggtacttaggtataataattgggtaaattgtacctttaatgctgaaagatctccctagactcacctctttaggctggttgaatttcatgtttaactgaggatctcagattagcctgggtcttgaactggatcaggaggggggcatccacaagagaaccactccagaaatgtaagctgttccaaaactgtcaagatgagttatctgttcataaaggaattcattgtatggagaaattgtattttcaagaaggcaggattttcatgttaacttgaccatgctaaatgtagcatatccaaaatctgtttgcatgaaaacctcagacagaagcactttctgatgtcctggaattgattctgatatttaaaagactgtaaagagtacagcctgacaagcttcttgacataactggttaaggaacctactttttcatggtggatatgggagaggaatggacaagcagaggatcaggaagacttagataataatccatattgattttgcagggccatactagggtaagactttttttcattgatctagtcttcctctcaagatgtctcaaggaggtcccaggaccaccaccaacctctctatgttagttgttttttcaataaaatatcaaaaaaagcattactgtttctgatgatgtggcctacacattgagatcttaagtttgttccactgtttagaagataaagtaacttccaggaaattttgtgttcaaaatcttcaaaccattgtgtttgaatggttgaaaaggtgaaaagaaaaaaaaatgaccatgatttcctacctaactcacccacagatagaaaacagttatctttaatattttttggtgtatttctttcttatttttttcatgccttttttttttttttttttttttttgagacaggcttgctccatcacccagactggagtgcagtgacatgaacacagctcactgcagcctcgatctcctgggctcaagccatcccctcctctctcagcctcgttagtagctgggactacaggtgcatgccaacatgcctggctaatttttttgtattttcagtagagatggggttttgccatgttggtcaggctggcctcagactcctgacctcaagtgatcctctgccttagcctcccaagtgttgggattacaggcttgagccactgtgcctggcctgaatgctcttaaaagcattaagacggttttaaaattaaatgttttggtgcatggtaaagcacttccaaactactcaagtctctaaaataaaaggtaaatttttatttattcatttccaccctggcttgagtcccacagatagagataacacttatactagtttctactagtatagaaactacttttagtcaagatggattatcaaagaccaaagtagataaaactctaaagatggggaaaaaaaacagaacagaaaaactggaaactctaaaacatagcagcgcctctcctctgccaaaggaatgtagttcctcaccagcaacggaacaaagctggatggagaatgactttgacgagctgagagaagaaggcttcagatgatcaaattactctgagctacgggaggacattcaaaccaaaggcaagaagttgaacactttgaaaaaatttagaagaatgtataactagaataaccaatacagagaagtgcttaaagggagctgatggagctgaaaaccaaggctcgagaactacatgaggaatgcagaagcctcaggagccgatgcgatcaactggaagaagaggtatcagcaatggaagatagaatgaatgaaatgaagcgagaaggaagtttagagagaaaaaaagaataaaaagaaatgagcaaaagcctccaagaaatatgggactatctatagaaaaagaccaaatctacgtctgattggtgtacctgaaagtgatgggagaatgcaaccaatagttggaaaacactctgccggatattatccaggagggacttccctaatctagcaaggcaggccaacgttcagattcaggaaatacagagaacatacaagatactcctcgagaaagaacaactccaagacacataatttgtcagattcacaaagttgaaatgaaggaaaaaaaaatgttaagggcagccagagaaaggtcgggttaccctcaagggaaacccatcaggcgaacagcagatctctcggcagaaaccctacaaaccagaagagtggggccaatattcaacattctaaaagaaagaatttccaacccagaatttcatatcagccaaactaagcttcataagtggaaggagaaataaaatactttacagacaagcaaatgctgagagattttgtcaccaccaggcctgcctaaagagctcctgaaggaagagagcataaacatggaaaggaacagccggtaccagccactgcaaaatcatgccaaaatgctaagaccatcgagactaggaagaaactgcatcaactaacaagcaaaatcaccagctaacatcataatgacaggatcaaattcacacataacaatattaactttaaatgtaaatggactaaacactccaactaaaagacacagactggcttaaaattggataaaagagtccaagacccatcagtgtgctgtattcaggaaacccatctcacgtgcagagacacacataagctcaaaataaaaaggatggaggaagatctaccaagcaaatggaaaacaaaaaaaaggcaggggttgcaatcctggtctctgataaaacagactttaaaccaacaaagatgaaaagagacaaagaaggccattacacatactggtaaagggatcaattcaacaagaagagctaactatcctaaatatatgcacccaatgcaggagcaccaagattcataaagcaagtctgagtgacctacaaagagacttagactcccacacattaataatgggagactttaacaccccactgtcaacattagacagatcaatgagacagaaagtcaacaaggataccagaattgaactcagctctgcaccaagcggacccaatagacatctacagaactctccaccccaaatcaacagaatatacatttttttttcagcaccacaccacagctattccaaaattgaccacatggttggagtaaaaagctctcctcagcaaatgtaaaagaacagaaattataacaaactatctctcagaccacagtgcaatcaaactagaactcaggattaagaatctcactcaaagccactcaactacatggaaactgaacaacctgctcctgaatgactactgggtacataacgaaatgaaggcagaaataaagatgttctttgaaaccaacgaacaaagacacaacataccaaaatctctgggatgcataagcttccctggggcaactcaatctttactgtgtttttcttccataaaaagtcaaaaatttctgagtcacttcattgtctactgagaaaaaaagtgtgtggtttgaagcttatcagaaatagctaatggacaaattctaggctgctaagaaattattgaagctgcatgtatggagtgaacatcaaccacactctttttattatttcctcttaacccaaaagtcactaccaacatttccacttgcttgaaactcaagagcaagattatctatatataaaattctgtataaatttttgagacaactctgttgtaacaagaaatcagaataacctacaagtagctttatggctatgtctttattatttgtactattctcaagtctctctgaggattactttccaaaccaagagatatattctttatatcaatgtcagtaggtaaaacacattagaggtccttgctgagtttctagagcatactaattgaatgctggtatcctgaacctatctgcccctgcaaattctaggttatattggaatcatatccataggaaatttttttttttttttttttttttttttttttttttttttttttttttttttttttttttttttttttttttttttttttttttttttttttttttatttttctcatcagtttaataacatttttacgccagtaattgggtatattttgggtgctgtcgattcaagtttgtagtcgatctgtttgtccttaatacgtaagc

chr22: 29065782-29065437(-)chrX:108351907-108353215(del.)chrX:108357411-108359393 atgcat chrX:108351348-108351909 polyT chr22: 29065893-29065833(-)

atgcat chrX:108351348-108351909 polyT chr22: 29065893-29065833(-) (Primer gap) chr22: 29065782-29065437(-)chrX:108351907-108353215(del.)chrX:108357411-108359393 atgcat

Consensus sequence for insertion at chrY:15633117

ttggtatgcgtttcagttacgtattgctaagttaaacagacgactacaaacggaatcgacagcaccttctatggccattttatggaatgacaaactaagttcaatactgatagagttgtattctgtgtatacatataatagagtatatactgtgtgtataaaaaaattcaagcatatggaaaaacttatttaatcagtaataaacctttatattttctcatcaagtaactttttttgaccaaaattgtatgaaactagaaattaaaatagaaaatttttagaaattttgcaaatatgtagaaattaaacaacatgcttctgaaaataaataataatcgttgaagaaataaaaactaaacaatgtaaatgacaaaaaaaaaactgagttgttttttgaaaaaccaacaaaattgaaaaccttttacctagactatctcatgaaaaaaagagagaagggggatgaggtttaagatggctgactagaagcaacaagtgggcaacactttcataaagacgaaataaagtagcaagtaaatagtagctcttcaagtggatatctaagataccccatcaggattcaccaacgtggcaaaaggactcacagagaacagagcaaacccaggtagcagccacccccagaactgatgtggaactgggagaacctctcacacaatgaaagggtgaatgactgaggggtcccaggggatccacacttcccatatggaccttcaaatcctaggtatgggtaatcttccctgtccccccaggtctctaagaccaacatagagaggtgcctagagtttttgcagagcatcactcaagcccatgaggagctttgccagtcatggagccctgtgaaccctagcaccagctgccataaccacaatagatactgcagaagtttctgctatcttttgttcagctatgccctgccccagaggtggagtctacagaggcaagcaggtcttgttgagctgctgtgggctccaccagtttgaaacttcatgtctgctttgtttacctactcaagcctcagcaatggtggacaccctcccccagcctcacctctgccttgcagttagatctcagacagctgtgctagcagtgagtgaggctccatgggtgtaggatctgctgagccaggtgtgggatataatctcctgatatgccatttgctaagaccactgggaaagcacagtattaggttgggtgtcccaattttccaggtaccatctgtcatgggttccttggctaggaaggaatttcctaaccctttgtgcttcccaggtgaggcattgccctgcctgcttcagctcacactccatgggctacattcactgtccaacaagccccattgagatgaacctggtacctcagttggaaatgcagaaatcacccatcttccacgtcgctcaggctgggagctgtagactggagctgttcctattgcaccatcttggaacccacctacataatttatttttaaacaattatctttggccaaggaacagtaacaatggtggcatcagtccaagattggaaaggcaattaactgggcaaatgtcctcacataagtattttttttttctgtaaagttattattatagcttttgtgcaaggttgtagtttcagtagtgttttgtcatagttctggttatcaggtgtttgtgctaagaatacttccttcaggccctgtctgactcaaattttccacggtttgacaaaagtaactattttgattctaacagctttcacaagacacatacactgaaagtaatgggatggaaaagatattgcctaaaaatgaaaacagaacagaagacattaccacatatttttagaaaaagtagatatttagtcaaaaactgtgaaaaaaaagacaagaaaggtcattaaacagttacaggatcaactaatcaagagattatgacacttgtaaatatgtacccagcattaaagcacctacatatataaaaaaaatattaatagatctgaaatgagatatagatgcaaaagatagtaggggacatcaataccccactttcagcaacagacagattatcagagagaaaatcataaggaaatattgaacttaaactaaactttagactaaaggactaataaaaacagttggaacacttcatgtaatagaagtagaatgtacctcttctcaagcgcacatggaagattttctaagatagataatatattaggccacaaaacaagagttaacaaatttagtattgacattatgtcaagtaactttttttttttttttttttttttttttttttttttttttttttttttttttaaagatattaaagtctttattgccccccattttttattttcagtaatacagggtaaaatgggtaggattacttgatctggcaggtgctgtcgattccgtttgtagtcgtctgtttaaccttagcaatacgtaacccc

chr22: 29066032-29065877(-)chrY:15633103-15633775 ctgcag chrY:15631735-15633117 polyT chr22: 29066121-29066035(-)

ctgcag chrY:15631735-15633117 polyT chr22: 29066121-29066035(-) (Primer gap) chr22: 29066032-29065877(-)chrY:15633103-15633775 ctgcag
